# Size of chloroplasts in Arabidopsis mesophyll cells affects jasmonate biosynthesis

**DOI:** 10.1111/plb.70222

**Published:** 2026-05-11

**Authors:** R. Baral, H. Stellmach, S. N. Kariithi, M. Heilmann, S. Krüger, J. Ziegler, B. Hause

**Affiliations:** ^1^ Department of Cell and Metabolic Biology Leibniz Institute of Plant Biochemistry Halle/Saale Germany; ^2^ Program Center MetaCom Leibniz Institute of Plant Biochemistry Halle/Saale Germany; ^3^ Charles Tanford Protein Center Martin Luther University Halle‐Wittenberg, Institute of Biochemistry and Biotechnology Halle/Saale Germany; ^4^ Biozentrum, Core Facility Microscopy Martin Luther University Halle‐Wittenberg Halle/Saale Germany

**Keywords:** Allene oxide cyclase, *arc* mutants, chloroplast morphology, flowering time, galactolipids, jasmonate biosynthesis, organellar architecture, thigmomorphogenesis, touch sensitivity

## Abstract

- Chloroplasts are highly dynamic organelles whose morphology responds to developmental and environmental cues, yet whether organellar architecture directly influences metabolic capacity remains unclear. Using Arabidopsis *arc3* and *arc5* mutants harbouring defective division machinery resulting in giant chloroplasts, we investigated how chloroplast morphology is linked to the biosynthesis of jasmonates, such as jasmonic acid (JA) and jasmonoyl‐isoleucine (JA‐Ile).
- Quantitative three‐dimensional analysis using confocal laser scanning microscopy, jasmonate determination after wounding, galactolipid profiling and ultrastructural analysis were performed to characterize *arc* mutants relative to wild type, the chloroplast positioning mutant *chup1* and the chloroplast movement mutant *kac1/2*.
- Upon wounding, *arc* mutants accumulated higher levels of JA and JA‐Ile than the wild type, whereas levels of galactolipids enriched in α‐linolenic acid, the primary fatty acid substrate for jasmonate biosynthesis, were reduced. Giant chloroplasts in arc mutants possessed loosely organized thylakoid membranes with expanded stromal regions. Moreover, protein abundance of the JA biosynthetic enzyme allene oxide cyclase was increased in *arc* mutants. Enhanced jasmonate production resulted in a stronger mechanostimulation response in these mutants: Compared to wild type, arc mutants exhibited a more pronounced flowering delay under repeated mechanical stimulation.
- The combination of altered membrane architecture and increased enzyme abundance likely underlies enhanced jasmonate production. Collectively, these findings identify chloroplast morphology as a previously unrecognized factor associated with jasmonate biosynthesis and suggest a link between organellar architecture and hormone biosynthesis during plant stress adaptation.

## Graphical abstract

The relationship between chloroplast structure and hormone biosynthesis capacity is poorly understood. This study shows that mutants harbouring giant chloroplasts produce more jasmonic acid upon mechanical stress than wild‐type plants, suggesting that organellar architecture is a previously unrecognized factor associated with plant hormone biosynthesis and stress adaptation.

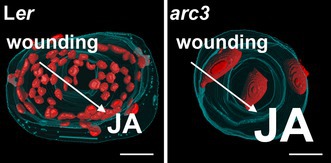

## INTRODUCTION

Chloroplasts are highly dynamic organelles whose division, movement and morphological changes respond to developmental and environmental cues (Jarvis & López‐Juez 2013; Osteryoung & Pyke 2014). Arabidopsis mutants defective in plastid division machinery, such as ACCUMULATION AND REPLICATION OF CHLOROPLASTS3 (*ARC3*) and *ARC5*, exhibit highly enlarged chloroplasts with markedly lower numbers per cell (Pyke & Leech 1994; Robertson *et al*. 1995). The ARC3 protein as a stromal Z‐ring accessory protein functions in conjunction with ARC6 to bind FtsZ proteins and to promote the dynamics of chloroplast Z rings (Maple *et al*. 2007; Zhang *et al*. 2013; Du *et al*. 2025). In contrast, ARC5 encodes a cytosolic dynamin‐related protein that functions late in chloroplast division by mediating the final constriction and separation of daughter chloroplasts (Gao *et al*. 2003; Miyagishima *et al*. 2006). ARC5 assembles into discontinuous rings on the cytosolic surface of the outer envelope membrane and is recruited to the division site through interactions with PLASTID DIVISION1 (PDV1) and PDV2 in a coincidence‐driven, ER‐mediated manner (Miyagishima *et al*. 2006; Ghosh *et al*. 2025).

Chloroplasts of the *arc3* and *arc5* mutants exhibit distinct but complementary division defects: *arc3* chloroplasts show multiple aberrant Z rings and irregular shapes due to defective division site specification, while *arc5* chloroplasts display a characteristic dumbbell shape with initiated constriction but failing complete separation (Gao *et al*. 2003; Zhang *et al*. 2013). Despite detailed analysis of division defects, the metabolic effects of the altered chloroplast architecture in *arc3* and *arc5* mutant plants have been analysed mainly with respect to photosynthesis as *arc* mutants are limited in their photosynthetic competence and display photosynthetic characteristics of low light acclimated plants (Austin II & Webber 2005). Other chloroplast‐localized metabolic pathways remain rather understudied in the *arc* mutants, among them the biosynthesis of defence hormones, such as abscisic acid, salicylic acid and jasmonic acid (JA).

JA and its derivatives, commonly named jasmonates, mediate one of the most important plant defence pathways, that is, responses to mechanical stress, herbivory and pathogen attack and are also involved in developmental processes (Wasternack & Hause 2013). The biosynthesis of JA begins in chloroplasts where a 13‐LIPOXYGENASE (LOX), a 13‐ALLENE OXIDE SYNTHASE (AOS) and an ALLENE OXIDE CYCLASE (AOC) convert galactolipid‐derived fatty acids into 12‐*cis*‐oxo‐phytodienoic acid (*cis*‐OPDA) (Wasternack & Song 2017). Among the four 13‐LOXs of *A. thaliana*, LOX2 is active in wound‐induced JA formation in aerial parts of the plant (Chauvin *et al*. 2016). In peroxisomes, *cis*‐OPDA is then reduced by OPDA REDUCTASE 3 (OPR3) followed by three rounds of β‐oxidation yielding JA (Stintzi & Browse 2000). Recent evidence indicates that cytosolic OPR2 also contributes to JA synthesis through an alternative pathway involving 4,5‐didehydro‐JA (Chini *et al*. 2018; Wasternack & Hause 2018). The final reaction leading to the bioactive form of jasmonates, (+)‐7‐*iso*‐jasmonoyl‐isoleucine (JA‐Ile), is performed by JASMONATE RESISTANT1 (JAR1), a cytosolic enzyme that conjugates JA to isoleucine (Staswick *et al*. 2002). JA‐Ile, the active ligand in most plants, directly interacts with CORONATINE INSENSITIVE1 (COI1), an integral component of the SKP1‐CUL1‐F‐box protein E3 ubiquitin ligase complex, thereby triggering the interaction between COI1 and JASMONATE ZIM‐domain proteins (JAZs) and leading to the degradation of JAZs through the 26S proteasome (Howe *et al*. 2018; Wasternack & Hause 2013). JAZs are the main repressors that inhibit JA signal transduction by binding to transcription factors like MYC2 (Zhang *et al*. 2015). It has been reported that functional jasmonate signalling mediates touch‐induced morphogenesis, since jasmonate deficient and insensitive mutants (e.g., *aos*, *jar1* and *coi1*) are non‐responsive to repetitive mechanical stimulation, which results in delayed flowering of wild‐type Arabidopsis (Chehab *et al*. 2012).

Despite the central role of chloroplasts in jasmonate biosynthesis, the relationship between chloroplast morphology and hormone production capacity has not been well investigated. Chloroplast galactolipids, particularly monogalactosyldiacylglycerol (MGDG) and digalactosyldiacylglycerol (DGDG), serve as major substrate sources for JA biosynthesis, with α‐linolenic acid (18:3) as the key precursor (Wang *et al*. 2018). The prevailing paradigm focuses primarily on substrate availability and enzyme abundance as the key drivers of metabolic output, leaving organellar architecture overshadowed in importance (Sweetlove & Fernie 2013). This substrate‐centric view is supported by studies that show galactolipid availability directly influencing jasmonate biosynthetic capacity (Ibrahim *et al*. 2011; Lin *et al*. 2016; Yu *et al*. 2020). However, emerging evidence from other metabolic systems suggests that membrane organization can significantly influence biosynthetic efficiency independent of substrate levels, as demonstrated in animal steroid hormone production where mitochondrial fusion enhances biosynthesis through optimized enzyme localization (Duarte *et al*. 2012). Similarly, chloroplast membrane dynamics affect photosynthetic efficiency and metabolite transport (Kirchhoff 2019; Johnson & Wientjes 2020; Schwenkert *et al*. 2022). The spatial organization of jasmonate biosynthetic enzymes across chloroplast membrane systems suggests that membrane architecture may influence biosynthetic efficiency, with LOX2 localizing to both envelope and thylakoid membranes, AOS to the inner envelope and AOC as a soluble enzyme within the stroma but in close proximity to AOS (Froehlich *et al*. 2001; Farmaki *et al*. 2007; Stenzel *et al*. 2012). As *arc* mutants exhibit aberrantly altered chloroplast architecture with distinct ultrastructural features (Pyke & Leech 1994), we hypothesized that structural reorganization in giant chloroplasts might influence jasmonate biosynthetic capacity through mechanisms beyond substrate availability.

Here, we employed quantitative 3D morphological analysis to characterize chloroplast architecture in *arc3* and *arc5* mutants in comparison to wild type and chloroplast positioning mutants, such *as kinesin‐like protein for actin‐based chloroplast movement1/2* (*kac1/2*) and *chloroplast unusual positioning1* (*chup1*) exhibiting altered chloroplast localization without major size changes (Oikawa *et al*. 2003, 2008; Suetsugu *et al*. 2010). We investigated the relationship between organellar morphology and jasmonate metabolism through hormone quantification using LC–MS/MS, galactolipid substrate analysis by GC–MS, quantitative gene expression analysis and ultrastructural examination by transmission electron microscopy. Our findings reveal an unexpected relationship between chloroplast size and jasmonate biosynthetic capacity, demonstrating that enlarged chloroplasts enhance hormone production despite reduced galactolipid substrate availability. The enhanced jasmonate biosynthesis translates into amplified thigmomorphogenetic responses, establishing chloroplast morphology as a previously unrecognized feature associated with jasmonate homeostasis that may influence plant environmental adaptation to mechanical stress.

## MATERIAL AND METHODS

### Plant growth, wounding and touch treatments

Seeds from *Arabidopsis thaliana* wild‐type (ecotype Col‐0 and L*er*) and the mutant lines *arc3* (Marrison *et al*. 1999), *arc5* (Pyke & Leech 1994), *chup1* (Oikawa *et al*. 2003) and *kac1/2* (Suetsugu *et al*. 2010) were stratified at 4 °C for 2 days before grown individually in pots containing steam‐sterilized clay, coir fibre and vermiculite for 4 weeks. Plant growth was conducted in Phytocabinets (Percival Scientific, www.percival‐scientific.com/) at a light intensity of 120 μEm^−2^ s^−1^ under short‐day‐conditions (10/14 h light/dark cycle), at 21/19 °C and 65% relative humidity. Microscopical analyses, wounding by inflicting a single wound in the midrib, RNA extraction and hormone measurements were done using leaf No. 8 of 4‐week‐old plants.

For touching experiments including determination of bolting time, two‐week‐old seedlings were gently brushed twice daily approximately 10 times with a soft art paint brush according to (Darwish *et al*. 2022). Touch treatment was applied for 18–26 days until all plants had bolted depending on the genotypes, that means when the inflorescence stem reached 1‐cm height. The rosette area of 31‐ to 35‐day‐old plants was measured using Fiji/ImageJ software.

### Cell wall staining and confocal laser scanning microscopy

Leaf discs (20–25 per genotype) from the 8^th^ leaf of 4‐week‐old plants were collected using a cork borer and stained with 0.01% (v/v) Renaissance 2200 (R2200; Renaissance Chemicals, UK) under gentle vacuum infiltration for 10 min, followed by two washing steps with distilled water. Leaf disks were analysed using a Zeiss LSM900 (Carl Zeiss GmbH, Oberkochen, Germany, http://www.zeiss.com) with excitation/emission at 405 nm/415–450 nm and 633 nm/650–700 nm for R2200 and chlorophyll, respectively. Z‐stack images were acquired using identical settings for all samples and image correction was performed in Zeiss ZEN Blue. 3D reconstructions were analysed using Arivis Vision4D software (Arivis AG, Rostock, Germany), where mesophyll cells were defined as parent objects and chloroplasts as child objects based on their respective fluorescence signals. This parent–child segmentation approach enabled quantification of cell volume, surface area and chloroplast number per cell from a minimum of 50 cells per genotype.

### Hormone measurements

Measurements of *cis*‐OPDA, JA and JA‐Ile were performed using a standardized Ultra‐Performance Liquid Chromatography–tandem Mass Spectrometry (UPLC–MS/MS)‐based method (Balcke *et al*. 2012). For this, 50 mg of powdered frozen tissue was extracted with 250 μl 100% LC–MS methanol supplemented with internal standards (5 ng/sample each of [^2^H_5_]OPDA, [^2^H_6_]JA and [^2^H_2_]JA‐Ile). Following a purification step done by solid‐phase extraction on HR‐XC (Chromabond, Macherey‐Nagel, Düren, Germany, www.mn‐net.com), 10 μl of the eluate was analysed via UPLC–MS/MS, and analyte content was determined relative to the internal standard peak heights.

### RNA isolation and quantitative RT‐PCR analysis

Total RNA was extracted from homogenized frozen plant material using the RNeasy Plant Mini Kit (Qiagen), followed by removal of genomic DNA with the DNA‐free™ DNA Removal Kit (Invitrogen, #AM1906). First‐strand cDNA synthesis was performed using 1 μg of DNA‐free RNA with the RevertAid H Minus Reverse Transcriptase and oligo(dT)_18_ primers (ThermoFisher Scientific™, Darmstadt, Germany, www.thermofisher.com). Quantitative PCR was carried out according to Mekkaoui *et al*. (2025) and using the primers given there. Gene expression was normalized to the housekeeping gene *PROTEIN PHOSPHATASE 2 A SUBUNIT A*3 (AT1G13320) using the 2^−ΔCT^ method (Schmittgen & Livak 2008) and included at least three biological replicates.

### MGDG/DGDG measurements and untargeted lipid analytics

For determination of MGDG and DGDG contents, total lipids were extracted from 50 mg fresh weight tissue using a modified Bligh and Dyer method (Bligh & Dyer 1959) with chloroform:methanol:0.15 M NaCl (1:2:1, v/v/v). After phase separation with chloroform, the organic phase was collected, the aqueous phase was re‐extracted twice and organic phases were combined, dried under nitrogen and dissolved in chloroform. The sample was split and one fifth volume was used for total fatty acid analysis and the rest for analysis for individual galactolipid classes. Galactolipids (MGDG and DGDG) were separated by TLC using acetone:toluene:water (90:30:7, v/v/v). The lipids were sprayed with 0.02% (w/v) primuline solution (acetone:water, 4:1, v/v) and visualized by UV light. For quantification, tripentadecanoin was used as the internal standard. MGDG and DGDG were isolated from the TLC plates and fatty acids were transesterified with 0.5 M sodium methoxide in toluene:methanol (1:2, v/v), extracted with n‐hexane after NaCl quenching, evaporated and dissolved in 50 μl acetonitrile.

GC‐FID analysis was performed on a Shimadzu GC‐2010plus system with a DB‐23 column (30 m × 0.25 mm × 0.25 μm). Operating conditions: split injection (5:1) at 220 °C, helium carrier gas at 1 ml/min, the temperature gradient was 150 °C for 1 min, 150 °C to 200 °C at a rate of 25 °C min^−1^, from 200 °C to 250 °C at 4°C min^−1^, 250 °C for 6 min. FAMEs were identified by comparison with menhaden oil FAMEs as authentic standard and quantified using the internal standard. Three biological replicates were analysed per treatment.

Untargeted lipid analytics was performed following Salem *et al*. (2016). Briefly, frozen leaves (30 mg fresh weight) were homogenized and extracted with 500 μl pre‐cooled methyl tert‐butyl ether (MTBE): methanol (3:1, v/v). An internal standard of deuterated 1‐pentadecanoyl‐2‐oleoyl(d7)‐sn‐glycero‐3‐phosphoethanolamine (15:0–18:1‐d7‐PE) was added into each sample to end up with a concentration of 1 μM. After vortexing (30 min, 4 °C) and sonication (15 min), phase separation was induced with water:methanol (3:1, v/v). Following centrifugation (20,000 × *g*, 10 min, 4 °C), the upper phase was dried under nitrogen and reconstituted in 300 μl acetonitrile:isopropanol (7:3, v/v). Eight biological replicates were analysed per treatment.

LC–MS/MS analysis was performed on an Agilent 1290 UHPLC coupled to a Bruker timsTOF Pro equipped with a VIP‐HESI source. Separation employed a Bruker Intensity Solo C18 column (100 × 2.1 mm, 1.8 μm) with gradient elution from 40% to 99% B over 8.5 min (mobile phase A: acetonitrile:water 60:40, v/v; B: isopropanol:acetonitrile 90:10, v/v; both containing 0.1% formic acid and 10 mM ammonium formate) at 0.45 ml/min. MS parameters: positive ionization mode, m/z 100–1350, ion mobility 1/K_0_ 0.70–1.80 V·s·cm^−2^. Data was processed using Bruker MetaboScape v2025b with the integrated Lipid Annotation Tool for compound classification and structural confirmation. The intensities of the compounds in each sample were then normalized to the intensity of the internal standard in that sample. Compounds showing statistically significant differences between wild type and mutants according to Student's *t*‐test (see Dataset S1) were log transformed and plotted in heatmaps.

### Protein extraction and immune blotting

Extraction of total proteins from leaves, separation by PAGE and immunoblotting were performed according to Mekkaoui *et al*. (2025). Quantification of AOC protein level was done using a rabbit anti‐AtAOC antibody (Stenzel *et al*. 2003b) in relation to β‐actin, which was detected using a mouse anti‐actin antibody (A0480, Sigma‐Aldrich). As secondary antibodies, anti‐rabbit or anti‐mouse IgG antibodies conjugated with alkaline phosphatase (1:4000, Sigma‐Aldrich) were used followed by incubation in the Immun‐Star™ AP (BioRad) substrate following the manufacturer's instructions.

### Electron microscopy

Leaf discs of 2 mm diameter were dissected from the middle of a fully developed leaf of plants at bolting stage and immediately fixed in 3% (v/v) glutaraldehyde in cacodylate buffer (pH 7.2) for 2 h. After post‐fixation with 2% osmium tetroxide, samples were dehydrated in an ethanol series and embedded in epoxy resin (Spurr 1969). Semithin sections (1 μm) were stained with toluidine blue and micrographs were taken using a Leica DMRB microscope equipped with a Leica DFC 450 camera (Leica Microsystems, Wetzlar, Germany, http://www.leica‐microsystems.com). Ultrathin sections (90 nm) were stained with uranyl acetate/lead citrate and observed with an EM 900 transmission electron microscope (Zeiss). Micrographs were processed through PHOTOSHOP 12.0.4 (Adobe Systems, http://www.adobe.com).

### Statistical analysis

The statistical analyses applied to the different datasets as indicated in the figures were performed using GraphPad Prism (www.graphpad.com).

## RESULTS

### Quantitative 3D analysis reveals distinct chloroplast morphology in division and positioning mutants

**Fig. 1:**
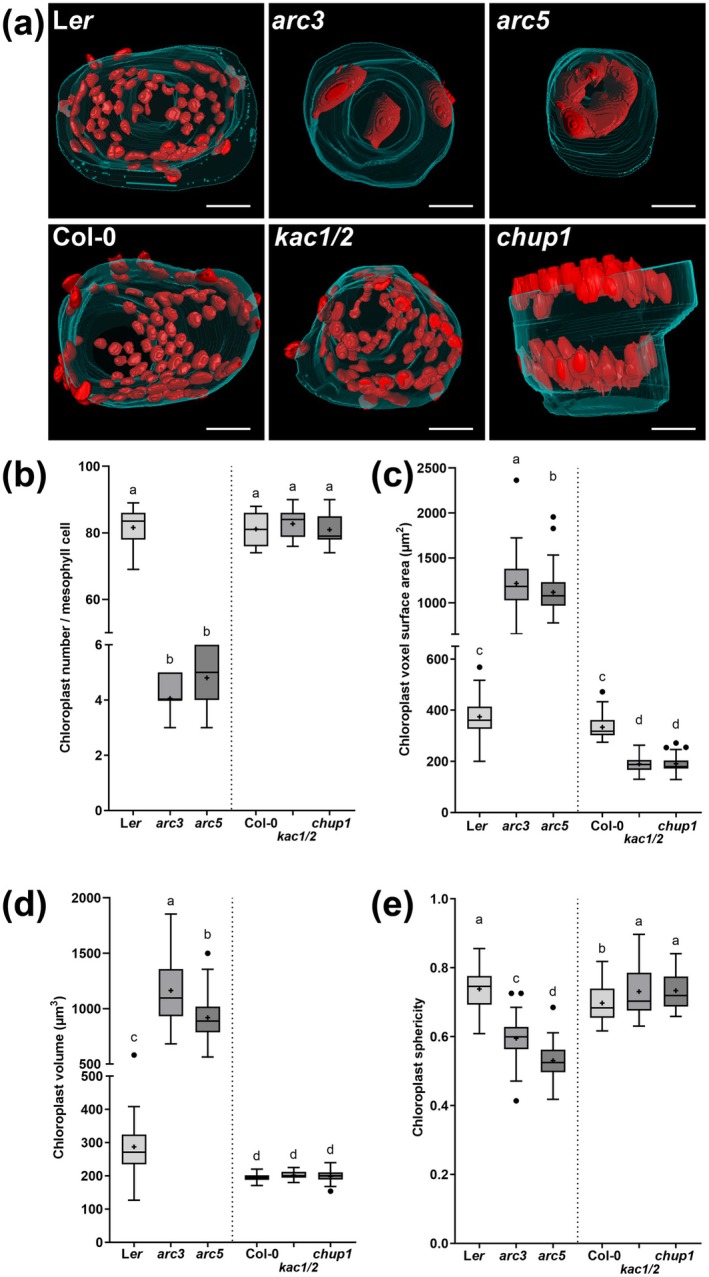
3D analysis of chloroplast morphology in mesophyll cells of chloroplast division and positioning mutants. (a) Representative confocal microscopy images after 3D reconstruction and parent–child segmentation showing chloroplast distribution and morphology in mesophyll cells of L*er*, *arc3*, *arc5*, Col‐0, *kac1/2* and *chup1* mutants. The rosette leaf No. 8 of 4‐week‐old plants was stained with R2200 for visualization of cell walls (turquoise), and chloroplasts were visualized by chlorophyll autofluorescence (red). Bars represent 10 μm. (b) Chloroplast number per mesophyll cell. (c) Chloroplast total surface area. (d) Chloroplast volume. (e) Chloroplast sphericity. Box plots display median, first and third quartiles, with whiskers drawn according to Tukey method. Dots represent outliers. Different letters indicate statistically significant differences between mutants and their respective wild type according to one‐way ANOVA followed by Tukey's HSD test (*P* < 0.05) with n = 50 cells and their respective number of chloroplasts per genotype.

To characterize the three‐dimensional (3D) architecture of chloroplasts in rosette leaves of Arabdiopsis, we employed confocal laser scanning microscopy (LSM) coupled with 3D image reconstruction to quantify chloroplast morphological parameters in mesophyll cells of *arc3*, *arc5*, *kac1/2* and *chup1* mutants as well as their corresponding wild type. Although qualitative chloroplast morphology changes in *arc* mutants have been previously reported (Pyke & Leech 1994; Holzinger *et al*. 2008), quantitative 3D parameters including surface area, volume and sphericity have not been comprehensively characterized. Chloroplasts from *arc3* and *arc5* mutants were analysed in comparison to *A. thaliana* cv. Landsberg *erecta* (L*er*), while those from *kac1/2* and *chup1* mutants were examined in comparison to *A. thaliana* cv. Columbia‐0 (Col‐0). Consistent with their defective chloroplast division phenotype, mesophyll cells in *arc3* and *arc5* mutants exhibited greatly reduced chloroplast number accompanied by proportionally increased individual chloroplast size compared to wild‐type L*er* (Fig. 1a). Quantitative analysis revealed that chloroplast number per mesophyll cell in *arc3* and *arc5* mutants in comparison to wild type appeared to be approximately 20‐fold reduced from about 80 to 4 chloroplasts per cell (Fig. 1b). Concomitant with these data, 3D morphometric analysis showed that the enlarged chloroplasts in both *arc* mutants had significantly increased surface area and volume compared to wild‐type chloroplasts (Fig. 1c,d). Interestingly, despite increase in individual chloroplast size, the total chloroplast volume per mesophyll cell appeared to be drastically reduced in *arc* mutants (around 4500 μm^3^) as compared to the wild type (around 23,000 μm^3^). To determine chloroplast shape regularity, we employed sphericity analysis, where a value of 1.0 indicates a perfect sphere. Chloroplasts from *arc3* and *arc5* mutants exhibited reduced sphericity values indicating more irregular chloroplast morphology compared to wild‐type chloroplasts, which showed a more regular, globular architecture (Fig. 1e). In contrast to the division mutants, the chloroplast positioning and movement mutants showed distinct morphological patterns. Consistent with previous findings (Oikawa *et al*. 2008), mesophyll chloroplasts from the *chup1* mutant exhibited aberrant subcellular localization, clustering predominantly at the upper and lower side of a cell rather than displaying the uniform distribution within the cytosol observed in wild‐type cells (Fig. 1a). Morphometric analysis revealed that chloroplast number per mesophyll cell remained unchanged in both *kac1/2* and *chup1* mutants compared to wild type (Fig. 1b), whereas chloroplast surface area was significantly reduced in both mutants (Fig. 1c). Notably, chloroplast volume in *kac1/2* and *chup1* mesophyll cells was similar to wild‐type chloroplasts (Fig. 1d), indicating that the decreased surface area was compensated by increased sphericity, resulting in more compact, spherical chloroplasts (Fig. 1e). These findings suggest that while KAC1/2 and CHUP1 do not affect chloroplast volume, they play critical roles in determining chloroplast shape and surface topology.

### Wounding induces elevated jasmonate production in genotypes harbouring enlarged chloroplasts

**Fig. 2:**
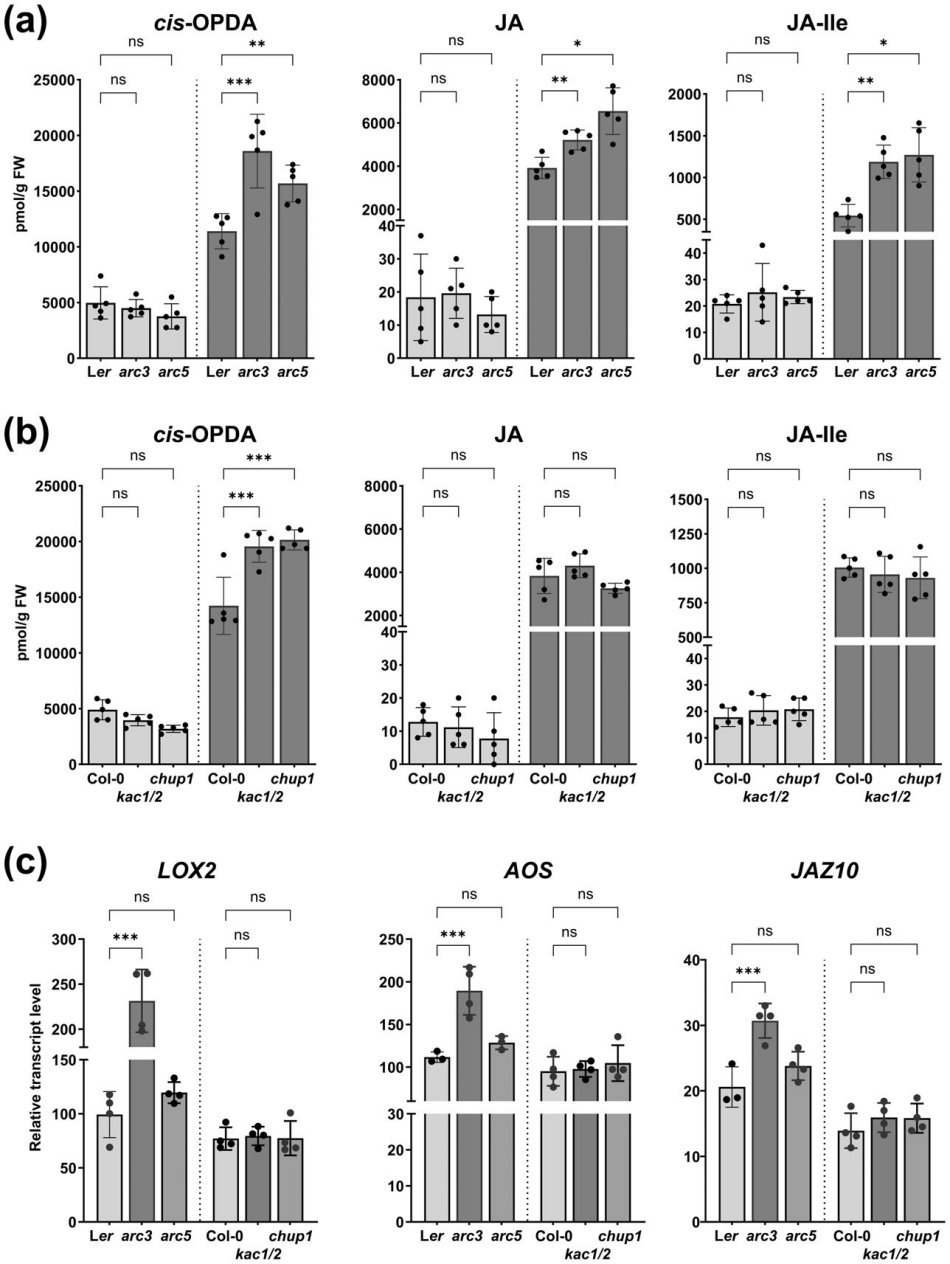
Enhanced jasmonate production in *arc* mutants after wounding. (a, b) Levels of *cis*‐OPDA, JA and JA‐Ile under basal conditions (light bars) and 1 h after wounding (dark bars) in (a) L*er*, *arc3* and *arc5* and (b) Col‐0, *chup1* and *kac1/2* rosette leaves of 4‐week‐old plants grown under short‐day conditions. (c) Transcript accumulation of *LOX2*, *AOS* and *JAZ10* in wounded plants normalized to *AtPP2A*. Data are presented as means ± SE (n = 5 biological replicates and n = 3–4 biological replicates with 3 plants pooled per replicate for (a–c), respectively). Asterisks indicate statistically significant differences compared to respective wild‐type levels (*P < 0.05, **P < 0.01, ***P < 0.001; one‐way ANOVA followed by Tukey's HSD test). ns, not significant.

To investigate the impact of chloroplast morphology on jasmonate biosynthesis, we quantified *cis*‐OPDA, JA, and JA‐Ile in the 8^th^ rosette leaf of 4‐week‐old plants at basal levels and at 1 h after wounding, since this point is commonly used to evaluate wound‐induced accumulation of JA (Mekkaoui *et al*. 2025). Under basal conditions, JA and JA‐Ile levels were at the limit of quantification in all genotypes tested. In contrast, *cis*‐OPDA was readily detectable and showed similar levels across all genotypes (Fig. 2a,b).

Mechanical wounding induced substantial increases in all three compounds across genotypes but revealed significant differences between chloroplast morphology mutants. In comparison to wild‐type leaves, leaves of the *arc3* and *arc5* mutants showed significantly elevated *cis*‐OPDA, JA and JA‐Ile levels at 1 h after wounding, with JA and JA‐Ile levels being about 1.5‐fold and 2.2‐fold higher, respectively (Fig. 2a). In contrast, the chloroplast positioning and movement mutants *kac1/2* and *chup1* showed no significant differences in JA and JA‐Ile levels compared to wild type before and at 1 h after wounding (Fig. 2b). Wounding resulted, however, in an enhanced *cis*‐OPDA accumulation in *chup1* and *kac1/2* leaves compared to wild type (Fig. 2b).

To further elucidate the effects of enhanced jasmonate production in *arc* mutants, we analysed transcript levels of genes being regulated by JA/JA‐Ile, among them genes encoding JA biosynthesis enzymes, such as *AtLOX2* and *AtAOS* (Mekkaoui *et al*. 2025), and proteins involved in JA‐signalling, such as *AtJAZ10* (Acosta *et al*. 2013). Determination of transcript accumulation using RT‐qPCR demonstrated that wounding of *arc3* leaves resulted in significantly elevated transcript levels of *AtLOX2*, *AtAOS* and *AtJAZ10* in comparison to wounded wild‐type leaves (Fig. 2c). Wounded leaves of the *arc5* mutant showed a slight but non‐significant upregulation of all three transcript levels in comparison to wild‐type leaves only (Fig. 2c). In contrast, wounding of leaves from the chloroplast positioning and movement mutants *kac1/2* and *chup1* did not result in any significant differences in the transcript accumulation of the tested genes compared to the respective wild type.

**Fig. 3:**
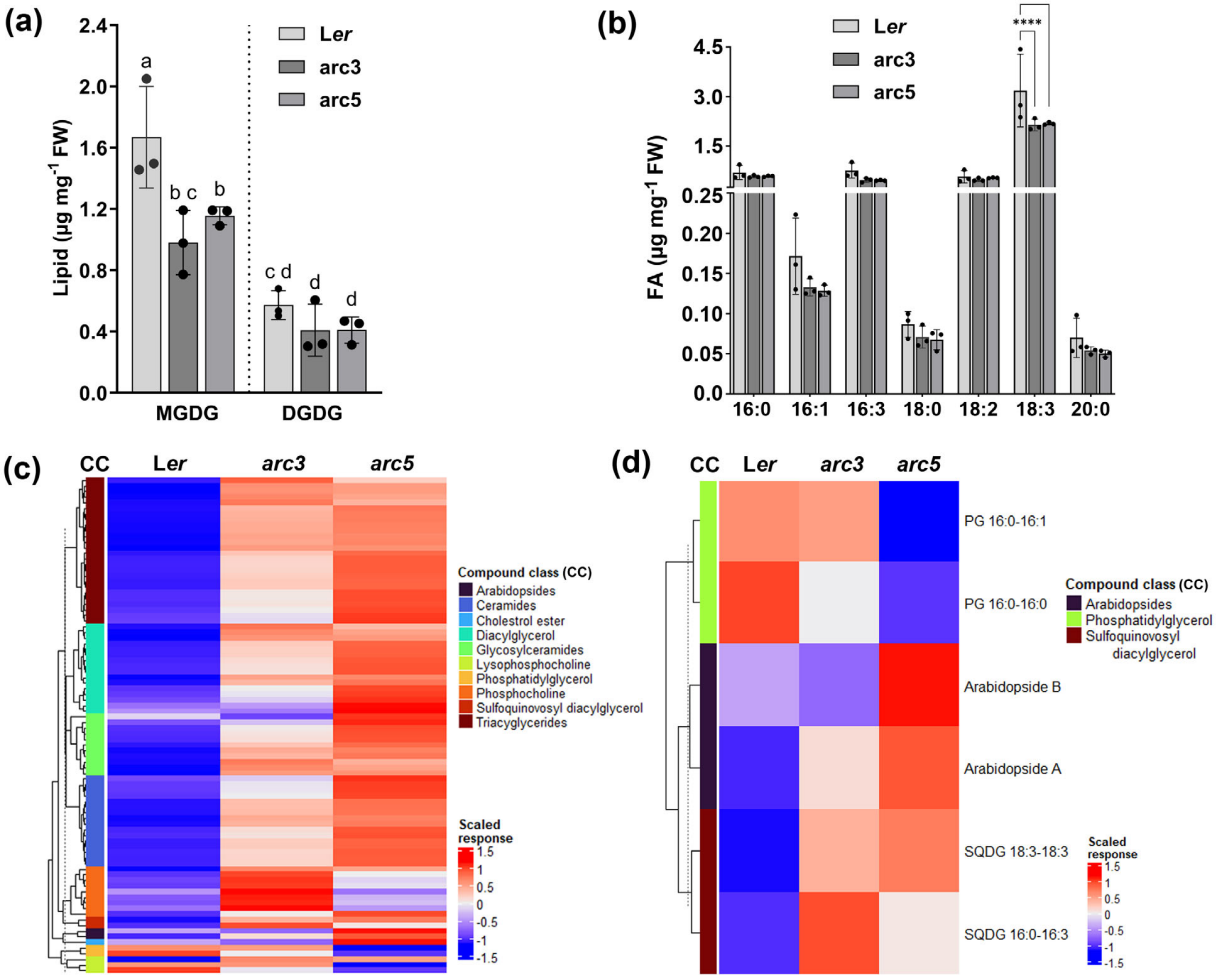
Lipidomic profiling reveals remodelling of chloroplast membrane lipids in *arc* mutants. (a) Quantification of total MGDG and DGDG content. Lipids were extracted from leaf tissue, separated by thin‐layer chromatography, and individual galactolipid bands were recovered and transesterified to fatty acid methyl esters for GC–MS quantification. Values represent total galactolipid content expressed as μg mg^−1^ fresh weight (FW). Data are presented as means ± SD (n = 3–4 biological replicates). Different letters denote statistically significant differences according to one‐way ANOVA followed by Tukey's multiple comparison test (*P* < 0.05). (b) Fatty acid composition of total lipid extracts determined by GC–MS analysis. Fatty acid species are designated by the number of carbons and double bonds and given as μg mg^−1^ FW. Data are presented as means ± SD (n = 3–4 biological replicates). Statistical comparisons were performed for each fatty acid species separately using one‐way ANOVA followed by Tukey's multiple comparison test. Asterisks indicate statistically significant differences between genotypes (***P < 0.0001). (c) Heatmap showing hierarchical clustering of lipid compound classes (CC) in wild‐type L*er* and *arc* mutants (n = 5 biological replicates per genotype). Each row represents an individual lipid species indicated by colour‐coded bars (right) and columns represent genotypes. Hierarchical clustering was performed using Euclidean distance and complete linkage methods using compounds showing significant differences between wild type and mutants. The heatmap displays row‐wise *z*‐score normalized abundances with red indicating elevated abundance and blue indicating reduced abundance as defined in ‘Scaled response’ at the right. (b) Heatmap showing individual molecular species within major chloroplast lipid classes including arabidopsides, phosphatidylglycerol (PG) and sulfoquinovosyldiacylglycerol (SQDG). Lipid molecular species are annotated with acyl chain compositions (carbon number:number of double bonds). Compound classes are indicated by colour‐coded bars in the right margin and are displayed as row‐wise *z*‐score normalized abundances as defined in ‘Scaled response’ at the right. Raw data for (c, d) are provided in Dataset S1.

To understand the biochemical basis of enhanced JA biosynthesis in *arc* mutants we quantified levels of MGDG and DGDG and their fatty acid composition in wild type and mutants. Analysing them by TLC and GC‐FID, total MGDG and DGDG content of both *arc* mutants was reduced by 30%–40% in comparison to wild‐type plants (Fig. 3a). The MGDG:DGDG ratio remained, however, unchanged (Fig. S1), indicating coordinated reduced levels of both galactolipid classes in the *arc* mutants rather than selective MGDG‐to‐DGDG conversion. Detailed fatty acid profiling showed that this reduction was driven primarily by reduction in the levels of esterified α‐linolenic acid (18:3), while other fatty acids (16:0, 16:1, 18:0, 20:0) remained unchanged (Fig. 3b). The reduced 18:3 levels were observed in both MGDG and DGDG pools, thereby changing the levels of the predominant fatty acid in chloroplast galactolipids and the major substrate for JA biosynthesis. To elucidate changes in other lipid species, untargeted lipidomics of chloroplast lipid molecular species was performed and revealed additional membrane remodelling in *arc* mutants (Fig. 3c, Data S1). Hierarchical clustering showed that *arc3* and *arc5* had distinct lipid profiles with a significant increase in some of the lipid classes, mainly triacylglycerides, Arabidopsides, phosphatidylglycerols and sulfoquinovosyldiacylglycerols (SQDG). A closer look at potential substrates of jasmonate biosynthesis showed significant changes in only few lipid species, which might serve as the primary reservoir of α‐linolenic acid for jasmonate biosynthesis (Schaller *et al*. 2004), such as Arabidopside A and B as well as SQDG 18:3–18:3 and SQDG 16:0–16:3 (Fig. 3d). In contrast, *kac1/2* and *chup1* mutant plants exhibited only small differences in lipid pools compared to Col‐0, concomitant with their unaltered chloroplast size and number (Fig. S2).

### Giant chloroplasts in mesophyll cells of *arc3* and *arc5* mutants show altered thylakoid organization and increased AOC levels

**Fig. 4:**
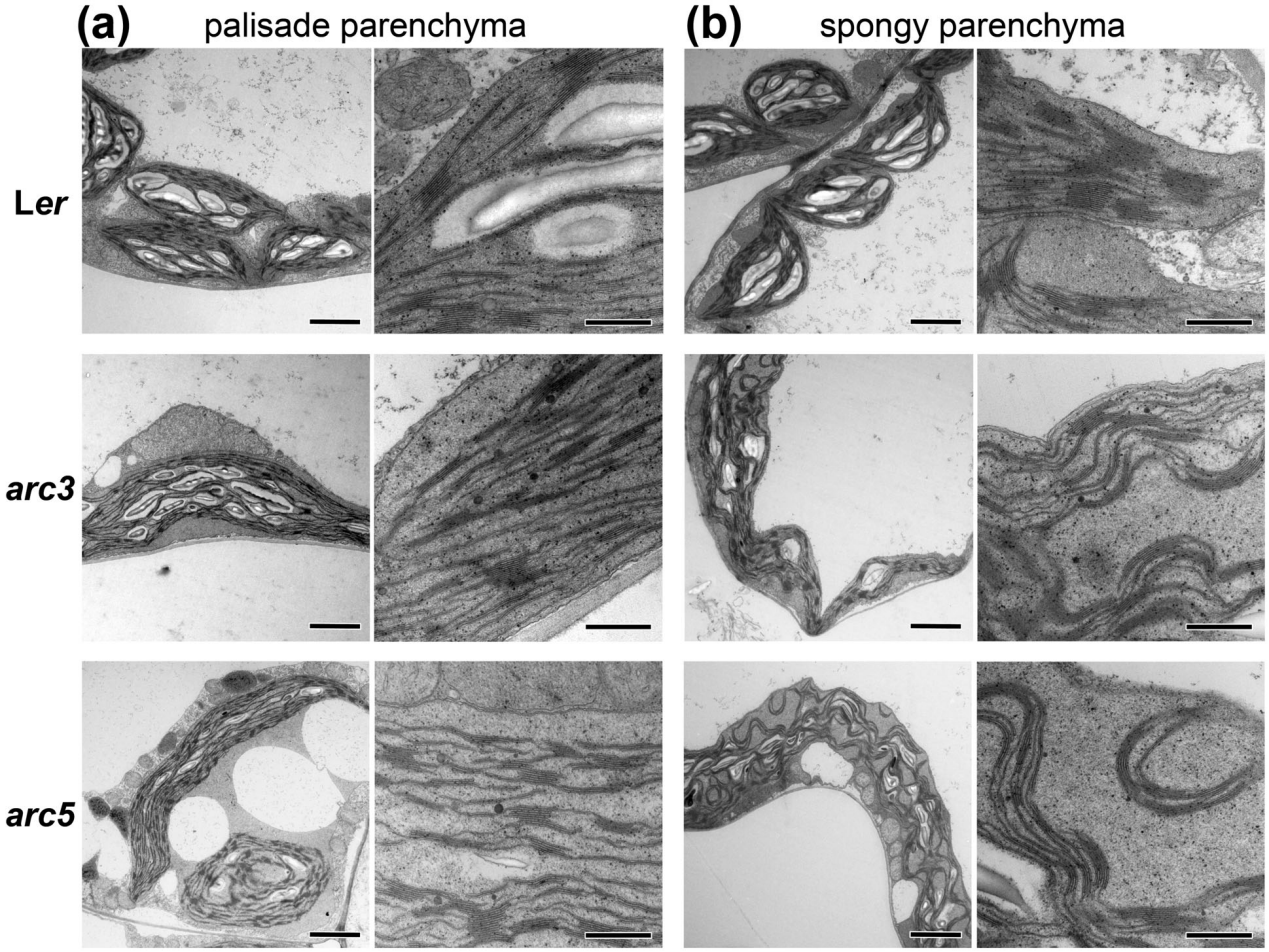
Mesophyll chloroplasts of *arc* mutants show altered thylakoid organization. Representative transmission electron micrographs showing chloroplast ultrastructure in palisade parenchyma (a) and spongy parenchyma (b) cells of wild‐type L*er*, *arc3* and *arc5* plants. Overview micrographs of chloroplasts (left) and details of thylakoid membranes (right) are presented. Note that wild‐type chloroplasts exhibit tightly stacked thylakoid membranes forming distinct grana, while *arc* mutant chloroplasts display loosely organized thylakoids with increased stromal spaces. Scale bars indicate 2.5 μm for overview pictures (left) and 0.5 μm for details (right).

The counterintuitive finding that *arc* mutants exhibit enhanced jasmonate production despite reduced galactolipid levels prompted us to examine whether structural alterations in the giant chloroplasts might contribute to their elevated metabolic activity. We employed transmission electron microscopy (TEM) to analyse whole leaf cross‐sections and individual chloroplasts in both palisade and spongy parenchyma cells. Analyses on tissue level did not show differences in tissue organization and cell size but revealed the pronounced size differences between wild type and *arc* mutant chloroplasts (Fig. S3). As already visualized by light microscopy (Fig. 1), mesophyll cells of wild‐type leaves contain numerous small, uniformly distributed chloroplasts, whereas mesophyll cells of *arc3* and *arc5* mutants showed substantially reduced chloroplast numbers with massively enlarged individual plastids that dominated the cellular space. On ultrastructural level, wild‐type L*er* chloroplasts showed the typical organization with well‐developed, tightly stacked thylakoid membranes forming distinct grana and interconnecting stroma lamellae (Fig. 4). With that, the chloroplasts displayed compact, organized internal membrane systems with dense thylakoid stacking characteristic of photosynthetically active plastids. In both palisade and spongy parenchyma tissues, wild‐type chloroplasts maintained consistent size and regular, oval morphology with uniform thylakoid distribution throughout the stroma. In contrast, the giant chloroplasts of *arc3* and *arc5* mutants displayed substantially altered internal organization. The enlarged plastids exhibited extensive regions with loosely organized thylakoid membranes, particularly evident in the peripheral areas of the chloroplasts. While some regions maintained organized grana stacks, large areas contained dispersed, less tightly packed thylakoids with increased spacing between membrane systems. Chloroplasts of both mutants showed heterogeneous internal organization with prominent stromal spaces, which appeared to be increased. Notably, the altered thylakoid architecture was consistent across both palisade and spongy parenchyma cells, indicating that the ultrastructural changes were intrinsic to the giant chloroplast phenotype rather than cell‐type specific.

**Fig. 5:**
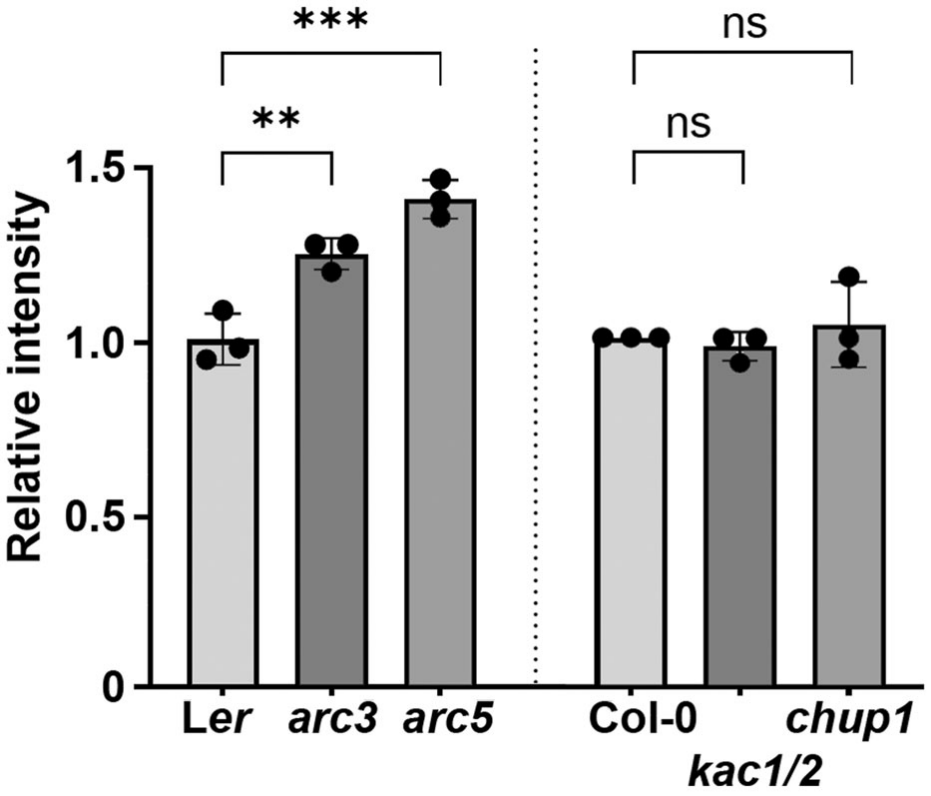
*arc* mutants exhibit increased AOC protein abundance. Relative AOC protein levels in chloroplast division mutants (*arc3*, *arc5*) and chloroplast positioning mutants (*chup1*, *kac1/2*) compared to their respective wild‐type controls, determined by Western blot analysis and normalized to β‐actin as loading control. Data are presented as means ± SE (n = 3). Asterisks indicate statistically significant differences compared to respective wild‐type controls (**P < 0.01, ***P < 0.001; one‐way ANOVA followed by Tukey's HSD test). ns, not significant. Underlying immune blots are shown in Fig. S4.

To determine whether the higher appearance of stromal area in the giant chloroplasts translated to increased abundance of JA biosynthesis enzymes, we quantified AOC protein levels using western blot analysis (Fig. 5 and Fig. S4). Both *arc3* and *arc5* mutants showed significantly increased AOC protein levels compared to wild type. In contrast, *kac1/2* and *chup1* mutants showed no substantial differences in AOC protein abundance relative to their wild‐type controls. These results indicate that chloroplast size and internal organization, rather than chloroplast positioning or number, are the critical determinants of jasmonate biosynthetic capacity following mechanical stress.

### *arc* mutants exhibit enhanced touch sensitivity and delayed flowering

**Fig. 6:**
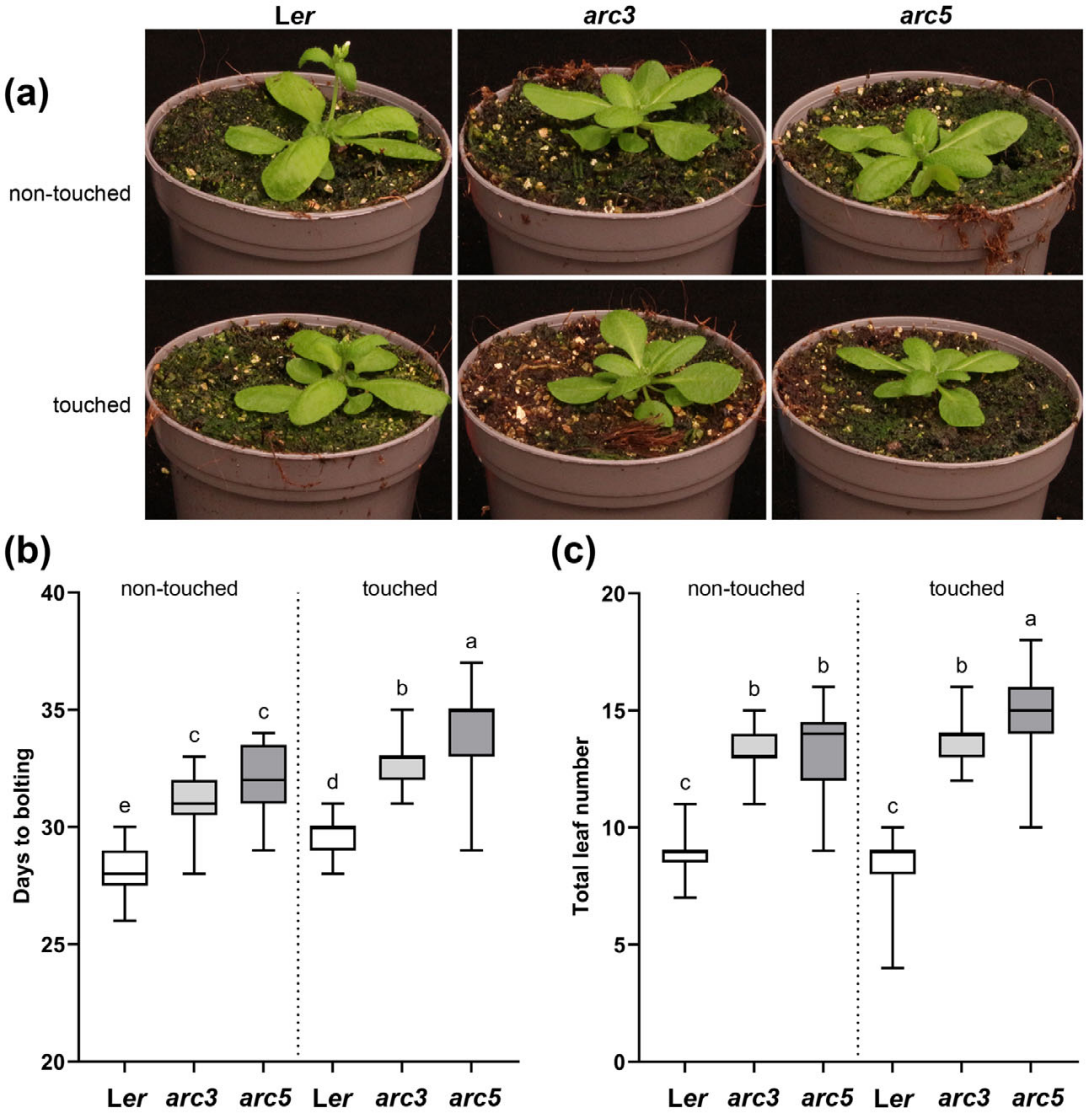
*arc* mutants exhibit delayed flowering and enhanced thigmomorphogenetic responses. (a) Representative images of L*er*, *arc3* and *arc5* plants at bolting time. Plants were grown under long‐day conditions and remained either non‐touched (upper panels) or were touched daily twice with a soft brush starting on day 14 after sowing. (b) Day of bolting of L*er*, *arc3* and *arc5* plants either non‐touched or touched. Both *arc* mutants exhibited enhanced sensitivity to mechanical stimulation as touch treatment delayed bolting by 4–5 days compared to their respective non‐touched controls, whereas L*er* plants showed less delay. (c) Number of rosette leaves of L*er*, *arc3* and *arc5* plants at bolting time either non‐touched or touched. Note that *arc5* plants developed significantly more leaves before bolting compared to wild type under both conditions indicating prolonged vegetative growth. Data are shown as box and whisker blots. Different letters indicate statistically significant differences (*P* < 0.05) according to one‐way ANOVA followed by Tukey's HSD test (n = 33 for non‐touched plants, n = 65 for touched plants).

Jasmonates are not only involved in the plant's wound response, but also mediate touch responses and thigmomorphogenesis (van Moerkercke *et al*. 2019; Darwish *et al*. 2022). This prompted us to assess the responses of *arc3*, *arc5* and L*er* plants to a reoccurring touch stimulus (Darwish *et al*. 2022) (Fig. 6). Under non‐touched conditions, wild‐type, *arc3* and *arc5* plants bolted at approximately 28, 31 and 32 days, respectively (Fig. 6a,b). However, mechanical stimulation revealed substantial differences in touch sensitivity between genotypes, with *arc* mutants exhibiting enhanced sensitivity compared to wild‐type plants. While wild‐type plants showed only a modest delay in bolting (approximately 2 days) in response to touching, touch treatment delayed bolting by approximately 4–5 days in *arc3* and *arc5* mutants compared to their respective non‐touched controls (Fig. 6b). Furthermore, total leaf number analysis revealed that touched *arc5* plants produced significantly more leaves before bolting compared to other genotypes, indicating a more pronounced vegetative growth phase under mechanical stress (Fig. 6c). Interestingly, despite the pronounced effects on developmental timing, rosette area remained unchanged across all genotypes under mechanical stimulation, suggesting that the enhanced touch sensitivity in *arc* mutants specifically affects reproductive transition rather than overall vegetative growth (Fig. S5). These results showed that the enhanced jasmonate biosynthetic capacity observed in *arc* mutants confers increased sensitivity to touch.

## DISCUSSION

Our findings show a novel association between chloroplast morphology and jasmonate biosynthetic capacity, demonstrating that organellar architecture is directly linked to hormone metabolism and environmental responsiveness. The enhanced jasmonate production in *arc* mutants upon wounding is particularly intriguing given the substantially reduced chloroplast numbers and, counterintuitively, significantly reduced galactolipid substrate levels. Repeatedly performed touch treatment resulted in an enhanced thigmomorphogenetic response in these mutants, demonstrating a typical phenotype of jasmonate‐overproducing plants. With this, the work establishes chloroplast morphology as a previously unrecognized determinant of jasmonate homeostasis driving the growth‐defence trade‐off.

Arabidopsis *arc3* and *arc5* mutants possess defects in chloroplast division machinery, leading to cells containing only a few but greatly enlarged chloroplasts, compared with much smaller chloroplasts in wild‐type plants (Pyke & Leech 1994; Fig. 1). These mutants provide an excellent model for examining how plastid size influences their metabolic activity. It has been shown that cells with fewer and larger chloroplasts exhibit a lower mesophyll conductance and limited photosynthetic competence than cells with more and smaller chloroplasts (Austin II & Webber 2005; Weise *et al*. 2015). Moreover, chloroplasts from *arc* mutants differ in the abundance of proteins when compared to wild type, whereas the profile of the native stromal and membrane complexes remains unchanged (Gargano *et al*. 2013). Here, we show another metabolic effect of enlarged chloroplasts: Leaves of *arc* mutants respond to wounding with an increased JA production, which is accompanied by enhanced transcript accumulation of JA‐regulated genes, such as *AtLOX2*, *AtAOS* and *AtJAZ10* (Fig. 2). The relationship between transcript levels of JA biosynthetic genes and JA accumulation is, however, not linear. Although *arc3* and *arc5* accumulate similar levels of JA/J‐Ile 1 h after wounding, their gene expression responses differ. *arc3* shows strongly elevated transcripts for all examined genes, whereas *arc5* shows only a non‐significant increase. This discrepancy likely reflects differences in their molecular division defects, as well as potentially different kinetics of transcript induction and JA‐independent wound‐signalling pathways. Regardless, the primary functional readout of JA/JA‐Ile levels is consistently elevated in both *arc* mutants compared to wild type. The specificity of this effect is underscored by data obtained from the positioning mutants *chup1* and *kac1/2*, which contain a similar number of small chloroplasts and retain jasmonate levels like wild‐type plants despite altered chloroplast distribution. The size of these chloroplasts was, however, approximately twice as large as previously described for wild‐type Col‐0 (Knoblauch *et al*. 2024). This discrepancy may be explained by differences in growth conditions, as all genotypes in the present study were cultivated under short‐day conditions and lower light intensities, whereas Knoblauch *et al*. (2024) used long‐day conditions and higher light intensities. Given that light is a key factor regulating chloroplast development and morphology, including chloroplast size (Cackett *et al*. 2022), these differences in growth conditions likely contributed to the observed variation. However, the accumulation of *cis*‐OPDA without corresponding JA/JA‐Ile increases in the chloroplast‐positioning mutants suggests possible defects in *cis*‐OPDA export or downstream conversion within the peroxisomes, warranting further study of chloroplast–peroxisome communication.

The lipidomics data showed a reduction of the MGDG and DGDG levels in *arc* mutants in comparison to wild type, while their ratio was not changed. This contrasts to stress responses, where MGDG is specifically converted to oligogalactolipids or even degraded, for example, upon freezing or nitrogen starvation, respectively (Moellering *et al*. 2010; Li *et al*. 2012). The unaltered MGDG:DGDG ratio might contribute to unaltered membrane integrity, since their ratio maintains the critical balance between bilayer and non‐bilayer phases in thylakoids (Demé *et al*. 2014; Kobayashi 2016), and its disruption causes severe photosynthetic impairment as shown for *mgd1* or *dgd1* mutants (Dörmann *et al*. 1995). By maintaining the MGDG:DGDG ratio through coordinated degradation, membrane architecture might be preserved in *arc* mutants, while fatty acids are channelled towards defence metabolism. The selective reduction in esterified 18:3 in *arc* mutants may reflect constitutively elevated basal LOX activity, potentially facilitated by loosely organized thylakoids increasing substrate accessibility. The elevated Arabidopside A and B levels in *arc* mutants (Fig. 3d) provide direct evidence for enhanced in situ oxygenation of galactolipid esterified 18:3 under basal conditions. The connection between Arabidopside accumulation and reduced esterified 18:3 represents a plausible mechanistic link between chloroplast architecture and altered galactolipid composition, although further experimental validation is required. Interestingly, *kac1/2* and *chup1* mutants show almost no lipidomic alterations and no JA elevation revealing that enlarged chloroplasts may create a unique metabolic state for channelling membrane components into defence signalling. Although substrate limitation is one hypothetical constraint for jasmonate biosynthesis (Wasternack & Hause 2013), the reduced MGDG and DGDG contents including the reduced levels in 18:3 fatty acid in *arc* mutants might still be sufficient to enable the enhanced jasmonate production under wounding stress. Classical models predict reduced biosynthetic efficiency in enlarged organelles due to unfavourable surface area‐to‐volume ratios and diffusion limitations (Rafelski 2013; Marshall 2016). However, our findings show that giant chloroplasts maintain – or even enhance – specific metabolic functions despite substantial size increases, implying the presence of structural and kinetic compensation mechanisms that defy classical scaling expectations. It is tempting to speculate that enhanced JA production in *arc* mutants might occur through active substrate mobilization and/or higher substrate availability.

Structural alterations within the giant chloroplasts appear to be a central factor leading to enhanced jasmonate production. Such architecture‐driven optimization was exemplified for mitochondrial fusion in steroidogenic cells promoting enzyme channelling (Rone *et al*. 2009; Duarte *et al*. 2012). Our ultrastructural analyses revealed that *arc* mutants harbour chloroplasts with loosely organized thylakoids and expanded membrane spacing, unlike the compact stacking in wild‐type chloroplasts (Fig. 4). This organization might generate membrane microdomains that enhance enzyme accessibility and metabolic flux. The more open thylakoid networks in giant chloroplasts of *arc* mutants may increase enzyme accessibility by reducing diffusion barriers, favouring processes such as jasmonate biosynthesis, which is spatially separated with localization of LOX on thylakoids and AOS/AOC on the inner envelope and stroma (Froehlich *et al*. 2001; Farmaki *et al*. 2007; Stenzel *et al*. 2012), but depends on substrate channelling (Schaller & Stintzi 2009). Moreover, *arc* mutants contain increased levels of AOC (Fig. 5), thereby facilitating closer enzyme interactions and possibly leading to improved metabolic channelling needed for the synthesis of *cis*‐OPDA (Schaller & Stintzi 2009). Although none of the enzymes involved in JA biosynthesis has been identified as rate‐limiting, increased abundance of a single enzyme can be sufficient to enhance JA production during stress, as demonstrated by ectopic overexpression of either AOS (Laudert *et al*. 2000) or AOC (Stenzel *et al*. 2003a). In *arc* mutants, elevated *AOC* transcript and protein levels co‐occur with significantly increased OPDA and JA accumulation after wounding, suggesting active mobilization of the available substrate towards JA biosynthesis. These findings collectively support the conclusion that elevated AOC abundance might drive enhanced jasmonate accumulation in the altered chloroplast structural context of *arc* mutants.

Thigmomorphogenetic responses of *arc* mutants were enhanced in comparison to wild‐type plants. Upon repeated brushing, *arc* mutants exhibited a 4–5‐day bolting delay compared with the modest 2‐day delay in wild type (Fig. 6). Jasmonates have been shown to mediate touch responses, as JA levels increase in touched plants (Tretner *et al*. 2008; Chehab *et al*. 2012) and JA‐deficient or insensitive mutants (*aos*, *coi1*, *jar1*) completely lack touch responses (Yan *et al*. 2007; Chehab *et al*. 2012). The increased touch sensitivity of *arc* mutants is indicative of an enhanced JA biosynthesis as demonstrated for plants overexpressing *OPR3* showing constitutive JA overproduction accompanied by enhanced touch responses (Chehab *et al*. 2012). Repeatedly performed touching stimulates JA biosynthesis resulting in a positive feedback loop further enhancing jasmonate levels and is accompanied by reduced growth and delayed flowering as the defence‐growth balance is altered (van Moerkercke *et al*. 2019). Touch signalling operates, however, through two complementary branches: a JA‐dependent pathway mediated by MYC2/3/4 transcription factors and a CAMTA3‐dependent and JA‐independent pathway (van Moerkercke *et al*. 2019). Moreover, thigmomorphogenesis involves hormonal crosstalk, notably JA‐mediated gibberellin catabolism via upregulation of *GIBBERELLIN 2‐OXIDASE7* encoding a protein that inactivates bioactive gibberellins (Wang *et al*. 2023; Fernandez‐Moreno *et al*. 2024). Here, a reduced level of bioactive GA and an increased level of JA is required for the touch‐mediated growth alteration (Chehab *et al*. 2012; Lange & Lange 2015). Enhanced jasmonate levels in *arc* mutants may preferentially affect the bolting time without major effects on vegetative growth (Fig. S4) suggesting a more pronounced effect on reproductive developmental program than on general growth inhibition. The selective effect on bolting rather than vegetative growth is consistent with the distinct hormonal sensitivities of these two developmental processes, whereby floral transition is critically dependent on GA signalling while rosette growth involves multiple redundant hormonal crosstalks. In our experimental setup, mechanical stimulation was applied from 14 days after sowing, representing a mild touch regime under which even wild‐type plants showed no significant reduction in rosette leaf area (Fig. S4), indicating that the stimulus intensity was insufficient to trigger strong growth‐inhibitory responses. Together, these observations suggest that the enhanced JA biosynthetic capacity of *arc* mutants amplifies touch‐induced JA accumulation sufficiently to suppress the GA‐dependent floral transition, leaving vegetative growth largely unaffected under mild stimulation conditions. This is consistent with ecological models proposing thigmomorphogenesis as an adaptive mechanism for optimizing reproductive timing under mechanical stress (Coutand 2010; Moulia *et al*. 2015).

Natural variation in chloroplast size and morphology exists across plant species and even within populations, suggesting that morphology‐dependent metabolic regulation could potentially serve as a mechanism for fine‐tuning stress responses to local environmental conditions. Notably, allelic variations in the chloroplast division gene *FtsZ2‐2* drive natural variation in chloroplast size across Arabidopsis ecotypes, suggesting that such morphological differences may have broader adaptive significance (Kadirjan‐Kalbach *et al*. 2019). Our findings suggest that this natural variation in organelle architecture may have previously unappreciated consequences for jasmonate biosynthetic capacity, and thereby for stress hormone homeostasis and environmental responsiveness. While the exaggerated chloroplast phenotypes of *arc* mutants are unlikely to occur in wild‐type tissues under normal physiological conditions, the present study establishes the proof of principle that plastid architecture is associated with jasmonate biosynthesis output. Future studies using natural ecotype variation or inducible perturbations may assess the quantitative contribution of this association under more physiological conditions. Nevertheless, the effect of enlarged chloroplasts on the capacity of JA biosynthesis in rosette leaves of Arabidopsis demonstrated here represents another example of plants' flexibility in their adaptation to stress conditions. The ability to enhance jasmonate production through chloroplast architectural changes could provide resilience against multiple simultaneous stresses and may represent an evolutionary mechanism for environmental adaptation that operates independently of changes in biosynthetic gene sequences or expression levels.

## AUTHOR CONTRIBUTIONS

RB and HS designed experiments with input from JZ and BH. RB, HS, MH, SK and SNK performed experiments. RB, MH, SK and SNK analysed the data and performed statistical analyses. RB and BH wrote the manuscript with suggestions and approval of all authors.

## CONFLICT OF INTEREST STATEMENT

No conflict of interest declared.

## Supporting information


**Fig. S1.** Ratio of MGDG and DGDG in rosette leaves from L*er*, *arc3* and *arc5*.
**Fig. S2.** Hierarchical clustering of lipid profiles from *kac1/2*, *chup1* and their wild‐type Col‐0.
**Fig. S3.** Leaf morphology of L*er* and *arc*‐mutants.
**Fig. S4.** Western blot analysis of AOC protein levels in chloroplast mutants.
**Fig. S5.** Touch treatment does not alter rosette area.

**Data S1.** Non‐targeted lipid analysis.

## Data Availability

Seeds and data underlying the figures are available upon request from Ranjit Baral (ranjit.baral@ipb-halle.de) or Bettina Hause (bhause@ipb-halle.de).
