# Supplementary material for: Size of chloroplasts in Arabidopsis mesophyll cells affects jasmonate biosynthesis
Source: Plant Biol (Stuttg). 2026 May 11;28(5):1330–42. doi: 10.1111/plb.70222 (PMC13358708; doi:10.1111/plb.70222)
Supplement: Supplementary file 1 — Fig. S1. Ratio of MGDG and DGDG in rosette leaves from Ler, arc3 and arc5. Fig. S2. Hierarchical clustering of lipid profiles from kac1/2, chup1 and their wild‐type Col‐0. Fig. S3. Leaf morphology of Ler and arc‐mutants. Fig. S4. Western blot analysis of AOC protein levels in chloroplast mutants. Fig. S5. Touch treatment does not alter rosette area. [file PLB-28-1330-s001.zip › plb70222-sup-0001-Supinfo1@150c10d5-0bc6-4166-b872-11f17d14e24b.pdf]

Original Article

Size of chloroplasts in Arabidopsis mesophyll cells affects jasmonate biosynthesis

Submission ID 150c10d5-0bc6-4166-b872-11f17d14e24b

Submission Version Initial Submission

PDF Generation 19 Jan 2026 10:46:00 EST by Atypon ReX

Authors

Mr. Ranjit Baral

Affiliations
- Department of Cell and Metabolic Biology, Leibniz Institute of Plant Biochemistry, Weinberg 3, D-06120 Halle/Saale, Germany

Mr. Hagen Stellmach

Affiliations
- Department of Cell and Metabolic Biology, Leibniz Institute of Plant Biochemistry, Weinberg 3, D-06120 Halle/Saale, Germany

Dr. Samuel Ngure Kariithi

Affiliations
- Program Center MetaCom, Leibniz Institute of Plant Biochemistry, Weinberg 3, D-06120 Halle/Saale, Germany

Dr. Mareike Heilmann

Affiliations
- Martin-Luther-University Halle-Wittenberg, Institute of Biochemistry and Biotechnology, Charles Tanford Protein Center, Kurt-Mothes-Str. 3a, D-06120 Halle/Saale, Germany

Dr. Stephanie Krüger

ORCiD
https://orcid.org/0000-0002-7159-8316

Affiliations
- Martin Luther University Halle Wittenberg, Biozentrum, Core Facility Microscopy, Weinbergweg 22, D-06120 Halle/Saale, Germany

Dr. Joerg Ziegler

Affiliations

- Program Center MetaCom, Leibniz Institute of Plant Biochemistry, Weinberg 3, D-06120 Halle/Saale, Germany

Prof. Bettina Hause
Corresponding Author
Submitting Author

ORCiD
https://orcid.org/0000-0001-9697-4990

Affiliations

- Department of Cell and Metabolic Biology, Leibniz Institute of Plant Biochemistry, Weinberg 3, D-06120 Halle/Saale, Germany

Files for peer review

All files submitted by the author for peer review are listed below. Files that could not be converted to PDF are indicated; reviewers are able to access them online.

Name	Type of File	Size	Page
Baral etal_submission.docx	Main Document - MS Word	136.7 KB	Page 4
Figure 1.tif	Figure	6.4 MB	Page 39
Figure 2.tif	Figure	8.3 MB	Page 40
Figure 3.tif	Figure	3.7 MB	Page 41
Figure 4.tif	Figure	7.7 MB	Page 42
Figure 5.tif	Figure	388.9 KB	Page 43
Figure 6.tif	Figure	2.8 MB	Page 44
Baral etal_Supplementary figures.pdf	Supplementary Material for Review	722.0 KB	Page 45
Baral etal_Supplementary_data set 1.xlsx	Supplementary Material for Review	517.0 KB	Page 51

Size of chloroplasts in Arabidopsis mesophyll cells affects jasmonate biosynthesis

Ranjit Baral[1], Hagen Stellmach[1], Samuel Ngure Kariithi[2], Mareike Heilmann[3], Stephanie Krüger[4], Joerg Ziegler[2], Bettina Hause[1]

[1]Department of Cell and Metabolic Biology, Leibniz Institute of Plant Biochemistry, Weinberg 3, D-06120 Halle/Saale, Germany

[2]Program Center MetaCom, Leibniz Institute of Plant Biochemistry, Weinberg 3, D-06120 Halle/Saale, Germany

[3]Martin-Luther-University Halle-Wittenberg, Institute of Biochemistry and Biotechnology, Charles Tanford Protein Center, Kurt-Mothes-Str. 3a, D-06120 Halle/Saale, Germany

[4]Martin Luther University Halle Wittenberg, Biozentrum, Core Facility Microscopy, Weinbergweg 22, D-06120 Halle/Saale, Germany

Summary

Chloroplasts are highly dynamic organelles whose morphology responds to developmental and environmental cues, yet whether organellar architecture directly influences metabolic capacity remains unclear. Using Arabidopsis *arc3* and *arc5* mutants harboring defective division machinery, we investigated how chloroplast morphology affects biosynthesis of jasmonates, such as jasmonic acid (JA) and jasmonoyl-isoleucine (JA-Ile). Quantitative three-dimensional analysis using confocal laser scanning microscopy revealed enlarged chloroplasts with correspondingly reduced numbers per cell in *arc* mutants. Upon wounding, *arc* mutants accumulated higher levels of JA and JA-Ile than wild type, whereas chloroplast positioning mutants, such as *chup1* and *kac1/2*, harbor chloroplasts of similar size and number and produced similar levels of jasmonates as wild-type plants. Determination of galactolipids revealed reduced levels in *arc* mutants, particularly in the jasmonate precursor α-linolenic acid. Ultrastructural analysis demonstrated that giant chloroplasts in *arc* mutants possess loosely organized thylakoid membranes with expanded stromal regions. Moreover, levels of the JA biosynthetic enzyme allene oxide cyclase were enhanced in *arc* mutants. The combination of altered membrane architecture and increased enzyme abundance likely underlies the enhanced jasmonate production, resulting in a stronger mechanostimulation response of these mutants: Compared to wild type, *arc* mutants exhibited more pronounced flowering delays under repeated mechanical stimulation. Collectively, these findings identify chloroplast morphology as a previously unrecognized determinant of jasmonate biosynthesis and establish a direct link between organellar architecture and hormone biosynthesis during plant stress adaptation.

Keywords

Chloroplast morphology, jasmonate biosynthesis, organellar architecture, *arc* mutants, thigmomorphogenesis, galactolipids, allene oxide cyclase, touch sensitivity, flowering time

Significance Statement

The relationship between chloroplast structure and hormone biosynthesis capacity is poorly understood. This study shows that mutants affected in plastid division and therefore harboring giant chloroplasts with altered ultrastructure produce more jasmonic acid upon mechanical stress than wild-type plants, establishing organellar architecture as a previously unrecognized regulatory mechanism in plant hormone biosynthesis and stress adaptation.

Introduction

Chloroplasts are highly dynamic organelles whose division, movement, and morphological changes respond to developmental and environmental cues (Jarvis and López-Juez 2013, Osteryoung and Pyke 2014). Arabidopsis mutants defective in plastid division machinery, such as ACCUMULATION AND REPLICATION OF CHLOROPLASTS3 (ARC3) and ARC5, exhibit highly enlarged chloroplasts with markedly lower numbers per cell (Pyke and Leech 1994, Robertson *et al.* 1995). The ARC3 protein as a stromal Z-ring accessory protein functions in conjunction with ARC6 to bind FtsZ proteins and to promote the dynamics of chloroplast Z rings (Du *et al.* 2025, Maple *et al.* 2007, Zhang *et al.* 2013). In contrast, ARC5 encodes a cytosolic dynamin-related protein that functions late in chloroplast division by mediating the final constriction and separation of daughter chloroplasts (Gao *et al.* 2003, Miyagishima *et al.* 2006). ARC5 assembles into discontinuous rings on the cytosolic surface of the outer envelope membrane and is recruited to the division site through interactions with PLASTID DIVISION1 (PDV1) and PDV2 in a coincidence-driven, ER-mediated manner (Ghosh *et al.* 2025, Miyagishima *et al.* 2006).

Chloroplasts of the *arc3* and *arc5* mutants exhibit distinct but complementary division defects: *arc3* chloroplasts show multiple aberrant Z-rings and irregular shapes due to defective division site specification, while *arc5* chloroplasts display a characteristic dumbbell-shape with initiated constriction but failing complete separation (Gao *et al.* 2003, Zhang *et al.* 2013)**.** Despite detailed analysis of division defects, the metabolic effects of the altered chloroplast architecture in *arc3* and *arc5* mutant plants have been analyzed mainly with respect to photosynthesis as *arc* mutants are limited in their photosynthetic competence and display photosynthetic characteristics of low light acclimated plants (Austin II and Webber 2005). Other chloroplast-localized metabolic pathways remain rather understudied in the *arc* mutants, among them the biosynthesis of defense hormones, such as abscisic acid, salicylic acid and jasmonic acid (JA).

JA and its derivatives, commonly named jasmonates, mediate one of the most important plant defense pathways, i.e., responses to mechanical stress, herbivory, and pathogen attack, and are also involved in developmental processes (Wasternack and Hause 2013). The biosynthesis of JA begins in chloroplasts where a 13-LIPOXYGENASE (LOX), a 13-ALLENE OXIDE SYNTHASE (AOS), and an ALLENE OXIDE CYCLASE (AOC) convert galactolipid-derived fatty acids into 12-*cis*-oxo-phytodienoic acid (*cis*-OPDA) (Wasternack and Song 2017). Among the four 13-LOXs of *A. thaliana*, LOX2 is active in wound-induced JA formation in aerial parts of the plant (Chauvin *et al.* 2016). In peroxisomes, *cis*-OPDA is then reduced by OPDA REDUCTASE 3 (OPR3) followed by three rounds of β-oxidation yielding JA (Stintzi and Browse 2000). Recent evidence indicates that cytosolic OPR2 also contributes to JA synthesis through an alternative pathway involving 4,5-didehydro-JA (Chini *et al.* 2018, Wasternack and Hause 2018). The final reaction leading to the bioactive form of jasmonates, (+)-7-*iso*-jasmonoyl-isoleucine (JA-Ile), is performed by JASMONATE RESISTANT1 (JAR1), a cytosolic enzyme that conjugates JA to isoleucine (Staswick *et al.* 2002). JA-Ile, the active ligand in most plants, directly interacts with CORONATINE INSENSITIVE1 (COI1), an integral component of the SKP1-CUL1-F-box protein E3 ubiquitin ligase complex, thereby triggering the interaction between COI1 and JASMONATE ZIM-domain proteins (JAZs) and leading to the degradation of JAZs through the 26S proteasome (Howe *et al.* 2018,Wasternack, 2013 #8536). JAZs are the main repressors that inhibit JA signal transduction by binding to transcription factors like MYC2 (Zhang *et al.* 2015). It has been reported that functional jasmonate signalling mediates touch-induced morphogenesis, since jasmonate deficient and insensitive mutants (e.g., *aos*, *jar1*, and *coi1*) are nonresponsive to repetitive mechanical stimulation, which results in delayed flowering of wild-type Arabidopsis (Chehab *et al.* 2012).

Despite the central role of chloroplasts in jasmonate biosynthesis, the relationship between chloroplast morphology and hormone production capacity has not been well investigated.

Chloroplast galactolipids, particularly monogalactosyldiacylglycerol (MGDG) and digalactosyldiacylglycerol (DGDG), serve as major substrate sources for JA biosynthesis, with α-linolenic acid (18:3) as the key precursor (Wang *et al.* 2018). The prevailing paradigm focuses primarily on substrate availability and enzyme abundance as the key drivers of metabolic output, leaving organellar architecture overshadowed in importance (Sweetlove and Fernie 2013). This substrate-centric view is supported by studies that show galactolipid availability directly influencing jasmonate biosynthetic capacity (Ibrahim *et al.* 2011, Lin *et al.* 2016, Yu *et al.* 2020). However, emerging evidence from other metabolic systems suggests that membrane organization can significantly influence biosynthetic efficiency independent of substrate levels, as demonstrated in animal steroid hormone production where mitochondrial fusion enhances biosynthesis through optimized enzyme localization (Duarte *et al.* 2012). Similarly, chloroplast membrane dynamics affect photosynthetic efficiency and metabolite transport (Johnson and Wientjes 2020, Kirchhoff 2019, Schwenkert *et al.* 2022). The spatial organization of jasmonate biosynthetic enzymes across chloroplast membrane systems suggests that membrane architecture may influence biosynthetic efficiency, with LOX2 localizing to both envelope and thylakoid membranes, AOS to the inner envelope, and AOC as soluble enzyme within the stroma but in close proximity to AOS (Farmaki *et al.* 2007, Froehlich *et al.* 2001, Stenzel *et al.* 2012). Since *arc* mutants exhibit aberrantly altered chloroplast architecture with distinct ultrastructural features (Pyke and Leech 1994), we hypothesized that structural reorganization in giant chloroplasts might influence jasmonate biosynthetic capacity through mechanisms beyond substrate availability.

Here, we employed quantitative 3D morphological analysis to characterize chloroplast architecture in *arc3* and *arc5* mutants in comparison to wild type and chloroplast positioning mutants, such as *kinesin-like protein for actin-based chloroplast movement1/2* (*kac1/2*) and *chloroplast unusual positiong1* (*chup1*) exhibiting altered chloroplast localization without major

size changes (Oikawa et al., 2003, 2008; Suetsugu et al., 2010). We investigated the relationship between organellar morphology and jasmonate metabolism through hormone quantification using LC-MS/MS, galactolipid substrate analysis by GC-MS, quantitative gene expression analysis, and ultrastructural examination by transmission electron microscopy. Our findings reveal an unexpected relationship between chloroplast size and jasmonate biosynthetic capacity, demonstrating that enlarged chloroplasts enhance hormone production despite reduced galactolipid substrate availability. The enhanced jasmonate biosynthesis translates into amplified thigmomorphogenetic responses, establishing chloroplast morphology as a previously unrecognized regulatory mechanism in plant environmental adaptation to mechanical stress.

Results

Quantitative 3D analysis reveals distinct chloroplast morphology in division and positioning mutants

To characterize the three-dimensional (3D) architecture of chloroplasts in rosette leaves of Arabdiopsis, we employed confocal laser scanning microscopy (LSM) coupled with 3D image reconstruction to quantify chloroplast morphological parameters in mesophyll cells of *arc3*, *arc5*, *kac1/2*, and *chup1* mutants as well as their corresponding wild type. Although qualitative chloroplast morphology changes in *arc* mutants have been previously reported (Holzinger *et al.* 2008, Pyke and Leech 1994), quantitative 3D parameters including surface area, volume, and sphericity have not been comprehensively characterized. Chloroplasts from *arc3* and *arc5* mutants were analyzed in comparison to *A. thaliana* cv. Landsberg *erecta* (L*er*), while those from *kac1/2* and *chup1* mutants were examined in comparison to *A. thaliana* cv. Columbia-0 (Col-0). Consistent with their defective chloroplast division phenotype, mesophyll cells in *arc3* and *arc5* mutants exhibited greatly reduced chloroplast number accompanied by proportionally increased

individual chloroplast size compared to wild type L*er* (Figure 1a). Quantitative analysis revealed that chloroplast number per mesophyll cell in *arc3* and *arc5* mutants in comparison to wild type appeared to be approximately 20-fold reduced from about 80 to 4 chloroplasts per cell (Figure 1b). Concomitant with these data, 3D morphometric analysis showed that the enlarged chloroplasts in both *arc* mutants had significantly increased surface area and volume compared to wild-type chloroplasts (Figure 1c, d). Interestingly, despite increase in individual chloroplast size, the total chloroplast volume per mesophyll cell appeared to be drastically reduced in *arc* mutants (around 4,500 μm^3) as compared to the wild type (around 23,000 μm^3). To determine chloroplast shape regularity, we employed sphericity analysis, where a value of 1.0 indicates a perfect sphere. Chloroplasts from *arc3* and *arc5* mutants exhibited reduced sphericity values indicating more irregular chloroplast morphology compared to wild-type chloroplasts, which showed a more regular, globular architecture (Figure 1e). In contrast to the division mutants, the chloroplast positioning and movement mutants showed distinct morphological patterns. Consistent with previous findings (Oikawa *et al.* 2008), mesophyll chloroplasts from the *chup1* mutant exhibited aberrant subcellular localization, clustering predominantly at the upper and lower side of a cell rather than displaying the uniform distribution within the cytosol observed in wild-type cells (Figure 1a). Morphometric analysis revealed that chloroplast number per mesophyll cell remained unchanged in both *kac1/2* and *chup1* mutants compared to wild type (Figure 1b), whereas chloroplast surface area was significantly reduced in both mutants (Figure 1c). Notably, chloroplast volume in *kac1/2* and *chup1* mesophyll cells was similar to wild-type chloroplasts (Figure 1d), indicating that the decreased surface area was compensated by increased sphericity, resulting in more compact, spherical chloroplasts (Figure 1e). These findings suggest that while KAC1/2 and CHUP1 do not affect chloroplast volume, they play critical roles in determining chloroplast shape and surface topology.

Wounding induces elevated jasmonate production in genotypes harboring enlarged chloroplasts

To investigate the impact of chloroplast morphology on jasmonate biosynthesis, we quantified *cis*-OPDA, JA, and JA-Ile in the 8th rosette leaf of 4-week-old plants at basal levels and at one hour after wounding, since this point is commonly used to evaluate wound-induced accumulation of JA (Mekkaoui *et al.* 2025). Under basal conditions, JA and JA-Ile levels were at the limit of quantification in all genotypes tested. In contrast, *cis*-OPDA was readily detectable and showed similar levels across all genotypes (Figure 2a, b).

Mechanical wounding induced substantial increases in all three compounds across genotypes but revealed significant differences between chloroplast morphology mutants. In comparison to wild-type leaves, leaves of the *arc3* and *arc5* mutants showed significantly elevated *cis*-OPDA, JA, and JA-Ile levels at one hour after wounding, with JA and JA-Ile levels being about 1.5-fold and 2.2-fold higher, respectively (Figure 2a). In contrast, the chloroplast positioning and movement mutants *kac1/2* and *chup1* showed no significant differences in JA and JA-Ile levels compared to wild type before and at one hour after wounding (Figure 2b). Wounding resulted, however, in an enhanced *cis*-OPDA accumulation in *chup1* and *kac1/2* leaves compared to wild type (Figure 2b).

To further elucidate the effects of enhanced jasmonate production in *arc* mutants, we analyzed transcript levels of genes being regulated by JA/JA-Ile, among them genes encoding JA biosynthesis enzymes, such as *AtLOX2* and *AtAOS* (Mekkaoui *et al.* 2025), and proteins involved in JA-signaling, such as *AtJAZ10* (Acosta *et al.* 2013). Determination of transcript accumulation using RT-qPCR demonstrated that wounding of *arc3* leaves resulted in significantly elevated transcript levels of *AtLOX2*, *AtAOS*, and *AtJAZ10* in comparison to wounded wild-type leaves (Figure 2c). Wounded leaves of *arc5* mutant showed a slight but non-significant upregulation of all three transcript levels in comparison to wild-type leaves only (Figure 2c). In contrast, wounding of leaves from the chloroplast positioning and movement mutants *kac1/2* and *chup1* did not result

in any significant differences in the transcript accumulation of the tested genes compared to the respective wild type.

To understand the biochemical basis of enhanced JA biosynthesis in *arc* mutants we quantified levels of MGDG and DGDG and their fatty acid composition in wild type and mutants. Analyzing them by TLC and GC-FID, total MGDG and DGDG content of both *arc* mutants was reduced by 30-40 % in comparison to wild-type plants (Figure 3a). The MGDG:DGDG ratio remained, however, unchanged (Figure S1), indicating coordinated reduced levels of both galactolipid classes in the *arc* mutants rather than selective MGDG-to-DGDG conversion. Detailed fatty acid profiling showed that this reduction was driven primarily by reduction in the levels of esterified α-linolenic acid (18:3), while other fatty acids (16:0, 16:1, 18:0, 20:0) remained unchanged (Figure 3b). The reduced 18:3 levels were observed in both MGDG and DGDG pools, thereby changing the levels of the predominant fatty acid in chloroplast galactolipids and the major substrate for JA biosynthesis. To elucidate changes in other lipid species, untargeted lipidomics of chloroplast lipid molecular species was performed and revealed additional membrane remodeling in *arc* mutants (Figure 3c, Supplementary data 1). Hierarchical clustering showed that *arc3* and *arc5* had distinct lipid profiles with a significant increase in some of the lipid classes, mainly triacylglycerides, Arabidopsides, phosphatidylglycerols and sulfoquinovosyldiacylglycerols (SQDG). A closer look at potential substrates of jasmonate biosynthesis showed significant changes in only few lipid species, which might serve as the primary reservoir of α-linolenic acid for jasmonate biosynthesis (Schaller *et al.* 2004), such as Arabidopside A and B as well as SQDG 18:3-18:3 and SQDG 16:0-16:3 (Figure 3d). In contrast, *kac1/2* and *chup1* mutant plants exhibited only small differences in lipid pools compared to Col-0, concomitant with their unaltered chloroplast size and number (Figure S2).

Giant chloroplasts in mesophyll cells of *arc3* and *arc5* mutants show altered thylakoid organization and increased AOC levels

The counterintuitive finding that *arc* mutants exhibit enhanced jasmonate production despite reduced galactolipid levels prompted us to examine whether structural alterations in the giant chloroplasts might contribute to their elevated metabolic activity. We employed transmission electron microscopy (TEM) to analyze whole leaf cross-sections and individual chloroplasts in both palisade and spongy parenchyma cells. Analyses on tissue level did not show differences in tissue organization and cell size but revealed the pronounced size differences between wild type and *arc* mutant chloroplasts (Figure S3). As already visualized by light microscopy (Figure 1), mesophyll cells of wild-type leaves contain numerous small, uniformly distributed chloroplasts, whereas mesophyll cells of *arc3* and *arc5* mutants showed substantially reduced chloroplast numbers with massively enlarged individual plastids that dominated the cellular space. On ultrastructural level, wild-type L*er* chloroplasts showed the typical organization with well-developed, tightly stacked thylakoid membranes forming distinct grana and interconnecting stroma lamellae (Figure 4). With that, the chloroplasts displayed compact, organized internal membrane systems with dense thylakoid stacking characteristic of photosynthetically active plastids. In both palisade and spongy parenchyma tissues, wild-type chloroplasts maintained consistent size and regular, oval morphology with uniform thylakoid distribution throughout the stroma. In contrast, the giant chloroplasts of *arc3* and *arc5* mutants displayed substantially altered internal organization. The enlarged plastids exhibited extensive regions with loosely organized thylakoid membranes, particularly evident in the peripheral areas of the chloroplasts. While some regions maintained organized grana stacks, large areas contained dispersed, less tightly packed thylakoids with increased spacing between membrane systems. Chloroplasts of both mutants showed heterogeneous internal organization with prominent stromal spaces, which appeared to be increased. Notably, the altered thylakoid architecture was consistent across both palisade and

spongy parenchyma cells, indicating that the ultrastructural changes were intrinsic to the giant chloroplast phenotype rather than cell-type specific.

To determine whether the higher appearance of stromal area in the giant chloroplasts translated to increased abundance of JA biosynthesis enzymes, we quantified AOC protein levels using western blot analysis (Figures 5, S4). Both *arc3* and *arc5* mutants showed significantly increased AOC protein levels compared to wild type. In contrast, *kac1/2* and *chup1* mutants showed no substantial differences in AOC protein abundance relative to their wild-type controls. These results indicate that chloroplast size and internal organization, rather than chloroplast positioning or number, is the critical determinant of jasmonate biosynthetic capacity following mechanical stress.

arc mutants exhibit enhanced touch sensitivity and delayed flowering

Jasmonates are not only involved in the plant's wound response, but also mediate touch responses and thigmomorphogenesis (Darwish *et al.* 2022, Van Moerkercke *et al.* 2019). This prompted us to assess the responses of *arc3*, *arc5* and L*er* plants to a reoccurring touch stimulus (Darwish *et al.* 2022) (Figure 6). Under non-touched conditions, wild-type, *arc3*, and *arc5* plants bolted at approximately 28, 31, and 32 days, respectively (Figure 6a, b). However, mechanical stimulation revealed substantial differences in touch sensitivity between genotypes, with *arc* mutants exhibiting enhanced sensitivity compared to wild-type plants. While wild-type plants showed only a modest delay in bolting (approximately 2 days) in response to touching, touch treatment delayed bolting by approximately 4-5 days in *arc3* and *arc5* mutants compared to their respective non-touched controls (Figure 6b). Furthermore, total leaf number analysis revealed that touched *arc5* plants produced significantly more leaves before bolting compared to other genotypes, indicating a more pronounced vegetative growth phase under mechanical stress

(Figure 6c). Interestingly, despite the pronounced effects on developmental timing, rosette area remained unchanged across all genotypes under mechanical stimulation, suggesting that the enhanced touch sensitivity in *arc* mutants specifically affects reproductive transition rather than overall vegetative growth (Figure S5). These results showed that the enhanced jasmonate biosynthetic capacity observed in *arc* mutants confers increased sensitivity to touch.

Discussion

Our findings show a novel relationship between chloroplast morphology and jasmonate biosynthetic capacity, demonstrating that organellar architecture directly influences hormone metabolism and environmental responsiveness. The enhanced jasmonate production in *arc* mutants upon wounding is particularly intriguing given the substantially reduced chloroplast numbers and, counterintuitively, significantly reduced galactolipid substrate levels. Repeatedly performed touch treatment resulted in an enhanced thigmomorphogenetic response in these mutants demonstrating a typical phenotype of jasmonate-overproducing plants. With this, the work establishes chloroplast morphology as a previously unrecognized determinant of jasmonate homeostasis driving the growth-defense tradeoff.

Arabidopsis *arc3* and *arc5* mutants possess defects in chloroplast division machinery, leading to cells containing only a few but greatly enlarged chloroplasts, compared with much smaller chloroplasts in wild-type plants (Pyke and Leech 1994), Figure 1). These mutants provide an excellent model for examining how plastid size influences their metabolic activity. It has been shown that cells with fewer and larger chloroplasts exhibit a lower mesophyll conductance and limited photosynthetic competence than cells with more and smaller chloroplasts (Austin II and Webber 2005, Weise *et al.* 2015). Moreover, chloroplasts from *arc* mutants differ in the abundance of proteins when compared to wild type, whereas the profile of the native stromal and

membrane complexes remains unchanged (Gargano *et al.* 2013). Here, we show another metabolic effect of enlarged chloroplasts: Leaves of *arc* mutants respond to wounding with an increased JA production, which is accompanied by enhanced transcript accumulation of JA-regulated genes, such as *AtLOX2*, *AtAOS* and *AtJAZ10* (Figure 2). The specificity of this effect is underscored by data obtained from the positioning mutants *chup1* and *kac1/2*, which contain a similar number of small chloroplasts and retain jasmonate levels like wild-type plants despite altered chloroplast distribution. However, the accumulation of *cis*-OPDA without corresponding JA/JA-Ile increases in the chloroplast positioning mutants suggests possible defects in *cis*-OPDA export or downstream conversion within the peroxisomes, warranting further study of chloroplast–peroxisome communication.

The lipidomics data showed a reduction of the MGDG and DGDG levels in *arc* mutants in comparison to wild type, while their ratio was not changed. This contrasts to stress responses, where MGDG are specifically converted to oligogalactolipids or even degraded, e.g., upon freezing or nitrogen starvation, respectively (Li *et al.* 2012, Moellering *et al.* 2010). The unaltered MGDG:DGDG ratio might contribute to unaltered membrane integrity, since their ratio maintains the critical balance between bilayer and non-bilayer phases in thylakoids (Demé *et al.* 2014, Kobayashi 2016), and its disruption causes severe photosynthetic impairment as shown for *mgd1* or *dgd1* mutants (Dörmann *et al.* 1995). By maintaining the MGDG:DGDG ratio through coordinated degradation, membrane architecture might be preserved in *arc* mutants, while fatty acids are channeled toward defense metabolism. Interestingly, *kac1/2* and *chup1* mutants show almost no lipidomic alterations and no JA elevation revealing that enlarged chloroplasts may create a unique metabolic state for channeling membrane components into defense signaling. Although substrate limitation is one hypothetical constraint for jasmonate biosynthesis (Wasternack and Hause 2013), the reduced MGDG and DGDG contents including the reduced levels in 18:3 fatty acid in *arc* mutants might still be sufficient to enable the enhanced jasmonate

production under wounding stress. Classical models predict reduced biosynthetic efficiency in enlarged organelles due to unfavorable surface area-to-volume ratios and diffusion limitations (Marshall 2016, Rafelski 2013). However, our findings show that giant chloroplasts maintain – or even enhance – specific metabolic functions despite substantial size increases, implying the presence of structural and kinetic compensation mechanisms that defy classical scaling expectations. It is tempting to speculate that enhanced JA production in *arc* mutants might occur through active substrate mobilization and/or higher substrate availability.

Structural alterations within the giant chloroplasts appear to be a central factor leading to enhanced jasmonate production. Such architecture-driven optimization was exemplified shown for mitochondrial fusion in steroidogenic cells promoting enzyme channeling (Duarte *et al.* 2012, Rone *et al.* 2009). Our ultrastructural analyses revealed that *arc* mutants harbor chloroplasts with loosely organized thylakoids and expanded membrane spacing, unlike the compact stacking in wild-type chloroplasts (Figure 4). This organization might generate membrane microdomains that enhance enzyme accessibility and metabolic flux. The more open thylakoid networks in giant chloroplasts of *arc* mutants may increase enzyme accessibility by reducing diffusion barriers, favoring processes such as jasmonate biosynthesis, which is spatially separated with localization of LOX on thylakoids and AOS/AOC on the inner envelope and stroma (Farmaki *et al.* 2007, Froehlich *et al.* 2001, Stenzel *et al.* 2012), but depends on substrate channeling (Schaller and Stintzi 2009). Moreover, *arc* mutants contain increased levels of AOC (Figure 5), thereby facilitating closer enzyme interactions and possibly leading to improved metabolic channeling needed for the synthesis of *cis*-OPDA (Schaller and Stintzi 2009).

Thigmomorphogenetic responses of *arc* mutants were enhanced in comparison to wild-type plants. Upon repeated brushing, *arc* mutants exhibited a 4-5-day bolting delay compared with the modest 2-day delay in wild type (Figures 6). Jasmonates have been shown to mediate touch responses, as JA levels increase in touched plants (Chehab *et al.* 2012, Tretner *et al.* 2008) and

JA-deficient or insensitive mutants (*aos*, *coi1*, *jar1*) completely lack touch responses (Chehab *et al.* 2012, Yan *et al.* 2007). The increased touch sensitivity of *arc* mutants is indicative for an enhanced JA-biosynthesis as demonstrated for plants overexpressing *OPR3* showing constitutive JA overproduction accompanied by enhanced touch responses (Chehab *et al.* 2012). Repeatedly performed touching stimulates JA biosynthesis resulting in a positive feedback loop further enhancing jasmonate levels and is accompanied by reduced growth and delayed flowering as the defense-growth-balance is altered (Van Moerkercke *et al.* 2019). Touch signaling operates, however, through two complementary branches: a JA-dependent pathway mediated by MYC2/3/4 transcription factors, and a CAMTA3-dependent and JA-independent pathway (Van Moerkercke *et al.* 2019). Moreover, thigmomorphogenesis involves hormonal crosstalk, notably JA-mediated gibberellin catabolism via upregulation of *GIBBERELLIN 2-OXIDASE7* encoding a protein that inactivates bioactive gibberellins (Fernandez-Moreno *et al.* 2024, Wang *et al.* 2023). Here, a reduced level of bioactive GA and an increased level of JA is required for the touch-mediated growth alteration (Chehab *et al.* 2012, Lange and Lange 2015). Enhanced jasmonate levels in *arc* mutants may preferentially affect the bolting time without major effects on vegetative growth (Figure S4) suggesting a more pronounced effect on reproductive developmental program than on general growth inhibition. This might be consistent with ecological models proposing thigmomorphogenesis as an adaptive mechanism for optimizing reproductive timing under mechanical stress (Coutand 2010, Moulia *et al.* 2015).

Natural variation in chloroplast size and morphology exists across plant species and even within populations, suggesting that morphology-dependent metabolic regulation could potentially serve as a mechanism for fine-tuning stress responses to local environmental conditions (Kadirjan-Kalbach *et al.* 2019). The effect of enlarged chloroplasts on the capacity of JA biosynthesis in rosette leaves of Arabidopsis demonstrated in our study represents another example of plant’s flexibility in their adaptation to stress conditions. The ability to enhance jasmonate production

through chloroplast architectural changes could provide resilience against multiple simultaneous stresses and may represent an evolutionary mechanism for environmental adaptation that operates independently of changes in biosynthetic gene sequences or expression levels.

Material and Methods

Plant growth, wounding and touch treatments

Seeds from *Arabidopsis thaliana* wild-type (ecotype Col-0 and L*er*) and the mutant lines *arc3* (Marrison *et al.* 1999), *arc5* (Pyke and Leech 1994), *chup1* (Oikawa *et al.* 2003) and *kac1/2* (Suetsugu *et al.* 2010) were stratified at 4°C for 2 days before grown individually in pots containing steam-sterilized clay, coir fiber, and vermiculite for four weeks. Plant growth was conducted in Phytocabinets (Percival Scientific, www.percival-scientific.com/) at a light intensity of 120 $\mu Em^{-2} s^{-1}$ under short-day-conditions (10/14 h light/dark cycle), at 21/19 °C and 65% relative humidity. Microscopical analyses, wounding by inflicting a single wound in the midrib, RNA extraction and hormone measurements were done using leaf No 8 of 4-week-old plants.

For touching experiments including determination of bolting time, two-week-old seedlings were gently brushed twice daily approximately 10 times with a soft art paint brush according to (Darwish *et al.* 2022). Touch treatment was applied for 18 to 26 days until all plants had bolted depending on the genotypes, that means when the inflorescence stem reached to 1-cm height. The rosette area of 31- to 35-day-old plants was measured using Fiji/ImageJ software.

Cell wall staining and Confocal Laser Scanning Microscopy

Leaf discs (20-25 per genotype) from the 8th leaf of 4-week-old plants were collected using a cork borer and stained with 0.01% (v/v) Renaissance 2200 (R2200; Renaissance Chemicals, UK)

under gentle vacuum infiltration for 10 min, followed by two washing steps with distilled water. Leaf disks were analyzed using a Zeiss LSM900 (Carl Zeiss GmbH, http://www.zeiss.com) with excitation/emission at 405 nm/415-450 nm and 633nm/650-700 nm for R2200 and chlorophyll, respectively. Z-stack images were acquired using identical settings for all samples and image correction was performed in Zeiss ZEN Blue. 3D reconstructions were analyzed using Arivis Vision4D software (Arivis AG, Germany), where mesophyll cells were defined as parent objects and chloroplasts as child objects based on their respective fluorescence signals. This parent-child segmentation approach enabled quantification of cell volume, surface area, and chloroplast number per cell from a minimum of 50 cells per genotype.

Hormone measurements

Measurements of *cis*-OPDA, JA, and JA-Ile were performed using a standardized Ultra-Performance Liquid Chromatography-tandem Mass Spectrometry (UPLC– MS/MS)-based method (Balcke *et al.* 2012). For this, 50 mg of powdered frozen tissue were extracted with 250 µl 100% LC-MS methanol supplemented with internal standards (5 ng/sample each of [$^2H_5$]OPDA, [$^2H_6$]JA, and [$^2H_2$]JA-Ile). Following a purification step done by solid-phase extraction on HR-XC (Chromabond, Macherey-Nagel, www.mn-net.com), 10 µl of the eluate were analyzed via UPLC–MS/MS, and analyte content was determined relative to the internal standard peak heights.

RNA isolation and quantitative RT-PCR analysis

Total RNA was extracted from homogenized frozen plant material using the RNeasy Plant Mini Kit (Qiagen), followed by removal of genomic DNA with the DNA-free™ DNA Removal Kit (Invitrogen, #AM1906). First-strand cDNA synthesis was performed using 1 µg of DNA-free RNA with the RevertAid H Minus Reverse Transcriptase and oligo(dT)$_{18}$ primers (ThermoFisher

Scientific™, www.thermofisher.com). Quantitative PCR was carried out according to (Mekkaoui *et al.* 2025) and using the primers given there. Gene expression was normalized to the housekeeping gene *PROTEIN PHOSPHATASE 2 A SUBUNIT A*3 (AT1G13320) using the $2^{-\Delta CT}$ method (Schmittgen and Livak 2008) and included at least three biological replicates.

MGDG/DGDG measurements and untargeted lipid analytics

For determination of MGDG and DGDG contents, total lipids were extracted from 50 mg fresh weight tissue using a modified Bligh and Dyer method (Bligh and Dyer 1959) with chloroform:methanol:0.15M NaCl (1:2:1, v/v/v). After phase separation with chloroform, the organic phase was collected, the aqueous phase was re-extracted twice and organic phases were combined, dried under nitrogen, and dissolved in chloroform. Sample was split and one fifth volume was used for total fatty acid analysis and the rest for analysis for individual galactolipid classes. Galactolipids (MGDG and DGDG) were separated by TLC using acetone:toluene:water (90:30:7, v/v/v). The lipids were sprayed with 0.02% (w/v) primuline solution (acetone:water, 4:1, v/v) and visualized by UV light. For quantification, tripentadecanoin was used as the internal standard. MGDG and DGDG were isolated from the TLC plates and fatty acids were transesterified with 0.5 M sodium methoxide in toluene:methanol (1:2, v/v), extracted with n-hexane after NaCl quenching, evaporated and dissolved in 50 µL acetonitrile.

GC-FID analysis was performed on a Shimadzu GC-2010plus system with a DB-23 column (30 m × 0.25 mm × 0.25 µm). Operating conditions: split injection (5:1) at 220°C, helium carrier gas at 1 mL/min, the temperature gradient was 150°C for 1 min, 150°C to 200°C at a rate of 25°C min^{-1}, from 200°C to 250°C at 4°C min^{-1} , 250°C for 6 min. FAMEs were identified by comparison with menhaden oil FAMEs as authentic standard and quantified using the internal standard. Three biological replicates were analyzed per treatment.

Untargeted lipid analytics was performed following (Salem *et al.* 2016). Briefly, frozen leaves (30 mg fresh weight) were homogenized and extracted with 500 µL pre-cooled methyl tert-butyl ether (MTBE):methanol (3:1, v/v). An internal standard of deuterated 1-pentadecanoyl-2-oleoyl(d7)-sn-glycero-3-phosphoethanolamine (15:0-18:1-d7-PE) was added into each sample to end up with a concentration of 1 µM. After vortexing (30 min, 4°C) and sonication (15 min), phase separation was induced with water:methanol (3:1, v/v). Following centrifugation (20,000 × g, 10 min, 4°C), the upper phase was dried under nitrogen and reconstituted in 300 µL acetonitrile:isopropanol (7:3, v/v). Eight biological replicates were analyzed per treatment.

LC-MS/MS analysis was performed on an Agilent 1290 UHPLC coupled to a Bruker timsTOF Pro equipped with a VIP-HESI source. Separation employed a Bruker Intensity Solo C18 column (100 × 2.1 mm, 1.8 µm) with gradient elution from 40% to 99% B over 8.5 min (mobile phase A: acetonitrile:water 60:40, v/v; B: isopropanol:acetonitrile 90:10, v/v; both containing 0.1% formic acid and 10 mM ammonium formate) at 0.45 mL/min. MS parameters: positive ionization mode, m/z 100-1350, ion mobility $1/K_0$ 0.70-1.80 $V \cdot s \cdot cm^{-2}$. Data was processed using Bruker MetaboScape v2025b with the integrated Lipid Annotation Tool for compound classification and structural confirmation. The intensities of the compounds in each sample were then normalized to the intensity of the internal standard in that sample. Compounds showing statistically significant differences between wild type and mutants according to Student's t-test (see Supporting information Dataset 1) were log transformed and plotted in heatmaps.

Protein extraction and immune blotting

Extraction of total proteins from leaves, separation by PAGE and immunoblotting were performed according to (Mekkaoui *et al.* 2025). Quantification of AOC protein level was done using a rabbit anti-AtAOC antibody (Stenzel *et al.* 2003) in relation to β-actin, which was detected using a mouse

anti-actin antibody (A0480, Sigma-Aldrich). As secondary antibodies, anti-rabbit or anti-mouse IgG antibodies conjugated with alkaline phosphatase (1:4000, Sigma-Aldrich) were used followed by incubation in the Immun-Star™ AP (BioRad) substrate following the manufacturer´s instructions.

Electron microscopy

Leaf discs of 2 mm diameter were dissected from the middle of a fully developed leaf of plants at bolting stage and immediately fixed in 3 % (v/v) glutaraldehyde in cacodylate buffer (pH 7.2) for 2 h. After post-fixation with 2% osmium tetroxide, samples were dehydrated in an ethanol series and embedded in epoxy resin (Spurr 1969). Semithin sections (1 µm) were stained with toluidine blue and micrographs were taken using a Leica DMRB microscope equipped with a Leica DFC 450 camera (Leica Microsystems, http://www.leica-microsystems.com). Ultrathin sections (90 nm) were stained with uranyl acetate/lead citrate and observed with an EM 900 transmission electron microscope (Zeiss). Micrographs were processed through PHOTOSHOP 12.0.4 (Adobe Systems, http://www.adobe.com).

Statistical analysis

The statistical analyses applied to the different datasets as indicated in the figures were performed using GraphPad Prism (www.graphpad.com).

Acknowledgments

We thank Simone Fraas (Martin-Luther-University Halle-Wittenberg) for performing embedding and sectioning of Arabidopsis leaves, and Dr. Martin Schattat (Martin-Luther-University Halle-Wittenberg) for providing seeds of *arc3*, *arc5*, *kac1kac2* and *chup1* mutants. Claus Wasternack (IPB Halle) is highly acknowledged for critical reading of the manuscript. This work was supported by the Deutsche Forschungsgemeinschaft (DFG, German Research Foundation) grant No 400681449/GRK2498, project P02.

Author contributions

RB and HS designed experiments with input from JZ and BH. RB, HS, MH, SK and SNK performed experiments. RB, MH, SK and SNK analyzed the data and performed statistical analyses. RB and BH wrote the manuscript with suggestions and approval of all authors.

Conflict of Interest Statement

No conflict of interest declared.

Data availability statement

Seeds and data underlying the figures are available upon request from Ranjit Baral (ranjit.baral@ipb-halle.de) or Bettina Hause (bettina.hause@ipb-halle.de).

Supporting information:

Additional Supporting Information may be found in the online version of this article.

Figure S1. Ratio of MGDG and DGDG in rosette leaves from L*er*, *arc3* and *arc5*.

Figure S2. Hierarchical clustering of lipid profiles from *kac1/2*, *chup1* and their wild type Col-0.

Figure S3. Tissue-level organization of rosette leaves in wild type and *arc* mutants.

Figure S4. Western blot analysis of AOC protein levels in chloroplast mutants.

Figure S5. Touch treatment does not alter rosette area.

Data set 1. Non-targeted lipid analysis.

References

Acosta, I.F., Gasperini, D., Chételat, A., Stolz, S., Santuari, L. and Farmer, E.E. (2013) Role of NINJA in root jasmonate signaling. *Proceedings of the National Academy of Sciences*, **110**, 15473-15478.

Austin II, J. and Webber, A.N. (2005) Photosynthesis in *Arabidopsis thaliana* mutants with reduced chloroplast number. *Photosynthesis Research*, **85**, 373-384.

Balcke, G., Handrick, V., Bergau, N., Fichtner, M., Henning, A., Stellmach, H., Tissier, A., Hause, B. and Frolov, A. (2012) An UPLC-MS/MS method for highly sensitive high-throughput analysis of phytohormones in plant tissues. *Plant Methods*, **8**, 47.

Bligh, E.G. and Dyer, W.J. (1959) A rapid method of total lipid extraction and purification. *Can J Biochem Physiol*, **37**, 911-917.

Chauvin, A., Lenglet, A., Wolfender, J.-L. and Farmer, E. (2016) Paired hierarchical organization of 13-lipoxygenases in Arabidopsis. *Plants*, **5**, 16.

Chehab, E.W., Yao, C., Henderson, Z., Kim, S. and Braam, J. (2012) Arabidopsis touch-induced morphogenesis is jasmonate mediated and protects against pests. *Current Biology*, **22**, 701-706.

Chini, A., Monte, I., Zamarreño, A.M., Hamberg, M., Lassueur, S., Reymond, P., Weiss, S., Stintzi, A., Schaller, A., Porzel, A., García-Mina, J.M. and Solano, R. (2018) An OPR3-independent pathway uses 4,5-didehydrojasmonate for jasmonate synthesis. *Nature Chemical Biology*, **14**, 171.

Coutand, C. (2010) Mechanosensing and thigmomorphogenesis, a physiological and biomechanical point of view. *Plant Science*, **179**, 168-182.

Darwish, E., Ghosh, R., Ontiveros-Cisneros, A., Tran, H.C., Petersson, M., De Milde, L., Broda, M., Goossens, A., Van Moerkercke, A., Khan, K. and Van Aken, O. (2022) Touch signaling and thigmomorphogenesis are regulated by complementary CAMTA3- and JA-dependent pathways. *Sci Adv*, **8**, eabm2091.

Demé, B., Cataye, C., Block, M.A., Maréchal, E. and Jouhet, J. (2014) Contribution of galactoglycerolipids to the 3-dimensional architecture of thylakoids. *The FASEB Journal*, **28**, 3373-3383.

Dörmann, P., Hoffmann-Benning, S., Balbo, I. and Benning, C. (1995) Isolation and characterization of an Arabidopsis mutant deficient in the thylakoid lipid digalactosyl diacylglycerol. *Plant Cell*, **7**, 1801-1810.

Du, W., Cao, L., Zhou, Y., Jackson, S., Naeem, M., Chen, Z., Yang, Y., Glynn, J.M., Porter, K.J., Zhang, R., He, Q., Liu, M., Xu, J., Liang, W., Feng, P., Osteryoung, K.W. and Chen, C. (2025) Enhanced chloroplast FtsZ-ring constriction by the ARC6–ARC3 module in *Arabidopsis*. *Proceedings of the National Academy of Sciences*, **122**, e2425129122.

Duarte, A., Poderoso, C., Cooke, M., Soria, G., Cornejo Maciel, F., Gottifredi, V. and Podestá, E.J. (2012) Mitochondrial fusion is essential for steroid biosynthesis. *PLoS One*, **7**, e45829.

Farmaki, T., Sanmartin, M., Jimenez, P., Paneque, M., Sanz, C., Vancanneyt, G., Leon, J. and Sanchez-Serrano, J.J. (2007) Differential distribution of the lipoxygenase pathway enzymes within potato chloroplasts. *J. Exp. Bot.*, **58**, 555-568.

Fernandez-Moreno, J.-P., Yaschenko, A.E., Neubauer, M., Marchi, A.J., Zhao, C., Ascencio-Ibanez, J.T., Alonso, J.M. and Stepanova, A.N. (2024) A rapid and scalable approach to build synthetic repetitive hormone-responsive promoters. *Plant Biotechnology Journal*, **22**, 1942-1956.

Froehlich, J., Itoh, A. and Howe, G. (2001) Tomato allene oxide synthase and fatty acid hydroperoxied lyase, two cytochrome P450s involved in oxylipin metabolism, are targeted to different membranes of chloroplast envelope. *Plant Physiol.*, **125**, 306-317.

Gao, H., Kadirjan-Kalbach, D., Froehlich, J.E. and Osteryoung, K.W. (2003) ARC5, a cytosolic dynamin-like protein from plants, is part of the chloroplast division machinery. *Proceedings of the National Academy of Sciences*, **100**, 4328-4333.

Gargano, D., Maple-Grødem, J., Reisinger, V., Eichacker, L.A. and Møller, S.G. (2013) Analysis of the chloroplast proteome in arc mutants and identification of novel protein components associated with FtsZ2. *Plant Molecular Biology*, **81**, 235-244.

Ghosh, P.P., Kunjumon, T.K. and Mathur, J. (2025) Binary division of plastids involves endoplasmic reticulum mediation. *Journal of Experimental Botany*.

Holzinger, A., Kwok, E.Y. and Hanson, M.R. (2008) Effects of *arc3*, *arc5* and *arc6* mutations on plastid morphology and stromule formation in green and nongreen tissues of *Arabidopsis thaliana*. *Photochemistry and Photobiology*, **84**, 1324-1335.

Howe, G.A., Major, I.T. and Koo, A.J. (2018) Modularity in jasmonate signaling for multistress resilience. *Annual Review of Plant Biology*, **69**, 387-415.

Ibrahim, A., Schütz, A.-L., Galano, J.-M., Herrfurth, C., Feussner, K., Durand, T., Brodhun, F. and Feussner, I. (2011) The alphabet of galactolipids in *Arabidopsis thaliana*. *Frontiers in Plant Science*, **Volume 2 - 2011**.

Jarvis, P. and López-Juez, E. (2013) Biogenesis and homeostasis of chloroplasts and other plastids. *Nat Rev Mol Cell Biol*, **14**, 787-802.

Johnson, M.P. and Wientjes, E. (2020) The relevance of dynamic thylakoid organisation to photosynthetic regulation. *Biochimica et Biophysica Acta (BBA) - Bioenergetics*, **1861**, 148039.

Kadirjan-Kalbach, D.K., Turmo, A., Wang, J., Smith, B.C., Chen, C., Porter, K.J., Childs, K.L., DellaPenna, D. and Osteryoung, K.W. (2019) Allelic variation in the chloroplast division gene *FtsZ2-2* leads to natural variation in chloroplast size. *Plant Physiology*, **181**, 1059-1074.

Kirchhoff, H. (2019) Chloroplast ultrastructure in plants. *New Phytologist*, **223**, 565-574.

Kobayashi, K. (2016) Role of membrane glycerolipids in photosynthesis, thylakoid biogenesis and chloroplast development. *Journal of Plant Research*, **129**, 565-580.

Lange, M.J.P. and Lange, T. (2015) Touch-induced changes in Arabidopsis morphology dependent on gibberellin breakdown. *Nature Plants*, **1**, 14025.

Li, X., Moellering, E.R., Liu, B., Johnny, C., Fedewa, M., Sears, B.B., Kuo, M.-H. and Benning, C. (2012) A galactoglycerolipid lipase is required for triacylglycerol accumulation and survival following nitrogen deprivation in *Chlamydomonas reinhardtii* *The Plant Cell*, **24**, 4670-4686.

Lin, Y.-T., Chen, L.-J., Herrfurth, C., Feussner, I. and Li, H.-m. (2016) Reduced biosynthesis of digalactosyldiacylglycerol, a major chloroplast membrane lipid, leads to oxylipin overproduction and phloem cap lignification in Arabidopsis. *The Plant Cell*, **28**, 219-232.

Maple, J., Vojta, L., Soll, J. and Møller, S.G. (2007) ARC3 is a stromal Z-ring accessory protein essential for plastid division. *EMBO reports*, **8**, 293-299.

Marrison, J.L., Rutherford, S.M., Robertson, E.J., Lister, C., Dean, C. and Leech, R.M. (1999) The distinctive roles of five different ARC genes in the chloroplast division process in Arabidopsis. *The Plant Journal*, **18**, 651-662.

Marshall, W.F. (2016) Cell geometry: How cells count and measure size. *Annu Rev Biophys*, **45**, 49-64.

Mekkaoui, K., Baral, R., Smith, F., Klein, M., Feussner, I. and Hause, B. (2025) Transcriptomics and trans-organellar complementation reveal limited signaling of 12-*cis*-oxo-phytodienoic acid during early wound response in *Arabidopsis*. *Nature Communications*, **16**, 6684.

Miyagishima, S.-y., Froehlich, J.E. and Osteryoung, K.W. (2006) PDV1 and PDV2 mediate recruitment of the dynamin-related protein ARC5 to the plastid division site. *The Plant Cell*, **18**, 2517-2530.

Moellering, E.R., Muthan, B. and Benning, C. (2010) Freezing tolerance in plants requires lipid remodeling at the outer chloroplast membrane. *Science*, **330**, 226-228.

Moulia, B., Coutand, C. and Julien, J.-L. (2015) Mechanosensitive control of plant growth: bearing the load, sensing, transducing, and responding. *Frontiers in Plant Science*, **Volume 6 - 2015**.

Oikawa, K., Kasahara, M., Kiyosue, T., Kagawa, T., Suetsugu, N., Takahashi, F., Kanegae, T., Niwa, Y., Kadota, A. and Wada, M. (2003) CHLOROPLAST UNUSUAL POSITIONING1 is essential for proper chloroplast positioning. *The Plant Cell*, **15**, 2805-2815.

Oikawa, K., Yamasato, A., Kong, S.-G., Kasahara, M., Nakai, M., Takahashi, F., Ogura, Y., Kagawa, T. and Wada, M. (2008) Chloroplast outer envelope protein CHUP1 is essential for chloroplast anchorage to the plasma membrane and chloroplast movement *Plant Physiology*, **148**, 829-842.

Osteryoung, K.W. and Pyke, K.A. (2014) Division and dynamic morphology of plastids. *Annual Review of Plant Biology*, **65**, 443-472.

Pyke, K.A. and Leech, R.M. (1994) A genetic analysis of chloroplast division and expansion in *Arabidopsis thaliana*. *Plant Physiology*, **104**, 201-207.

Rafelski, S.M. (2013) Mitochondrial network morphology: building an integrative, geometrical view. *BMC Biology*, **11**, 71.

Robertson, E.J., Pyke, K.A. and Leech, R.M. (1995) *arc6*, an extreme chloroplast division mutant of *Arabidopsis* also alters proplastid proliferation and morphology in shoot and root apices. *Journal of Cell Science*, **108**, 2937-2944.

Rone, M.B., Fan, J. and Papadopoulos, V. (2009) Cholesterol transport in steroid biosynthesis: Role of protein–protein interactions and implications in disease states. *Biochimica et Biophysica Acta (BBA) - Molecular and Cell Biology of Lipids*, **1791**, 646-658.

Salem, M.A., Jüppner, J., Bajdzienko, K. and Giavalisco, P. (2016) Protocol: a fast, comprehensive and reproducible one-step extraction method for the rapid preparation of polar and semi-polar metabolites, lipids, proteins, starch and cell wall polymers from a single sample. *Plant Methods*, **12**, 45.

Schaller, A. and Stintzi, A. (2009) Enzymes in jasmonate biosynthesis - Structure, function, regulation. *Phytochemistry*, **70**, 1532-1538.

Schaller, F., Schaller, A. and Stintzi, A. (2004) Biosynthesis and metabolism of jasmonates. *Journal of Plant Growth Regulation*, **23**, 179-199.

Schmittgen, T.D. and Livak, K.J. (2008) Analyzing real-time PCR data by the comparative C(T) method. *Nature Protocols*, **3**, 1101-1108.

Schwenkert, S., Fernie, A.R., Geigenberger, P., Leister, D., Möhlmann, T., Naranjo, B. and Neuhaus, H.E. (2022) Chloroplasts are key players to cope with light and temperature stress. *Trends in Plant Science*, **27**, 577-587.

Spurr, A. (1969) A low-viscosity epoxy resin embedding medium for electron microscopy. *J. Ultrastructure Res.*, **26**, 31–43.

Staswick, P., Tiryaki, I. and Rowe, M. (2002) Jasmonate response locus *JAR1* and several related Arabidopsis genes encode enzymes of the firefly luciferase superfamily that show

activity on jasmonic, salicylic, and indole-3-acetic acids in an assay for adenylation. *The Plant Cell*, **14**, 1405-1415.

Stenzel, I., Hause, B., Miersch, O., Kurz, T., Maucher, H., Weichert, H., Ziegler, J., Feussner, I. and Wasternack, C. (2003) Jasmonate biosynthesis and the allene oxide cyclase family of *Arabidopsis thaliana*. *Plant Mol. Biol.*, **51**, 895-911.

Stenzel, I., Otto, M., Delker, C., Kirmse, N., Schmidt, D., Miersch, O., Hause, B. and Wasternack, C. (2012) *ALLENE OXIDE CYCLASE* (*AOC*) gene family members of *Arabidopsis thaliana*: tissue- and organ-specific promoter activities and *in vivo* heteromerization. *Journal of Experimental Botany*, **63**, 6125-6138.

Stintzi, A. and Browse, J. (2000) The Arabidopsis male-sterile mutant, *opr3*, lacks the 12-oxophytodienoic acid reductase required for jasmonate synthesis. *Proc. Natl. Acad. Sci. USA*, **97**, 10625-10630.

Suetsugu, N., Yamada, N., Kagawa, T., Yonekura, H., Uyeda, T.Q.P., Kadota, A. and Wada, M. (2010) Two kinesin-like proteins mediate actin-based chloroplast movement in *Arabidopsis thaliana*. *Proceedings of the National Academy of Sciences*, **107**, 8860-8865.

Sweetlove, L.J. and Fernie, A.R. (2013) The spatial organization of metabolism within the plant cell. *Annu Rev Plant Biol*, **64**, 723-746.

Tretner, C., Huth, U. and Hause, B. (2008) Mechanostimulation of *Medicago truncatula* leads to enhanced levels of jasmonic acid. *J. Exp. Bot.*, **59**, 2847-2856.

Van Moerkercke, A., Duncan, O., Zander, M., Šimura, J., Broda, M., Vanden Bossche, R., Lewsey, M.G., Lama, S., Singh, K.B., Ljung, K., Ecker, J.R., Goossens, A., Millar, A.H. and Van Aken, O. (2019) A MYC2/MYC3/MYC4-dependent transcription factor network regulates water spray-responsive gene expression and jasmonate levels. *Proceedings of the National Academy of Sciences*, **116**, 23345-23356.

Wang, K., Guo, Q., Froehlich, J.E., Hersh, H.L., Zienkiewicz, A., Howe, G.A. and Benning, C. (2018) Two abscisic acid-responsive plastid lipase genes involved in jasmonic acid biosynthesis in *Arabidopsis thaliana*. *The Plant Cell*, **30**, 1006-1022.

Wang, L., Ma, C., Wang, S., Yang, F., Sun, Y., Tang, J., Luo, J. and Wu, J. (2023) Ethylene and jasmonate signaling converge on gibberellin catabolism during thigmomorphogenesis in Arabidopsis. *Plant Physiology*, **194**, 758-773.

Wasternack, C. and Hause, B. (2013) Jasmonates: biosynthesis, perception, signal transduction and action in plant stress response, growth and development. An update to the 2007 review in Annals of Botany. *Ann Bot*, **111**, 1021-1058.

Wasternack, C. and Hause, B. (2018) A bypass in jasmonate biosynthesis - the OPR3-independent formation. *Trends in Plant Science*, **23**, 276-279.

Wasternack, C. and Song, S. (2017) Jasmonates: biosynthesis, metabolism, and signaling by proteins activating and repressing transcription. *Journal of Experimental Botany*, **68**, 1303-1321.

Weise, S.E., Carr, D.J., Bourke, A.M., Hanson, D.T., Swarthout, D. and Sharkey, T.D. (2015) The *arc* mutants of *Arabidopsis* with fewer large chloroplasts have a lower mesophyll conductance. *Photosynthesis Research*, **124**, 117-126.

Yan, Y., Stolz, S., Chetelat, A., Reymond, P., Pagni, M., Dubugnon, L. and Farmer, E.E. (2007) A downstream mediator in the growth repression limb of the jasmonate pathway. *Plant Cell*, **19**, 2470-2483.

Yu, C.-W., Lin, Y.-T. and Li, H.-m. (2020) Increased ratio of galactolipid MGDG : DGDG induces jasmonic acid overproduction and changes chloroplast shape. *New Phytologist*, **228**, 1327-1335.

Zhang, F., Yao, J., Ke, J., Zhang, L., Lam, V.Q., Xin, X.-F., Zhou, X.E., Chen, J., Brunzelle, J., Griffin, P.R., Zhou, M., Xu, H.E., Melcher, K. and He, S.Y. (2015) Structural basis of

JAZ repression of MYC transcription factors in jasmonate signalling. *Nature*, **525**, 269-273.

Zhang, M., Schmitz, A.J., Kadirjan-Kalbach, D.K., TerBush, A.D. and Osteryoung, K.W. (2013) Chloroplast division protein ARC3 regulates chloroplast FtsZ-ring assembly and positioning in *Arabidopsis* through interaction with FtsZ2 *The Plant Cell*, **25**, 1787-1802.

Figure legends:

Figure 1. 3D analysis of chloroplast morphology in mesophyll cells of chloroplast division and positioning mutants.

(a) Representative confocal microscopy images after 3D reconstruction and parent-child segmentation showing chloroplast distribution and morphology in mesophyll cells of L*er*, *arc3*, *arc5*, Col-0, *kac1/2*, and *chup1* mutants. The rosette leaf No 8 of 4-week-old plants was stained with R2200 for visualization of cell walls (turquoise), and chloroplasts were visualized by chlorophyll autofluorescence (red). Bars represent 10 µm. **(b)** Chloroplast number per mesophyll cell. **(c)** Chloroplast total surface area. **(d)** Chloroplast volume. **(e)** Chloroplast sphericity. Box plots display median, first and third quartiles, with whiskers drawn according to Tukey method. Dots represent outliers. Different letters indicate statistically significant differences between mutants and their respective wild type according to one-way ANOVA followed by Tukey's HSD test ($p < 0.05$) with $n = 50$ cells and their respective number of chloroplasts per genotype.

Figure 2. Enhanced jasmonate production in *arc* mutants after wounding.

(a, b) Levels of *cis*-OPDA, JA, and JA-Ile under basal conditions (light bars) and one hour after wounding (dark bars) in (**a**) L*er*, *arc3*, and *arc5* and (**b**) Col-0, *chup1*, and *kac1/2* rosette leaves of 4-week-old plants grown under short-day conditions. **(c)** Transcript accumulation of *LOX2*, *AOS*, and *JAZ10* in wounded plants normalized to *AtPP2A*. Data are presented as means ± SE ($n = 5$ biological replicates and $n = 3$-4 biological replicates with 3 plants pooled per replicate for a,b and c, respectively). Asterisks indicate statistically significant differences compared to respective wild-type levels (*$p < 0.05$, **$p < 0.01$, ***$p < 0.001$; one-way ANOVA followed by Tukey's HSD test). ns, not significant.

Figure 3. Lipidomic profiling reveals remodeling of chloroplast membrane lipids in *arc* mutants.

(a) Quantification of total MGDG and DGDG content. Lipids were extracted from leaf tissue, separated by thin-layer chromatography, and individual galactolipid bands were recovered and transesterified to fatty acid methyl esters for GC-MS quantification. Values represent total galactolipid content expressed as μg mg^{-1} fresh weight (FW). Data are presented as means ± SD (n = 3-4 biological replicates). Different letters denote statistically significant differences according to one-way ANOVA followed by Tukey's multiple comparison test ($p < 0.05$). **(b)** Fatty acid composition of total lipid extracts determined by GC-MS analysis. Fatty acid species are designated by the number of carbons and double bonds and given as μg mg^{-1} FW. Data are presented as means ± SD (n = 3-4 biological replicates). Statistical comparisons were performed for each fatty acid species separately using one-way ANOVA followed by Tukey's multiple comparison test. Asterisks indicate statistically significant differences between genotypes (***$p < 0.0001$). **(c)** Heatmap showing hierarchical clustering of lipid compound classes (CC) in wild type L*er* and *arc* mutants (n = 5 biological replicates per genotype). Each row represents an individual lipid species indicated by color-coded bars (right) and columns represent genotypes. Hierarchical clustering was performed using Euclidean distance and complete linkage methods using compounds showing significant differences between wild type and mutants. The heatmap displays row-wise z-score normalized abundances with red indicating elevated abundance and blue indicating reduced abundance as defined in “Scaled response” at the right. **(b)** Heatmap showing individual molecular species within major chloroplast lipid classes including arabidopsides, phosphatidylglycerol (PG), and sulfoquinovosyldiacylglycerol (SQDG). Lipid molecular species are annotated with acyl chain compositions (carbon number:number of double bonds). Compound classes are indicated by color-coded bars in the right margin and are displayed as row-wise z-score normalized abundances as defined in “Scaled response” at the right. Raw data for (**c**) and (**d**) are provided in Supporting information Dataset 1.

Figure 4. Mesophyll chloroplasts of *arc* mutants show altered thylakoid organization. Representative transmission electron micrographs showing chloroplast ultrastructure in palisade parenchyma **(a)** and spongy parenchyma **(b)** cells of wild type L*er*, *arc3*, and *arc5* plants. Overview micrographs of chloroplasts (left) and details of thylakoid membranes (right) are presented. Note that wild-type chloroplasts exhibit tightly stacked thylakoid membranes forming distinct grana, while *arc* mutant chloroplasts display loosely organized thylakoids with increased stromal spaces. Scale bars indicate 2.5 μm for overview pictures (left) and 0.5 μm for details (right).

Figure 5. *arc* mutants exhibit increased AOC protein abundance.

Relative AOC protein levels in chloroplast division mutants (*arc3*, *arc5*) and chloroplast positioning mutants (*chup1*, *kac1/2*) compared to their respective wild-type controls, determined by Western blot analysis and normalized to β-actin as loading control. Data are presented as means ± SE (n = 3). Asterisks indicate statistically significant differences compared to respective wild-type controls (**p < 0.01, ***p < 0.001; one-way ANOVA followed by Tukey's HSD test). ns, not significant. Underlying immune blots are shown in Figure S4.

Figure 6. *arc* mutants exhibit delayed flowering and enhanced thigmomorphogenetic responses.

(a) Representative images of L*er*, *arc3*, and *arc5* plants at bolting time. Plants were grown under long-day conditions and remained either non-touched (upper panels) or were touched daily twice with a soft brush starting on day 14 after sowing. **(b)** Day of bolting of L*er*, *arc3*, and *arc5* plants either non-touched or touched. Both *arc* mutants exhibited enhanced sensitivity to mechanical stimulation as touch treatment delayed bolting by 4-5 days compared to their respective non-touched controls, whereas L*er* plants showed less delay. **(c)** Number of rosette leaves of L*er*, *arc3*, and *arc5* plants at bolting time either non-touched or touched. Note that *arc5* plants

developed significantly more leaves before bolting compared to wild type under both conditions indicating prolonged vegetative growth. Data are shown as box and whisker blots. Different letters indicate statistically significant differences ($p < 0.05$) according to one-way ANOVA followed by Tukey's HSD test ($n = 33$ for non-touched plants, $n = 65$ for touched plants).

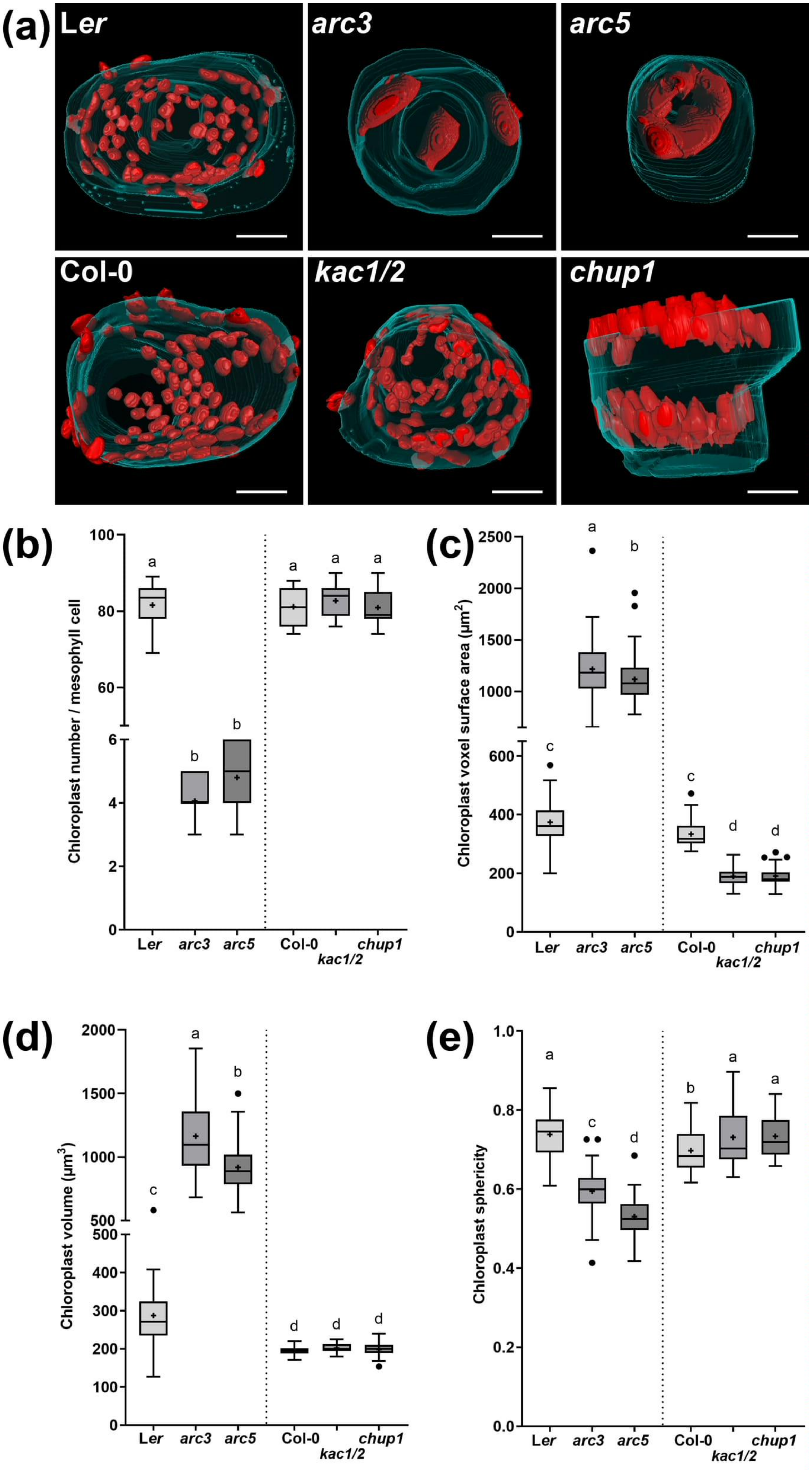
(a)
Ler
arc3
arc5
Col-0
kac1/2
chup1
(b)
Chloroplast number / mesophyll cell
Ler
arc3
arc5
Col-0
kac1/2
chup1
(c)
Chloroplast voxel surface area (μm2)
(d)
Chloroplast volume (μm3)
(e)
Chloroplast sphericity

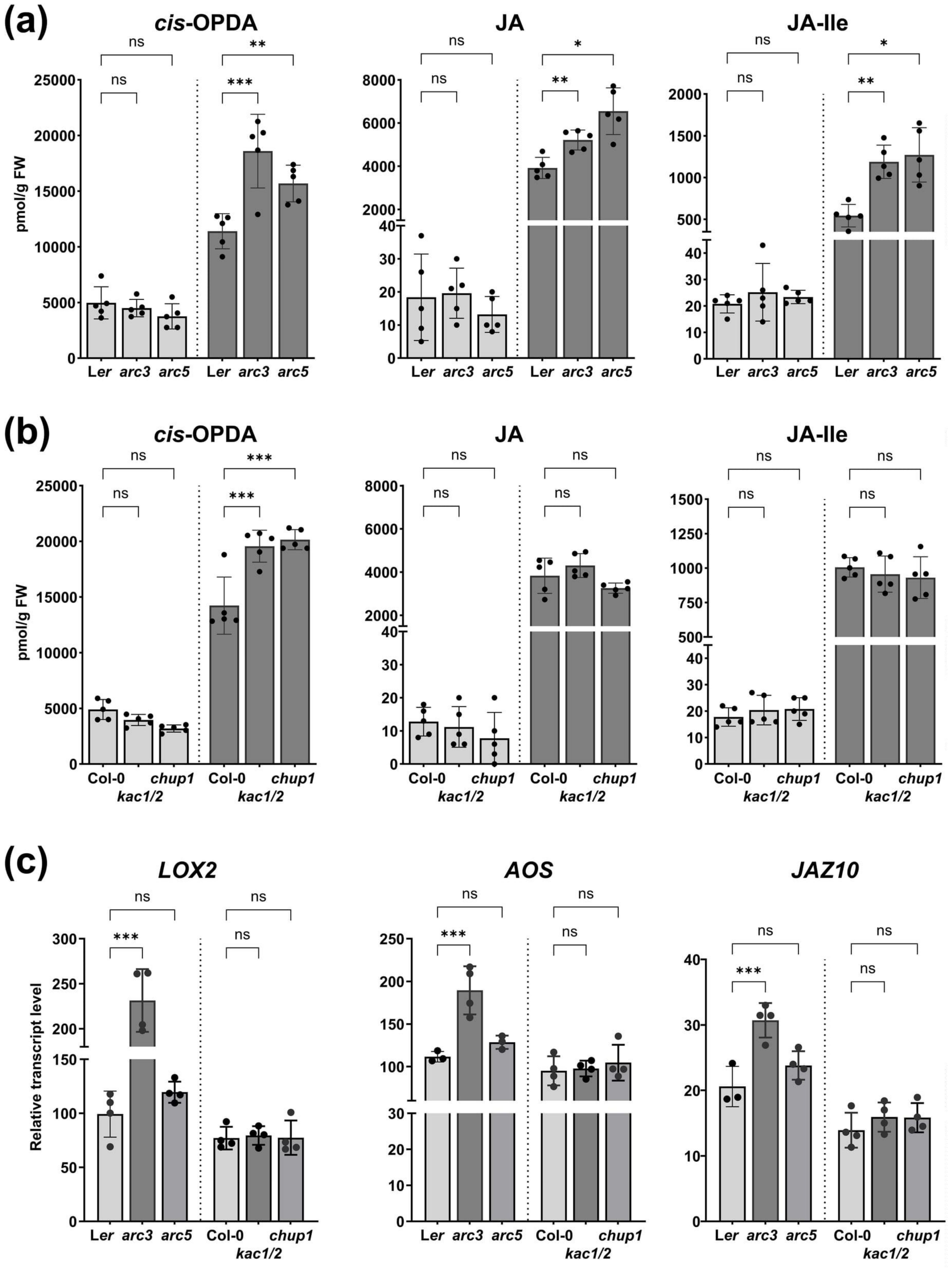

(a)
cis-OPDA
JA
JA-Ile
pmol/g FW
ns
**
***
*
Ler arc3 arc5 Ler arc3 arc5
(b)
Col-0 chup1
kac1/2
(c)
LOX2
AOS
JAZ10
Relative transcript level

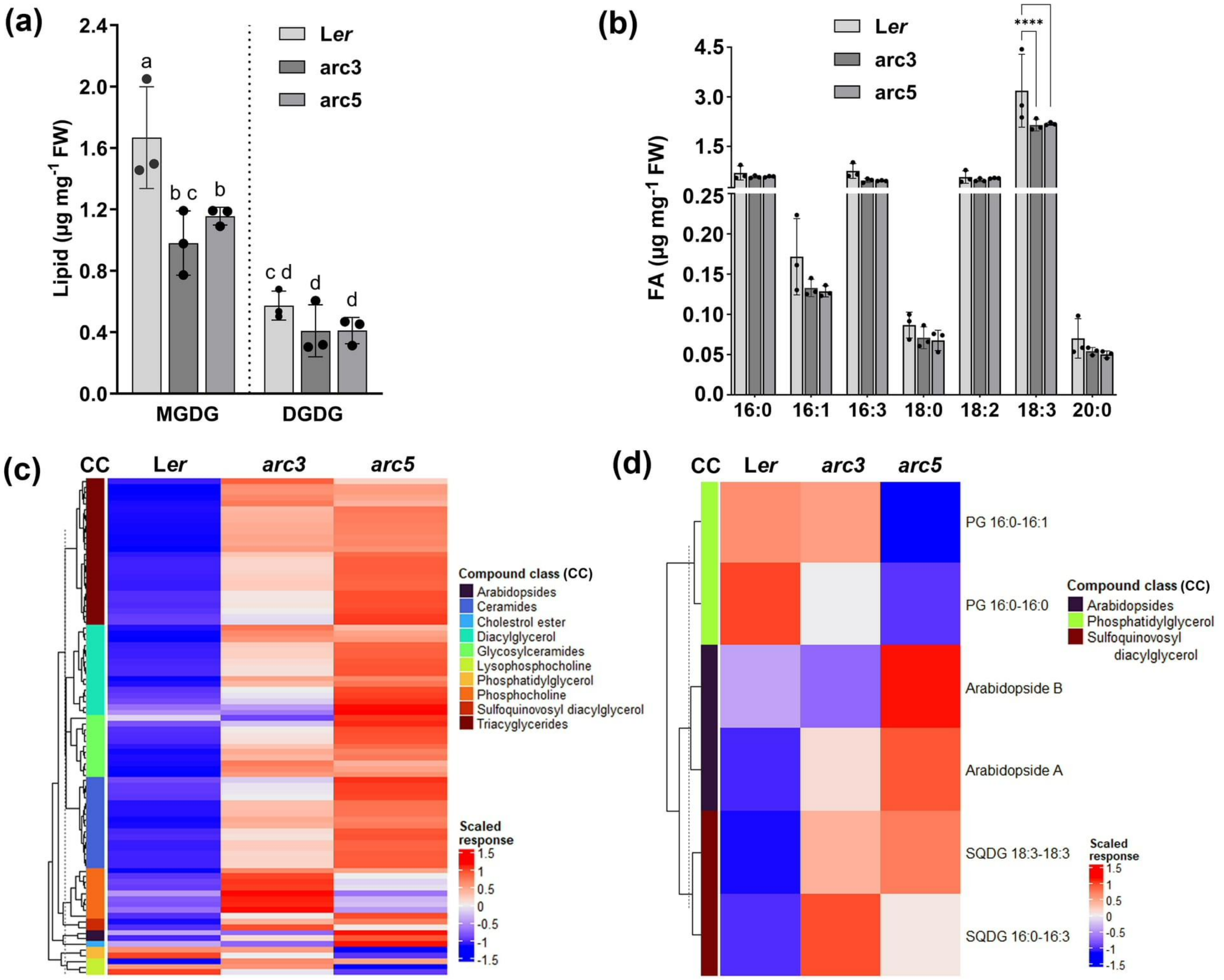
(a)
Ler
arc3
arc5
Lipid (µg mg-1 FW)
a
b c
b
c d
d
d
MGDG
DGDG
(b)
Ler
arc3
arc5
****
FA (µg mg-1 FW)
16:0
16:1
16:3
18:0
18:2
18:3
20:0
(c)
CC
Ler
arc3
arc5
Compound class (CC)
Arabidopsides
Ceramides
Cholestrol ester
Diacylglycerol
Glycosylceramides
Lysophosphocholine
Phosphatidylglycerol
Phosphocholine
Sulfoquinovosyl diacylglycerol
Triacylglycerides
Scaled response
(d)
CC
Ler
arc3
arc5
PG 16:0-16:1
PG 16:0-16:0
Arabidopside B
Arabidopside A
SQDG 18:3-18:3
SQDG 16:0-16:3
Compound class (CC)
Arabidopsides
Phosphatidylglycerol
Sulfoquinovosyl diacylglycerol
Scaled response

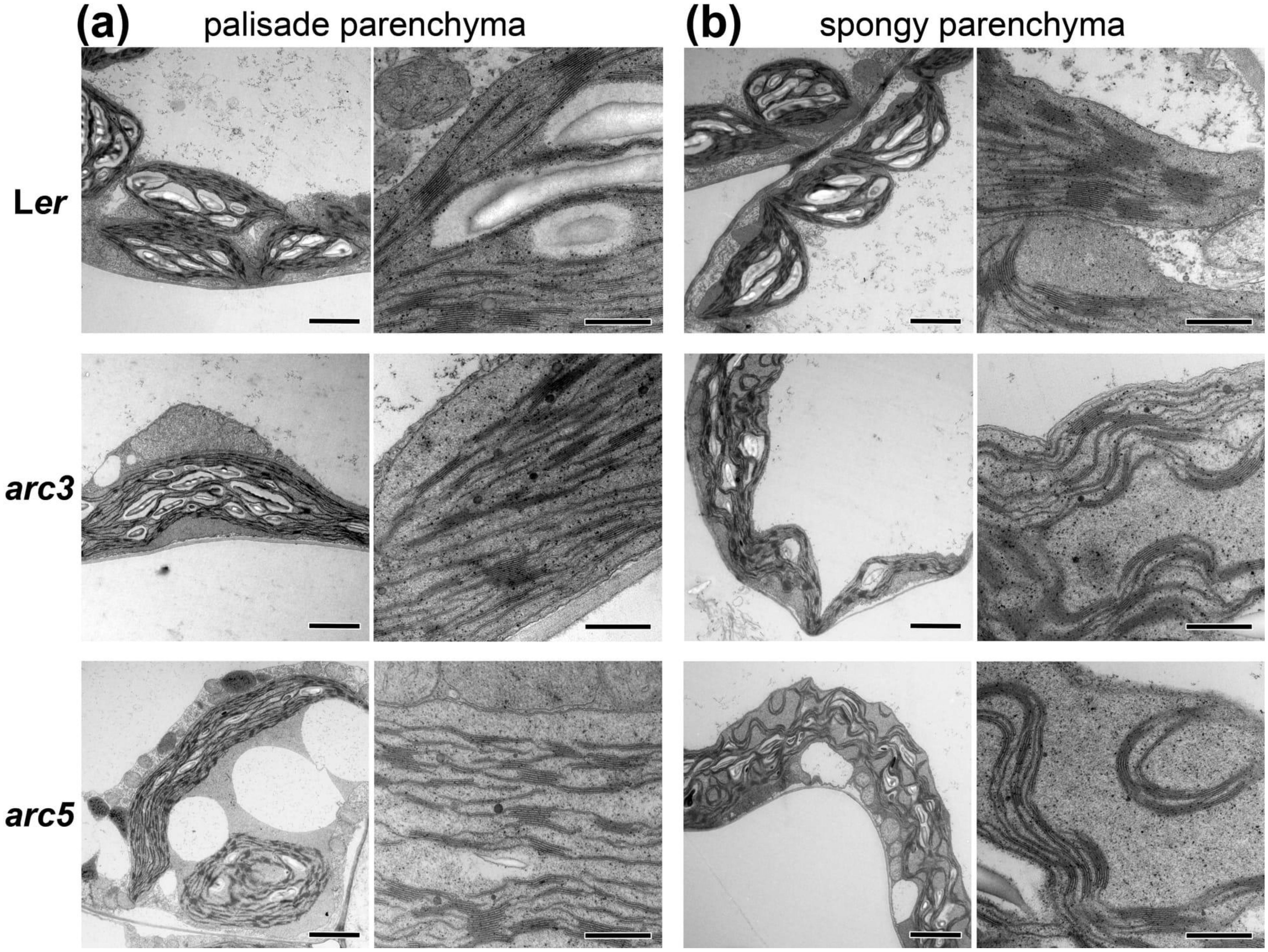
(a)
palisade parenchyma
(b)
spongy parenchyma
Ler
arc3
arc5

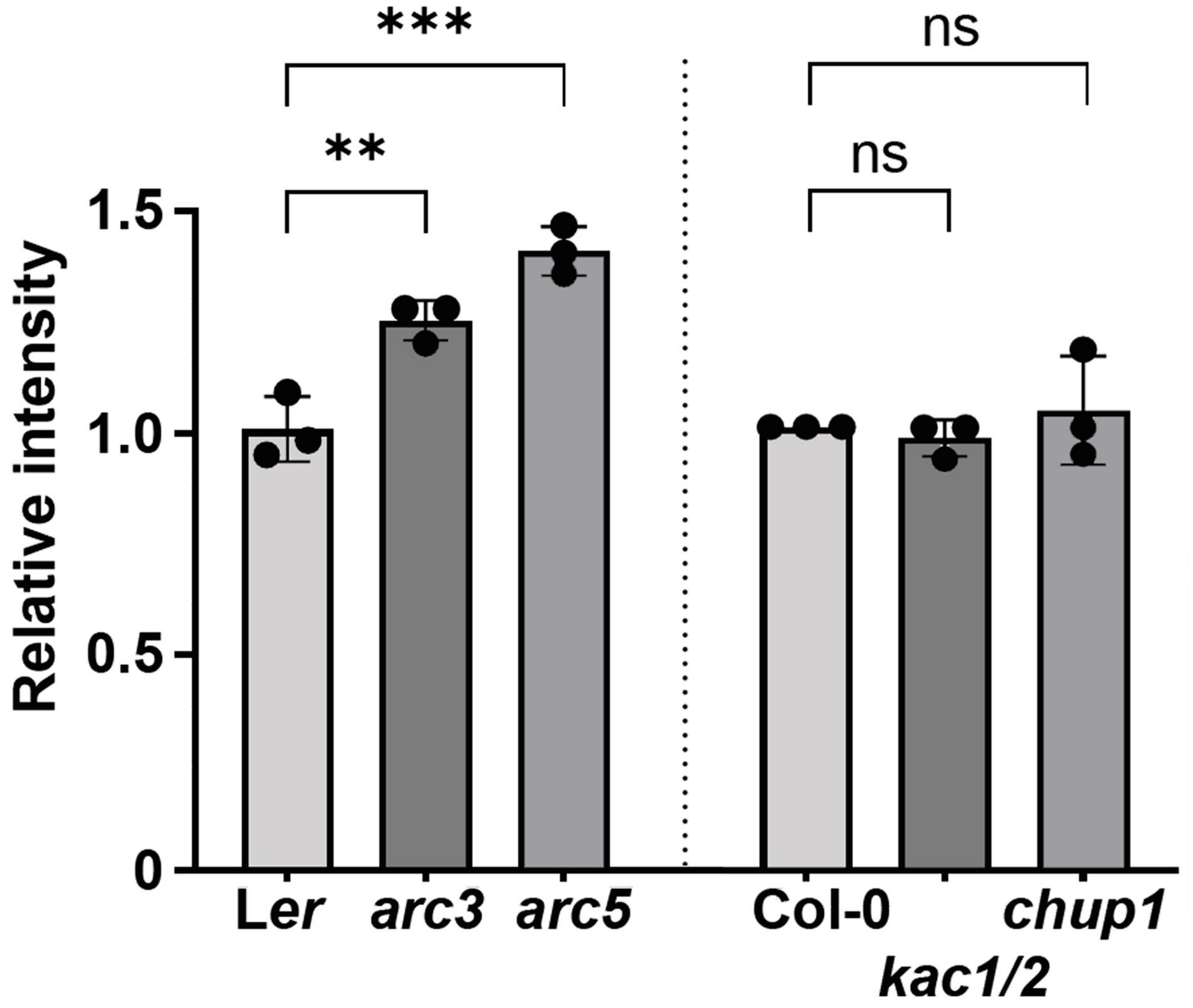
***
**
ns
ns
1.5
1.0
0.5
0
Relative intensity
Ler
arc3
arc5
Col-0
chup1
kac1/2

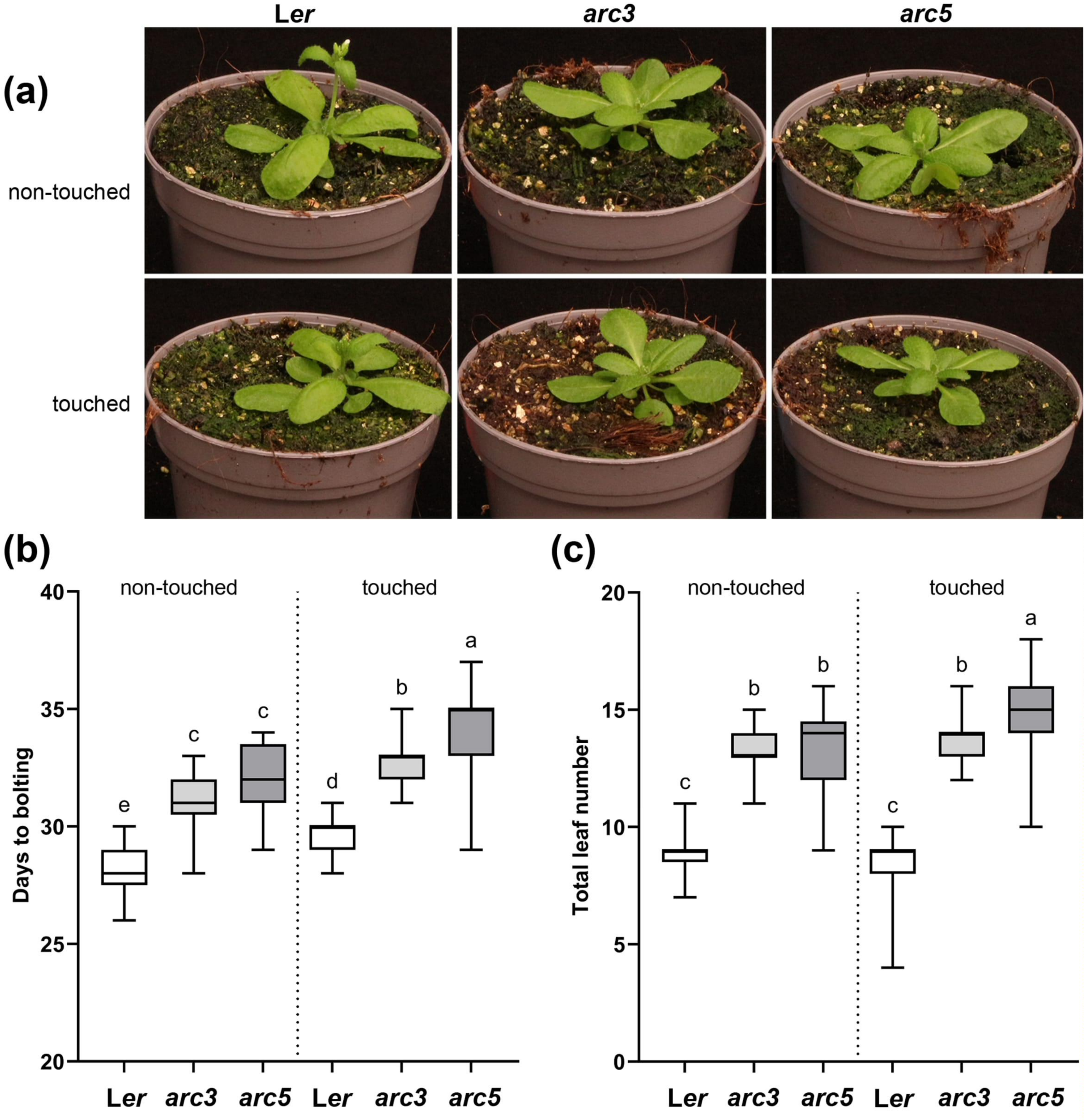
Ler
arc3
arc5
(a)
non-touched
touched
(b)
non-touched
touched
Days to bolting
40
35
30
25
20
e
c
c
d
b
a
Ler arc3 arc5 Ler arc3 arc5
(c)
non-touched
touched
Total leaf number
20
15
10
5
0
c
b
b
c
b
a
Ler arc3 arc5 Ler arc3 arc5

Supplementary figures to:

Size of chloroplasts in Arabidopsis mesophyll cells affects jasmonate biosynthesis

by

Ranjit Baral, Hagen Stellmach, Samuel Ngure Kariithi, Mareike Heilmann, Stephanie Krüger, Joerg Ziegler, Bettina Hause

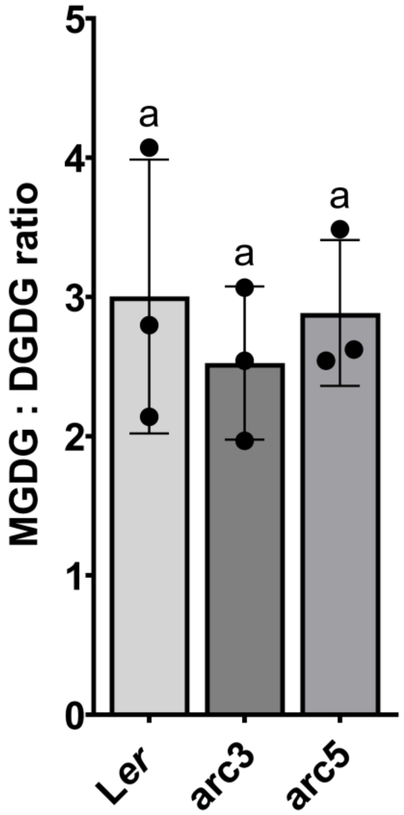

Figure S1. Ratio of MGDG and DGDG in rosette leaves from L*er*, *arc3* and *arc5*.

Ratio of MGDG to DGDG content in wild-type L*er*, *arc3*, and *arc5* plants calculated from total galactolipid quantification. Bars represent means and error bars represent standard deviation (n = 3-4 biological replicates). Different letters denote statistically significant differences according to one-way ANOVA followed by Tukey's multiple comparison test ($p < 0.05$).

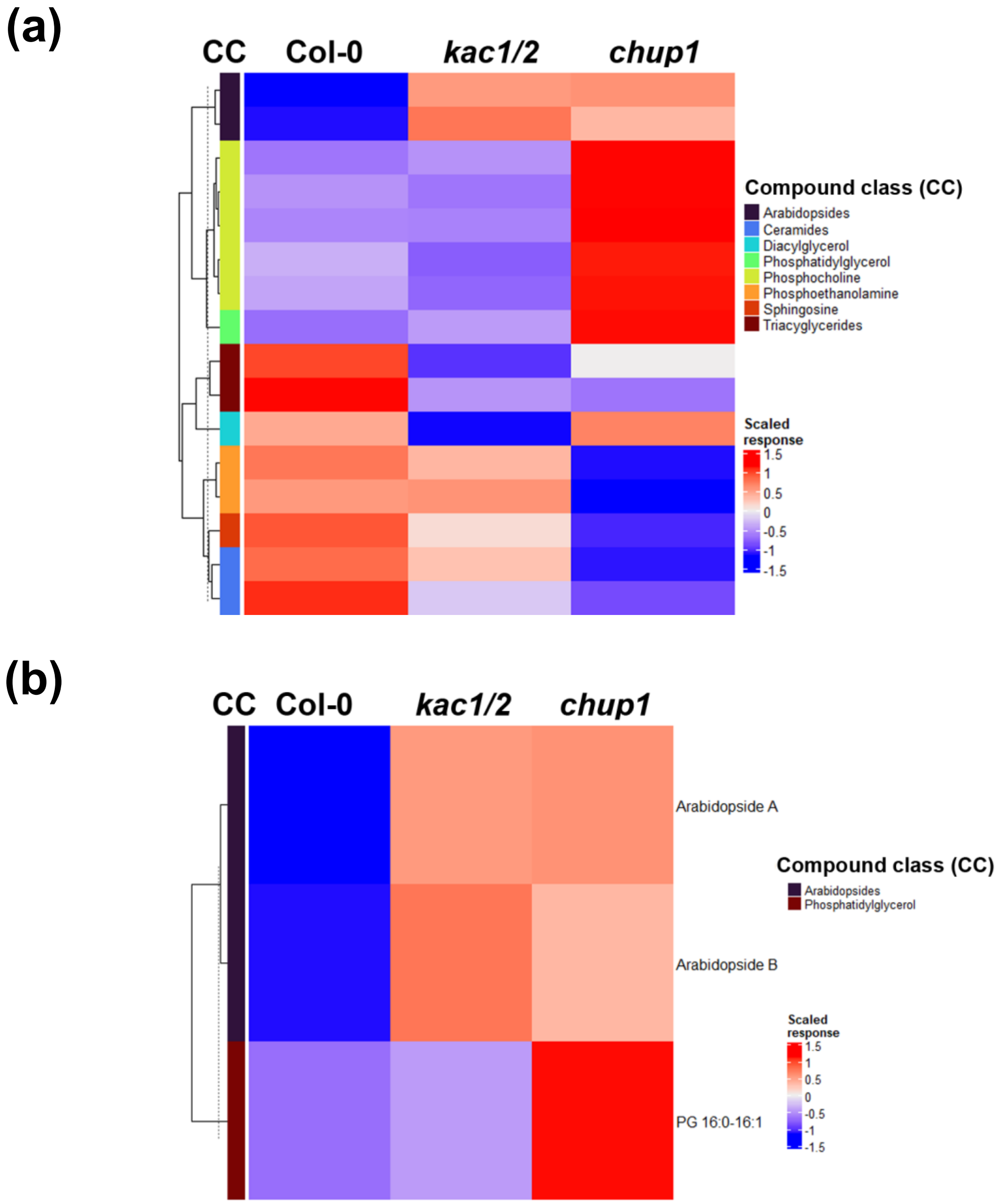

Figure S2. Hierarchical clustering of lipid profiles from *kac1/2*, *chup1* and their wild type Col-0.
(a) Heatmap showing hierarchical clustering of all lipid compound classes (CC) significantly changed across wild-type Col-0 as well as *kac1/2*, and *chup1* mutants (*n* = 5 biological replicates per genotype). Lipid analysis methods as described in Figure 3.
(b) Heatmap showing individual molecular species showing significant changes between wild type and mutants and were annotated with acyl chain compositions (carbon number:number of double bonds).
Compound classes are indicated by color-coded bars in the right margin and are displayed as row-wise z-score normalized abundances as defined in “Scaled response” at the right.

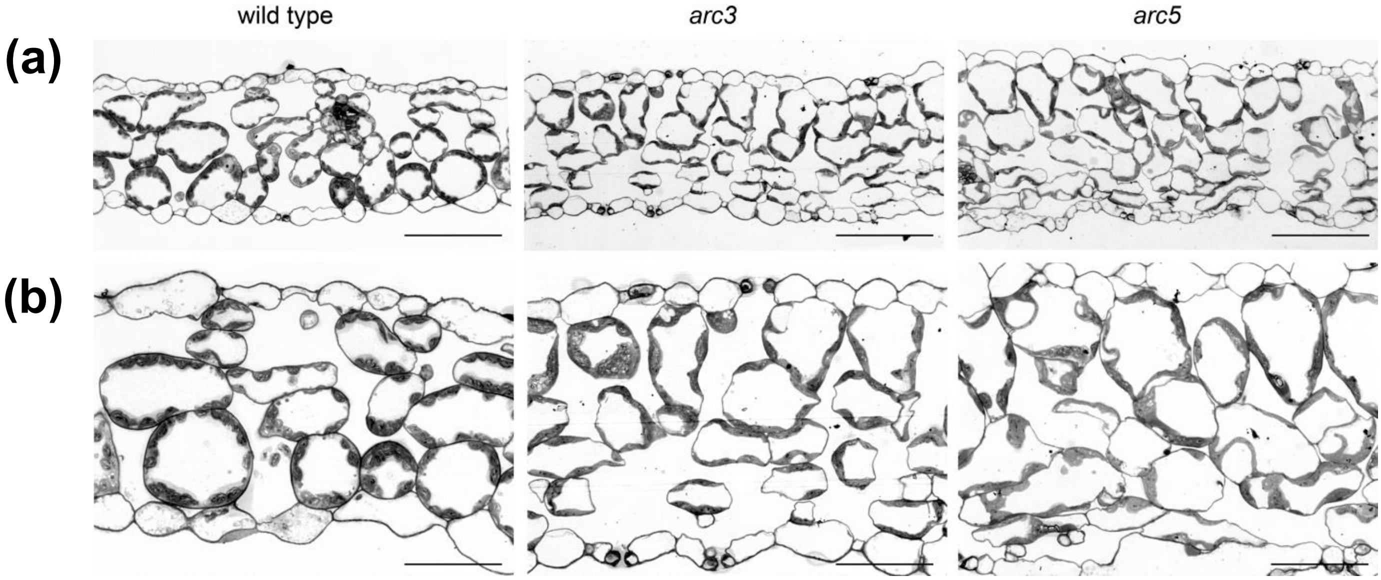

Figure S3. Leaf morphology of L*er* and *arc*-mutants.
Bright field microscopy images of leaf cross-sections showing leaf morphology (a) and chloroplast distribution in mesophyll cells (b) of wild-type L*er*, *arc3*, and *arc5* plants. Note that overall leaf morphology is similar in all three genotypes. Wild-type mesophyll cells contain numerous small, uniformly distributed chloroplasts, whereas *arc* mutant mesophyll cells exhibit reduced chloroplast numbers with enlarged individual plastids. Scale bars indicate 100 μm in (a) and 50 μm in (b).

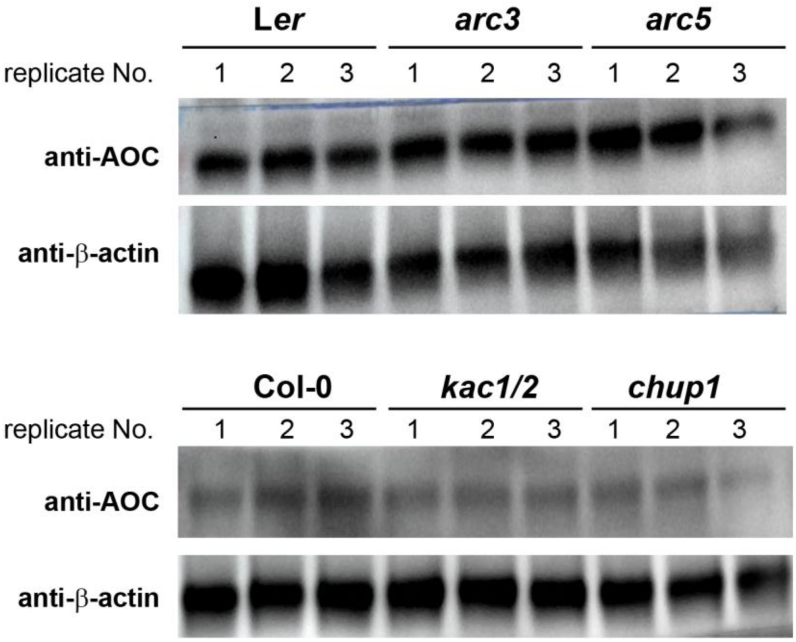

Figure S4. Western blot analysis of AOC protein levels in chloroplast mutants.
Representative Western blots showing AOC protein abundance in L*er*, *arc3*, *arc5* (upper panel) and Col-0, *kac1/2*, *chup1* (lower panel) with three independent biological replicates per genotype. β-actin serves as loading control. Quantification and statistical analysis are presented in Figure 5.

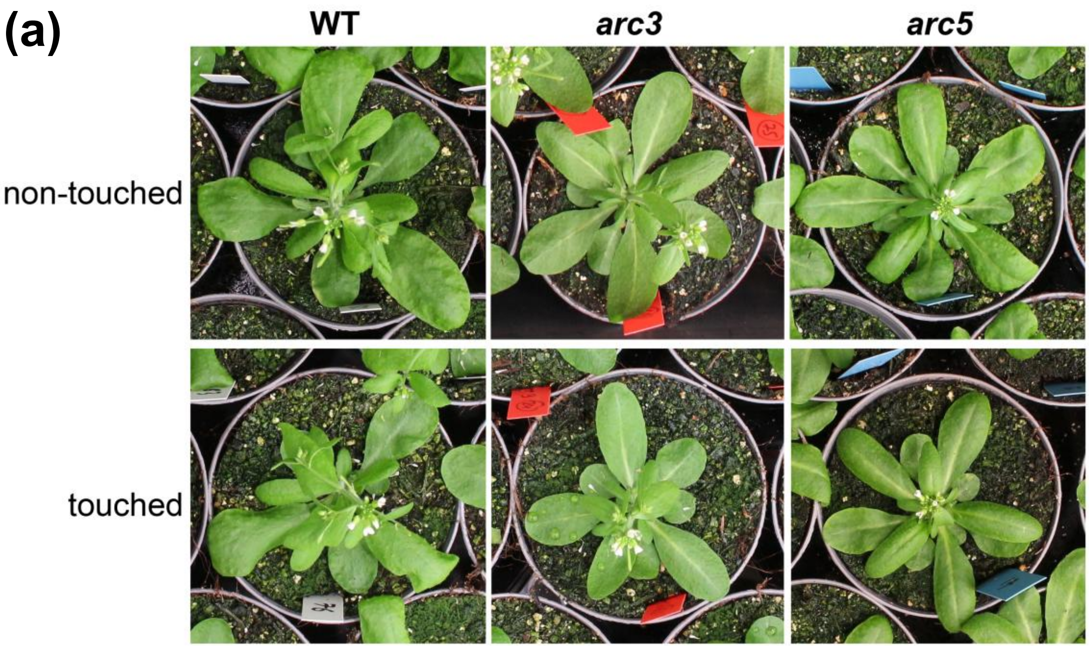

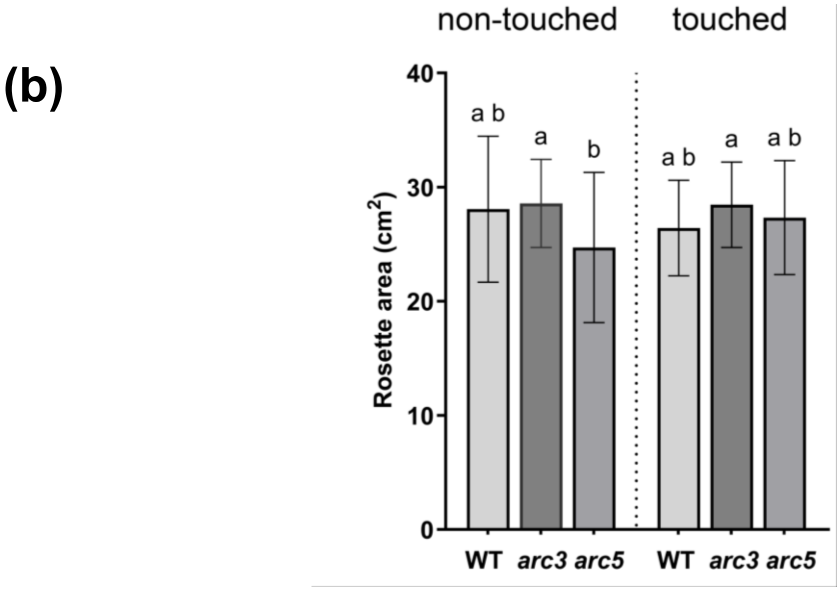

Figure S4. Touch treatment does not alter rosette area

(a) Representative images of wild-type Ler (WT), *arc3*, and *arc5* plants under non-touched (upper panels) and touched (lower panels) conditions at bolting time. Beginning at 14 days after sowing (DAS), rosettes were either left non-touched or gently touched twice daily with a soft brush until bolting.

(b) Bar graphs showing rosette area (cm^2) in non-touched and touched plants (non-touched, n = 33; touched, n = 65). Data are presented as means ± SE. Different letters indicate statistically significant differences ($p < 0.05$) according to one-way ANOVA followed by Tukey's HSD test.

non-targeted lipid analysis

Mass (M)	Name	Compound Class	FORMULA (M)	Ler-R1	Ler-R2	Ler-R3	Ler-R4	Ler-R5	arc3-R1	arc3-R2	arc3-R3	arc3-R4	arc3-R5	arc5-R1	arc5-R2	arc5-R3	arc5-R4	arc5-R5	Col-0-R1	Col-0-R2	Col-0-R3	Col-0-R4	Col-0-R5	kac1/2-R1	kac1/2-R2	kac1/2-R3	kac1/2-R4	kac1/2-R5	chup1-R1	chup1-R2	chup1-R3	chup1-R4	chup1-R5
774.45519	Arabidopside A	Arabidopsides	C43H66O12	187429.65	182704.095	80889.675	221455.575	177291.365	187583.2	272616.315	216479.14	354788.625	231479.225	209479.125	468664.285	271896.955	328266.175	284747.66	265396.445	250642.375	293345.425	261585.075	179598.44	623029.28	473561.72	687975.845	685939.97	736264.815	663534.66	642150.735	743773.69	578007.035	430220.595
802.4869	Arabidopside B	Arabidopsides	C45H70O12	1513.485	8850.205	302.697	5254.38	4168.81	4915.53	6099.43	302.697	302.697	302.697	302.697	302.697	7807.35	10257.345	10293.655	5343.9	4643.93	2779.5	7077.46	1915.01	13361.6	15992.015	23510.965	25991.1	29759.535	12519.635	22261.8	20591.795	14271.65	302.697
537.51165	Cer d18:1h16:0	Ceramides	C34H67NO3	65932.05	112136.98	15360.42	85712.76	112682.29	49988.1	43493.18	51079.12	66137.09	57663.91	78755.8	79961.94	65234.54	94070.59	95400.29	52161.99	59771.05	68647.91	76244.45	43133.02	25443.66	38952.85	60447.23	38726.06	48452.33	26568.36	30261.37	48749.63	37488.58	32550.47
553.50719	Cer d16:0h18:1	Ceramides	C34H67NO4	70063.6475	139473.3	17963.6475	121814.69	148599.815	86196.8475	85210.2975	86964.335	104486.85	105478.5125	104886.7425	122136.9225	98712.1575	125934.25	134485.6275	118501.3	138392.9825	120777.6525	170622.29	141397.845	71182.82	90076.45	144345.55	96300.295	106883.6425	67722.5475	107442.0525	112927.52	91863.575	94758.74
555.52274	Cer d16:0h18:0	Ceramides	C34H69NO4	54310.33667	101524.78	15951.47333	90702.84667	106116.0533	56176.90667	53266.77333	60501.33	64312.14667	64102.49333	93006.20333	82724.14	73732.41333	74802.37	97389.46667	62576.6	95036.74667	94980.89	106409.04	61567.48	35782.39667	40081.95667	64663.91333	40673.72667	45190.96333	22215.56667	36040.57333	41566.12667	32377.39333	41279.64
569.50143	Cer t18:1-16:0	Ceramides	C34H67NO5	64858.25	137481.145	19873.9225	130776.8575	151506.725	125490.975	130863.195	127293.745	150749.8375	141648.97	153494.2325	149160.6975	124278.42	135752.9475	176654.0975	141402.54	135176.4025	124946.3275	161583.0675	173451.98	96895.1075	114653.9825	174776.17	117875.4825	128677.18	90860.1725	133897.9025	147628.4	115548.315	125072.34
571.51723	Cer t18:0h16:0	Ceramides	C34H69NO5	56555.42	94807.82	14384.36	105860.63	101857.73	79275.45	89878.49	85487.13	88556.41	99807.36	113576.65	121816.67	105654.05	89640.89	123703.17	39351.77	57058.14	56181.87	61296.11	48717.67	25751.66	33112.96	71241.5	38948.21	42494.97	23122.36	34892.18	48167.91	29901.63	33675.27
581.53789	Cer d18:0h18:1	Ceramides	C36H71NO4	13313.96	38899.01	4682.55	31985.35	27929.44	302.697	20399.45	18212.81	14819.02	25529.99	41881.8	19399.05	17356.47	26465.6	24845.88	13047.65	16631.05	15879.39	24144.2	19814.95	10131.45	11778.24	21665.73	11743.12	15396.61	6941.54	10796.73	14175.2	16254.6	12654.88
609.56915	Cer d18:1h20:0	Ceramides	C38H75NO4	91757.41	82156.3	35325.43	118303.28	92493.66	78473.17	78998.11	104017.26	74312.27	109650.26	105348.23	116962.63	100822.73	112319.41	92815.88	67440.33	69469.88	109970.77	96682.63	66347.94	54776.49	69401.43	76424.63	85238.44	102883.13	67225.85	74031.62	76663.98	59899.93	50556.85
625.56414	Cer t18:1h20:0	Ceramides	C38H75NO5	25065.595	38594.41	6412.82	38027.255	41520.01	38749.245	40015.62	36370.42	42762.86	39041.975	53689.46	46559.19	40896.64	49216.6	54593.585	46179.8	29484.73	38204.575	40739.995	36915.965	21997.685	30184.48	43992.105	37783.27	29645.9	20493.1	25808.27	37869.34	30494.825	35615.915
651.57974	Cer t18:2h22:0	Ceramides	C40H77NO5	14874.08	34027.05	6195.01	34146.14	46753.64	40762.25	33830.04	34184.77	36121.59	50493.03	53558.56	42389.49	51331.89	50312.52	50382.77	26206.1	33668.79	33636.43	35511.74	43948.89	20084.44	23489.75	36488.75	20668.18	28644.4	19333.76	27535.04	32271.16	24584.19	31471.77
651.65241	Cer d18:0h24:0	Ceramides	C42H85NO3	25076.22	40331.53	6031.13	40018.17	43722.13	28339.35	19410.38	31274.57	35260.75	25746.36	31168.19	32471.13	31780.82	35055.55	38056.33	34258.95	31203.22	27728.79	29414.04	20229.38	19068.79	28806.37	27266.61	28137.53	20392.95	18387.59	18932.52	24577.19	22622.74	24129.85
653.5954	Cer t18:1h22:0	Ceramides	C40H79NO5	365536.3567	583900.6867	104174.8333	555365.0167	606941.95	541458.3167	557297.7967	514386.04	576175.9967	604510.3433	789428.4233	679214.5267	621236.5867	635052.7333	720997.2533	462551.37	455683.6167	527380.5133	525118.57	515154.06	432164.5033	482979.5167	676874.35	452172.2233	483422.7767	334643.7467	472641.56	501923.1833	423515.3133	433137.9033
655.61099	Cer t18:0h22:0	Ceramides	C40H81NO5	72441.665	157296.72	15341.08	118319.83	171519.69	109322.945	106733.825	129339.14	144367.245	138351.92	205173.955	164664.375	156453.515	199598.735	207908	77080.865	72316.94	89782.92	106398.265	82243.155	62073.515	52385.67	88396.99	56242.805	66528.41	26573.29	46514.595	54237.12	44394.325	43929.33
663.61587	Cer d18:2h24:0	Ceramides	C42H81NO4	513643.78	841555.25	183217.83	856998.44	895940.25	480273.72	551700.19	551237	587503.81	649400.19	778977.31	750142.06	616588.25	806586.44	772850.56	491398.28	579566.81	446371.13	525657.81	521054.06	402060.5	399147.31	537799.38	456885.5	487570.97	362144.16	467428.91	533476.63	476209.34	469103.53
665.59501	Cer t18:2h23:0	Ceramides	C41H79NO5	102813	126044.27	41994.12	133879.42	137266.94	141583.02	143457.38	147296.02	145098.23	138176.11	152959.2	186806.09	189004.13	183523.66	156840.61	114670.68	139508.73	127965.66	128913.57	134120.22	109634.32	98648.22	155080.77	129933.78	141311.45	111961.28	145290.53	148522.19	115983.87	113288.17
665.63156	Cer d18:1h24:0	Ceramides	C42H83NO4	476649.4867	977196.5933	122783.31	831707.0367	1034630.683	623627.7133	548049.32	643457.07	704915.02	692403.3767	846085.6667	725049.7733	611364.0033	800547.7	897361.31	823512.24	670977.97	552123.3067	694165.4167	781229.59	437325.71	535152.8133	859601.3	585395.9567	588251.0533	363877.66	604788.7567	672241.2433	556128.5767	595895.23
667.61087	Cer t18:1h23:0	Ceramides	C41H81NO5	139811.61	300468.75	27536.72	225876.36	269908.28	196735.06	215943.36	224358.53	296373.63	257015.8	410177.97	315422.16	295010.75	357097.63	386567.06	230517	199267.19	205945.98	246160	263168.41	193128.23	168660.58	288127.97	205143.75	203574.27	112703.79	165459.13	204666.52	161386.84	164781.77
669.62645	Cer t18:0h23:0	Ceramides	C41H83NO5	21326.15	46906.14	302.697	30327.06	82085.17	39426.1	38905.99	38160.46	42646.93	33650.32	51326.59	34673.84	39574.91	34556.19	58790.36	38787.65	26271.38	39954.12	38327.5	29958.6	24874.32	25381.94	28747.4	29542.33	302.697	16635.13	31975.64	34878.19	30081.51	27602.89
675.57964	Cer t18:1h24:3	Ceramides	C42H77NO5	5692.29	9483.73	302.697	10352.15	12050.98	11858.43	8842.66	10424.58	12027.54	11058.05	17505.96	9166.01	9169.47	6389.05	9517.29	12646.06	12339.38	10433.82	14605.12	19594.21	6525.35	9292.13	15681.69	11605.9	10632.22	8795.49	14693.92	18728.85	13396.55	14091.93
677.63128	Cer d18:2h25:0	Ceramides	C43H83NO4	42386.9	97932.39	15273.79	93674.57	101729.56	73129.18	72185.64	76504.8	68459.41	80378.06	112489.7	106568.07	94109.06	99125.2	110080.52	73043.96	95343.35	67108.93	90739.86	79468.2	54642.64	54668.1	73667.44	64068.45	71312.13	42859.83	68711.09	64003.44	58747.14	54601.76
679.6113	Cer t18.2h24.0	Ceramides	C42H81NO5	1147266.713	2035868.61	315094.79	2012152.24	2177311.633	1924430.757	1951374.87	2068764.687	2033641.907	2048499.25	2386789.543	2200369.75	1914137.11	2019527.637	2426829.907	1565637.523	1458706.443	1287653.363	2098027.377	2145586.75	1298453.803	1433084.187	2187839.3	1400693.15	1602610.233	1060061.827	1541755.53	1766003.527	1479772.25	1628088.563
681.62716	Cer t18:1h24:0	Ceramides	C42H83NO5	562113.964	1080918.58	138178.958	1002191.02	1180436.102	909028.814	894084.264	953911.306	1113382.918	1005880.388	1188006.028	1048628.562	984114.424	1061507.542	1249536.188	998219.348	828945.46	821687.156	996483.662	1085695.21	707878	820433.11	1400261.11	854560.726	888892.712	567225.626	857727.186	996052.594	808446.466	808004.304
683.64133	Cer t18:0h24:0	Ceramides	C42H85NO5	101584.695	254519.66	30009.5	222776.115	412636.45	182530.3	179171.64	203451.895	246365.995	224445.02	292714.55	238285.69	211383.12	221757.715	297871.555	136422.44	132809.955	156095.07	201199.34	147939.995	104397.795	112345.63	201870.325	121382.8	116074.08	66567.995	130120.055	149780.12	111363.105	108115.355
691.64709	Cer d18:2h26:0	Ceramides	C44H85NO4	167800.92	404357.59	39874.98	286602.28	342906.09	226700.14	231984.05	247842.64	264865.63	298902.88	257535.7	254240.58	224913.92	284424.69	301955.97	315261.59	234736.8	223778.84	283217.31	287370.78	164176.84	208833.13	305721.72	217045.75	294320.16	160316.14	250689.98	288782.81	206608.86	229488.95
693.62651	Cer t18:1h25:1	Ceramides	C43H83NO5	244108.67	358398.5	91703.06	327832.53	368709.28	318697.88	392234.75	353571.34	391780.66	390946.34	401802.09	406946.97	385494.75	365407.19	398427.34	240071.94	248338.31	255659.77	300434.59	302263.88	224980.28	296685.91	359542.19	276524.41	267416.38	206313.84	268934.22	293822.41	249956.66	252464.05
693.6637	Cer d18:1h26:0	Ceramides	C44H87NO4	187063.8133	379998	62417.73333	387618.91	505551.2267	274378.0033	280824.87	323829.66	399502.3167	323277.91	332819.06	308502.3867	234550.77	290348.25	381575.6567	405379.94	351686.11	291099.7333	388328.97	348236.3733	224869.1333	239731.7067	558472.18	329134.9733	268568.8567	200489.5867	376059.9433	404213.4	330516.97	283342.8367
695.64215	Cer t18:1h25:0	Ceramides	C43H85NO5	43435.915	101326.015	11554.245	83238.45	104125.515	82634.925	77581.08	89439.65	110927.145	88211.75	132811.155	94629.02	82670.53	104274.74	121380.965	87324.67	80035.025	58043.1	91959.03	86664.445	56560.81	65899.745	113250.595	70277.095	73926.525	37872.545	69095.5	84867.245	71019.86	66729.46
695.67857	Cer d18:0h26:0	Ceramides	C44H89NO4	370032.41	729640.94	105230.01	653428.63	977204.13	419307.88	402038.09	523789.78	669362.13	503316	603580.13	602878.63	422781.25	523262.81	697328.88	398841.53	378850	353461.22	304715.75	316172.75	202203.28	237518.42	458865.13	289361.56	223360.11	168189.09	292112.59	303325.44	269072.31	242955.31
697.65737	Cer t18:0h25:0	Ceramides	C43H87NO5	16185.42	37064.66	4513.37	38180.36	90386.53	23884.53	25236.17	30698.32	40742.76	37530.47	29418.42	35105.34	33922.55	27276.57	42596.2	27201.39	32360.04	34052.3	40317.46	18616.02	20746.93	25669.73	30067.53	26139.8	21226.28	16033	31836.34	29001.1	32047.6	19604.17
707.64213	Cer t18:1h26:1	Ceramides	C44H85NO5	275827.91	674457.19	81392.39	555191.31	682862.75	562041.31	605109.13	666535.75	723675	774400.94	789313.25	638032.19	581366.5	671957.69	819194.81	546296.25	500040.47	467765.44	658268.94	727116.19	376181.13	421955.44	761116.38	505277.34	521167.91	327306.41	515035.22	603620.5	482210.19	468221.56
709.65925	Cer t18:1h26:0	Glycosylceramides	C44H87NO5	218024.63	444137.96	55074.17333	459007.7767	586332.8667	447932.32	456115.6033	522083.29	620928.4433	509002.4267	594158.8033	518643.8033	406828.9867	434848.9233	583048.4967	439141.4967	405147.6167	347683.6733	422895.6267	438468.04	293768.9333	336784.2667	606516.18	366411.6967	337029	221327.31	390209.7333	429321.52	363718.8733	322404.0233
711.67423	Cer t18:0h26:0	Ceramides	C44H89NO5	66980.09	142165.16	21295.46	156503.44	258845.13	112514.87	128134.68	150367.58	199494.97	143038.92	221771.73	196691.94	142528.84	146741.5	242510.19	78130.66	72727.2	61762.4	79702.17	69412.95	55121.27	50743.92	97375.89	66642.88	55369.35	36329.35	56543.74	70558.56	61998.77	56455.63
646.56836	CE 18:3	Cholestrol ester	C45H74O2	11158.51	19295.25	3083.18	19721.75	24664.46	11835.76	10103.94	8539.87	9725.18	13084.42	22171.5	19052.21	16966.44	21278.33	24766.1	17405.41	18570.44	14341.71	17748.56	14621.22	15299.34	14953.66	19888.09	16729.48	15975.1	11272.38	10379.56	15047.1	10270.66	12421.42
648.58405	CE 18:2	Cholestrol ester	C45H76O2	21771.27	21529.14	2467.2	17290.87	20319.66	16414.74	14260.12	14415.08	18196.63	14892.86	15566.36	19982.2	13776.53	21755.98	20648.5	31718.79	23018.55	20392.2	17254.58	14145.97	11939.75	17824.05	19579.99	21548.3	15122.65	14486.43	8785.23	15378.77	16677.48	16411.13
650.59942	CE 18:1	Cholestrol ester	C45H78O2	27857.13	18290.16	3459.44	17291.84	15821.76	19050.65	20007.86	19991.04	18599.3	15280.12	11540.45	17058.78	14800.29	25610.16	20125.51	30710	21979.21	18399.38	15626.1	12041.42	14939.28	19725.7	16549.08	27160.46	17195.23	16533.26	9113.81	21183.37	16038.51	21224.61
536.44391	DG 14:1-16:1	Diacylglycerol	C33H60O5	13549.08	302.697	302.697	302.697	302.697	302.697	10141.56	8721.18	302.697	302.697	302.697	302.697	302.697	302.697	302.697	17157.04	10547.06	6650.97	8600.29	302.697	6206.83	302.697	302.697	13263.58	7199.35	302.697	302.697	8281.82	7093.69	302.697
538.45928	DG 14:0-16:1	Diacylglycerol	C33H62O5	19064.42	13313.77	2232.24	17860.38	7688.85	302.697	26174.09	16410.33	15893.04	15873.37	10498.49	8102	15026.72	20531.26	20719.98	27569.65	22848.17	19417.08	20693.6	13641.68	14240.82	14826.12	14256.03	24824.55	15453.03	8027.89	8396.44	16967.94	15357.3	14317.42
540.47521	DG 14:0-16:0	Diacylglycerol	C33H64O5	21141.97	21602.38	4384.87	19621.48	14229.88	19336.21	26445.13	21621.67	30395.02	20004.82	16110.4	20328.7	19058.92	29840.01	27596.03	34627.44	24081.1	23038.61	32505.63	22005.98	15342.49	22172.77	21732.68	30563	20002.95	13172.82	11470.07	24010.18	20272.79	23281.63
552.47457	DG 15:0-16:1	Diacylglycerol	C34H64O5	24366.09	302.697	2270.44	15368.03	302.697	302.697	22152.28	15587.4	19093.63	13715.62	302.697	16023.85	302.697	21458.79	302.697	31289.04	17118.66	21329.86	20991.39	302.697	17549.39	23075.31	302.697	26418.62	19675.08	302.697	302.697	20079.95	18131.07	302.697
554.49037	DG 15:0-16:0	Diacylglycerol	C34H66O5	18157.12	18458.71	302.697	18007.86	13474.18	15770.81	21842.25	18457.42	15708.48	16710.73	12275.53	14316.21	16235.9	21594.66	18263.46	24417.63	20579.14	14043.96	20142.81	15715.69	9750.57	16373.66	12980.35	22171.93	14185.23	9154.83	10166.75	20223.35	18961.95	14548.81
562.45964	DG 16:0-16:3	Diacylglycerol	C35H62O5	9890.56	22108.72	302.697	18311.13	18747.12	18980.55	17449.49	19357.45	24412.04	15161.98	18390.05	26342.88	17368.69	25390.59	31595.98	26420.17	34379.26	25201.26	36043.21	29933.16	13354.49	18496.11	24427.46	23067.03	21951.72	28081.47	34638.23	31604.3	23156.54	31290.37
564.47524	DG 16:1-16:1	Diacylglycerol	C35H64O5	25436.56	15229.93	3389.19	23579.03	14213.05	18369.96	28303.72	19597.32	15902.04	16350.25	18186.37	16427.08	21014.85	20810.77	33125.82	35374.54	26170.19	19429.18	31826.18	19459.29	16995.88	18408.42	20533.9	29858.82	17026.81	15081.8	7396.73	24761.07	21276.23	20732.51
566.49079	DG 16:0-16:1	Diacylglycerol	C35H66O5	71493.73	47763.14	8805.43	55413.26	43813.99	50010.61	72883.81	61985.12	67169.83	50898.09	34128.85	56624.09	58023.09	76099.81	77233.45	112440.05	67742.3	67709.6	72378.44	53049.77	52780.25	68944.55	54592.58	96021.69	49461.57	30860.63	34347.06	67939.35	65764.83	60311.57
568.50603	DG 16:0-16:0	Diacylglycerol	C35H68O5	105442.09	98420.95	20928.34	89511.45	110176.5	80485.01	114544.57	81110.54	176377.95	85819.15	129906.95	93099.5	113697.02	120041.34	110688.87	139281.97	108219.79	84621.73	187913.86	128033.88	79554.9	89223.07	145839.19	112402.8	89979.68	66739.76	66442.24	143017.19	137769.11	151976.56
582.52147	DG 16:0-17:0	Diacylglycerol	C36H70O5	17050.24	302.697	3999.51	302.697	302.697	11215.14	26695.79	12742.68	19409.74	302.697	302.697	302.697	302.697	302.697	302.697	29647.13	302.697	302.697	302.697	302.697	302.697	14412.79	302.697	19662.2	302.697	9168.85	302.697	20768.3	18635.95	13499.29

584.44385	DG 16:3-18:3	Diacylglycerol	C37H60O5	24933.73	39638.02	13547.77	51969.26	69793.84	44663.77	35563.79	32352.02	56468.56	37593.37	51569.46	41789.55	42033.64	44615.6	63543.7	39491.9	53762.52	30832.15	66806.55	49988.64	18964.87	18327.41	31158.51	25266.35	19971.76	22303.49	38781.38	40934.64	46516.45	79117.34
586.45927	DG 16:3-18:2	Diacylglycerol	C37H62O5	5600.41	13504.51	1828.77	10785.82	20664.01	13135.66	12246.27	12769.04	16929.93	12137.57	18604.8	14637.93	19643.41	16895.33	29993.88	21762.24	19127.42	15818.37	30336.6	23975.03	12302.35	12339.55	18747.25	12956.64	15729.26	11072.43	19762.13	22923.13	17290.5	25283.86
588.47507	DG 16:1-18:3	Diacylglycerol	C37H64O5	25200.325	52270.315	8948.3	44586.765	60980.83	43981.64	43384.36	36573.535	53089.425	45658.745	58838.915	67082.22	62712.69	77346.51	117965.53	63727.795	74164.22	60188.03	93643.45	85614.025	34192.77	48425.485	96992.555	56847.135	62537.095	40737.775	85180.89	69545.83	54992.765	70516.34
590.49035	DG 16:1-18:2	Diacylglycerol	C37H66O5	23299.33	17183.22	302.697	21885.54	23004.55	14343.06	16037.8	18508.74	14762.79	15623.64	27990.98	18239.67	30858.14	16516.76	17713.99	27787.27	31186.54	30607.75	46578.83	24319	14767.57	15910.83	28368.94	25877.91	27237.13	21948.25	21591.76	46781.68	35884.83	31961.62
590.49043	DG 16:0-18:3	Diacylglycerol	C37H66O5	611419.19	1077951.13	213588.84	1207965.13	1385074.75	1122728.13	1186565.25	1175864.63	1443898.63	1242184	1424852.63	1438969	1278304.63	1475547.13	1902909.5	1601547.13	1813703.13	1480032.88	2329699.25	1963293.5	952651.19	1171796	1858143.38	1467334	1403248.13	1371467.63	1676798.13	1973980.25	1460071	2049419
592.50625	DG 16:1-18:1	Diacylglycerol	C37H68O5	736928.88	1238878.38	241055.13	1331356.88	1467591.88	1209578.75	1311646.88	1290990.75	1486750.63	1284217.25	1551303.75	1680848.38	1400186	1629802.38	2105349.75	1668352	1807276.88	1480155.38	2128755	1861465.88	914763.06	1108980.5	1732216.75	1358173.38	1357556.13	1249569.25	1689047.88	2096969.38	1637915.75	1768697.5
592.50635	DG 16:0-18:2	Diacylglycerol	C37H68O5	525039.75	577582.94	283248.09	697764.5	659837.75	656989.38	767906.19	752247.06	825965.25	778087.81	666243.25	678298.38	562693.5	604301.25	711179.38	624459.69	629400.44	584168.94	603148.81	533814.75	452921.53	468291.44	598768.75	592517.69	624860.94	674071.94	688879.25	823975.5	721396.88	622742.19
594.52194	DG 16:0-18:1	Diacylglycerol	C37H70O5	148302.44	163256.86	35204.29	171363.89	179781.09	126498.24	155227.39	160643.77	227828.83	222895.55	131749.19	176593.02	166894.75	167448.39	207950.83	254005.55	227185.84	190587.23	307744.34	228729.28	137084.64	170780.63	312951.47	226241.09	143560.23	132098.45	153870.66	285222.69	282559.34	277694.72
596.53746	DG 16:0-18:0	Diacylglycerol	C37H72O5	165409.66	193632.11	23000.7	159867.73	164336.08	194202.53	151401.55	138302.33	178610.59	160759.05	154801.09	151727.72	120272.47	135490.14	139940.2	198532.58	258211.17	211068.28	231926.11	189973.06	169537.38	175332.2	196470.88	194259.94	249242.33	176884.88	224985.92	193723.33	164170.63	177770.5
604.50606	DG 17:0-18:3	Diacylglycerol	C38H68O5	7794.33	11411.93	2232.6	11488.75	10436.58	9998.5	11229.31	10925.29	12591.54	11070.16	12793.09	16675.76	11759.84	11482.48	17164.11	16118.61	18845.87	14792.83	23332.8	18819.71	6651.21	9564.44	17777.36	14887.81	12285.06	9776.3	13668.88	18943	14830.51	19164.78
612.47497	DG 18:3-18:3	Diacylglycerol	C39H64O5	315437.72	382661.88	143929.55	480158.31	497956.47	529257	450099	501670.41	566235.75	475802.63	540718.81	514641.72	509346.91	468121.5	594137.81	500497.5	605378.81	510144.47	800535.81	552924.94	372815.75	329562.41	471587.59	429547.75	459133.47	429646.31	555857.19	594699.5	459400.31	582874.5
614.49064	DG 18:2-18:3	Diacylglycerol	C39H66O5	143439.985	256664.69	46296.995	306138.275	342419.945	256604.26	257607.575	273546.655	366781.485	294108.28	371128.215	339223.98	295780.045	360668.76	506875.125	359292.02	381812.4	281144.625	461383.69	399100.75	171466.97	186396.5	362394.485	253171.335	257415.9	245529.5	382562.68	402269.57	300771.63	345731.53
616.50625	DG 18:2-18:2	Diacylglycerol	C39H68O5	193319.97	216205.5	133502.86	238426.69	282869.44	205325.95	247800.3	249582.39	253751.23	245606.88	216174.84	279662.97	277045.91	273162.53	322163.88	211706.39	223698.03	206010.09	311754.5	222714.27	179083.67	150251.41	272664	162269.64	203306.27	192113.02	199639	247212.91	169551.81	244940.53
618.52191	DG 18:1-18:2	Diacylglycerol	C39H70O5	79428.445	128400.63	26979.895	130702.985	154910.93	93092.485	102346.22	104137.265	148436.37	115171.015	127227.625	131251.69	113562.885	141764.79	173739.695	133593.365	145752.265	112763.525	191571.93	167068.39	78526.715	87711.185	215069.815	117793.285	102593.33	80987.775	121107.345	161944.135	124050.395	131260.53
620.53744	DG 18:1-18:1	Diacylglycerol	C39H72O5	83261.76	63988.86	22119.25	57799.78	90390.06	60203.64	99651.57	110450.34	76994.83	134673.525	62595.955	61981.185	88769.16	44411.165	74506.75	113638.035	58894.85	54031.45	162264.62	125524.205	43031.215	48252.965	139850.73	62171.975	76510.51	54464.795	48922.1	191438.525	144801.77	177303.72
622.55259	DG 18:0-18:1	Diacylglycerol	C39H74O5	32013.69	32348.79	6179.83	28250.22	29260.33	28744.74	28640.63	30014.77	44669.49	60170.69	27252.92	35520.27	26330.01	38635.98	43687.35	46884.19	40512.23	30326.09	65969.89	58161.47	23274.48	33024.67	52077.25	37338.07	26588.64	23247.49	26489.4	64941.11	55606.52	60905.21
624.5691	DG 18:0-18:0	Diacylglycerol	C39H76O5	106307.5767	118808.36	11334.84333	98115.59333	51576.92333	149173.56	89333.85333	78869.33	46902.73333	85897.42333	38356.51	78494.08333	34672.93333	40020.71	38029.99667	125426.5633	188427.0233	178649.6333	78682.92333	59109.04667	120751.1967	103423.1667	52075.06333	121631.3233	171880.2867	106113.55	132977.21	60360.14667	44979.93667	47128.15
652.59915	DG 14:0-24:0	Diacylglycerol	C41H80O5	18077.8	20089.06	4375.82	17221.15	13360.96	21179.76	15922.3	15523.03	15530.13	18740.94	11457.66	15451.63	14163.99	15306.03	18709.75	29131.7	31926.05	29517.17	20790.59	18145.03	16931.57	18579.26	15584.45	24541.4	28190.42	14793.23	18359.24	16343.63	14544.45	15934.57
668.53968	DG 16:0-24:6	Diacylglycerol	C43H72O5	20422.49	31017.72	302.697	21355.05	60869.31	18351.92	6166.01	6363.38	61016.07	14734.98	75864.16	18384.68	71155.65	84452.04	84342.97	39453.11	32694.44	10970.48	114362.88	110417.93	23052.34	19621.18	94568.48	26125.85	31154.71	25592.02	18682.29	89548.45	93706.27	120178.07
680.63113	DG 16:0-24:0	Diacylglycerol	C43H84O5	18401.68	21296.995	6488.285	23908.125	17362.275	19350.97	18288.68	20904.425	22291.545	18430.975	17060.635	18174.57	12529	15953.66	18979.835	29177.725	24694.75	23228.82	20460.065	16850.485	16218.465	18860.435	17516.885	24129.525	19733.205	17126.535	17000.415	22398.98	19578.115	17390.135
694.64628	DG 16:0-25:0	Diacylglycerol	C44H86O5	10108.82	12158.21	3761.02	13104.76	10211.89	10427.15	11712.84	11137.62	13181.65	13296.11	9758.3	11090.41	11623.89	13054.8	12999.08	20737.73	13887.62	12004.23	13372.67	11032.75	9575.02	10495.57	9857.03	15842.37	13126.47	8047.64	7442.73	14158.01	13473.9	12269.17
704.63117	DG 18:2-24:0	Diacylglycerol	C45H84O5	8633.5	15430.85	302.697	14744.29	20237.82	14458.66	13152.97	13732.25	16649.89	17495.32	17112.63	17457.31	17525.04	16897.86	24728.94	25052.52	28945.06	23354.33	35684.18	27691.26	15022.41	16872.52	32008.15	20512.96	19577.22	13439.84	21822.7	21014.39	20174.62	22204.07
724.60225	DG 18:0-26:6	Diacylglycerol	C47H80O5	55631.55	62892.25	302.697	40287.09	76170.23	58516.29	17455.9	15415.78	94320.04	42181.89	119939.85	27654.9	104487.42	106622.41	107126.48	69521.55	79844.01	47643.13	140867.56	141106.27	59115.52	48165.61	114770.39	59758.87	81987.55	51185.48	58679.38	124921.19	135666.7	138433.14
699.56413	Glc-O-t18:1h16:0	Glycosylceramides	C40H77NO8	19990.99	39401.89	7010.47	36910.3	48341.43	28530.99	27967.28	25865.42	33068.95	28780.81	40772.39	36717.52	25842.07	51504.4	43186.35	34910.23	32935.63	35505.38	45261.09	37993.53	22440.98	23665.1	41970.59	28227.66	25886.68	24910.57	30075.75	35136.71	27370.82	28240.91
715.55953	Glc d18:1h16:0	Glycosylceramides	C40H77NO9	107462.0689	164900.0767	48377.84222	158307.32	163840.6389	134350.1589	135670.8711	135524.1778	155129.5189	149598.46	155083.0656	163414.0933	135623.4122	144605.3878	174973.8456	149654.45	157844.0544	155830.4456	168278.4989	156854.8244	143832.8544	138664.9622	179013.7989	153362.2689	152386.98	139226.2389	153894.8567	153284.9311	140837.7033	151183.3733
731.55404	Glc t18:1h16:0	Glycosylceramides	C40H77NO10	146181.09	226508.45	38627.32	218976.22	234302.13	205458.27	228686.36	217132.77	268690.66	255662.77	219156.36	236583.38	184571.88	205784.77	259826.52	411102.84	384128.44	323808.38	459913.25	411358.47	238465.63	259292.02	413329.03	279324.53	316825.53	292811.59	402238.94	400133.53	303625.53	361264.06
787.61636	Glc t18:1h20:0	Glycosylceramides	C44H85NO10	32982.43	61098.82	12403.71	58345.53	60636.07	61985.7	50640.8	51188.54	58656.16	66728.02	102103.73	62372.84	55087.75	69659.05	71476.09	79165.88	73244.68	69477.4	84603.13	74781.84	45038.91	54836.53	72800.77	54727.35	57106.02	46183.55	65114.82	72854.82	53616.28	69094.77
799.65293	Glc d18:1h22:0	Glycosylceramides	C46H89NO9	7235.52	16383.485	302.697	17528.745	22143.655	3628.245	5246.09	5739.635	6190.415	6858.035	9543.12	20364.085	18835.835	24197.39	27251.245	20303.245	19494.15	17411.455	21735.45	5639.49	12095.865	13393.9	25937.8	15502.965	17951.95	17171.985	16765.54	19403.97	14451.675	17406.675
815.6479	Glc t18:1h22:0	Glycosylceramides	C46H89NO10	206468.95	339501.31	72596.63	337149.5	387014.78	324462.97	320324.78	305354.47	329451.72	328278.09	464194.34	407152.16	355197.94	410788.16	466739.09	459606.84	463449.91	446749.91	526724.81	454421.94	318057.31	320366.22	505279.72	368349.09	374273.91	327628.31	448673.28	417973.78	371451.28	404519.38
825.66826	Glc d18:2h24:0	Glycosylceramides	C48H91NO9	62882.41	93438.55	20647.53	100276.71	105602.93	90731.04	80153.04	77652.13	82788.75	84041.41	107297.31	96959.47	86567.97	95119.21	98590.77	88769.45	84001.47	89999.8	89344.71	91450.84	65995.49	70303.77	103966.03	71254.44	77067.77	76584.02	90220.67	79167.68	78295.84	92276.64
839.6484	Glc t18:3h24:0	Glycosylceramides	C48H89NO10	16011.74	23482.98	4116.58	26767.72	24704.73	26728.15	25119.69	30240.83	30509.67	26580.97	41895.6	26494.99	24318.16	29949.06	36509.78	41912.01	37262.11	37082.55	37316.3	41329.84	22509.27	30364.14	44668.46	29137.06	32860.88	27962.73	41064.23	41344.68	34994.87	36579.05
841.6634	Glc t18:2h24:0	Glycosylceramides	C48H91NO10	733870.25	1321596.75	204745.11	1504752	1517586	1256870.13	1299928.88	1275025.25	1366346.88	1288231.5	1433854.63	1345582.25	1135040.88	1340491.38	1526408.38	1500461.25	1614778.75	1377516.5	2012720	1788875.38	1023353.44	1099168.63	1867241	1349837.63	1346564	1216333	1551273.88	1729018.75	1376826.75	1471247.63
843.67885	Glc t18:1h24:0	Glycosylceramides	C48H93NO10	1105707.13	1747563.13	372103.63	1612692.63	1780989.13	1209530.38	1503705.75	1546743.75	1659332.63	1621511.25	1572541.63	1514666.13	1202015	1415254.25	1561001.5	2210228	2258543.75	2026443.25	2512103.25	2036562.63	1690368.25	1854856.88	2485590.25	1779372.13	2106726.5	1678111.63	2211198	2291810.25	2082896.5	1724418.88
853.69978	Glc d18:2h26:0	Glycosylceramides	C50H95NO9	7679.57	11814.68	302.697	9631.14	11566.34	302.697	8250.06	302.697	302.697	7166.79	302.697	10444.05	8418.45	7057.53	11599.26	8098.48	11231.93	10329.23	11989.2	13548.6	8202.31	302.697	9923.53	302.697	9871.82	8209.37	8689.24	11123.66	8772.91	7940.66
857.69419	Glc t18:1h25:0	Glycosylceramides	C49H95NO10	16284.565	29003.21	3983.44	27679.2	34446.585	32815.61	33233.405	38012.05	35915.885	42818.615	39952.34	33955.945	26390.24	23434.665	31728.945	39839.11	41262.1	42324.835	44565.81	38878.24	30387.66	29004.975	46207.985	37488.515	28159.965	29404.945	36631.74	32494.82	28237.385	31297.545
869.69429	Glc t18:2h26:0	Glycosylceramides	C50H95NO10	128115.09	177362.53	40751.18	198594.47	201642.45	214684.23	215705.23	215799.69	241423.94	239306.17	187028.06	223113.05	165908.78	169894.19	203197.31	230186.13	235209.58	216907.39	297472.47	238217.16	185678.95	217968.36	278932.22	191899.83	194529.97	184172.22	254117.78	239025.89	217342.06	242901.63
871.7103	Glc t18:1h26:0	Glycosylceramides	C50H97NO10	242912.86	523757.88	46482.46	347854.22	475617.44	320730	361799.91	370590.72	451237.75	415934.31	391923.16	339836.22	288215.06	381903.34	419561.78	801272.56	537690.94	567326.81	629629.81	526000.63	338950.66	481023.34	599943.06	454284.88	634695.31	386963.09	562018.38	547571.75	442698.31	471205.84
495.33226	LPC 16:0	Lysophosphocholine	C24H50NO7P	16992.03	16473.39	4902.46	10351.68	9563.84	6275.34	10973.11	13222.65	21720.67	9300.22	302.697	9870.25	302.697	6773.11	302.697	21504.87	13327.75	302.697	22764.54	302.697	302.697	302.697	302.697	302.697	302.697	302.697	18722.98	302.697	22616.26	22308.75
517.31684	LPC 18:3	Lysophosphocholine	C26H48NO7P	15358.05	16182.23	5486.68	18169.87	13168.05	14763.97	15515.89	19435.1	35670.21	19155.6	15336.27	18752.93	16657.82	17475.56	19698.62	19805.44	15476.82	10916.25	22197.99	19837.99	12249.45	9554.46	15349.46	12508.59	12514.77	8644.92	43817.67	36771.46	14158.76	10772.78
519.33223	LPC 18:2	Lysophosphocholine	C26H50NO7P	21591.7	23356.31	9189.02	23964.37	18280	15426.54	21350.7	23425.08	45290.95	25829.71	21021.01	25670.06	19818.79	19767.82	26065.14	32722.75	20391.16	14271.75	28219.6	29003.72	20365.68	19425.87	26612.17	22672.64	19788.81	19088.25	58784.46	61729.66	33978.4	19871.2
521.34783	LPC 18:1	Lysophosphocholine	C26H52NO7P	7482.46	7039.62	2219.9	8140.44	7549.96	302.697	302.697	5765.79	7328.41	3921.36	302.697	4937.69	302.697	4045.99	302.697	12156.23	9035.49	302.697	11816.85	302.697	6867.94	7557.29	12250.92	9567.64	7574.57	302.697	21247.09	20577.87	11463.2	302.697
720.4932	PG 16:0-16:1	Phosphatidylglycerol	C38H73O10P	5211.18	302.697	3196.69	14178.75	26316.08	302.697	302.697	9818.83	24134.9	9029.52	302.697	302.697	302.697	8009.73	302.697	18821.06	12996.76	302.697	302.697	302.697	302.697	8159.76	302.697	11743.92	10053.56	9874.6	16134.28	15702.75	14295.68	7847.12
722.5105	PG 16:0-16:0	Phosphatidylglycerol	C38H75O10P	11269.12	14117.61	3191.71	13990.01	21927.77	8457.88	302.697	9113.97	23280.76	10662.24	6813.82	8097.19	4073.44	12460.09	6302.48	18657.91	15207.28	6753.89	9797.94	6966.18	7138.61	302.697	7550.52	7864.32	9928.43	7866.96	10926.66	9369.93	9937.42	10888.89
742.47756	PG 16:1-18:3	Phosphatidylglycerol	C40H71O10P	210453.39	282050.69	91379.49	386372.78	632614.13	169412.45	250218.14	312046.56	1292199.13	334372.63	221177.88	291253	95602.01	226521.84	245499.36	650637.44	377755.31	112366.37	244604.83	263893.75	166461.98	253875.25	212861.66	322916.69	303758.84	266684.03	515482.78	403157.38	357093.06	395935.56
744.49337	PG 16:1-18:2	Phosphatidylglycerol	C40H73O10P	22862.36	31910.59	12339.1	49633.73	70120.75	17607.88	25023.57	29076.24	111950.73	36220.58	25007.01	36675.56	12819.31	38525.83	27781.16	109912.21	63060.58	20710.11	34365.3	38704.32	25125.28	35521.96	32526.36	48399.99	54596.43	49141.02	85536.57	63887.01	63589.89	55633.07
744.49363	PG 16:0-18:3	Phosphatidylglycerol	C40H73O10P	86766.1	90708.59	39123.43	152609.77	221961.86	76377.21	95722.67	123650.8	568844.81	134986.45	122589.95	151589.05	46722.43	132366.53	105800.94	209597.78	143427.36	37650.32	93667.51	80725.73	49022.89	74077.23	68237.56	92801.1	94869.05	70311.42	112892.94	90375.93	92474.84	99978.84
746.50969	PG 16:0-18:2	Phosphatidylglycerol	C40H75O10P	17429.5	18754.295	9313.315	36607.7025	56807.69	10238.205	16643.4425	23627.1125	84776.1825	27632.3625	22745.72	31077.9475	9830.505	34695.1575	17269.765	62741.7325	45098.6425	11586.435	19928.1425	17764.085	13810.005	20283.78	15347.875	29523.7125	31928.34	27175.7475	45418.3175	33245.4675	35030.16	27864.1775
748.52477	PG 16:0-18:1	Phosphatidylglycerol	C40H77O10P	22363.4	24080.89	11988.17	52671.26	82013.59	15408.88	22994.5	33413.75	117853.29	43053.81	27495.91	47837.86	18995.03	69718.61	31307.81	94520.07	73937.73	18699.75	28390.87	26508.37	17473.32	31396.13	34485.54	50272.79	51780.11	34989.86	58181.39	51348.88	50779.15	34859.71

834.52738	Pil 18:2-16:0	Phosphatidylinositol	C43H79O13P	5268.05	302.697	2216.62	302.697	10598.95	6130.31	4378.94	4723.74	302.697	4019.31	6333.61	4073.57	2578.56	4458.12	5241.27	302.697	8924.07	3223.8	14997.31	7877.7	4159.39	302.697	7578.45	302.697	302.697	302.697	302.697	8202.03	6501.85	302.697
749.50235	PC 16:3-18:3	Phosphocholine	C42H72NO8P	6793.72	13542.6	2620.91	12235.16	14619.13	14316.52	18593.13	17716.55	24940.47	18374.41	14331.63	16993.7	11263.6	12727.24	23083.51	22304.04	18562.89	11573.7	19203.73	22071.45	12657.2	14080.78	18221.14	13153.59	9796.51	17223.14	19597.68	19477.6	12783.56	23100.3
755.54582	PC 16:0-18:3	Phosphocholine	C42H78NO8P	278292.245	438560.745	105264.1	418427.985	414985.26	379540.87	490715.08	473524.55	869158.71	481512.31	352241.815	436194.54	177392.69	264911.22	463194.565	705144.465	551498.145	314060.485	426804.48	403373.95	274637.25	299632.01	336179.5	292655.295	238537.85	503394.7	585717.53	490159.125	422348.54	634481.015
757.56143	PC 16:0-18:2	Phosphocholine	C42H80NO8P	416070.91	594240.69	169350.495	523731.185	563333.895	438114.765	552375.725	507512.77	1064913.88	570246.435	393698.11	557960.505	234355.755	366108.415	519354.66	798039.575	548057.175	345200.74	388047.815	400368.235	359629.275	336792.075	443299.03	359805.585	316644.595	553583.29	675327.23	575039.775	531919.255	649938.64
759.57703	PC 16:0-18:1	Phosphocholine	C42H82NO8P	192772.91	225078.44	64580.34	208116.81	214089.75	86981.66	129984.93	116364.96	239410.73	146469.69	106999.98	145055.75	55003.95	88013.34	128794.38	255627.13	224190.7	118593.53	136830.95	110744.36	126119.34	100654.66	203476.95	130369.09	114184.49	160855.09	207288.31	153218.31	154548.28	179217.88
767.55385	PC 17:1-18:3	Phosphocholine	C43H78NO8P	88077.62	208424.25	34009.3	197045.56	228469.59	189325.56	177853.64	176113.42	221980.78	209492.13	273041.88	206326.67	204771.34	340362.44	376672.78	351119.63	269259.53	275445.59	338960.5	475594.41	184477.3	221332.55	614354.63	227021.98	225917.22	206501.44	257674.11	328631.09	202382.14	286930.5
769.56659	PC 17:1-18:2	Phosphocholine	C43H80NO8P	16538.1	24539.35	4637.72	302.697	31226.28	25104.55	29370.33	27962	38060.58	302.697	302.697	27343.95	22224.24	30344.17	40186.78	33752.13	24840.27	19720.03	23060.97	19400.75	17185.64	15449.59	35343.69	18625.78	15839.72	23194.9	302.697	26590.51	21524.13	37179.9
777.53029	PC 18:3-18:3	Phosphocholine	C44H76NO8P	216106.5	367654.78	90583.59	355333	360975.88	394450.19	477089.91	495916.47	744753.94	487296.56	295656.13	353642.06	214487.42	301542.72	458254.59	595885.81	475105.78	277167.5	434540.13	477150.59	269348.19	287019.19	340287.97	263988.59	206769.77	431144.22	510955.38	484833.5	372785.44	718807.5
779.54366	PC 18:0-18:5	Phosphocholine	C44H78NO8P	65656.88	85625.07	25998.77	76474.27	82009.7	64840.41	89883.36	83150.99	174619.86	85056.66	55749.56	88119.3	31345.38	44457	73985.91	119166.2	76954.74	46711.18	55069.7	55467.1	53931.1	49330.8	59189.79	51376.6	46651.04	94752.49	109977.3	83797.99	85028.12	100874.89
779.54584	PC 18:2-18:3	Phosphocholine	C44H78NO8P	286992.39	502697.695	118419.385	469634.265	486297.785	469590.395	558730.74	555029.165	1042331.775	589010.865	344845.93	429989.555	202348.095	331114.96	510795.695	771272.99	527340.96	318683.885	436690.145	507005.365	312155.96	324000.355	407901.18	296166.595	244738.155	542849.34	586192.69	554235.915	436403.48	732070.845
781.56135	PC 18:2-18:2	Phosphocholine	C44H80NO8P	137456.51	170661.7975	59508.9575	183190.4425	160630.415	156132.0975	188911.64	180907.5825	271960.0825	191420.87	101553.29	152065.7925	75885.87	107051.555	150112.9025	198689.955	154714.67	115592.9725	134354.48	143874.9625	133889.8525	104065.3225	132856.5075	117872.8475	115570.1525	201224.6575	216802.6075	179281.9175	167648.3325	204628.2
783.57747	PC 18:1-18:2	Phosphocholine	C44H82NO8P	94331.0625	126054.4125	31345.67	122356.2825	119287.13	76078.2575	91306.195	90525.685	159309.535	91008.85	62999.965	83043.5875	35300.2525	57553.9425	77348.71	132182.58	100752.14	62608.8475	76501.47	69245.68	64680.3175	60827.405	111495.865	65640.5	58684.8125	96141.655	115153.8075	104980.13	91065.715	112276.74
785.59286	PC 18:1-18:1	Phosphocholine	C44H84NO8P	101730.8	145906.84	19544.19	138755.58	138243.38	95390.35	127506.62	111758.84	226473.5	117836.2	93324.15	132681.61	49037.12	79877.27	120962.55	150451.94	98986.02	72932.52	89632.87	78875.09	76461.86	74176.91	114428.99	89372.73	78757.25	108058.8	148365.97	146070.3	118346.55	134879.7
687.48343	PE 16:1-16:1	Phosphoethanolamine	C37H70NO8P	10596.26	5135.23	40428.76	83948.25	227310.75	3335.5	302.697	60047.94	302.697	128713.79	302.697	302.697	31052.59	148084.72	7751.68	302.697	68229.03	17351.07	9502.02	10945.3	302.697	10338.29	10308.6	302.697	80607.34	302.697	302.697	302.697	302.697	302.697
699.48312	PE 15:0-18:3	Phosphoethanolamine	C38H70NO8P	9227.14	18932.26	3876.79	16741.27	25162.17	7234.8	13062.27	12190.5	27119.17	7716.22	11670.11	10610.69	9089.62	19508.71	19854.79	39998.42	38665.58	14447.46	39463.44	29625.7	11670.43	21741.29	28222.36	19625.81	35403.15	12590.82	48929.36	39213.7	47042.05	12522.9
710.55849	PE 15:0-18:1-D7 STD	Phosphoethanolamine	C38H67D7NO8P	139695.67	159663.67	43764.79	182207.81	247903.66	84256.34	91213.33	125762.38	220240.38	150262.34	92499	126923.89	95160.84	189713.63	122419.28	142020.73	189164.31	101924.91	102628.45	114192.54	108542.91	91349.8	119496.82	140025.66	132819.63	93376.84	126627.83	103787.48	110926.41	102135.52
711.48317	PE 14:0-20:4	Phosphoethanolamine	C39H70NO8P	14536.22	7172.59	22615.15	68653.14	165646.5	5078.35	5899.65	42405.8	13398.12	82300.9	302.697	2877.94	28817.2	101147.09	10866.68	4020.59	63696.67	13367.79	11179.37	14889.11	9127.78	11713.11	12047.52	302.697	63419.34	10819.61	6329.98	302.697	5851.86	302.697
713.49895	PE 16:0-18:3	Phosphoethanolamine	C39H72NO8P	111450.94	169140.69	59586.57	198956.05	310971.5	80067.46	116775.48	208807.41	339774.47	185892.89	99066.91	155345.39	88572.69	169220.55	131253.88	190803.2	219253.56	120298.95	113301.23	109756.13	81602.34	81525.1	129336.51	114637.76	129461.28	85937.07	144367.48	85544.95	116082.29	112912.36
715.51514	PE 16:0-18:2	Phosphoethanolamine	C39H74NO8P	51111.02667	77324.44	25541.12333	83506.70333	156998.8267	36099.83	49462.22	81814.52333	146044.1833	87747.87667	49739.84667	64462.22333	30011.16667	77189.08	56251.63	80003.38667	98722.23667	44149.77	42847.86333	47303.17	31365.27667	26218.28	47624.52667	46929.78333	47376.46	40399.77667	62751.33	39211.41333	49210.50333	45436.89333
739.51479	PE 18:2-18:2	Phosphoethanolamine	C41H74NO8P	64295.13	99779.52	32387.21	87875.66	134750	51496.86	59187.4	107759.28	156569.88	76256.79	44019.73	73706.8	31132.24	53756.32	63088.88	79046.02	89349.55	39289.36	52218.76	42488.06	50007.63	34407.79	69556.78	48239.96	34687.75	52106.53	60380.56	19510.68	58368.54	39078.86
741.53284	PE 18:1-18:2	Phosphoethanolamine	C41H76NO8P	302.697	25944.4	5924.46	28335.29	39092.89	302.697	10383.34	17627.45	28211.02	16121.09	302.697	302.697	9348.57	11726.74	13868.31	302.697	25474.2	11550.86	16286.04	16818.29	302.697	302.697	21057.38	12777.74	13504.79	302.697	16302.17	302.697	302.697	302.697
813.6261	PE 18:2-23:0	Phosphoethanolamine	C46H88NO8P	10649.71	18204.89	4160.31	16658.74	17162.44	26235.89	15118.29	13469.79	15769.73	19364.06	11128.29	17038.74	12234.04	16090.94	14698.19	13502.9	15823.1	18519.67	14547.14	16343.49	12230.45	10124.15	13649.53	9268.47	13268.41	10688.08	13845.37	12636.02	12922.68	12862.36
827.63948	PE 18:2-24:0	Phosphoethanolamine	C47H90NO8P	4479.72	9029.39	4532.33	17919.44	33054.27	3679.56	5911.26	9820.53	19128.02	16437.4	4687.63	6551.11	4072.79	14799.44	5404.47	7682.14	19044.19	7509.26	6095.97	6678.25	4279.9	4344.42	8347.43	5626.46	13327.76	3977.94	6965.25	4639.06	6459.81	6095.39
245.23581	Sphinganine d14:0	Sphingosine	C14H31NO2	857884.44	484930.59	206199.31	169312.06	225329.52	794075.25	1362690.88	510240.53	302.697	302.697	2108206	167924.42	1877460.75	26097.85	1508976.5	88843.98	1292178.25	917593	302.697	302.697	72446.26	635078.31	769933.44	2341964.25	130506.79	55000.28	859010.63	302.697	302.697	302.697
259.25114	Sphinganine d15:0	Sphingosine	C15H33NO2	11590.93	6348.01	5811.77	2448.05	6178.98	11885.1	15870.88	11939.86	302.697	302.697	49765.18	4739.23	35206.31	302.697	20846.01	3783.82	21296.08	10240.24	302.697	302.697	302.697	3720.24	10208.35	38742.83	302.697	302.697	17197	302.697	302.697	302.697
261.23031	Sphingosine t14:0	Sphingosine	C14H31NO3	285870.59	166224.67	40307.73	58030.58	82120.64	112105.63	195424.52	293769.16	302.697	302.697	722060.75	21010.51	607432.56	5370.84	231495.77	7651.03	189325.41	159537.63	302.697	302.697	15840.63	136927.84	191956.09	496193.53	76087.23	14895.5	491210.63	302.697	302.697	302.697
271.25117	Sphinganine d16:1	Sphingosine	C16H33NO2	34254.6	41043.56	3709.02	302.697	19551.61	57048.72	71312.16	66624.91	302.697	302.697	79936.25	302.697	74870.71	302.697	71754.23	302.697	94460.81	31676.97	302.697	302.697	302.697	302.697	54436.97	81979.6	302.697	302.697	62325.65	302.697	302.697	302.697
273.26687	Sphinganine d16:0	Sphingosine	C16H35NO2	458296.66	372876.94	370451	79286.48	630289.69	773906.31	490152.81	1529726.88	7986.39	302.697	4946367.5	585104.31	2411378.75	95944.44	1301103.25	461409.81	910420.63	317875.59	16011.32	6820.32	371844.16	84739.82	392107.78	2674272	122826.54	27185.18	960348.38	15454.03	7823.65	14528.05
289.26149	Sphingosine t16:0	Sphingosine	C16H35NO3	43191.24	14655.585	22621.205	5865.475	62551.535	33505.85	30713.795	308129.835	302.697	302.697	378811.765	29955.03	207131.425	8446.35	37534.985	16404.655	52694.54	24232.92	302.697	302.697	16009.065	11391.515	36415.395	190348.615	20860.19	13040.16	219847.315	302.697	302.697	302.697
299.28142	Sphingosine d18:1	Sphingosine	C18H37NO2	8276.92	302.697	302.697	302.697	302.697	302.697	302.697	302.697	302.697	302.697	302.697	302.697	302.697	302.697	302.697	9469.27	12094.55	9320.69	9035.47	302.697	302.697	302.697	302.697	11735.26	302.697	302.697	302.697	302.697	302.697	302.697
301.298	Sphinganine d18:0	Sphingosine	C18H39NO2	5761.25	12410.04	13445.4	302.697	28844.19	10217.32	302.697	71902.38	302.697	302.697	284607.94	47056.09	59698.66	302.697	75471.06	19749.11	7196.06	302.697	302.697	302.697	31486.56	302.697	302.697	38847.41	302.697	302.697	14267.06	302.697	302.697	302.697
317.2932	Sphingosine t18:0	Sphingosine	C18H39NO3	12966.14	302.697	2266.94	302.697	9819.82	4294.07	302.697	35537.82	302.697	302.697	65053.27	9165.58	16274.27	302.697	302.697	8713.42	11669.77	302.697	302.697	302.697	7799.9	302.697	302.697	302.697	302.697	8176.72	302.697	302.697	302.697	302.697
788.47478	SQDG 16:0-16:3	Sulfoquinovosyl diacylglycerol	C41H72O12S	302.697	9002.19	302.697	4427.84	7224.19	6195.86	6047.04	4572.58	13580.09	5961.71	5438.21	4534.92	3662.91	302.697	5811.2	11706.95	5928.35	4482.97	8154.6	5594.94	3564.63	7917.87	7984.09	3950.23	2546.32	4282.86	12864.52	12651.63	9137.08	6903.04
792.50492	SQDG 16:0-16:1	Sulfoquinovosyl diacylglycerol	C41H76O12S	302.697	10195.57	302.697	302.697	302.697	302.697	302.697	302.697	14624.16	302.697	302.697	302.697	302.697	302.697	302.697	18976.94	302.697	302.697	8347.41	302.697	302.697	12864.56	302.697	302.697	302.697	302.697	15761.47	12274.69	16014.7	302.697
794.52025	SQDG 16:0-16:0	Sulfoquinovosyl diacylglycerol	C41H78O12S	18965.44	47947.15	8686.94	25800.86	37688.64	18039.44	14394.35	11581.37	61305.11	17260.01	17187.79	16265.53	6612.33	11104.79	17053.24	59002.3	32208.45	19771.87	26109.33	20955.08	17189.97	38540.14	25702.69	23631.29	16564.99	20500.71	85743.79	61443.02	64564.92	28452.88
816.50489	SQDG 16:0-18:3	Sulfoquinovosyl diacylglycerol	C43H76O12S	200159.09	581320.19	86217	307709.75	448162.22	319567.28	287686.41	257225.02	812963	274958.91	336859.5	294777.63	179213.66	242534.09	356012.91	842629.81	363910.06	253279.97	405686.25	321772.16	236493.2	549510.63	453945.69	259497.31	217499.55	179027.78	743012.31	643736.81	575829.44	360068
818.52068	SQDG 16:0-18:2	Sulfoquinovosyl diacylglycerol	C43H78O12S	29598.98	83272.23	12280.82	46002.15	70979.16	47734.73	39372.73	31859.85	119730.13	38561.21	53111.47	48515.04	32527.6	37621.45	56158.75	161348.92	80371.41	64706.95	86326.88	71051.48	38556.49	92871.85	77926.88	48908.68	45974.79	40186.39	155853.88	132125.66	120391.01	57483.79
820.53617	SQDG 16:0-18:1	Sulfoquinovosyl diacylglycerol	C43H80O12S	15146.57	39584.48	6394.74	23702.84	29647.98	21216.82	15401.44	11728.42	59548.35	16285.36	20960.13	22796.54	10944.53	17896.81	21713.16	77939.95	32303.6	24741.72	33180.2	21289.02	18714.89	41379.4	45776.89	25720.83	20380.73	19570.56	83798.7	69804.03	67435.34	29886.53
838.48924	SQDG 18:3-18:3	Sulfoquinovosyl diacylglycerol	C45H74O12S	54286.55	130715.52	24265.42	78213.7	113464.41	107421.33	85879.91	83841.69	181232.05	93841.02	124875.65	91527.54	71107.76	91393.09	139171.3	221462.56	110646.25	86594.68	161550	141647.28	91852.37	169159.05	158552.09	89504	72889.44	57686	183303.11	163323.36	153570.64	145122.78
840.50472	SQDG 18:2-18:3	Sulfoquinovosyl diacylglycerol	C45H76O12S	302.697	48704.06	302.697	302.697	302.697	302.697	302.697	302.697	60393.19	302.697	39684.95	302.697	302.697	302.697	302.697	84336.64	47545.81	52003.11	59898.71	46075.16	302.697	65116.52	51531.41	302.697	18639.46	302.697	69341.39	59196.17	53058.89	48485.07
842.52053	SQDG 18:1-18:3	Sulfoquinovosyl diacylglycerol	C45H78O12S	7635.06	20762.78	3675.7	13149.86	20842.16	11561.22	10121.5	7643.95	26368.24	10648.3	14489.36	13132.5	7176.39	9634.44	15133.46	31509.91	14756.1	11141.28	13707.34	11188.41	8746.62	16782.75	15025.11	8881.58	7599.74	6378.53	24837.07	22943.39	18620.42	13235.37
772.65725	TG 14:1-16:1-16:1	Triacyglycerides	C49H88O6	55098.05	41262.21	8338.26	40797	34115.31	41971.51	60877.26	47113.58	57954.24	42775.71	35366.46	42746.57	48628.6	72877.05	66517.57	112199.94	68105.3	55910.36	66394.85	49410.16	47975.63	58251.93	55689.18	91989.55	50742.01	34211.71	22560.44	63324.93	56921.16	47928.76
774.67294	TG 14:1-16:0-16:1	Triacyglycerides	C49H90O6	238460.64	137715.91	33063.16	170360.56	131616.22	159843.53	246073.2	215198.92	218694.63	177385.14	133810.91	172791.64	207895.09	283181.59	277920.31	442929.28	288091.25	220051.09	290510.91	201573.91	208612.53	245591.05	231226.58	406370.53	211880.86	127136.41	97023.42	276626.31	241473.52	208919.95
776.68856	TG 14:0-16:0-16:1	Triacyglycerides	C49H92O6	436474.56	266340.63	58371.01	291656.25	233602.89	328435.63	464334.53	378097.56	428282.06	329439.66	220992.23	287527.88	336758.22	462723.16	465837.94	856060.13	506676.31	412125.06	478185.91	325465.94	368820.38	418965.94	351900.78	762552.63	361396.22	235656.23	164260.08	477054.66	431562.38	377658.16
778.70401	TG 14:0-16:0-16:0	Triacyglycerides	C49H94O6	369019.28	312318.84	76122.09	284512.72	238454.66	316769.63	337962.06	350552.88	332733.19	308170.47	197069.47	261587.86	245754.94	355308.91	343905.72	626227.88	375381.06	318939.03	368072.75	258680.97	274488.44	307672.91	249795.78	541437.25	289391.56	205298.22	164213.25	392416.78	365194.53	336344.06
786.67312	TG 15:1-16:1-16:1	Triacyglycerides	C50H90O6	69880.25	56111.63	7254.85	76146.41	55215.52	61775.48	81504.29	69355.91	76660.75	60410.58	46533.31	42867.71	92902.81	109473.78	92238.72	147683.11	75545	90096.58	104287.45	56872.42	90961.89	79027.66	87320.72	149092.84	68193.38	41521.76	31422.14	105246.84	80566.57	51486.64
788.68856	TG 15:1-16:0-16:1	Triacyglycerides	C50H92O6	210939.39	134421.58	29409.17	157170.94	118490.3	171755.17	220436.11	195889.11	197459.78	163601.16	117170.42	151180.23	190612.88	252079.86	255800.64	412109.97	256270.48	220451.92	257043.2	181107.67	183756.34	218857.66	209302.5	360389.44	183765.61	117434.59	87196.7	242828.88	216136.38	184539.47
790.70415	TG 15:0-16:0-16:1	Triacyglycerides	C50H94O6	397105.53	273921.88	50674.89	293363.63	218474.53	304780.53	382721.09	338074.34	347100.31	278502.5	192406.56	262174.16	303404.78	414624.59	392949.66	741667.31	425149.19	381802.22	447077.38	290628.97	305878.31	383933.59	327079.59	639830.88	308964.34	197513.27	139143.73	409783.19	363864.22	329931.59

792.71956	TG 15:0-16:0-16:0	Triacyglycerides	C50H96O6	325661.31	266066.22	58711.63	235185.28	200228.41	273484.69	273776.53	274436.97	270605.53	247022.64	171717.92	217706.94	207455.34	305792.59	286691.72	585386.31	322117.81	287972.56	331386.72	220072.09	227520.09	286809.44	224552.2	447629.66	242575.02	176014.67	137529.92	320498.69	299748.78	299035.84
796.65676	TG 16:1-16:1-16:3	Triacyglycerides	C51H88O6	4846.95	302.697	302.697	302.697	302.697	302.697	302.697	302.697	4875.78	302.697	302.697	302.697	17591.34	3169.24	4169.98	6807.48	302.697	302.697	3395.45	302.697	302.697	3364.24	302.697	302.697	2416.31	302.697	302.697	302.697	302.697	3023.91
798.67278	TG 14:1-16:1-18:2	Triacyglycerides	C51H90O6	13768.27	9917.05	2300.6	12567.85	9600.17	11041.73	17082.95	15019.12	16498.25	12477.81	10288.28	11365.36	16483.54	19899.21	20537.56	31697.51	17912.44	14447.71	20452.19	14506.67	12424.63	16310.2	15287.02	24583.05	12783.35	9355.06	7115.09	18185.63	16390.66	12098.52
800.68845	TG 14:1-16:1-18:1	Triacyglycerides	C51H92O6	119576.07	68735.26	20749.92	80976.07	66224.7	76850.885	120956.155	103120.545	110685.155	90824.08	67345.895	77194.425	101237.29	130866.325	124308.875	204717.685	138613.76	114816.23	132387.03	95256.33	97417.305	113632.485	109273.54	195321.285	104051.535	63852.42	45610.335	123988.85	112250.33	93732.405
802.70428	TG 16:0-16:1-16:1	Triacyglycerides	C51H94O6	552279.63	332868.53	71717.92	410357.69	327516.88	423747.03	619877.63	466669.28	564417.69	427480.56	286336.22	385299.16	474735.28	615525.56	610051.19	1105219.88	657783.25	543604.69	593763.81	432259.09	495793.72	584468.88	497500.53	993888.69	492044.81	306295.31	203866.41	613829	558740.06	494702.5
804.71983	TG 16:0-16:0-16:1	Triacyglycerides	C51H96O6	725975	509702.53	89120.87	528473.56	416775.75	609705.69	693289.75	648489.06	668979.19	553491.06	377101.03	471041.5	547967.69	777086.56	741145.56	1410857	780756.69	681432.56	767206.94	488435.13	586864.19	740728.31	581232.06	1216085.38	584784.63	402944.97	265908.25	752642.25	695911.94	620100.31
806.73513	TG 16:0-16:0-16:0	Triacyglycerides	C51H98O6	151210.22	118294.68	59915.61	131913.11	95641.95	104858.07	127762.29	148333.69	143328.7	122471.8	96287.17	103447.09	95582.28	119049.2	122072.88	168268.67	110298.54	97502.68	116096.94	93133.38	104965.75	96505.87	96269.78	165835.25	115848.97	87374.77	65199.85	133611.97	129898.88	113936.55
806.73518	TG 14:0-16:0-18:0	Triacyglycerides	C51H98O6	525545.94	468513.91	106607.49	416145.09	395936	487000.41	435798.84	475495.31	472797.94	435924.84	292134.03	358360.47	339341.5	472769.97	418319	979051.13	497569	473104.13	508516.47	372836.31	386721.94	499753.25	371902.41	697358.19	419562.28	315158.97	212254.89	534053.31	467278.75	489369.5
814.7041	TG 15:0-16:1-18:2	Triacyglycerides	C52H94O6	99735.98	60525.14	14997.39	77537.73	60454.97	78988.26	105585.11	96054.13	95277.99	81523.1	53589.11	72821.16	86296.2	120756.15	117913.88	191929.47	124284.1	101145.19	121660.41	82504.98	91381.4	105651.82	97001.13	166824.58	91524.97	56622.05	41668.97	114800.57	98944.77	86078.65
816.71963	TG 15:0-16:1-18:1	Triacyglycerides	C52H96O6	330572.31	206931.06	39033.29	241160	197334.48	249930.34	304746.28	291239.88	307189	234135.89	164044.39	234700.86	266360.16	350092	349215.72	654483.5	374904.06	324687.69	373904.56	261081.98	274370.56	343945.16	300166.41	538596.5	282159.97	180422.98	120887.31	354700.28	313748	268436.56
818.73536	TG 16:0-16:1-17:0	Triacyglycerides	C52H98O6	413783.16	273136.41	49432.18	274035.34	248619.13	336640.69	369660.13	344022.97	348568.47	285537.97	196109.44	271670.44	295993.03	432046.97	411597	821548.88	446976.06	377310.5	421908.5	279862.72	296647.09	416170.69	346528.75	650043.63	314700.81	205991.48	144431.94	412954.09	364668.72	355476.56
820.75089	TG 16:0-16:0-17:0	Triacyglycerides	C52H100O6	240342.77	199870.47	45601.99	184010.17	154968.72	197615.19	182740.13	189086.63	187126.55	189911.69	119200.66	149682.28	135735.47	224137.63	183120.22	428981.25	222629.36	206055.33	221405.72	151942.41	160726.98	214143.31	153565.91	294872.25	176906.89	123652.16	96795.66	207416.06	204046	192045.27
822.67287	TG 16:0-16:3-18:3	Triacyglycerides	C53H90O6	302.697	10935.79	2574.87	19226.94	14475.9	16050.72	13603.07	18224.83	23955.09	11263.92	25904.75	30773.05	30991.31	23257.45	32201.66	23020.5	24006.82	25608.13	35338.8	20084.08	18239.34	15723.21	19159.47	15587.99	16482.77	14643.64	23689.7	26490.67	23733.29	25705.51
826.70399	TG 16:0-16:1-18:3	Triacyglycerides	C53H94O6	44334.09	32803.14	7772.57	37895.31	33433.93	34923.57	51159.05	48385.91	54697.69	42895.71	40444.28	45688.24	47760.41	59859.65	59613.1	84912.79	59976.73	51645.88	71818.97	45076.68	39980.92	52594.4	51166.45	79975.45	47081.93	32522.26	30385.8	66454.76	51672.61	50455.61
828.71972	TG 16:0-16:1-18:2	Triacyglycerides	C53H96O6	274101.97	165817.58	37499.91	209085.55	175483.25	211958.17	305841.59	237902.69	309177.16	221401	180595.13	234623.23	255050.77	303405.75	332124.22	536016.63	350141.84	306576.03	338153.38	219344.27	243187.38	291741.56	249745.88	483421.19	249917.09	170695.86	128088.99	336527.88	288802.5	253722.83
830.73549	TG 16:0-16:1-18:1	Triacyglycerides	C53H98O6	577197.94	389112.31	71641.29	442953.91	379039.53	531817.56	593769.56	566609.5	597544.06	499119.59	349909.38	460663.69	502388.25	686280.75	687970.88	1189422.88	690464.94	610640.38	668251.88	453056.25	497050.69	674849.56	515528.47	1019452.25	488228.16	365624.47	247028.98	689894	585980.44	538605.81
832.75104	TG 16:0-16:0-18:1	Triacyglycerides	C53H100O6	581193.63	433894.38	65090.96	411685.34	363405.34	511157.47	485626.56	508687.47	562127	497763	280074.03	396034.16	380075.91	565203	504372	1157324.38	576666.44	525941.31	596433.56	434739.66	457041.25	576294.06	455863.28	837302.25	433412.06	302019.03	203285.08	634120.69	578811.63	528088.56
834.76647	TG 16:0-16:0-18:0	Triacyglycerides	C53H102O6	330288.44	306935.81	49470.22	260701.56	231366.36	303783.41	226113.83	232538.83	262946.13	260572.83	162482.25	210803.02	179316.25	267084.81	224510.44	496635.22	310331.19	279038.13	309695.34	204313.84	217532.81	328844.38	218317.02	331538.16	241692.13	227515.98	147858.8	300534.25	297542.13	271974.78
836.6796	TG 15:0-18:3-18:3	Triacyglycerides	C54H92O6	302.697	302.697	1592.52	8020.93	8153.74	10361.59	12699.8	10810.83	8029.92	10756.52	302.697	8802.93	9063.97	11481.19	14515.9	12323.87	16030.29	11740.51	16670.66	7674.67	4721.42	302.697	6486.58	12948.76	302.697	302.697	302.697	302.697	10214.74	10477.25
840.71944	TG 15:1-18:1-18:2	Triacyglycerides	C54H96O6	25299.38	16323.69	3117.03	18464.83	17385.07	21720.15	27226.29	26128.84	28409.82	22593.72	20140.39	24549.26	25478.92	34428.75	31168.52	52564.02	31717.65	29308.34	39894.54	25183.77	23348.22	28939.66	32581.14	45019.09	23499.91	16902.85	13505.87	32611.59	30632.7	26779.8
842.73512	TG 16:0-17:1-18:2	Triacyglycerides	C54H98O6	106230.36	64527.31	14092.4	78011.45	64577.27	78631.38	104947.98	93157.56	100796.13	80949.56	60863.44	79649.36	86463.36	116886.96	122447.66	206506.7	122064.94	106911.02	129747.42	83342.02	88708.4	109081.84	104019.76	175243.89	89374.24	58266.93	43757.39	118941.84	100753.71	92610.27
844.65715	TG 16:3-18:3-18:3	Triacyglycerides	C55H88O6	16751.99	18342.37	8965.95	30537.22	31755.92	24622.1	25228.2	21308.02	47431.82	29222.91	34549.7	32961.26	34599.71	32604.35	52612.97	46367.94	63646.46	47007.48	77741.95	43255.2	64249.25	34613.78	30791.65	40046.11	31552.97	29176.98	56468.53	53598.84	52495.38	55733.41
844.75082	TG 16:1-17:0-18:1	Triacyglycerides	C54H100O6	195052.56	143209.25	24811.74	145731.98	128484.52	176213.61	196348.92	187163.7	182445.59	151731.89	105759.32	137194.53	149311.73	224718.33	222020.36	415494.69	238700.02	209641.3	216450.84	153443.08	158444.39	224668.09	180287.08	335534.75	174009.92	114938.73	78078.58	224611.69	204023.91	192218.55
846.76645	TG 16:0-17:0-18:1	Triacyglycerides	C54H102O6	191086.56	136330.34	24256.03	132638.41	112366.35	166361.42	157604.02	146056.25	153546.39	142779.59	87652.33	109781.23	114927.02	188677.72	164222.94	385028.06	185661.42	164083.56	178024.5	124024.35	125873.91	189767.23	134916.95	259412.66	141386.09	97941.9	67803.45	169399.55	161261.14	142968.63
848.68839	TG 16:1-18:3-18:3	Triacyglycerides	C55H92O6	36435.96	51971.21	11441.52	61921.68	63211.37	59258.78	59151.61	55587.6	76284.86	62614.05	63382.1	72657.65	64324.04	68019.18	96853.21	105358.68	106989.79	86350.26	125259.82	105134.6	68706.09	68403.2	66278.73	70085.83	71669.43	72486.52	107208.52	108221.7	85666.12	96979.83
848.78207	TG 16:0-17:0-18:0	Triacyglycerides	C54H104O6	87592.28	83224.03	15498.83	77847.35	65682.23	80032.75	70557.95	64067.98	68474.34	71726.23	43509.66	56495.23	47569.3	78205.27	64966.97	150611.36	88284.63	85233.21	84016.02	50907.41	59764.16	84321.73	56795.86	104296.44	60634.92	56438.14	37651.3	81366.55	72341.88	76108.75
850.70385	TG 16:0-18:3-18:3	Triacyglycerides	C55H94O6	75423.32	106700.52	33446.135	141789.64	150556.815	167205.815	154691.665	153583.055	207628.765	154583.3	208241.91	213382.3	205743.655	196804.95	253013.46	202265.985	229497.355	184734.81	293117.995	185332.91	133086.715	142915.93	121673.34	155799.495	158554.45	139810.245	220619.08	225178.725	198231.555	221144.14
852.71956	TG 16:0-18:2-18:3	Triacyglycerides	C55H96O6	175515.14	245938.08	55329.34	321766.44	329884.97	328197.41	338866.56	325338.44	440533.84	371816.16	450662.63	467827.69	455614.56	466188.88	601177.56	450476.38	509677.53	399434.44	597951.06	382404.84	246780.33	272489.03	254956.64	335944.66	314978.03	293263.63	408813.34	467844.56	395974.19	440069.13
854.73521	TG 16:0-18:2-18:2	Triacyglycerides	C55H98O6	233347.19	267041.31	48406.74	251709.5	307041.78	276156.97	338573.5	314248.06	418194.25	343316.41	325051.97	372468.59	389677.91	436743.94	476624.38	456750.28	388844.38	313778.56	537655.69	345860.13	234053.39	263893.91	511338.44	412431	263293.66	198580.59	241293	424874.31	350776.78	367143.09
856.75081	TG 16:0-18:1-18:2	Triacyglycerides	C55H100O6	215595.57	179284.48	37522.33	160233.52	177912.14	174721.455	214950.445	218054.71	263150.315	229118.14	138468.16	212552.635	184542.66	242272.72	228750.095	342289.84	244703.065	210673.635	279453.175	204380.875	163483.755	194414.725	200434.325	307323.175	175642.88	120245.69	111072.875	280757.75	240420	211297.21
858.76667	TG 16:0-18:1-18:1	Triacyglycerides	C55H102O6	419176.41	293135.44	49723.93	276834.22	276351.22	351625.16	343498.81	366883.16	506500	454123.09	218068.58	325358.53	273399.81	440966.5	363088.63	747232.19	394616.19	396782.13	552005.94	393527.28	302042.81	385094.41	338838.25	546965.13	304351.88	200998.16	171256.97	600047.81	486180.5	423553.22
860.78212	TG 16:0-18:0-18:1	Triacyglycerides	C55H104O6	271670.78	210474.17	31251.8	182399.28	164212.06	225474.53	209600.23	191029.14	279822.69	295874.19	110474.13	163096.5	154885.13	234045.48	207326.13	442173.91	233797.08	218623.16	288772.81	222459.98	171803.3	252200.91	175819.8	319922.69	168758.2	142535.77	95839.83	298647.09	301805.16	254490.56
862.79785	TG 16:0-18:0-18:0	Triacyglycerides	C55H106O6	231540.73	238065.53	28784.36	244103.63	154977.39	253522.3	155398.7	160646	166052.25	202227.8	101493.38	141813.47	91653.31	152280.55	122446.84	341871.13	276613.56	232136.73	184324.28	121792.64	205313.64	261745.28	139794.38	217902.84	210486.34	180605.72	159022.03	172660.94	178836.89	158386.08
870.76643	TG 17:1-18:1-18:1	Triacyglycerides	C56H102O6	53735.88	42697.02	6181.39	35162.22	34382.18	43627.22	54273.45	49044.87	51383.46	44253.97	30485.2	44353.5	42723.1	64393.28	56371.94	110088.82	62498.86	54425.95	62727.11	44777.52	44549.17	59366.67	51140.17	84639.06	43610.11	32573.63	21917.13	59913.22	56698.83	50873.5
872.68819	TG 14:3-18:3-22:3	Triacyglycerides	C57H92O6	172608.73	270657.59	62063.67	359698.06	376865	319597.22	305111.47	325406.63	414382.78	316911.81	391370.13	377772.38	388551.5	338601.81	523751.31	612798.31	664659.63	439973.56	731827.19	542724.88	341114.72	393316.53	324047.31	393813.75	431271.69	416637.41	650169.31	641190.31	534189.06	562213.38
874.70406	TG 18:2-18:3-18:3	Triacyglycerides	C57H94O6	506914.25	817507.06	171863.86	1026858.19	1134566.5	978372.25	921500.75	885597.06	1127877.88	951569.75	1084657.63	1131033.38	1144971.38	1029278.25	1583847.38	1429250	1514463.38	1119112.63	1558205.13	1309719.5	841331.44	934083	770901.69	897655.06	984892.63	1027548.88	1558334.5	1512950.63	1179374.13	1429944.38
876.71936	TG 18:2-18:2-18:3	Triacyglycerides	C57H96O6	138050.61	194962.73	68777.34	232329.38	223641.7	195648.28	204510.42	193787.28	231016.13	220343.59	199435.72	220619.48	231889.19	204794.22	272305.34	208956.52	220616.98	174697.19	248724.83	196950.97	144521.11	149715.48	134055.38	159253.16	173336.55	175478.34	238314.16	239215.59	211051.64	207441.63
876.71963	TG 18:1-18:2-18:4	Triacyglycerides	C57H96O6	255411.72	422752.84	85817.48	517402.72	534321	431370.53	437695.06	439225.91	575961.5	486975.03	539000.38	611717.19	559806.63	555572.81	850564.69	618499.94	668467.19	456693.44	786426.31	590891.75	370475	371273.75	376656.81	403281.09	436377.88	425997.91	617577.88	718876.19	559158	605976.44
878.73496	TG 18:2-18:2-18:2	Triacyglycerides	C57H98O6	91513.24	150815.31	37823.4	117751.25	136329.7	92199.82	94448.54	106133.99	129476.91	146021.84	119876.58	124066.59	137355.67	129872.09	153851.95	106137.73	114070.65	96485.77	160768.03	133076.67	77195.74	65472.38	300912.16	105925.15	85530.16	66733.33	107441.23	127007.58	110701.04	108553.84
878.73508	TG 18:0-18:3-18:3	Triacyglycerides	C57H98O6	181963.67	320399.63	49675.84	264005.41	326297.38	182245.08	224988.48	238711.83	313377.88	319622.06	282997.22	303507.31	353638.34	333640.63	418302.78	305023.47	329939.69	240017.8	526438.44	385581	189975.03	172911.78	982941	299071.31	223553.97	158602.63	264622.03	377659.13	310044.25	291279.13
880.75054	TG 18:1-18:2-18:2	Triacyglycerides	C57H100O6	189300.28	307937.63	32506.19	162265.64	263912.56	156580.81	186039.17	199421.02	266956.97	272557.25	208933.78	237698.8	283598.34	269986.38	279730.19	302314.41	240676.06	208722.08	528153	375076.19	152972.41	151386	377498.81	290176.03	174582.83	96314.82	138770.69	319489.41	265167.22	229774.98
882.76616	TG 18:1-18:1-18:2	Triacyglycerides	C57H102O6	204663.95	255361.39	29973.12	148380.14	241062.88	171708.78	182443.91	202042.56	248009.55	258434.06	161515.7	229359.3	219757.72	232613.34	239148.77	338117.22	235246.81	214830.17	399363.28	294617.56	140298.13	149438.97	218433.27	267684.19	174653.03	84527.16	117220.05	297504.53	240463.41	203956.55
884.78168	TG 18:1-18:1-18:1	Triacyglycerides	C57H104O6	93238.73	91224.69	26659.14	60746.55	78739.27	69705.58	79854.06	89467.71	115138.02	89477.8	51435.44	71176.57	61623.15	82075.34	82345.9	100827.52	69866.88	75660.75	115968.91	92798.18	50110.36	56125.5	67206.02	85922.82	62955.45	37884.64	45921.52	116289.62	89844.24	86564.95
884.78201	TG 18:0-18:1-18:2	Triacyglycerides	C57H104O6	278277.81	299288.47	37055.44	160664.5	276885.44	227778.5	230395.36	243915.89	338173.44	292234.31	147320.92	222992.19	186617.7	276899.78	268275.66	462428.44	264254.56	293658.94	496847.69	352396.94	153013.33	223073.08	213673.55	320547.28	203137.3	113243.16	136841.48	449475.25	294757.66	295231.34
886.79734	TG 18:0-18:1-18:1	Triacyglycerides	C57H106O6	144847.34	147293.72	18591.05	106565.48	135424.47	118106.43	117304.99	117505.13	146620.73	156401.55	79856.89	109559.17	100112.31	153442.14	119491.31	242703.8	139282.22	140013.61	201305	140801.8	91396	128040.42	107272.2	184242.67	102091	75204.49	64088.75	188644.94	144957.02	135240.91
890.81661	TG 16:0-18:0-20:0	Triacyglycerides	C57H110O6	50282.65	49162.47	10661.76	47381.1	36395.65	46209.52	43468.25	44490.04	46913.35	46211.52	31137.37	38427.28	30128.37	46637.36	38938.59	76923.89	58920.92	43945.42	50692.6	37029.07	50017.47	48802.3	37644.83	59621.22	47928.02	40808.29	35071.73	52593.23	50818.75	45989.06
890.82881	TG 14:0-16:0-24:0	Triacyglycerides	C57H110O6	209002.42	201452.05	23023.18	428227.5	119536.54	242648.14	148065.59	119069.98	92869.17	182288.7	53755.63	119374.67	53784.24	92184.29	73846.16	278188.38	266540.44	251686.14	143690.67	81542.81	185213.59	234006.19	99130.27	191775.64	188799.61	160877.64	174543.67	130948.62	96516.18	108301.89
898.79698	TG 18:1-18:1-19:1	Triacyglycerides	C58H106O6	17669.42	15541.4	1794.25	12986.58	11704.66	17593.6	16626.45	17654.92	17734.76	14419.84	9519.66	13075.06	13868.08	19255.71	13707.64	44173.64	19564.64	19947.01	19158.58	16516.43	12925.9	19741.33	17067.02	25943.51	15615.95	10558.24	8811.39	22243.38	17353.25	13496.17

900.81273	TG 15:1-16:1-24:0	Triacyglycerides	$C_{58}H_{108}O_6$	37804.33	32596.89	4926.07	28005.29	26550.13	33577.47	35331.35	29405.27	30735.49	32306.8	19285.74	23717.64	24060.09	42745.69	35503.66	74174.63	41254.15	40901.83	40199.11	24808.1	32086.83	37871.06	31510.48	57121.51	27413.76	22570.11	15655.41	39294.26	31336.71	32174.44
904.74945	TG 18:2-18:3-20:2	Triacyglycerides	$C_{59}H_{100}O_6$	24227.99	189717.31	5627.68	38859	92818.16	16840.56	19255.75	19899.95	24980.81	21175.54	23295.55	38368.88	30087.23	99424.98	33863.66	35579.49	86145.38	27860.26	127598.25	43039.76	18139.11	16775.51	23171.33	20622.08	19820.36	20045.29	30648.71	28071.77	23112.02	29579.84
904.8441	TG 15:0-16:0-24:0	Triacyglycerides	$C_{58}H_{112}O_6$	66349.46	60238.69	11063.99	65179.07	54806.14	54864.04	53518.06	54190.2	48870.75	53749.94	29255.57	43976.97	34413.6	62909.55	45838.31	113586.18	72482.77	55595.32	67126.05	41039.53	48175.96	61373.98	46208.13	80427.52	43978.47	44695.59	30022.99	57297.27	52151.55	53813.33
906.7652	TG 18:3-18:3-20:0	Triacyglycerides	$C_{59}H_{102}O_6$	43963.49	291672.69	6779.99	51988.73	195349.95	20095.48	19290.62	302.697	25378.38	22192.29	25445.4	47414.5	43260	162567.52	40678.91	55910.93	139428.17	46439.84	226845.17	74575.71	20649.73	16995.58	42441.69	21783.88	19924.62	16188.11	48030.54	26949.98	20167.68	49037.54
908.77978	TG 18:2-18:2-20:1	Triacyglycerides	$C_{59}H_{104}O_6$	53814.84	288930.63	7080.37	55907.21	227431.89	20906.97	22073.01	23120.7	27223.35	24125.27	24066.06	51194.77	51703.38	172451.94	37427.98	56863.6	159153.02	45436.5	233247.59	79991.02	19958.09	18324.4	27195.43	28505.78	19284.86	15817.8	43127.57	31796.83	22989.64	50288.64
910.7971	TG 18:1-18:2-20:1	Triacyglycerides	$C_{59}H_{106}O_6$	38350.36	169504.56	5048.8	39311.78	152012.53	27443.94	25381.52	26706.77	30506.09	29003.02	24762.38	38516.34	36050.44	103568.94	35884.41	61370.46	103132.8	41867.93	173844.63	54161.76	24882.01	25603.42	27908.36	36456.19	19799.1	16333.98	29619.02	28365.97	25500.64	40424.54
912.81262	TG 18:1-18:1-20:1	Triacyglycerides	$C_{59}H_{108}O_6$	40257.56	146032.16	5194.15	40020.08	138893.52	32951.99	29164.37	27724.68	29583	30628.14	18309.62	35866.14	31114.36	87390.27	33374.18	71940.91	85505.28	39828.43	146384.52	52097.56	27541.78	36256.27	31227.7	43475.61	24957.68	19594.02	24151.65	38117.53	32616.78	45231.19
914.81643	TG 18:0-18:2-20:0	Triacyglycerides	$C_{59}H_{110}O_6$	28928.59	49591.77	6547.13	23517.51	48069.62	22661.48	25257.55	22061.29	29384.68	29488.66	16086.94	24763.38	25783.09	47321.38	26364.99	41417.43	36449.84	26959.78	63423.27	35589.66	21593.18	24585.81	21517.5	39723.7	22725.69	18296.37	15684.4	35876.3	30837.51	29506.16
914.82844	TG 16:1-18:1-22:0	Triacyglycerides	$C_{59}H_{110}O_6$	79273.59	87822.12	8529.41	58772.73	87003.96	59291.04	58895.64	57465.77	61415.02	61303.14	36456.52	51355.73	48180.12	94353.86	66167.07	147168.78	83546.92	72963.31	102573.73	54769.04	61276.17	74522.84	57996.2	101374.08	54412.24	44037.14	28535.45	70109.62	61486.11	61569.1
916.84401	TG 16:0-16:1-24:0	Triacyglycerides	$C_{59}H_{112}O_6$	126391.3	106031.33	12973.24	106420.59	84540.55	98166.2	101774.11	85439.9	85859.13	103655.91	44936.38	83380.51	61742.47	129491.67	95272.34	243849.23	124608.85	125410.38	138284.11	73958.11	92132.73	116708.3	84585.49	165208.11	84337.84	77543.57	46901.98	118562.95	104001.66	96182.59
918.85943	TG 16:0-16:0-24:0	Triacyglycerides	$C_{59}H_{114}O_6$	68061.17	67421.46	9787.19	94587.55	60393.86	57876.96	52778.45	48032.2	44394.89	57929.79	27387.24	44062.59	30047.56	57856.18	44026.64	105752.94	77564.2	58123.5	71777.37	39807.69	49593.09	72250.2	52715.77	78329.61	40652.05	49450.68	33805.4	65037.12	56591.86	55469.9
928.84366	TG 16:1-17:1-24:0	Triacyglycerides	$C_{60}H_{112}O_6$	46955.09	39778.88	5572.2	31330.02	32032.2	36949.39	41226.38	34513.89	33742.21	38941.85	20655.17	29398.13	28326.82	48752.82	39100.99	81535.01	48670.79	43882.95	48496.08	29294.09	36562.31	43086.86	33827.5	63632.99	32253.1	29487.3	19583.38	40572.05	37205.05	32049.15
930.85941	TG 16:1-18:0-23:0	Triacyglycerides	$C_{60}H_{114}O_6$	85023.57	73217.46	10847.54	72138.26	59807.96	71318.91	76898.57	68884.61	62930.3	69368.04	37277.06	59684.72	48911.07	93296.94	72889.88	156861.14	97797.21	79699.5	97926.85	47867.27	68386.41	79586.42	64936.48	115694.61	63320.12	53842.28	33810.01	81830.1	70454.16	65935.82
932.78096	TG 18:3-18:3-22:1	Triacyglycerides	$C_{61}H_{104}O_6$	4106.18	39465.14	302.697	8005.47	17295.36	302.697	302.697	302.697	302.697	302.697	4206.27	7153.33	302.697	22225.99	302.697	302.697	16449.85	302.697	23197.57	11326.16	302.697	3215.99	302.697	302.697	302.697	4053.01	7210.76	302.697	302.697	5872.8
932.87488	TG 15:0-16:0-26:0	Triacyglycerides	$C_{60}H_{116}O_6$	43839.23	41753.81	7172.59	43032.05	36911.42	37003.81	34577.68	33133.8	33641.5	39484.71	20168.33	29825.94	23556.44	40386.87	32244.85	79456.58	51886.63	42160.27	50604.12	32796.92	34112.11	46293.26	34962.14	54003.08	30650.5	33204.2	23895.35	46478.46	37410.42	37852.25
934.79564	TG 18:2-18:3-22:1	Triacyglycerides	$C_{61}H_{106}O_6$	15560.26	87975.06	1776.5	14064.93	47173.53	7840.39	6529.57	302.697	302.697	5531.27	7385.31	13735.57	13541.59	46609.81	10348.82	16297.15	37934.87	14541.62	54737.55	20398.37	6455.25	5130.87	302.697	5969.59	7605.19	6598.89	11974.95	14496.43	9370.2	12055.69
936.81079	TG 18:3-20:1-20:1	Triacyglycerides	$C_{61}H_{108}O_6$	24255.13	131943.66	302.697	24877.99	76989.24	302.697	8639.28	10521.25	10662.1	10381.26	9112.38	20347.74	16620.56	70596.52	16090.37	25584.37	60145.68	20265.95	98445.24	30549.24	9211.92	10588.32	11493.08	302.697	302.697	302.697	17517.64	13140.46	9030.2	22334.84
938.82801	TG 18:2-20:1-20:1	Triacyglycerides	$C_{61}H_{110}O_6$	302.697	121846.76	2459.12	22822.08	86674.6	16158.67	16121.09	13578.89	18530.51	20105.69	13707.92	21770.98	23245.68	302.697	18300.73	28116.36	67351.37	24974.43	302.697	302.697	12376.54	16026.7	18653.89	20452.58	11858.56	9816.61	16984.63	20369.32	15935.03	302.697
940.84327	TG 18:2-20:0-20:1	Triacyglycerides	$C_{61}H_{112}O_6$	30413.11	82593.4	4008.49	24703.99	63067.29	22919.7	24341.89	22418.48	22258.83	26303.71	15285.19	23452.57	23065.94	50002.78	25450.84	50046.43	42578.7	28528.45	73304.89	29617.01	22155.23	26028.76	24202.97	36302.53	18393.42	15456.33	16546.85	27358.26	23958.7	28422.25
942.85891	TG 16:1-18:1-24:0	Triacyglycerides	$C_{61}H_{114}O_6$	64523.62	63504.64	6912.45	55666.2	52910.11	52011.5	56910.66	45551.71	52072.79	55854.42	31023.87	49520.7	39034.95	72494.49	50307.91	121686.24	71936.63	68652.18	89494	43242.85	50745.5	59464.53	48801.76	93336.5	46977.06	43558.14	28592.54	65272.22	62874.41	53294.57
944.8746	TG 16:0-18:1-24:0	Triacyglycerides	$C_{61}H_{116}O_6$	78030.27	65898.64	8433.73	68624.51	58553.74	59517.71	60797.28	49762.29	54753.5	60772.01	30430.33	49848.44	37798.07	80877.02	61196.52	132258.55	82359.41	67332.05	85255.16	46267.56	55041.96	72160.24	58947.83	100496.5	48987.44	51719.64	29691.2	75269.09	64716.62	64834.13
946.89025	TG 16:0-18:0-24:0	Triacyglycerides	$C_{61}H_{118}O_6$	26510.69	26372.49	3973.96	28832.71	25212.39	21876.49	21643.55	22016.1	18517.4	24692.09	10486.15	17181.51	11155.37	20382.02	17429.61	42395.86	29578.64	25748.39	29064.64	16583.51	19397.76	28239.84	22333.63	28230.4	16593.6	20803.19	13908.43	27678.88	23515.51	21852.77
956.87438	TG 16:1-18:1-25:0	Triacyglycerides	$C_{62}H_{116}O_6$	35466.37	26937.73	5267.44	34608.37	25102.02	28980.57	32643.38	29523.67	26848.38	31073.48	15041.22	26306.53	22933.18	39850.46	31205.2	63186.86	40133.36	32086.54	38043.2	23439.09	29033.36	35805.92	27703.95	49052.23	26746.17	24025.26	14291.48	33291.84	28084.95	26698.13
958.88993	TG 16:0-18:1-25:0	Triacyglycerides	$C_{62}H_{118}O_6$	43110.96	34815.73	5145.65	34314.18	27480.52	31855.7	32719.13	31727.37	28858.98	32486.87	15246.55	27027.35	22338.43	38604.41	32752.66	71578.13	42481.83	36057.5	42736.19	26881.6	29906.4	36547.66	30170.77	53119.59	26436.37	27012.64	16481.82	38667	32000.71	31549.22
960.90597	TG 16:0-17:0-26:0	Triacyglycerides	$C_{62}H_{120}O_6$	13513.99	14287.08	2312.17	15151.73	13683.82	12199.39	9917.3	11383.37	10221.21	14742.53	5908.47	8793.17	6418.76	12871.79	11049.41	21191.16	15502.45	11715.65	16103.96	7493.49	10488.07	13166.58	12327.08	15132.79	9111.99	9995.46	7143.12	14984.16	10477.64	13070.71
964.84008	TG 18:3-20:1-22:1	Triacyglycerides	$C_{63}H_{112}O_6$	6762.61	26827.06	302.697	8154.56	15968.4	7552.06	6233.87	302.697	6441.21	7387.62	4961.53	7081.43	6923.46	17959.3	6899.5	11573.67	15232.16	9003.42	24349.66	8698.02	6156.87	6595.44	302.697	6751.97	5624.85	5110.64	6952.08	8035.45	6141.82	8768.29
966.85849	TG 18:2-20:1-22:1	Triacyglycerides	$C_{63}H_{114}O_6$	10511.86	24025.76	302.697	9889.44	15772.46	8768.92	8604.19	7946.99	9528.75	11115.32	7921.44	10180.54	9567.06	16615.33	11169.61	17604.34	15510.49	11554.94	19510.69	12585.26	8075.82	10563.21	9214.34	12643.33	7358.32	6116.67	6770.98	12048.06	9304.22	10705.14
968.87382	TG 18:1-18:2-24:0	Triacyglycerides	$C_{63}H_{116}O_6$	18282.72	22868.54	2100.57	14085.26	17054.94	14111.52	16608.43	13681.8	13289.44	15607.2	9200.43	14076.6	12688.94	23392.14	15454.34	31051.6	23913.64	16296.1	27095.02	14589.98	14334.65	17526.29	15408.17	22523.37	12465.22	11776.33	8379.6	16059.8	14765.04	15485.09
970.88959	TG 16:1-18:1-26:0	Triacyglycerides	$C_{63}H_{118}O_6$	33645.39	32002.16	3563.29	28835.95	28243.01	28429.8	25913.88	24847.29	24250.53	30353.02	15055.54	25488.85	19452.82	39660.14	29034.57	59544.22	37417.18	31152.28	39343.84	23122.71	28045.95	31249.69	28176.47	48754.06	24710.55	22807.36	14859.41	34025.35	30134.13	28929.55
972.90479	TG 16:0-18:1-26:0	Triacyglycerides	$C_{63}H_{120}O_6$	25497.26	23796.53	3052.41	21522.89	18959.18	19327.78	19871.71	20746.31	17322.72	20570.81	8401.39	16771.8	11532.75	23927.77	18658.87	42777.58	26485.31	22590.14	27583.95	15772.65	18800.8	24075.31	19598.86	32393.48	15954.44	18971.99	10801.12	24544.18	22799.23	19986.36
974.91315	TG 16:0-18:0-26:0	Triacyglycerides	$C_{63}H_{122}O_6$	10484.09	6659.96	302.697	9561.46	8014.69	6268.06	8626.87	9128.59	9090.06	8306.46	4680.37	8234.33	6270.58	10187.28	9077.32	10517.21	9872.4	7857.26	13144.46	7294.17	7199.77	9326.57	9078.3	12933.59	7526.97	7989.3	302.697	10410.64	9493.26	9277.64
982.88938	TG 18:1-18:2-25:0	Triacyglycerides	$C_{64}H_{118}O_6$	9783.87	6684.44	302.697	5919.48	6189.82	6222.68	6336.03	6678.49	6355.89	6690.61	3578.95	6576.29	5329.18	8977.73	6458.3	15312.82	9805.94	8203.2	9783.94	4780.91	6374.41	8585.77	7389.37	11530.23	6431.45	5473.14	3000.39	8098.76	6235.23	6644.17

Mass (M)	Name	Compound	FORMULA (	Ler-R1	Ler-R2	Ler-R3	Ler-R4	Ler-R5	arc3-R1	arc3-R2	arc3-R3	arc3-R4	arc3-R5	arc5-R1	arc5-R2	arc5-R3	arc5-R4	arc5-R5	Col-0-R1	Col-0-R2	Col-0-R3	Col-0-R4	Col-0-R5	kac1/2-R1	kac1/2-R2	kac1/2-R3	kac1/2-R4	kac1/2-R5	chup1-R1	chup1-R2	chup1-R3	chup1-R4	chup1-R5
774.4552	Arabidopsi	Arabidopsi	C43H66O1	1.3417	1.144306	1.848282	1.215401	0.715162	2.226339	2.988777	1.721335	1.610915	1.540501	2.264664	3.692483	2.857236	1.730325	2.326003	1.868716	1.324998	2.878054	2.548855	1.572769	5.739935	5.184048	5.757273	4.898673	5.543343	7.105988	5.071166	7.166314	5.210725	4.212252
802.4869	Arabidopsi	Arabidopsi	C45H70O1	0.010834	0.05543	0.006916	0.028837	0.016816	0.05834	0.06687	0.002407	0.001374	0.002014	0.003272	0.002385	0.082044	0.054068	0.084085	0.037628	0.02455	0.02727	0.068962	0.01677	0.1231	0.175063	0.19675	0.185617	0.22406	0.134076	0.175805	0.198403	0.128659	0.002964
537.5117	Cer d18:1h	Ceramides	C34H67NC	0.471969	0.702332	0.350977	0.470412	0.454541	0.593286	0.476829	0.406156	0.300295	0.383755	0.851423	0.629999	0.685519	0.495856	0.779291	0.367284	0.315974	0.673515	0.742917	0.377722	0.234411	0.426414	0.505848	0.276564	0.364798	0.284528	0.238979	0.469706	0.337959	0.318699
553.5072	Cer d16:0h	Ceramides	C34H67NC	0.501545	0.873544	0.410459	0.668548	0.599426	1.023031	0.934187	0.691497	0.474422	0.701962	1.133923	0.962285	1.037319	0.663812	1.098566	0.834394	0.731602	1.184967	1.662524	1.238241	0.655803	0.986061	1.207945	0.687733	0.804728	0.725261	0.848487	1.088065	0.828149	0.927775
555.5227	Cer d16:0h	Ceramides	C34H69NC	0.388776	0.635867	0.364482	0.497799	0.428054	0.666738	0.58398	0.481077	0.292009	0.426604	1.005483	0.651762	0.774819	0.394291	0.79554	0.440616	0.502403	0.931871	1.036838	0.539155	0.329661	0.438774	0.541135	0.290473	0.340243	0.237913	0.284618	0.400493	0.291882	0.404165
569.5014	Cer t18:1-1	Ceramides	C34H67NC	0.464282	0.861067	0.454108	0.717735	0.611152	1.489395	1.434694	1.012177	0.684479	0.942678	1.659415	1.175198	1.305983	0.715568	1.443025	0.995647	0.714598	1.225866	1.574447	1.518943	0.892689	1.255109	1.462601	0.841813	0.968811	0.973048	1.057413	1.42241	1.041666	1.224572
571.5172	Cer t18:0h1	Ceramides	C34H69NC	0.404847	0.593797	0.328674	0.580988	0.410876	0.940884	0.985366	0.679751	0.40209	0.664221	1.227869	0.959762	1.110268	0.472506	1.010488	0.277085	0.301633	0.551208	0.597262	0.426627	0.237249	0.362485	0.596179	0.278151	0.319945	0.247624	0.275549	0.464101	0.269563	0.329712
581.5379	Cer d18:0h	Ceramides	C36H71NC	0.095307	0.243631	0.106994	0.175543	0.112662	0.003593	0.223645	0.144819	0.067286	0.169903	0.452781	0.15284	0.182391	0.139503	0.202957	0.091871	0.087919	0.155795	0.235258	0.173522	0.093341	0.128936	0.181308	0.083864	0.115921	0.074339	0.085263	0.136579	0.146535	0.123903
609.5692	Cer d18:1h	Ceramides	C38H75NC	0.656838	0.514559	0.807166	0.649277	0.373103	0.931362	0.866081	0.827094	0.337414	0.729725	1.138912	0.921518	1.059498	0.592047	0.75818	0.474863	0.367246	1.078939	0.942065	0.581018	0.504653	0.759733	0.639554	0.608734	0.774608	0.719941	0.584639	0.738663	0.539997	0.494998
625.5641	Cer t18:1h2	Ceramides	C38H75NC	0.17943	0.241723	0.146529	0.208703	0.167484	0.459897	0.438704	0.2892	0.194164	0.259825	0.580433	0.366828	0.429763	0.259426	0.445956	0.325162	0.155868	0.374831	0.396966	0.323278	0.202663	0.330427	0.368145	0.269831	0.223204	0.219467	0.203812	0.364874	0.27491	0.348712
651.5797	Cer t18:2h2	Ceramides	C40H77NC	0.106475	0.213117	0.141552	0.187402	0.188596	0.483789	0.370889	0.27182	0.16401	0.336033	0.579018	0.333976	0.539422	0.265202	0.411559	0.184523	0.177987	0.330012	0.346022	0.384867	0.185037	0.257141	0.305353	0.147603	0.215664	0.207051	0.217449	0.310935	0.221626	0.308137
651.6524	Cer d18:0h	Ceramides	C42H85NC	0.179506	0.252603	0.137808	0.219629	0.176367	0.336347	0.212802	0.24868	0.160101	0.171343	0.336957	0.255832	0.33397	0.184781	0.310869	0.241225	0.164953	0.272051	0.286607	0.177152	0.17568	0.315341	0.228179	0.200946	0.153539	0.196918	0.149513	0.236803	0.203944	0.236253
653.5954	Cer t18:1h2	Ceramides	C40H79NC	2.616662	3.657067	2.380334	3.047976	2.448298	6.426321	6.109828	4.090142	2.616123	4.023033	8.534454	5.351353	6.52828	3.347428	5.889573	3.256929	2.40893	5.174206	5.116696	4.511276	3.981508	5.287144	5.664371	3.22921	3.639694	3.583798	3.732525	4.836067	3.817984	4.240816
655.611	Cer t18:0h2	Ceramides	C40H81NC	0.518568	0.985175	0.350535	0.649367	0.69188	1.297504	1.170156	1.028441	0.655499	0.920736	2.218121	1.297347	1.644096	1.052105	1.698327	0.542744	0.382297	0.880873	1.036733	0.720215	0.57188	0.573462	0.739743	0.401661	0.500893	0.284581	0.367333	0.522579	0.400214	0.430108
663.6159	Cer d18:2h	Ceramides	C42H81NC	3.676877	5.2708	4.186421	4.703412	3.614066	5.700149	6.04846	4.383163	2.667557	4.321776	8.421467	5.910172	6.479433	4.2516	6.313144	3.460046	3.063827	4.379412	5.12195	4.562943	3.704162	4.369438	4.500533	3.26287	3.670926	3.878308	3.69136	5.140087	4.29302	4.592952
665.595	Cer t18:2h2	Ceramides	C41H79NC	0.735978	0.789436	0.959541	0.734762	0.553711	1.680384	1.572768	1.171225	0.658818	0.919566	1.653631	1.471796	1.986154	0.967372	1.281176	0.807422	0.7375	1.25549	1.256119	1.174509	1.010055	1.079895	1.297782	0.927928	1.063935	1.199026	1.147382	1.431022	1.045593	1.109195
665.6316	Cer d18:1h	Ceramides	C42H83NC	3.412056	6.120344	2.805527	4.564607	4.173519	7.401552	6.008435	5.116451	3.200662	4.607963	9.146971	5.712477	6.424533	4.219769	7.330229	5.798535	3.547064	5.416961	6.763869	6.841336	4.029058	5.858281	7.193508	4.180633	4.428947	3.896873	4.776112	6.477094	5.013491	5.834358
667.6109	Cer t18:1h2	Ceramides	C41H81NC	1.00083	1.881886	0.629198	1.239663	1.088763	2.334959	2.367454	1.783988	1.345683	1.710447	4.434404	2.485128	3.100128	1.882298	3.15773	1.623122	1.053408	2.020566	2.398555	2.304602	1.77928	1.846316	2.411177	1.465044	1.532712	1.206978	1.306657	1.971977	1.4549	1.613364
669.6265	Cer t18:0h2	Ceramides	C41H83NC	0.152661	0.293781	0.006916	0.166442	0.331117	0.46793	0.426538	0.303433	0.193638	0.223944	0.554888	0.273186	0.415874	0.182149	0.480238	0.273113	0.138881	0.391996	0.373459	0.262352	0.229166	0.277854	0.24057	0.210978	0.002279	0.17815	0.252517	0.336054	0.271184	0.270257
675.5796	Cer t18:1h2	Ceramides	C42H77NC	0.040748	0.059398	0.006916	0.056815	0.048612	0.140742	0.096945	0.082891	0.054611	0.073592	0.189256	0.072217	0.096358	0.033677	0.077743	0.089044	0.065231	0.102368	0.142311	0.171589	0.060118	0.10172	0.131231	0.082884	0.08005	0.094193	0.11604	0.180454	0.12077	0.137973
677.6313	Cer d18:2h	Ceramides	C43H83NC	0.303423	0.613367	0.348997	0.514108	0.410359	0.867937	0.791394	0.608328	0.31084	0.534918	1.216118	0.839622	0.988947	0.522499	0.899209	0.514319	0.504024	0.658415	0.884159	0.695914	0.50342	0.598448	0.61648	0.457548	0.53691	0.458999	0.542622	0.616678	0.529605	0.534601
679.6113	Cer t18.2h2	Ceramides	C42H81NC	8.212615	12.75098	7.199733	11.04317	8.782894	22.84019	21.39353	16.44979	9.233738	13.63282	25.80341	17.33614	20.11476	10.64514	19.82392	11.02401	7.71132	12.63335	20.44294	18.7892	11.96259	15.68787	18.30877	10.00312	12.06606	11.35251	12.17549	17.01557	13.34013	15.94047
681.6272	Cer t18:1h2	Ceramides	C42H83NC	4.023847	6.769972	3.157309	5.500264	4.761673	10.78885	9.802123	7.585029	5.055308	6.694162	12.84345	8.261869	10.34159	5.595315	10.20702	7.028688	4.382145	8.061691	9.709624	9.507584	6.521642	8.981225	11.71798	6.102887	6.69248	6.074586	6.773607	9.59704	7.288133	7.9111
683.6413	Cer t18:0h2	Ceramides	C42H85NC	0.727186	1.594099	0.6857	1.222649	1.664503	2.166369	1.964314	1.617748	1.118623	1.493688	3.164516	1.87739	2.221325	1.168908	2.433208	0.960581	0.702088	1.531471	1.960464	1.295531	0.961811	1.22984	1.689336	0.866861	0.873923	0.712896	1.027579	1.443142	1.003937	1.058548
691.6471	Cer d18:2h	Ceramides	C44H85NC	1.201189	2.532559	0.91112	1.572942	1.383223	2.6906	2.543313	1.970722	1.202621	1.989207	2.7842	2.003095	2.363513	1.499232	2.466572	2.219828	1.240915	2.195526	2.759637	2.516546	1.512552	2.286082	2.558409	1.550043	2.215939	1.716873	1.979738	2.782444	1.862576	2.246906
693.6265	Cer t18:1h2	Ceramides	C43H83NC	1.747432	2.244709	2.095362	1.799223	1.487309	3.78248	4.300191	2.811424	1.778878	2.601759	4.343853	3.206228	4.050981	1.926099	3.254613	1.690401	1.312818	2.508315	2.927401	2.646967	2.072731	3.2478	3.008801	1.974812	2.01338	2.209475	2.123816	2.831001	2.253356	2.471854
693.6637	Cer d18:1h	Ceramides	C44H87NC	1.339081	2.37999	1.426209	2.127345	2.039305	3.256467	3.07877	2.574933	1.813938	2.151423	3.598083	2.430609	2.464782	1.530455	3.116957	2.854372	1.859157	2.856021	3.783834	3.049554	2.071707	2.624327	4.673532	2.350533	2.022057	2.147102	2.969805	3.894626	2.979606	2.774185
695.6422	Cer t18:1h2	Ceramides	C43H85NC	0.310932	0.634622	0.264008	0.456833	0.420024	0.980756	0.850545	0.71118	0.503664	0.587052	1.435812	0.745557	0.868745	0.549643	0.991518	0.614873	0.423098	0.569469	0.896038	0.758933	0.521092	0.7214	0.947729	0.501887	0.556593	0.405588	0.545658	0.817702	0.640243	0.653342
695.6786	Cer d18:0h	Ceramides	C44H89NC	2.648847	4.569862	2.404445	3.586172	3.941871	4.976574	4.407668	4.164916	3.039234	3.349582	6.525261	4.749922	4.442807	2.758172	5.696234	2.808333	2.002756	3.467859	2.969116	2.768769	1.862888	2.600098	3.839978	2.06649	1.68168	1.801186	2.306859	2.922563	2.425683	2.378754
697.6574	Cer t18:0h2	Ceramides	C43H87NC	0.115862	0.232142	0.103128	0.209543	0.364603	0.283475	0.276672	0.244098	0.184992	0.249766	0.31804	0.276586	0.356476	0.143778	0.347953	0.191531	0.171068	0.334092	0.392849	0.163023	0.19114	0.281005	0.251618	0.186679	0.159813	0.171702	0.251417	0.279428	0.288909	0.191943
707.6421	Cer t18:1h2	Ceramides	C44H85NC	1.974491	4.224237	1.859769	3.047023	2.754549	6.670611	6.633999	5.299961	3.285842	5.15366	8.533208	5.026888	6.109304	3.541958	6.691714	3.846595	2.643419	4.589314	6.414098	6.367458	3.465737	4.619117	6.369344	3.608462	3.923877	3.50522	4.067315	5.815928	4.347118	4.584317
709.6593	Cer t18:1h2	Glycosylcer	C44H87NC	1.560711	2.78171	1.258413	2.519144	2.365164	5.316304	5.000537	4.151347	2.819322	3.387425	6.423408	4.086258	4.275172	2.292133	4.762718	3.092094	2.141776	3.411175	4.120647	3.839726	2.706477	3.686754	5.075584	2.616747	2.537494	2.370259	3.081548	4.136544	3.27892	3.15663
711.6742	Cer t18:0h2	Ceramides	C44H89NC	0.479471	0.890404	0.486589	0.858928	1.044136	1.335388	1.40478	1.195648	0.905806	0.951928	2.397558	1.549684	1.497768	0.773489	1.98098	0.550136	0.384466	0.60596	0.776609	0.607859	0.507829	0.55549	0.814883	0.475933	0.416876	0.389062	0.446535	0.679837	0.558918	0.552752
646.5684	CE 18:3	Cholestrol	C45H74O2	0.079877	0.120849	0.070449	0.108238	0.099492	0.140473	0.110773	0.067905	0.044157	0.087077	0.239694	0.150107	0.178292	0.11216	0.202306	0.122555	0.098171	0.140709	0.17294	0.12804	0.140952	0.163697	0.166432	0.119474	0.120277	0.120719	0.081969	0.14498	0.09259	0.121617
648.5841	CE 18:2	Cholestrol	C45H76O2	0.155848	0.134841	0.056374	0.094896	0.081966	0.194819	0.156338	0.114622	0.082622	0.099112	0.168287	0.157435	0.144771	0.114678	0.16867	0.223339	0.121685	0.200071	0.168127	0.123878	0.11	0.195119	0.163854	0.153888	0.113859	0.155139	0.069378	0.148176	0.150347	0.16068
650.5994	CE 18:1	Cholestrol	C45H78O2	0.199413	0.114554	0.079046	0.094902	0.063822	0.226103	0.219352	0.158959	0.08445	0.10169	0.124763	0.134402	0.155529	0.134994	0.164398	0.216236	0.116191	0.180519	0.152259	0.105448	0.137635	0.215936	0.13849	0.193968	0.129463	0.17706	0.071973	0.204103	0.144587	0.207808
536.4439	DG 14:1-16	Diacylglyce	C33H60O5	0.09699	0.001896	0.006916	0.001661	0.001221	0.003593	0.111185	0.069346	0.001374	0.002014	0.003272	0.002385	0.003181	0.001596	0.002473	0.120807	0.055756	0.065254	0.0838	0.002651	0.057183	0.003314	0.002533	0.094722	0.054204	0.003242	0.00239	0.079796	0.06395	0.002964
538.4593	DG 14:0-16	Diacylglyce	C33H62O5	0.136471	0.083386	0.051005	0.098022	0.031015	0.003593	0.286955	0.130487	0.072162	0.105638	0.113498	0.063834	0.157909	0.108222	0.169254	0.194124	0.120785	0.190504	0.201636	0.119462	0.1312	0.162301	0.1193	0.177286	0.116346	0.085973	0.066308	0.163487	0.138446	0.140181
540.4752	DG 14:0-16	Diacylglyce	C33H64O5	0.151343	0.135299	0.100192	0.107687	0.057401	0.229493	0.289926	0.171925	0.138008	0.133133	0.174168	0.160164	0.200281	0.15729	0.225422	0.24382	0.127303	0.226035	0.316731	0.192709	0.14135	0.242724	0.181868	0.218267	0.150602	0.141072	0.090581	0.23134	0.182759	0.227948
552.4746	DG 15:0-16	Diacylglyce	C34H64O5	0.174423	0.001896	0.051878	0.084343	0.001221	0.003593	0.242862	0.123943	0.086695	0.091278	0.003272	0.126248	0.003181	0.113111	0.002473	0.220313	0.090496	0.20927	0.204538	0.002651	0.161682	0.252604	0.002533	0.18867	0.148134	0.003242	0.00239	0.193472	0.163451	0.002964
554.4904	DG 15:0-16	Diacylglyce	C34H66O5	0.129976	0.11561	0.006916	0.098831	0.054352	0.187177	0.239463	0.146764	0.071324	0.11121	0.13271	0.112794	0.170615	0.113828	0.149188	0.17193	0.10879	0.137787	0.196269	0.137624	0.089831	0.179241	0.108625	0.158342	0.106801	0.098042	0.080288	0.194853	0.170942	0.142446
562.4596	DG 16:0-16	Diacylglyce	C35H62O5	0.070801	0.138471	0.006916	0.100496	0.075623	0.225271	0.191304	0.153921	0.110843	0.100903	0.198814	0.207549	0.182519	0.133836	0.258096	0.18603	0.181743	0.247253	0.351201	0.262129	0.123034	0.202476	0.204419	0.164734	0.165275	0.300733	0.273544	0.30451	0.208756	0.306361
564.4752	DG 16:1-16	Diacylglyce	C35H64O5	0.182086	0.095388	0.077441	0.129407	0.057333	0.218025	0.310302	0.155828	0.072203	0.108811	0.196612	0.129425	0.220835	0.109696	0.270593	0.24908	0.138346	0.190622	0.310111	0.170408	0.156582	0.201516	0.171836	0.213238	0.128195	0.161515	0.058413	0.238575	0.191805	0.20299

566.4908	DG 16:0-16	Diacylglyce	C35H66O5	0.511782	0.299148	0.201199	0.304121	0.176738	0.593553	0.799048	0.492875	0.304984	0.338728	0.368965	0.446126	0.609737	0.40113	0.630893	0.791716	0.358114	0.664309	0.705247	0.464564	0.486262	0.754731	0.456854	0.685744	0.372397	0.330496	0.271244	0.654601	0.592869	0.590505
568.506	DG 16:0-16	Diacylglyce	C35H68O5	0.754799	0.616427	0.4782	0.49126	0.444433	0.95524	1.255788	0.644951	0.800843	0.571129	1.404415	0.733507	1.194788	0.63275	0.904178	0.980716	0.572094	0.830236	1.831011	1.121211	0.732935	0.976719	1.220444	0.80273	0.677458	0.714736	0.524705	1.377981	1.241987	1.487989
582.5215	DG 16:0-17	Diacylglyce	C36H70O5	0.122053	0.001896	0.091386	0.001661	0.001221	0.133107	0.292674	0.101323	0.08813	0.002014	0.003272	0.002385	0.003181	0.001596	0.002473	0.208752	0.0016	0.00297	0.002949	0.002651	0.002789	0.157776	0.002533	0.140419	0.002279	0.098192	0.00239	0.200104	0.168003	0.13217
584.4439	DG 16:3-18	Diacylglyce	C37H60O5	0.178486	0.248259	0.309559	0.28522	0.281536	0.530094	0.389897	0.257247	0.256395	0.250185	0.557514	0.329249	0.441712	0.235173	0.519066	0.278071	0.284211	0.302499	0.650955	0.437757	0.174722	0.200629	0.260748	0.180441	0.150368	0.238855	0.306263	0.394408	0.419345	0.774631
586.4593	DG 16:3-18	Diacylglyce	C37H62O5	0.04009	0.084581	0.041786	0.059195	0.083355	0.155901	0.13426	0.101533	0.07687	0.080776	0.201135	0.115328	0.206423	0.089057	0.245009	0.153233	0.101115	0.155196	0.295596	0.209953	0.113341	0.13508	0.156885	0.09253	0.118426	0.118578	0.156065	0.220866	0.155874	0.247552
588.4751	DG 16:1-18	Diacylglyce	C37H64O5	0.180394	0.327378	0.204463	0.244703	0.245986	0.521998	0.475636	0.290815	0.241052	0.30386	0.636103	0.528523	0.659018	0.407701	0.963619	0.448722	0.392062	0.590513	0.912451	0.749734	0.315016	0.53011	0.811675	0.405977	0.470842	0.436273	0.672687	0.670079	0.495759	0.690419
590.4904	DG 16:1-18	Diacylglyce	C37H66O5	0.166786	0.107621	0.006916	0.120113	0.092796	0.170231	0.175827	0.147172	0.06703	0.103976	0.302608	0.143706	0.324274	0.087062	0.144699	0.195656	0.164865	0.300297	0.453859	0.212965	0.136053	0.174175	0.237403	0.184808	0.205069	0.23505	0.170514	0.450745	0.323501	0.312933
590.4904	DG 16:0-18	Diacylglyce	C37H66O5	4.376794	6.751386	4.880381	6.629601	5.587149	13.32515	13.00868	9.349892	6.556012	8.266769	15.40398	11.33726	13.4331	7.77776	15.5442	11.27685	9.587977	14.52082	22.70033	17.19284	8.776724	12.82757	15.54973	10.47904	10.56507	14.68745	13.24194	19.01944	13.16252	20.06568
592.5063	DG 16:1-18	Diacylglyce	C37H68O5	5.275245	7.7593	5.50797	7.306805	5.920009	14.35594	14.37999	10.26532	6.750581	8.546501	16.77103	13.24296	14.71389	8.590855	17.19786	11.74724	9.554006	14.52202	20.74235	16.30112	8.427663	12.13993	14.49592	9.699461	10.22105	13.382	13.33868	20.20445	14.76579	17.31716
592.5064	DG 16:0-18	Diacylglyce	C37H68O5	3.758454	3.617498	6.472054	3.829498	2.66167	7.797507	8.418794	5.981495	3.75029	5.178196	7.202708	5.344135	5.913078	3.185334	5.809374	4.396962	3.327268	5.731366	5.877014	4.67469	4.172742	5.126354	5.01075	4.231494	4.704583	7.218834	5.440188	7.939065	6.503383	6.097215
594.5219	DG 16:0-18	Diacylglyce	C37H70O5	1.061611	1.022505	0.804398	0.940486	0.725205	1.50135	1.701806	1.277359	1.034455	1.483376	1.424331	1.39133	1.753818	0.882638	1.698677	1.78851	1.200997	1.869879	2.998626	2.003014	1.262953	1.869524	2.61891	1.615712	1.080866	1.414681	1.215141	2.748142	2.547268	2.718885
596.5375	DG 16:0-18	Diacylglyce	C37H72O5	1.184071	1.21275	0.525553	0.877392	0.662903	2.304901	1.659862	1.099711	0.81098	1.069856	1.673543	1.195423	1.263886	0.714182	1.143122	1.397913	1.36501	2.070821	2.259862	1.663621	1.561939	1.91935	1.644152	1.387317	1.876547	1.894312	1.776749	1.866539	1.479996	1.740536
604.5061	DG 17:0-18	Diacylglyce	C38H68O5	0.055795	0.071475	0.051014	0.063053	0.042099	0.118668	0.12311	0.086872	0.057172	0.073672	0.138305	0.131384	0.123579	0.060525	0.140208	0.113495	0.099627	0.145135	0.227352	0.164807	0.061277	0.104701	0.148768	0.106322	0.092494	0.104697	0.107945	0.182517	0.133697	0.187641
612.475	DG 18:3-18	Diacylglyce	C39H64O5	2.258035	2.396675	3.288707	2.635224	2.008669	6.281509	4.934575	3.989034	2.57099	3.16648	5.845672	4.054727	5.352484	2.467516	4.853303	3.524116	3.20028	5.005101	7.80033	4.842041	3.434731	3.607697	3.946445	3.067636	3.456819	4.601209	4.389692	5.729973	4.141487	5.706874
614.4906	DG 18:2-18	Diacylglyce	C39H66O5	1.026803	1.607533	1.057859	1.68016	1.381262	3.045519	2.824232	2.175107	1.665369	1.957299	4.01224	2.672657	3.108212	1.901122	4.140484	2.529856	2.018417	2.75835	4.49567	3.494981	1.579716	2.04047	3.032671	1.808035	1.938086	2.629448	3.021158	3.875897	2.711452	3.385027
616.5063	DG 18:2-18	Diacylglyce	C39H68O5	1.383865	1.354131	3.050463	1.308543	1.141046	2.43692	2.716711	1.984555	1.152156	1.634521	2.337051	2.203391	2.911344	1.439868	2.631643	1.490672	1.182559	2.021195	3.037701	1.95034	1.649888	1.644792	2.281768	1.158856	1.530694	2.057395	1.576581	2.381915	1.528507	2.398191
618.5219	DG 18:1-18	Diacylglyce	C39H70O5	0.568582	0.804194	0.616475	0.717329	0.624884	1.104872	1.122053	0.828048	0.673974	0.766466	1.375449	1.034098	1.193378	0.747257	1.419218	0.940661	0.770506	1.106339	1.866655	1.463041	0.723462	0.960168	1.799795	0.841226	0.772426	0.867322	0.956404	1.560344	1.118313	1.28516
620.5374	DG 18:1-18	Diacylglyce	C39H72O5	0.596022	0.400773	0.505412	0.317219	0.364618	0.714529	1.092511	0.878246	0.349595	0.896256	0.67672	0.488333	0.932833	0.234096	0.608619	0.800151	0.311342	0.53011	1.581088	1.099233	0.396444	0.528222	1.17033	0.444004	0.576048	0.583279	0.386346	1.844524	1.305386	1.735965
622.5526	DG 18:0-18	Diacylglyce	C39H74O5	0.229167	0.202606	0.141206	0.155044	0.118031	0.341158	0.313996	0.238663	0.202822	0.400438	0.294629	0.279855	0.27669	0.203654	0.356867	0.330122	0.214164	0.297534	0.642803	0.509328	0.214427	0.361519	0.435804	0.266652	0.200186	0.248964	0.209191	0.625712	0.501292	0.596318
624.5691	DG 18:0-18	Diacylglyce	C39H76O5	0.760994	0.744116	0.258995	0.538482	0.208052	1.770473	0.979395	0.62713	0.212962	0.57165	0.414669	0.618434	0.364361	0.210953	0.310654	0.883157	0.996102	1.752757	0.766677	0.517626	1.112474	1.132166	0.435786	0.868636	1.294088	1.136401	1.050142	0.581574	0.405493	0.461428
652.5992	DG 14:0-24	Diacylglyce	C41H80O5	0.129408	0.125821	0.099985	0.094514	0.053896	0.251373	0.174561	0.123431	0.070514	0.124721	0.123868	0.121739	0.148843	0.08068	0.152833	0.205123	0.168774	0.289597	0.202581	0.158899	0.15599	0.203386	0.130417	0.175264	0.212246	0.158425	0.144986	0.157472	0.131118	0.156014
668.5397	DG 16:0-24	Diacylglyce	C43H72O5	0.146193	0.194269	0.006916	0.117202	0.245536	0.217811	0.0676	0.050598	0.277043	0.098062	0.820162	0.144848	0.747741	0.445155	0.688968	0.277798	0.172836	0.107633	1.114339	0.966945	0.21238	0.214792	0.791389	0.186579	0.234564	0.274072	0.147537	0.862806	0.844761	1.176653
680.6311	DG 16:0-24	Diacylglyce	C43H84O5	0.131727	0.133387	0.148254	0.131214	0.070036	0.229668	0.200504	0.166222	0.101215	0.122659	0.184441	0.143193	0.131661	0.084093	0.15504	0.205447	0.130547	0.227901	0.199361	0.147562	0.14942	0.206464	0.146589	0.172322	0.148571	0.183413	0.134255	0.215816	0.176496	0.170265
694.6463	DG 16:0-25	Diacylglyce	C44H86O5	0.072363	0.076149	0.085937	0.071922	0.041193	0.123755	0.128411	0.088561	0.059851	0.088486	0.105496	0.087378	0.12215	0.068813	0.106185	0.146019	0.073416	0.117775	0.130302	0.096615	0.088214	0.114894	0.082488	0.113139	0.098829	0.086185	0.058776	0.136413	0.121467	0.120126
704.6312	DG 18:2-24	Diacylglyce	C45H84O5	0.061802	0.096646	0.006916	0.08092	0.081636	0.171603	0.1442	0.109192	0.075599	0.116432	0.185003	0.137542	0.184162	0.08907	0.202002	0.1764	0.153015	0.229133	0.347703	0.242496	0.138401	0.184702	0.267858	0.146494	0.147397	0.143931	0.172337	0.202475	0.181874	0.217398
724.6023	DG 18:0-26	Diacylglyce	C47H80O5	0.398234	0.393905	0.006916	0.221105	0.307257	0.694503	0.191374	0.122579	0.42826	0.280722	1.296661	0.217886	1.098009	0.562018	0.875079	0.489517	0.422088	0.467434	1.372598	1.235687	0.544628	0.527266	0.960447	0.426771	0.617285	0.54816	0.4634	1.203625	1.223033	1.355387
699.5641	Glc-O-t18:	Glycosylcei	C40H77NC	0.143104	0.246781	0.160185	0.202573	0.195001	0.338621	0.306614	0.205669	0.150149	0.191537	0.440787	0.289288	0.271562	0.271485	0.352774	0.245811	0.174111	0.348348	0.441019	0.332715	0.206748	0.25906	0.351228	0.201589	0.194901	0.266775	0.237513	0.338545	0.246748	0.276504
715.5595	Glc d18:1h	Glycosylcei	C40H77NC	0.769258	1.032796	1.105406	0.868828	0.660904	1.594541	1.487402	1.077621	0.704365	0.995582	1.676592	1.287497	1.425202	0.76223	1.4293	1.053751	0.834428	1.528875	1.639687	1.3736	1.325124	1.517956	1.498063	1.095244	1.147323	1.491015	1.215332	1.476912	1.26965	1.480223
731.554	Glc t18:1h1	Glycosylcei	C40H77NC	1.046425	1.41866	0.882612	1.201794	0.945134	2.43849	2.507159	1.726532	1.219988	1.701443	2.369284	1.863978	1.939578	1.084713	2.122431	2.894668	2.03066	3.176931	4.481343	3.602323	2.196971	2.838452	3.458912	1.99481	2.385382	3.135805	3.176545	3.855316	2.73718	3.537105
787.6164	Glc t18:1h2	Glycosylcei	C44H85NC	0.236102	0.382672	0.283418	0.320214	0.244595	0.73568	0.555191	0.407026	0.266328	0.444077	1.103836	0.491419	0.578891	0.36718	0.583863	0.557425	0.387201	0.681653	0.824363	0.654875	0.414941	0.600292	0.609228	0.390838	0.429952	0.494593	0.514222	0.701962	0.48335	0.676501
799.6529	Glc d18:1h	Glycosylcei	C46H89NC	0.051795	0.102612	0.006916	0.096202	0.089324	0.043062	0.057515	0.045639	0.028108	0.04564	0.10317	0.160443	0.197937	0.127547	0.222606	0.14296	0.103054	0.170826	0.211788	0.049386	0.111439	0.146622	0.217058	0.110715	0.13516	0.1839	0.1324	0.186959	0.130282	0.170427
815.6479	Glc t18:1h2	Glycosylcei	C46H89NC	1.477991	2.126353	1.658791	1.850357	1.56115	3.850903	3.51182	2.428027	1.495873	2.1847	5.018371	3.207845	3.732606	2.165307	3.812627	3.236195	2.449986	4.383128	5.132347	3.979436	2.930245	3.507027	4.228395	2.630583	2.817911	3.508668	3.543244	4.027208	3.348628	3.960614
825.6683	Glc d18:2h	Glycosylcei	C48H91NC	0.450139	0.585221	0.471784	0.550343	0.425984	1.076845	0.878743	0.617451	0.375902	0.559298	1.159983	0.763918	0.909702	0.501383	0.805353	0.625046	0.444066	0.883001	0.870565	0.800848	0.608013	0.769611	0.870032	0.508867	0.580244	0.820161	0.712487	0.762786	0.705836	0.903473
839.6484	Glc t18:3h2	Glycosylcei	C48H89NC	0.114619	0.147078	0.094061	0.146908	0.099655	0.317224	0.275395	0.24046	0.138529	0.176897	0.45293	0.208747	0.255548	0.157865	0.298236	0.295112	0.196983	0.363822	0.363606	0.361931	0.207377	0.332394	0.373805	0.208084	0.24741	0.299461	0.324291	0.398359	0.315478	0.358142
841.6634	Glc t18:2h2	Glycosylcei	C48H91NC	5.25335	8.277379	4.678307	8.258439	6.121676	14.91722	14.25152	10.13837	6.203889	8.573216	15.5013	10.60149	11.9276	7.065868	12.46869	10.56509	8.536382	13.51501	19.61172	15.66543	9.4281	12.03252	15.62586	9.63993	10.13829	13.02607	12.25066	16.65922	12.41207	14.40486
843.6789	Glc t18:1h2	Glycosylcei	C48H93NC	7.915114	10.94528	8.502352	8.850843	7.184199	14.35536	16.48559	12.29894	7.534189	10.7912	17.00063	11.93366	12.6314	7.45995	12.75127	15.56271	11.93959	19.88173	24.47765	17.83446	15.57327	20.30499	20.80047	12.70747	15.86156	17.97139	17.46218	22.08176	18.77728	16.88364
853.6998	Glc d18:2h	Glycosylcei	C50H95NC	0.054974	0.073997	0.006916	0.052858	0.046657	0.003593	0.090448	0.002407	0.001374	0.047695	0.003272	0.082286	0.088465	0.037201	0.09475	0.057023	0.059377	0.101342	0.116821	0.118647	0.075567	0.003314	0.083044	0.002162	0.074325	0.087917	0.06862	0.107177	0.079088	0.077746
857.6942	Glc t18:1h2	Glycosylcei	C49H95NC	0.116572	0.181652	0.091019	0.15191	0.138951	0.389473	0.364348	0.302253	0.163076	0.284959	0.431922	0.26753	0.277322	0.123527	0.259183	0.280516	0.218128	0.415255	0.434244	0.340462	0.27996	0.317515	0.386688	0.267726	0.212017	0.314906	0.289287	0.31309	0.25456	0.306432
869.6943	Glc t18:2h2	Glycosylcei	C50H95NC	0.917101	1.110851	0.931141	1.089934	0.81339	2.547989	2.364843	1.715932	1.096184	1.592589	2.021947	1.757849	1.743456	0.89553	1.659847	1.620792	1.243414	2.12811	2.898538	2.086101	1.71065	2.386085	2.334223	1.370462	1.464618	1.972354	2.006808	2.303032	1.959336	2.378229
871.7103	Glc t18:1h2	Glycosylcei	C50H97NC	1.738872	3.280382	1.062097	1.909107	1.918558	3.806598	3.966525	2.946753	2.048842	2.768054	4.237053	2.67748	3.028715	2.013052	3.427252	5.641941	2.842454	5.566125	6.135042	4.606261	3.122734	5.26573	5.020578	3.244297	4.778626	4.144101	4.438348	5.275894	3.990919	4.613535
495.3323	LPC 16:0	Lysophospl	C24H50NC	0.121636	0.103176	0.112018	0.056812	0.038579	0.074479	0.120302	0.10514	0.098623	0.061893	0.003272	0.077765	0.003181	0.035702	0.002473	0.151421	0.070456	0.00297	0.221815	0.002651	0.002789	0.003314	0.002533	0.002162	0.002279	0.003242	0.147858	0.002917	0.203885	0.218423
517.3168	LPC 18:3	Lysophospl	C26H48NC	0.109939	0.101352	0.125367	0.099721	0.053118	0.175227	0.170106	0.154538	0.16196	0.127481	0.165799	0.147749	0.175049	0.092115	0.160911	0.139455	0.081817	0.107101	0.216295	0.173724	0.112854	0.104592	0.128451	0.089331	0.094224	0.092581	0.346035	0.354296	0.127641	0.105475
519.3322	LPC 18:2	Lysophospl	C26H50NC	0.154562	0.146284	0.209964	0.131522	0.073738	0.183091	0.234074	0.186265	0.205643	0.171897	0.227257	0.202248	0.208266	0.104198	0.212917	0.230408	0.107796	0.140022	0.274969	0.25399	0.187628	0.212654	0.222702	0.161918	0.14899	0.204422	0.46423	0.59477	0.306315	0.194557
521.3478	LPC 18:1	Lysophospl	C26H52NC	0.053563	0.04409	0.050723	0.044677	0.030455	0.003593	0.003319	0.045847	0.033275	0.026097	0.003272	0.038903	0.003181	0.021327	0.002473	0.085595	0.047765	0.00297	0.115142	0.002651	0.063274	0.082729	0.102521	0.068328	0.057029	0.003242	0.167792	0.198269	0.103341	0.002964

720.4932	PG 16:0-16	Phosphatid	C38H73O1	0.037304	0.001896	0.073043	0.077816	0.106154	0.003593	0.003319	0.078074	0.109584	0.060092	0.003272	0.002385	0.003181	0.04222	0.002473	0.132523	0.068706	0.00297	0.002949	0.002651	0.002789	0.089324	0.002533	0.08387	0.075693	0.10575	0.127415	0.151297	0.128875	0.07683
722.5105	PG 16:0-16	Phosphatid	C38H75O1	0.080669	0.088421	0.072929	0.076781	0.088453	0.100383	0.003319	0.07247	0.105706	0.070958	0.073664	0.063796	0.042806	0.065678	0.051483	0.131375	0.080392	0.066263	0.09547	0.061004	0.065768	0.003314	0.063186	0.056163	0.074751	0.08425	0.08629	0.09028	0.089586	0.106612
742.4776	PG 16:1-18	Phosphatid	C40H71O1	1.506513	1.76653	2.087968	2.120506	2.551855	2.010679	2.743219	2.481239	5.867222	2.225259	2.391138	2.294706	1.004636	1.19402	2.005398	4.581285	1.996969	1.102443	2.383402	2.310954	1.533605	2.779155	1.781317	2.306125	2.287003	2.855998	4.070849	3.884451	3.219189	3.876571
744.4934	PG 16:1-18	Phosphatid	C40H73O1	0.163658	0.199861	0.281941	0.272402	0.282855	0.20898	0.274341	0.2312	0.508312	0.241049	0.270349	0.288957	0.134712	0.203074	0.226935	0.773917	0.333364	0.20319	0.334852	0.338939	0.231478	0.388856	0.272194	0.345651	0.411057	0.526266	0.675496	0.615556	0.573262	0.544699
744.4936	PG 16:0-18	Phosphatid	C40H73O1	0.621108	0.568123	0.893948	0.837559	0.895355	0.906486	1.049437	0.98321	2.582836	0.898339	1.325311	1.19433	0.490984	0.697718	0.864251	1.475825	0.758216	0.369393	0.912686	0.706926	0.451645	0.810918	0.571041	0.662744	0.71427	0.752986	0.891533	0.870779	0.833659	0.978884
746.5097	PG 16:0-18	Phosphatid	C40H75O1	0.124768	0.117461	0.212804	0.200912	0.229152	0.121513	0.182467	0.187871	0.384926	0.183894	0.245902	0.244855	0.103304	0.182882	0.141071	0.441779	0.23841	0.113676	0.194178	0.155563	0.127231	0.222045	0.128438	0.210845	0.240389	0.291033	0.358676	0.320323	0.315796	0.272816
748.5248	PG 16:0-18	Phosphatid	C40H77O1	0.160087	0.150823	0.273923	0.289072	0.330828	0.182881	0.252096	0.26569	0.535112	0.286524	0.297256	0.376902	0.19961	0.367494	0.255742	0.665537	0.390865	0.183466	0.276637	0.232137	0.160981	0.343691	0.28859	0.359026	0.389853	0.374717	0.459468	0.49475	0.457773	0.341308
834.5274	PiI 18:2-16:	Phosphatid	C43H79O1	0.037711	0.001896	0.050648	0.001661	0.042754	0.072758	0.048008	0.037561	0.001374	0.026749	0.068472	0.032095	0.027097	0.023499	0.042814	0.002131	0.047176	0.031629	0.146132	0.068986	0.03832	0.003314	0.06342	0.002162	0.002279	0.003242	0.00239	0.079027	0.058614	0.002964
749.5024	PC 16:3-18	Phosphoch	C42H72NC	0.048632	0.08482	0.059886	0.067149	0.058971	0.169916	0.203842	0.140873	0.113242	0.122282	0.154938	0.133889	0.118364	0.067087	0.188561	0.157048	0.098131	0.113551	0.187119	0.193283	0.11661	0.154141	0.152482	0.093937	0.073758	0.184448	0.154766	0.187668	0.115244	0.226173
755.5458	PC 16:0-18	Phosphoch	C42H78NC	1.992132	2.746779	2.405223	2.296433	1.673978	4.504597	5.379861	3.765232	3.946409	3.204478	3.808061	3.436662	1.864135	1.396374	3.783673	4.965081	2.915445	3.081293	4.158735	3.532402	2.530218	3.280051	2.813292	2.090012	1.795953	5.391002	4.625504	4.722719	3.807466	6.212148
757.5614	PC 16:0-18	Phosphoch	C42H80NC	2.978409	3.721828	3.86956	2.874362	2.27239	5.199784	6.055866	4.03549	4.835234	3.795006	4.256242	4.396024	2.462733	1.929795	4.242425	5.619177	2.897255	3.386814	3.781094	3.50608	3.313245	3.68684	3.709714	2.569569	2.38402	5.928486	5.333166	5.540551	4.795244	6.363493
759.577	PC 16:0-18	Phosphoch	C42H82NC	1.379949	1.409704	1.475623	1.142195	0.863601	1.032346	1.425065	0.925276	1.087043	0.97476	1.156769	1.142856	0.57801	0.463927	1.052076	1.799928	1.185164	1.163538	1.333265	0.969804	1.161931	1.10186	1.702781	0.931037	0.859696	1.722644	1.636989	1.47627	1.393251	1.754707
767.5539	PC 17:1-18	Phosphoch	C43H78NC	0.630496	1.305396	0.777093	1.081433	0.921606	2.247019	1.949865	1.400366	1.007902	1.394176	2.951836	1.625594	2.151845	1.794085	3.076907	2.472313	1.423416	2.702436	3.302793	4.164847	1.699579	2.422912	5.14118	1.621288	1.700932	2.211485	2.034893	3.166385	1.824472	2.809312
769.5666	PC 17:1-18	Phosphoch	C43H80NC	0.118387	0.153694	0.105969	0.001661	0.125961	0.297954	0.321996	0.22234	0.172814	0.002014	0.003272	0.215436	0.233544	0.159947	0.328272	0.237656	0.131316	0.193476	0.224703	0.169895	0.15833	0.169126	0.295771	0.133017	0.119257	0.248401	0.00239	0.256202	0.19404	0.364025
777.5303	PC 18:3-18	Phosphoch	C44H76NC	1.546981	2.302683	2.069782	1.950152	1.456114	4.681549	5.230485	3.943282	3.38155	3.242972	3.196317	2.786253	2.253946	1.589463	3.74332	4.195766	2.511604	2.71933	4.23411	4.178474	2.48149	3.141979	2.847674	1.885287	1.556771	4.61725	4.035095	4.671406	3.360655	7.037782
779.5437	PC 18:0-18	Phosphoch	C44H78NC	0.469999	0.536284	0.594057	0.419709	0.330813	0.769561	0.985419	0.661175	0.79286	0.566054	0.602704	0.694269	0.329394	0.234337	0.604365	0.839076	0.406814	0.45829	0.536593	0.485733	0.496864	0.540021	0.495325	0.366908	0.351236	1.014732	0.868508	0.8074	0.766527	0.987657
779.5458	PC 18:2-18	Phosphoch	C44H78NC	2.054411	3.148479	2.705814	2.577465	1.96164	5.573354	6.125538	4.413316	4.732701	3.919883	3.728104	3.387775	2.12638	1.745341	4.17251	5.430707	2.78774	3.126654	4.255059	4.439917	2.875876	3.54681	3.41349	2.115088	1.842635	5.813533	4.629256	5.340104	3.934171	7.167642
781.5613	PC 18:2-18	Phosphoch	C44H80NC	0.983971	1.068883	1.359745	1.005393	0.647955	1.853061	2.071097	1.438487	1.234833	1.273911	1.097885	1.198086	0.797449	0.56428	1.226219	1.399021	0.817885	1.134099	1.309135	1.259933	1.23352	1.139196	1.1118	0.841795	0.870129	2.154974	1.712124	1.727394	1.511347	2.003497
783.5775	PC 18:1-18	Phosphoch	C44H82NC	0.675261	0.7895	0.71623	0.671521	0.481183	0.902938	1.001018	0.719815	0.723344	0.605666	0.681088	0.654279	0.370954	0.303373	0.631834	0.930727	0.532617	0.614264	0.745422	0.606394	0.595896	0.665873	0.933045	0.468775	0.441838	1.029609	0.909388	1.011491	0.820956	1.099292
785.5929	PC 18:1-18	Phosphoch	C44H84NC	0.728232	0.913839	0.446573	0.761524	0.55765	1.132144	1.397895	0.888651	1.028301	0.784203	1.008921	1.045364	0.515308	0.421041	0.9881	1.059366	0.523281	0.715551	0.873373	0.69072	0.704439	0.81201	0.95759	0.63826	0.592964	1.157233	1.17167	1.407398	1.066892	1.320595
687.4834	PE 16:1-16:	Phosphoetl	C37H70NC	0.075852	0.032163	0.923774	0.460728	0.916932	0.039588	0.003319	0.477471	0.001374	0.856594	0.003272	0.002385	0.326317	0.78057	0.063321	0.002131	0.360687	0.170234	0.092587	0.09585	0.002789	0.113173	0.086267	0.002162	0.606893	0.003242	0.00239	0.002917	0.002729	0.002964
699.4831	PE 15:0-18:	Phosphoetl	C38H70NC	0.066052	0.118576	0.088582	0.09188	0.1015	0.085867	0.143206	0.096933	0.123134	0.051352	0.126165	0.083599	0.095518	0.102832	0.162187	0.281638	0.204402	0.141746	0.384527	0.259436	0.107519	0.238	0.236177	0.140159	0.266551	0.134839	0.386403	0.377827	0.424083	0.122611
710.5585	PE 15:0-18:	Phosphoetl	C38H67D7	1	1	1	1	1	1	1	1	1	1	1	1	1	1	1	1	1	1	1	1	1	1	1	1	1	1	1	1	1	1
711.4832	PE 14:0-20:	Phosphoetl	C39H70NC	0.104056	0.044923	0.516743	0.376785	0.668189	0.060273	0.06468	0.33719	0.060834	0.547715	0.003272	0.022675	0.302826	0.533157	0.088766	0.02831	0.336727	0.131153	0.108931	0.130386	0.084094	0.128223	0.100819	0.002162	0.477485	0.11587	0.049989	0.002917	0.052754	0.002964
713.499	PE 16:0-18:	Phosphoetl	C39H72NC	0.797812	1.059356	1.361518	1.091918	1.254405	0.950284	1.280246	1.660333	1.542744	1.237122	1.071005	1.223926	0.930768	0.891979	1.072167	1.343488	1.159064	1.18027	1.103994	0.96115	0.751798	0.89245	1.082343	0.818691	0.974715	0.920325	1.140093	0.824232	1.04648	1.105515
715.5151	PE 16:0-18:	Phosphoetl	C39H74NC	0.365874	0.484296	0.5836	0.458305	0.633306	0.428452	0.54227	0.650548	0.663113	0.583965	0.537734	0.507881	0.315373	0.406872	0.4595	0.563322	0.521886	0.43316	0.417505	0.41424	0.288967	0.28701	0.398542	0.335151	0.356698	0.432653	0.495557	0.377805	0.443632	0.444869
739.5148	PE 18:2-18:	Phosphoetl	C41H74NC	0.460251	0.624936	0.740029	0.482283	0.543558	0.611193	0.64889	0.856848	0.710905	0.507491	0.475894	0.580717	0.327154	0.283355	0.515351	0.556581	0.472338	0.385474	0.508814	0.372074	0.460718	0.37666	0.582081	0.344508	0.261164	0.558024	0.476835	0.187987	0.526192	0.382618
741.5328	PE 18:1-18:	Phosphoetl	C41H76NC	0.002167	0.162494	0.13537	0.155511	0.157694	0.003593	0.113836	0.140165	0.128092	0.107286	0.003272	0.002385	0.09824	0.061813	0.113285	0.002131	0.134667	0.113327	0.158689	0.14728	0.002789	0.003314	0.176217	0.091253	0.101678	0.003242	0.128741	0.002917	0.002729	0.002964
813.6261	PE 18:2-23:	Phosphoetl	C46H88NC	0.076235	0.11402	0.095061	0.091427	0.06923	0.311382	0.165746	0.107105	0.071602	0.128868	0.120307	0.134244	0.128562	0.084817	0.120064	0.095077	0.083647	0.181699	0.141746	0.143122	0.112678	0.110828	0.114225	0.066191	0.099898	0.114462	0.109339	0.121749	0.116498	0.125934
827.6395	PE 18:2-24:	Phosphoetl	C47H90NC	0.032068	0.056553	0.103561	0.098346	0.133335	0.043671	0.064807	0.078088	0.086851	0.109391	0.050678	0.051614	0.042799	0.078009	0.044147	0.054092	0.100675	0.073674	0.059398	0.058482	0.03943	0.047558	0.069855	0.040182	0.100345	0.042601	0.055006	0.044698	0.058235	0.059679
245.2358	Sphinganin	Sphingosin	C14H31NC	6.141095	3.037201	4.711534	0.929225	0.90894	9.424516	14.9396	4.057179	0.001374	0.002014	22.79166	1.323032	19.72934	0.137564	12.3263	0.625571	6.830983	9.002637	0.002949	0.002651	0.667444	6.952159	6.443129	16.72525	0.982587	0.589014	6.783743	0.002917	0.002729	0.002964
259.2511	Sphinganin	Sphingosin	C15H33NC	0.082973	0.039759	0.132796	0.013435	0.024925	0.141059	0.173997	0.09494	0.001374	0.002014	0.538008	0.037339	0.369966	0.001596	0.170284	0.026643	0.11258	0.100468	0.002949	0.002651	0.002789	0.040725	0.085428	0.276684	0.002279	0.003242	0.135807	0.002917	0.002729	0.002964
261.2303	Sphingosin	Sphingosin	C14H31NC	2.046381	1.041093	0.921008	0.318486	0.33126	1.33053	2.1425	2.335906	0.001374	0.002014	7.806147	0.165536	6.38322	0.02831	1.891007	0.053873	1.000852	1.565247	0.002949	0.002651	0.145939	1.49894	1.60637	3.54359	0.572861	0.15952	3.879168	0.002917	0.002729	0.002964
271.2512	Sphinganin	Sphingosin	C16H33NC	0.245209	0.257063	0.084749	0.001661	0.078868	0.677085	0.781817	0.529768	0.001374	0.002014	0.864185	0.002385	0.786781	0.001596	0.586135	0.002131	0.499359	0.310787	0.002949	0.002651	0.002789	0.003314	0.455552	0.585461	0.002279	0.003242	0.492196	0.002917	0.002729	0.002964
273.2669	Sphinganin	Sphingosin	C16H35NC	3.280679	2.33539	8.46459	0.435143	2.542478	9.18514	5.373697	12.16363	0.036262	0.002014	53.47482	4.609883	25.34003	0.505733	10.62825	3.248891	4.812856	3.118723	0.156012	0.059726	3.42578	0.927641	3.281324	19.09844	0.924762	0.291134	7.584023	0.148901	0.07053	0.142243
289.2615	Sphingosin	Sphingosin	C16H35NC	0.309181	0.09179	0.516881	0.032191	0.252322	0.397666	0.336725	2.450095	0.001374	0.002014	4.095307	0.236008	2.176646	0.044522	0.30661	0.115509	0.278565	0.237753	0.002949	0.002651	0.147491	0.124702	0.304739	1.359384	0.157057	0.139651	1.736169	0.002917	0.002729	0.002964
299.2814	Sphingosin	Sphingosin	C18H37NC	0.05925	0.001896	0.006916	0.001661	0.001221	0.003593	0.003319	0.002407	0.001374	0.002014	0.003272	0.002385	0.003181	0.001596	0.002473	0.066675	0.063937	0.091447	0.088041	0.002651	0.002789	0.003314	0.002533	0.083808	0.002279	0.003242	0.00239	0.002917	0.002729	0.002964
301.298	Sphinganin	Sphingosin	C18H39NC	0.041241	0.077726	0.30722	0.001661	0.116352	0.121265	0.003319	0.571732	0.001374	0.002014	3.076876	0.370743	0.627345	0.001596	0.616497	0.139058	0.038041	0.00297	0.002949	0.002651	0.290084	0.003314	0.002533	0.277431	0.002279	0.003242	0.112669	0.002917	0.002729	0.002964
317.2932	Sphingosin	Sphingosin	C18H39NC	0.092817	0.001896	0.051798	0.001661	0.039611	0.050964	0.003319	0.282579	0.001374	0.002014	0.703286	0.072213	0.171019	0.001596	0.002473	0.061353	0.061691	0.00297	0.002949	0.002651	0.07186	0.003314	0.002533	0.002162	0.002279	0.087567	0.00239	0.002917	0.002729	0.002964
788.4748	SQDG 16:0	Sulfoquino	C41H72O1	0.002167	0.056382	0.006916	0.024301	0.029141	0.073536	0.066296	0.036359	0.06166	0.039675	0.058792	0.035729	0.038492	0.001596	0.04747	0.082431	0.03134	0.043983	0.079457	0.048996	0.032841	0.086676	0.066814	0.028211	0.019171	0.045866	0.101593	0.121899	0.082371	0.067587
792.5049	SQDG 16:0	Sulfoquino	C41H76O1	0.002167	0.063857	0.006916	0.001661	0.001221	0.003593	0.003319	0.002407	0.066401	0.002014	0.003272	0.002385	0.003181	0.001596	0.002473	0.133621	0.0016	0.00297	0.081336	0.002651	0.002789	0.140827	0.002533	0.002162	0.002279	0.003242	0.124471	0.118268	0.144372	0.002964
794.5203	SQDG 16:0	Sulfoquino	C41H78O1	0.135763	0.300301	0.198492	0.141601	0.152029	0.214102	0.15781	0.092089	0.278355	0.114866	0.185816	0.128152	0.069486	0.058534	0.139302	0.415449	0.170267	0.193985	0.254406	0.183507	0.15837	0.421896	0.215091	0.168764	0.124718	0.219548	0.677132	0.592008	0.582052	0.27858
816.5049	SQDG 16:0	Sulfoquino	C43H76O1	1.432822	3.640905	1.970008	1.688785	1.807808	3.792798	3.153995	2.045326	3.691253	1.829859	3.641764	2.322476	1.883271	1.278422	2.908144	5.933147	1.923778	2.484966	3.952961	2.817804	2.178799	6.015455	3.79881	1.853213	1.637556	1.917261	5.867686	6.202451	5.191094	3.525394
818.5207	SQDG 16:0	Sulfoquino	C43H78O1	0.211882	0.521548	0.28061	0.252471	0.286318	0.566542	0.431655	0.253334	0.543634	0.256626	0.574184	0.382237	0.341817	0.198307	0.458741	1.136094	0.424876	0.634849	0.841159	0.622208	0.355219	1.016662	0.652125	0.349284	0.346145	0.430368	1.230803	1.27304	1.085323	0.562819

820.5362	SQDG 16:0	Sulfoquino	C43H80O1	0.108425	0.247924	0.146116	0.130087	0.119595	0.251813	0.168851	0.093259	0.270379	0.10838	0.226598	0.179608	0.115011	0.094336	0.177367	0.548793	0.17077	0.242745	0.323304	0.186431	0.172419	0.452977	0.38308	0.183687	0.153447	0.209587	0.661772	0.672567	0.607929	0.292616
838.4892	SQDG 18:3	Sulfoquino	C45H74O1	0.388606	0.818693	0.554451	0.429255	0.457696	1.274935	0.941528	0.666667	0.822883	0.624515	1.350022	0.721121	0.747238	0.481742	1.136841	1.559368	0.584921	0.849593	1.574125	1.240425	0.846231	1.851773	1.326831	0.639197	0.548785	0.617776	1.447574	1.573633	1.384437	1.420885
840.5047	SQDG 18:2	Sulfoquino	C45H76O1	0.002167	0.305042	0.006916	0.001661	0.001221	0.003593	0.003319	0.002407	0.274215	0.002014	0.429031	0.002385	0.003181	0.001596	0.002473	0.593833	0.251347	0.51021	0.583646	0.403487	0.002789	0.712826	0.431237	0.002162	0.140337	0.003242	0.5476	0.570359	0.478325	0.474713
842.5205	SQDG 18:1	Sulfoquino	C45H78O1	0.054655	0.130041	0.083988	0.07217	0.084074	0.137215	0.110965	0.060781	0.119725	0.070865	0.156643	0.103468	0.075413	0.050784	0.12362	0.221868	0.078007	0.109309	0.133563	0.097978	0.080582	0.18372	0.125736	0.063428	0.057219	0.06831	0.196142	0.221061	0.167863	0.129586
772.6573	TG 14:1-16	Triacylglyce	C49H88O6	0.394415	0.258432	0.190524	0.223904	0.137615	0.498141	0.667416	0.374624	0.263141	0.284674	0.382344	0.336789	0.511015	0.384142	0.543359	0.790025	0.360033	0.548545	0.646944	0.432692	0.441997	0.63768	0.466031	0.656948	0.382037	0.366383	0.178163	0.61014	0.513143	0.469266
774.6729	TG 14:1-16	Triacylglyce	C49H90O6	1.707001	0.862538	0.755474	0.934979	0.530917	1.89711	2.697777	1.711155	0.992982	1.180503	1.44662	1.36138	2.184671	1.492679	2.270233	3.118765	1.522968	2.158953	2.830705	1.765211	1.921936	2.688468	1.935002	2.902115	1.595253	1.361541	0.766209	2.665315	2.17688	2.045517
776.6886	TG 14:0-16	Triacylglyce	C49H92O6	3.124467	1.668135	1.333744	1.600679	0.942313	3.898052	5.090643	3.006444	1.944612	2.19243	2.389131	2.265357	3.538832	2.439061	3.805266	6.027713	2.678498	4.043418	4.659389	2.850151	3.397922	4.586391	2.944855	5.445806	2.720955	2.523712	1.297188	4.596457	3.890529	3.697618
778.704	TG 14:0-16	Triacylglyce	C49H94O6	2.641594	1.956105	1.739345	1.561474	0.961884	3.759594	3.705183	2.787422	1.510773	2.050883	2.130504	2.060982	2.582522	1.87287	2.809245	4.409412	1.984418	3.129157	3.586459	2.265305	2.528847	3.368074	2.090397	3.8667	2.178831	2.198599	1.296818	3.780965	3.292223	3.293115
786.6731	TG 15:1-16	Triacylglyce	C50H90O6	0.500232	0.351436	0.165769	0.41791	0.22273	0.733185	0.893557	0.551484	0.348078	0.402034	0.503068	0.337743	0.976271	0.577048	0.753466	1.03987	0.399362	0.883951	1.016165	0.49804	0.838027	0.86511	0.730737	1.064754	0.513428	0.444669	0.248146	1.014061	0.726306	0.504101
788.6886	TG 15:1-16	Triacylglyce	C50H92O6	1.509992	0.841905	0.671982	0.862592	0.477969	2.038484	2.416709	1.557613	0.896565	1.08877	1.266721	1.191109	2.00306	1.328739	2.089545	2.901759	1.354751	2.162886	2.5046	1.585985	1.692937	2.39582	1.751532	2.573739	1.383573	1.257642	0.688606	2.339674	1.948466	1.80681
790.7042	TG 15:0-16	Triacylglyce	C50H94O6	2.842647	1.715618	1.157892	1.61005	0.881288	3.617301	4.19589	2.688199	1.576007	1.853442	2.080093	2.065601	3.188337	2.185529	3.209867	5.222247	2.247513	3.745917	4.356271	2.545078	2.81804	4.202895	2.737141	4.569383	2.326195	2.115228	1.09884	3.948291	3.280231	3.230332
792.7196	TG 15:0-16	Triacylglyce	C50H96O6	2.33122	1.666417	1.341527	1.290753	0.807686	3.245865	3.001497	2.182187	1.228683	1.643942	1.85643	1.715256	2.180049	1.611864	2.341884	4.121837	1.702847	2.82534	3.228995	1.927202	2.09613	3.139683	1.879148	3.196769	1.826349	1.884993	1.086096	3.088028	2.702231	2.927834
796.6568	TG 16:1-16	Triacylglyce	C51H88O6	0.034696	0.001896	0.006916	0.001661	0.001221	0.003593	0.003319	0.002407	0.022138	0.002014	0.003272	0.002385	0.184859	0.016705	0.034063	0.047933	0.0016	0.00297	0.033085	0.002651	0.002789	0.036828	0.002533	0.002162	0.018192	0.003242	0.00239	0.002917	0.002729	0.029607
798.6728	TG 14:1-16	Triacylglyce	C51H90O6	0.098559	0.062112	0.052567	0.068975	0.038725	0.131049	0.187286	0.119425	0.07491	0.08304	0.111226	0.089545	0.173218	0.104891	0.167764	0.223189	0.094692	0.141749	0.199284	0.127037	0.114467	0.178547	0.127928	0.175561	0.096246	0.100186	0.056189	0.17522	0.147762	0.118456
800.6885	TG 14:1-16	Triacylglyce	C51H92O6	0.855975	0.4305	0.474124	0.444416	0.267139	0.912108	1.32608	0.819963	0.502565	0.604437	0.728072	0.608195	1.063855	0.68981	1.015435	1.441463	0.732769	1.126479	1.289964	0.834173	0.8975	1.243927	0.914447	1.394896	0.783405	0.683814	0.360192	1.194642	1.011935	0.917726
802.7043	TG 16:0-16	Triacylglyce	C51H94O6	3.953448	2.084811	1.638713	2.252141	1.321146	5.02926	6.795911	3.710722	2.562735	2.844895	3.09556	3.035671	4.988767	3.244498	4.983293	7.782103	3.477312	5.333384	5.785567	3.785353	4.567721	6.398141	4.163295	7.097904	3.704609	3.280206	1.609965	5.914288	5.037034	4.843589
804.7198	TG 16:0-16	Triacylglyce	C51H96O6	5.196833	3.192351	2.03636	2.900389	1.6812	7.236318	7.60075	5.156463	3.037496	3.683498	4.076812	3.711212	5.758332	4.096103	6.054157	9.934162	4.1274	6.685633	7.475578	4.277295	5.406748	8.108702	4.863996	8.684732	4.402848	4.315256	2.09992	7.251763	6.273636	6.071348
806.7351	TG 16:0-16	Triacylglyce	C51H98O6	1.082426	0.740899	1.369037	0.723971	0.385803	1.244513	1.400698	1.179476	0.650783	0.815053	1.040954	0.815032	1.004429	0.627521	0.99717	1.184818	0.583083	0.956613	1.131235	0.815582	0.967044	1.056443	0.805626	1.18432	0.872228	0.935722	0.514894	1.287361	1.171037	1.115543
806.7352	TG 14:0-16	Triacylglyce	C51H98O6	3.762078	2.93438	2.435919	2.283904	1.597137	5.779985	4.777798	3.780903	2.146736	2.901092	3.15824	2.823428	3.565978	2.492019	3.417101	6.89372	2.630353	4.641693	4.954927	3.26498	3.562848	5.470765	3.112237	4.980217	3.158888	3.37513	1.67621	5.145643	4.212511	4.791374
814.7041	TG 15:0-16	Triacylglyce	C52H94O6	0.713952	0.379079	0.342682	0.425546	0.243865	0.937476	1.157562	0.763775	0.432609	0.542538	0.579348	0.573739	0.906846	0.636518	0.963197	1.351419	0.657017	0.99235	1.185445	0.722508	0.841892	1.156563	0.811747	1.191386	0.689092	0.606382	0.329066	1.106112	0.891986	0.842789
816.7196	TG 15:0-16	Triacylglyce	C52H96O6	2.366375	1.296043	0.891888	1.323544	0.796013	2.966309	3.341028	2.315795	1.39479	1.558181	1.773472	1.849146	2.799052	1.845371	2.85262	4.608366	1.981896	3.185558	3.643284	2.286331	2.527761	3.765144	2.51192	3.846413	2.124385	1.932203	0.954666	3.417563	2.828434	2.628239
818.7354	TG 16:0-16	Triacylglyce	C52H98O6	2.962033	1.710699	1.129497	1.503971	1.002886	3.995435	4.052699	2.7355	1.582673	1.900263	2.120125	2.14042	3.11045	2.277364	3.362191	5.784711	2.362898	3.701848	4.111029	2.450797	2.732994	4.555792	2.899899	4.642318	2.369385	2.206023	1.140602	3.978843	3.287483	3.48044
820.7509	TG 16:0-16	Triacylglyce	C52H100O	1.720474	1.251822	1.041979	1.009892	0.625117	2.345404	2.003437	1.503523	0.849647	1.263868	1.28867	1.179307	1.426379	1.181452	1.495845	3.020554	1.17691	2.021639	2.157352	1.330581	1.480769	2.344212	1.285105	2.105844	1.331933	1.324227	0.764411	1.998469	1.839472	1.880299
822.6729	TG 16:0-16	Triacylglyce	C53H90O6	0.002167	0.068493	0.058834	0.105522	0.058393	0.190499	0.149135	0.144915	0.108768	0.074962	0.280054	0.242453	0.325673	0.122592	0.263044	0.162093	0.12691	0.251245	0.344337	0.175879	0.168038	0.172121	0.160335	0.111322	0.124099	0.156823	0.187081	0.25524	0.213955	0.25168
826.704	TG 16:0-16	Triacylglyce	C53H94O6	0.317362	0.205451	0.177599	0.207979	0.134867	0.414492	0.560873	0.384741	0.248355	0.285472	0.43724	0.359966	0.501891	0.315526	0.486958	0.59789	0.317062	0.506705	0.699796	0.394743	0.368342	0.575747	0.428183	0.571149	0.35448	0.34829	0.239961	0.640296	0.465828	0.494006
828.7197	TG 16:0-16	Triacylglyce	C53H96O6	1.962136	1.038543	0.856851	1.147511	0.707869	2.515635	3.353036	1.891684	1.403817	1.47343	1.952401	1.848535	2.680207	1.599283	2.713006	3.774214	1.850993	3.007862	3.294928	1.920828	2.240472	3.193675	2.089979	3.452376	1.881628	1.828032	1.011539	3.242471	2.60355	2.484178
830.7355	TG 16:0-16	Triacylglyce	C53H98O6	4.131824	2.437075	1.636962	2.431037	1.528979	6.3119	6.50968	4.505397	2.713145	3.321655	3.782845	3.629448	5.279359	3.617456	5.619792	8.374995	3.65008	5.991081	6.511371	3.967477	4.579301	7.387532	4.314161	7.280467	3.675874	3.91558	1.950827	6.647179	5.282605	5.273443
832.751	TG 16:0-16	Triacylglyce	C53H100O	4.160427	2.717552	1.487291	2.259428	1.465914	6.066694	5.324074	4.04483	2.552334	3.312626	3.02786	3.120249	3.994037	2.979243	4.120037	8.148982	3.048495	5.160086	5.811581	3.807076	4.210696	6.308652	3.814857	5.979634	3.263163	3.23441	1.605374	6.109799	5.217979	5.170469
834.7665	TG 16:0-16	Triacylglyce	C53H102O	2.364343	1.92239	1.130366	1.430792	0.933291	3.605466	2.478956	1.849033	1.193905	1.734119	1.756584	1.660862	1.884349	1.407831	1.833947	3.496921	1.640538	2.737683	3.017636	1.789205	2.004118	3.599837	1.826969	2.367696	1.819702	2.436535	1.167664	2.89567	2.682338	2.662881
836.6796	TG 15:0-18	Triacylglyce	C54H92O6	0.002167	0.001896	0.036388	0.044021	0.032891	0.122977	0.139232	0.085962	0.03646	0.071585	0.003272	0.069356	0.095249	0.060519	0.118575	0.086775	0.084743	0.115188	0.162437	0.067208	0.043498	0.003314	0.054282	0.092474	0.002279	0.003242	0.00239	0.002917	0.092086	0.102582
840.7194	TG 15:1-18	Triacylglyce	C54H96O6	0.181104	0.102238	0.071222	0.101339	0.070128	0.257787	0.29849	0.207764	0.128995	0.150362	0.217736	0.193417	0.267746	0.181477	0.254605	0.370115	0.167672	0.287548	0.388728	0.220538	0.215106	0.3168	0.272653	0.321506	0.176931	0.181018	0.106658	0.314215	0.276153	0.262199
842.7351	TG 16:0-17	Triacylglyce	C54H98O6	0.760441	0.404145	0.322003	0.428145	0.260493	0.93324	1.150577	0.740743	0.457664	0.538722	0.65799	0.627536	0.908602	0.616123	1.000232	1.45406	0.645285	1.048919	1.264244	0.729838	0.817266	1.194111	0.870481	1.251513	0.672899	0.623998	0.345559	1.146013	0.908293	0.906739
844.6572	TG 16:3-18	Triacylglyce	C55H88O6	0.119918	0.114881	0.204867	0.167596	0.128098	0.292228	0.276585	0.169431	0.215364	0.194479	0.373514	0.259693	0.363592	0.171861	0.429777	0.326487	0.336461	0.461197	0.757509	0.378792	0.591925	0.378915	0.257678	0.285991	0.237563	0.312465	0.445941	0.516429	0.473245	0.545681
844.7508	TG 16:1-17	Triacylglyce	C54H100O	1.396268	0.896943	0.566934	0.799812	0.518284	2.091399	2.152634	1.488233	0.828393	1.00978	1.143356	1.08092	1.569046	1.184513	1.813606	2.925592	1.261866	2.056821	2.109072	1.343722	1.45974	2.459426	1.508719	2.396238	1.310122	1.230913	0.616599	2.16415	1.839273	1.881995
846.7665	TG 16:0-17	Triacylglyce	C54H102O	1.367877	0.853859	0.554236	0.727951	0.453266	1.974468	1.727862	1.161367	0.697176	0.950202	0.947603	0.864937	1.207713	0.99454	1.341479	2.711069	0.981482	1.609847	1.734651	1.086099	1.15967	2.077369	1.129042	1.852608	1.064497	1.048889	0.535455	1.632177	1.453767	1.399793
848.6884	TG 16:1-18	Triacylglyce	C55H92O6	0.260824	0.325504	0.261432	0.339841	0.254984	0.703315	0.648497	0.442005	0.346371	0.416698	0.685219	0.572451	0.675951	0.358536	0.79116	0.741854	0.565592	0.847195	1.220518	0.920678	0.632986	0.748805	0.554648	0.500521	0.5396	0.776279	0.846643	1.042724	0.772279	0.949521
848.7821	TG 16:0-17	Triacylglyce	C54H104O	0.627022	0.521246	0.354139	0.427245	0.264951	0.949872	0.773549	0.509437	0.310907	0.47734	0.47038	0.445111	0.499883	0.412228	0.530692	1.060489	0.466709	0.836235	0.818643	0.445803	0.550604	0.923064	0.475292	0.744838	0.456521	0.604413	0.297338	0.783973	0.652161	0.745174
850.7039	TG 16:0-18	Triacylglyce	C55H94O6	0.539912	0.668283	0.764225	0.778175	0.60732	1.984489	1.695933	1.221216	0.942737	1.028756	2.251288	1.681183	2.162062	1.037379	2.066778	1.4242	1.213217	1.81246	2.856109	1.622986	1.226121	1.564491	1.018214	1.11265	1.193758	1.497269	1.742264	2.169614	1.787055	2.165203
852.7196	TG 16:0-18	Triacylglyce	C55H96O6	1.256411	1.540351	1.264243	1.765931	1.330698	3.895225	3.715099	2.58693	2.000241	2.474447	4.872081	3.685892	4.787837	2.457329	4.910808	3.171906	2.694364	3.918909	5.826367	3.348773	2.273574	2.982919	2.133585	2.399165	2.371472	3.140646	3.228464	4.507717	3.569702	4.308679
854.7352	TG 16:0-18	Triacylglyce	C55H98O6	1.670397	1.672524	1.106066	1.381442	1.238553	3.277581	3.711886	2.498745	1.898808	2.28478	3.514113	2.934582	4.09494	2.302122	3.893377	3.216082	2.055591	3.078527	5.238856	3.028745	2.156321	2.888829	4.279097	2.945396	1.98234	2.126658	1.905529	4.093695	3.162248	3.594666
856.7508	TG 16:0-18	Triacylglyce	C55H100O	1.543323	1.122888	0.857363	0.8794	0.717666	2.073689	2.356568	1.733863	1.194832	1.524788	1.496969	1.674646	1.939271	1.277044	1.868579	2.41014	1.293601	2.066949	2.72296	1.789792	1.506167	2.128245	1.677319	2.194763	1.322417	1.287746	0.87716	2.705122	2.167383	2.068793
858.7667	TG 16:0-18	Triacylglyce	C55H102O	3.00064	1.835956	1.136163	1.519332	1.114752	4.173278	3.765884	2.917273	2.29976	3.022202	2.357524	2.563414	2.873029	2.32438	2.965943	5.26143	2.086103	3.892887	5.378683	3.446173	2.782704	4.215602	2.835542	3.906178	2.291468	2.152548	1.352443	5.781505	4.38291	4.146973
860.7821	TG 16:0-18	Triacylglyce	C55H104O	1.944733	1.318235	0.714085	1.001051	0.662403	2.676054	2.297912	1.518969	1.270533	1.969051	1.194328	1.284994	1.627614	1.233678	1.693574	3.113446	1.235947	2.144943	2.81377	1.948113	1.582815	2.760826	1.471335	2.284743	1.270582	1.526457	0.756862	2.877487	2.720769	2.491695
862.7979	TG 16:0-18	Triacylglyce	C55H106O	1.657465	1.491044	0.657706	1.339699	0.625152	3.00894	1.703684	1.277377	0.753959	1.345832	1.097238	1.117311	0.963141	0.802686	1.000225	2.407192	1.462293	2.277527	1.796035	1.066555	1.891544	2.865308	1.169859	1.556164	1.584753	1.93416	1.255822	1.663601	1.612212	1.550744

870.7664	TG 17:1-18	Triacyglyce	C56H102O	0.384664	0.267419	0.141241	0.192979	0.138692	0.517792	0.595017	0.38998	0.233306	0.294511	0.329573	0.34945	0.448957	0.339424	0.460483	0.77516	0.330395	0.533981	0.611206	0.392123	0.410429	0.649883	0.427963	0.604454	0.328341	0.348841	0.173083	0.577268	0.511139	0.498098
872.6882	TG 14:3-18	Triacyglyce	C57H92O6	1.235605	1.695173	1.418119	1.974109	1.520207	3.793153	3.345032	2.587472	1.881502	2.109057	4.231074	2.976369	4.083103	1.784805	4.27834	4.314851	3.513663	4.316644	7.130841	4.752717	3.142672	4.305609	2.711765	2.81244	3.247048	4.461892	5.13449	6.177916	4.815707	5.504582
874.7041	TG 18:2-18	Triacyglyce	C57H94O6	3.628704	5.120182	3.926989	5.635643	4.576643	11.61185	10.1027	7.041828	5.121122	6.332723	11.72616	8.911115	12.03196	5.425431	12.93789	10.06367	8.006074	10.97978	15.18297	11.4694	7.751141	10.22534	6.451232	6.410647	7.415264	11.00432	12.30641	14.57739	10.63204	14.00046
876.7194	TG 18:2-18	Triacyglyce	C57H96O6	0.988224	1.221084	1.571522	1.275079	0.902131	2.32206	2.242111	1.5409	1.048927	1.466393	2.156085	1.738203	2.436813	1.079491	2.224366	1.47131	1.166272	1.713979	2.423547	1.724727	1.331465	1.638925	1.121832	1.137314	1.305052	1.879249	1.882005	2.30486	1.902628	2.031043
876.7196	TG 18:1-18	Triacyglyce	C57H96O6	1.828344	2.647771	1.96088	2.83963	2.155357	5.11974	4.798587	3.492506	2.615149	3.240832	5.827094	4.819559	5.882742	2.928481	6.947964	4.354998	3.533791	4.480685	7.662849	5.174521	3.413166	4.064308	3.152024	2.880051	3.285492	4.562137	4.87711	6.926425	5.040801	5.933063
878.735	TG 18:2-18	Triacyglyce	C57H98O6	0.65509	0.944581	0.864243	0.646247	0.54993	1.094278	1.035469	0.843925	0.587889	0.971779	1.295977	0.977488	1.443405	0.684569	1.256762	0.74734	0.603024	0.946636	1.566505	1.165371	0.7112	0.716722	2.51816	0.75647	0.643957	0.714667	0.84848	1.223727	0.997968	1.062841
878.7351	TG 18:0-18	Triacyglyce	C57H98O6	1.302572	2.006716	1.135064	1.448925	1.316227	2.162984	2.466618	1.898118	1.42289	2.127094	3.059462	2.391254	3.716217	1.758654	3.416968	2.147739	1.744196	2.354849	5.129557	3.376587	1.75023	1.892853	8.225667	2.135832	1.68314	1.698522	2.089762	3.638773	2.795044	2.851889
880.7505	TG 18:1-18	Triacyglyce	C57H100O	1.355091	1.928664	0.742748	0.890553	1.064577	1.858386	2.039605	1.585697	1.212116	1.813876	2.258768	1.872766	2.9802	1.423126	2.285017	2.128664	1.272312	2.047802	5.146263	3.284595	1.409327	1.657212	3.15907	2.072306	1.314435	1.031464	1.095894	3.078304	2.390479	2.249707
882.7662	TG 18:1-18	Triacyglyce	C57H102O	1.46507	1.599371	0.684868	0.814346	0.972405	2.037933	2.000189	1.606542	1.126086	1.719886	1.746135	1.807062	2.309329	1.226129	1.953522	2.38076	1.243611	2.10773	3.891351	2.580007	1.292559	1.635898	1.827942	1.91168	1.314964	0.905226	0.925705	2.866478	2.167774	1.996921
884.7817	TG 18:1-18	Triacyglyce	C57H104O	0.667442	0.571355	0.609146	0.333392	0.31762	0.827304	0.875465	0.711403	0.522783	0.595477	0.556065	0.560782	0.647568	0.432628	0.672655	0.709949	0.369345	0.742319	1.129988	0.812647	0.461664	0.614402	0.562408	0.613622	0.473992	0.405718	0.36265	1.120459	0.809945	0.84755
884.782	TG 18:0-18	Triacyglyce	C57H104O	1.992029	1.874493	0.846695	0.881765	1.116907	2.703399	2.525896	1.939498	1.535474	1.944827	1.592676	1.756897	1.961077	1.459567	2.191449	3.256063	1.396958	2.88113	4.841228	3.085989	1.409704	2.441966	1.788111	2.289204	1.529422	1.212754	1.080659	4.330727	2.657236	2.890584
886.7973	TG 18:0-18	Triacyglyce	C57H106O	1.036878	0.922525	0.424795	0.584857	0.546279	1.401751	1.286051	0.934342	0.66573	1.040857	0.863327	0.863188	1.052033	0.808809	0.976082	1.708932	0.736303	1.373694	1.961493	1.233021	0.842026	1.40165	0.897699	1.315778	0.768644	0.805387	0.506119	1.817608	1.306785	1.324132
890.8166	TG 16:0-18	Triacyglyce	C57H110O	0.359944	0.307913	0.243615	0.260039	0.146814	0.54844	0.476556	0.353763	0.21301	0.307539	0.336624	0.302758	0.316605	0.24583	0.318076	0.541638	0.31148	0.431155	0.493943	0.324269	0.460808	0.534235	0.315028	0.425788	0.36085	0.437028	0.276967	0.50674	0.45813	0.450275
890.8288	TG 14:0-16	Triacyglyce	C57H110O	1.496127	1.261728	0.526066	2.350215	0.482189	2.87988	1.623289	0.946785	0.421672	1.213136	0.581148	0.940522	0.565193	0.485913	0.603223	1.958787	1.409042	2.469329	1.400106	0.714082	1.706363	2.56165	0.829564	1.369575	1.421474	1.722886	1.378399	1.2617	0.870092	1.060374
898.797	TG 18:1-18	Triacyglyce	C58H106O	0.126485	0.097338	0.040998	0.071273	0.047215	0.20881	0.182281	0.140383	0.080525	0.095964	0.102916	0.103015	0.145733	0.101499	0.111973	0.311037	0.103427	0.195703	0.186679	0.144637	0.119086	0.216107	0.142824	0.185277	0.117573	0.113071	0.069585	0.214317	0.156439	0.13214
900.8127	TG 15:1-16	Triacyglyce	C58H108O	0.270619	0.20416	0.112558	0.1537	0.107099	0.398516	0.387349	0.233816	0.139554	0.215003	0.208497	0.186865	0.252836	0.225317	0.290017	0.52228	0.218086	0.401294	0.391696	0.217248	0.295614	0.414572	0.263693	0.407936	0.206398	0.24171	0.123633	0.378603	0.2825	0.315017
904.7495	TG 18:2-18	Triacyglyce	C59H100O	0.173434	1.188231	0.128589	0.213267	0.374412	0.199873	0.211107	0.158235	0.113425	0.140924	0.251847	0.302298	0.316172	0.524079	0.27662	0.250523	0.4554	0.273341	1.243303	0.376905	0.167115	0.18364	0.193908	0.147274	0.149228	0.214671	0.242038	0.270474	0.208355	0.289614
904.8441	TG 15:0-16	Triacyglyce	C58H112O	0.474957	0.377285	0.252806	0.357718	0.221078	0.651156	0.586735	0.430894	0.221897	0.357707	0.31628	0.346483	0.361636	0.331603	0.374437	0.799786	0.383174	0.545454	0.654069	0.359389	0.443843	0.671857	0.386689	0.574377	0.331114	0.478658	0.237096	0.552063	0.470145	0.526882
906.7652	TG 18:3-18	Triacyglyce	C59H102O	0.314709	1.826794	0.154919	0.285327	0.788008	0.238504	0.211489	0.002407	0.11523	0.14769	0.275088	0.373566	0.454599	0.85691	0.332292	0.393681	0.737074	0.455628	2.210354	0.65307	0.190245	0.186049	0.35517	0.155571	0.150013	0.173363	0.379305	0.259665	0.181811	0.480122
908.7798	TG 18:2-18	Triacyglyce	C59H104O	0.385229	1.80962	0.161782	0.306832	0.91742	0.248135	0.241993	0.183844	0.123607	0.160554	0.260176	0.40335	0.543326	0.909012	0.305736	0.400389	0.841348	0.445784	2.272738	0.700493	0.183873	0.200596	0.227583	0.203575	0.145196	0.169397	0.340585	0.306365	0.207251	0.492372
910.7971	TG 18:1-18	Triacyglyce	C59H106O	0.274528	1.061635	0.115362	0.215752	0.613192	0.32572	0.278265	0.212359	0.138513	0.193016	0.267704	0.30346	0.378837	0.545923	0.293127	0.432123	0.545202	0.410772	1.693922	0.474302	0.229237	0.280279	0.233549	0.260354	0.149068	0.174925	0.233906	0.273308	0.229888	0.395793
912.8126	TG 18:1-18	Triacyglyce	C59H108O	0.28818	0.914624	0.118683	0.21964	0.560272	0.391092	0.319738	0.220453	0.134321	0.203831	0.197944	0.28258	0.326966	0.460643	0.272622	0.506552	0.452016	0.390762	1.426354	0.456226	0.253741	0.396895	0.261327	0.310483	0.187907	0.209838	0.190729	0.367265	0.29404	0.442855
914.8164	TG 18:0-18	Triacyglyce	C59H110O	0.207083	0.310601	0.149598	0.12907	0.193904	0.268959	0.276906	0.17542	0.133421	0.196248	0.173915	0.195104	0.270942	0.249436	0.215366	0.291629	0.192689	0.264506	0.617989	0.311664	0.198937	0.269139	0.180068	0.283689	0.171102	0.195941	0.123862	0.345671	0.278	0.288892
914.8284	TG 16:1-18	Triacyglyce	C59H110O	0.567473	0.550044	0.194892	0.322559	0.350959	0.703698	0.645691	0.456939	0.278854	0.407974	0.394129	0.404618	0.506302	0.497349	0.540496	1.036249	0.441663	0.715854	0.999467	0.47962	0.564534	0.815796	0.485337	0.723968	0.40967	0.471607	0.225349	0.675511	0.554296	0.602818
916.844	TG 16:0-16	Triacyglyce	C59H112O	0.904762	0.664092	0.296431	0.584062	0.341022	1.16509	1.115781	0.679376	0.389843	0.689833	0.485804	0.656933	0.648822	0.682564	0.778246	1.716997	0.658733	1.230419	1.347425	0.647661	0.848814	1.277598	0.707847	1.179842	0.63498	0.830437	0.370392	1.142363	0.937573	0.941715
918.8594	TG 16:0-16	Triacyglyce	C59H114O	0.48721	0.422272	0.223632	0.519119	0.243618	0.686915	0.578627	0.381928	0.201575	0.385524	0.296081	0.347158	0.315756	0.304966	0.359638	0.74463	0.410036	0.570258	0.699391	0.348601	0.456898	0.790918	0.441148	0.559395	0.30607	0.529582	0.266967	0.626637	0.510175	0.543101
928.8437	TG 16:1-17	Triacyglyce	C60H112O	0.336124	0.249142	0.127322	0.171947	0.129212	0.438535	0.451978	0.274437	0.153206	0.259159	0.223302	0.23162	0.297673	0.256981	0.319402	0.574106	0.257294	0.430542	0.47254	0.256532	0.336847	0.471669	0.283083	0.454438	0.242834	0.315788	0.154653	0.390915	0.335403	0.31379
930.8594	TG 16:1-18	Triacyglyce	C60H114O	0.608634	0.458573	0.24786	0.395912	0.241255	0.846452	0.843063	0.547736	0.285735	0.461646	0.403	0.47024	0.513983	0.491778	0.595412	1.104495	0.516996	0.781943	0.954188	0.41918	0.63004	0.871227	0.543416	0.826239	0.476738	0.576613	0.267003	0.788439	0.635143	0.645572
932.781	TG 18:3-18	Triacyglyce	C61H104O	0.029394	0.247177	0.006916	0.043936	0.069766	0.003593	0.003319	0.002407	0.001374	0.002014	0.045474	0.056359	0.003181	0.117155	0.002473	0.002131	0.086961	0.00297	0.226034	0.099185	0.002789	0.035205	0.002533	0.002162	0.002279	0.043405	0.056945	0.002917	0.002729	0.0575
932.8749	TG 15:0-16	Triacyglyce	C60H116O	0.31382	0.261511	0.16389	0.23617	0.148894	0.439181	0.379086	0.263464	0.152749	0.262772	0.218038	0.234991	0.247543	0.212883	0.263397	0.559472	0.274294	0.41364	0.493081	0.287207	0.314273	0.506769	0.292578	0.385666	0.230768	0.355594	0.188705	0.447823	0.337254	0.370608
934.7956	TG 18:2-18	Triacyglyce	C61H106O	0.111387	0.551002	0.040592	0.077192	0.19029	0.093054	0.071586	0.002407	0.001374	0.036811	0.079842	0.108219	0.142302	0.245685	0.084536	0.114752	0.200539	0.14267	0.533356	0.178631	0.059472	0.056167	0.002533	0.042632	0.05726	0.070669	0.094568	0.139674	0.084472	0.118036
936.8108	TG 18:3-20	Triacyglyce	C61H108O	0.173628	0.826385	0.006916	0.136536	0.310561	0.003593	0.094715	0.08366	0.048411	0.069088	0.098513	0.160315	0.174658	0.372121	0.131437	0.180145	0.317955	0.198832	0.959239	0.267524	0.084869	0.11591	0.096179	0.002162	0.002279	0.003242	0.13834	0.126609	0.081407	0.218678
938.828	TG 18:2-20	Triacyglyce	C61H110O	0.002167	0.763146	0.056189	0.125253	0.34963	0.19178	0.176741	0.107973	0.084138	0.133804	0.148195	0.171528	0.244278	0.001596	0.149492	0.197974	0.356047	0.245028	0.002949	0.002651	0.114024	0.175443	0.156104	0.146063	0.089283	0.105129	0.13413	0.19626	0.143654	0.002964
940.8433	TG 18:2-20	Triacyglyce	C61H112O	0.21771	0.517296	0.091592	0.135581	0.254402	0.272023	0.266868	0.178261	0.101066	0.175052	0.165247	0.184777	0.242389	0.26357	0.207899	0.352388	0.225088	0.279897	0.714275	0.25936	0.204115	0.284935	0.202541	0.259256	0.138484	0.165526	0.130673	0.263599	0.215987	0.27828
942.8589	TG 16:1-18	Triacyglyce	C61H114O	0.461887	0.39774	0.157945	0.305509	0.21343	0.617301	0.623929	0.362205	0.236436	0.371713	0.335397	0.390161	0.4102	0.382126	0.410948	0.85682	0.380286	0.673556	0.872019	0.378684	0.467516	0.650954	0.408394	0.666567	0.353691	0.466477	0.2258	0.628903	0.566812	0.521803
944.8746	TG 16:0-18	Triacyglyce	C61H116O	0.558573	0.412734	0.192706	0.376628	0.236196	0.706389	0.666539	0.395685	0.248608	0.404439	0.32898	0.392743	0.397202	0.426311	0.499893	0.931262	0.435386	0.660604	0.830717	0.405171	0.507099	0.789933	0.4933	0.717701	0.368827	0.553881	0.234476	0.725223	0.583419	0.634785
946.8903	TG 16:0-18	Triacyglyce	C61H118O	0.189775	0.165175	0.090803	0.158241	0.101702	0.259642	0.237285	0.175061	0.084078	0.164327	0.113365	0.135369	0.117226	0.107436	0.142376	0.298519	0.156365	0.252621	0.283203	0.145224	0.178711	0.30914	0.186897	0.201609	0.124933	0.222787	0.109837	0.266688	0.211992	0.213959
956.8744	TG 16:1-18	Triacyglyce	C62H116O	0.253883	0.168715	0.120358	0.189939	0.101257	0.343957	0.357879	0.234758	0.121905	0.206795	0.16261	0.207262	0.240994	0.210056	0.254904	0.444913	0.212161	0.314806	0.370689	0.205259	0.267483	0.391965	0.231838	0.350309	0.201372	0.257294	0.112862	0.320769	0.253185	0.261399
958.8899	TG 16:0-18	Triacyglyce	C62H118O	0.308606	0.218057	0.117575	0.188324	0.110852	0.378081	0.35871	0.25228	0.131034	0.216201	0.164829	0.212941	0.234744	0.203488	0.267545	0.503998	0.224576	0.353765	0.416417	0.235406	0.275526	0.400085	0.252482	0.379356	0.19904	0.289286	0.13016	0.372559	0.288486	0.308896
960.906	TG 16:0-17	Triacyglyce	C62H120O	0.096739	0.089482	0.052832	0.083156	0.055198	0.144789	0.108726	0.090515	0.046409	0.098112	0.063876	0.069279	0.067452	0.067849	0.090259	0.149212	0.081952	0.114944	0.156915	0.065622	0.096626	0.144134	0.103158	0.108072	0.068604	0.107044	0.05641	0.144373	0.094456	0.127974
964.8401	TG 18:3-20	Triacyglyce	C63H112O	0.04841	0.168022	0.006916	0.044754	0.064414	0.089632	0.068344	0.002407	0.029246	0.049165	0.053639	0.055793	0.072755	0.094665	0.05636	0.081493	0.080523	0.088334	0.23726	0.07617	0.056723	0.0722	0.002533	0.04822	0.04235	0.054731	0.054902	0.077422	0.055368	0.08585
966.8585	TG 18:2-20	Triacyglyce	C63H114O	0.075248	0.150477	0.006916	0.054276	0.063623	0.104074	0.09433	0.063191	0.043265	0.073973	0.085638	0.08021	0.100536	0.087581	0.091241	0.123956	0.081995	0.113367	0.19011	0.110211	0.074402	0.115635	0.077109	0.090293	0.055401	0.065505	0.053471	0.116084	0.083877	0.104813
968.8738	TG 18:1-18	Triacyglyce	C63H116O	0.130875	0.143229	0.047997	0.077303	0.068797	0.167483	0.182083	0.108791	0.060341	0.103866	0.099465	0.110906	0.133342	0.123302	0.126241	0.218641	0.126417	0.159883	0.264011	0.127766	0.132064	0.191859	0.128942	0.160852	0.093851	0.126116	0.066175	0.154737	0.133107	0.151613
970.8896	TG 16:1-18	Triacyglyce	C63H118O	0.240848	0.200435	0.081419	0.158259	0.113927	0.33742	0.284102	0.197573	0.110109	0.202	0.162764	0.20082	0.20442	0.209053	0.237173	0.419264	0.197803	0.30564	0.383362	0.202489	0.258386	0.342088	0.235793	0.348179	0.186046	0.244251	0.117347	0.327837	0.271659	0.283247

972.9048	TG 16:0-18	Triacyglyce	C63H120O	0.18252	0.149042	0.069746	0.118123	0.076478	0.229393	0.21786	0.164964	0.078654	0.136899	0.090827	0.132141	0.121192	0.126126	0.152418	0.301207	0.140012	0.221635	0.268775	0.138123	0.173211	0.263551	0.164012	0.23134	0.120121	0.203177	0.085298	0.236485	0.205535	0.195685
974.9132	TG 16:0-18	Triacyglyce	C63H122O	0.075049	0.041712	0.006916	0.052476	0.03233	0.074393	0.094579	0.072586	0.041273	0.05528	0.050599	0.064876	0.065895	0.053698	0.074149	0.074054	0.05219	0.077089	0.128078	0.063876	0.066331	0.102097	0.075971	0.092366	0.056671	0.08556	0.00239	0.100307	0.085582	0.090837
982.8894	TG 18:1-18	Triacyglyce	C64H118O	0.070037	0.041866	0.006916	0.032488	0.024969	0.073854	0.069464	0.053104	0.028859	0.044526	0.038692	0.051813	0.056002	0.047323	0.052756	0.107821	0.051838	0.080483	0.095334	0.041867	0.058727	0.093988	0.061837	0.082344	0.048422	0.058613	0.023695	0.078032	0.056211	0.065052

Mass (M)	Name	Compound	FORMULA (	Ler-R1	Ler-R2	Ler-R3	Ler-R4	Ler-R5	arc3-R1	arc3-R2	arc3-R3	arc3-R4	arc3-R5	arc5-R1	arc5-R2	arc5-R3	arc5-R4	arc5-R5	Col-0-R1	Col-0-R2	Col-0-R3	Col-0-R4	Col-0-R5	kac1/2-R1	kac1/2-R2	kac1/2-R3	kac1/2-R4	kac1/2-R5	chup1-R1	chup1-R2	chup1-R3	chup1-R4	chup1-R5
774.45519	Arabidopside A	Arabidopsi	C43H66O1	0.048612	0.040292	0.055173	0.043099	0.023144	0.060663	0.083485	0.064956	0.050341	0.059942	0.080593	0.127768	0.081635	0.052593	0.081614	0.068703	0.044463	0.098902	0.086402	0.052251	0.241174	0.194889	0.24499	0.158533	0.166467	0.293636	0.145723	0.286653	0.177236	0.131633
802.4869	Arabidopside B	Arabidopsi	C45H70O1	0.000393	0.001952	0.000206	0.001023	0.000544	0.00159	0.001868	9.08E-05	4.29E-05	7.84E-05	0.000116	8.25E-05	0.002344	0.001643	0.00295	0.001383	0.000824	0.000937	0.002338	0.000557	0.005172	0.006581	0.008372	0.006007	0.006729	0.00554	0.005052	0.007936	0.004376	9.26E-05
537.51165	Cer d18:1h16:0	Ceramides	C34H67NC	0.0171	0.02473	0.010477	0.016681	0.01471	0.016166	0.013319	0.015327	0.009384	0.014932	0.0303	0.021799	0.019586	0.015072	0.027344	0.013503	0.010603	0.023145	0.025184	0.012549	0.009849	0.016031	0.021525	0.00895	0.010955	0.011757	0.006867	0.018788	0.011495	0.009959
553.50719	Cer d16:0h18:1	Ceramides	C34H67NC	0.018172	0.030759	0.012253	0.023707	0.019399	0.027876	0.026095	0.026094	0.014826	0.027314	0.040353	0.033297	0.029638	0.020177	0.038546	0.030676	0.02455	0.040721	0.056357	0.041138	0.027555	0.03707	0.051402	0.022257	0.024166	0.029969	0.024382	0.043523	0.028168	0.028993
555.52274	Cer d16:0h18:0	Ceramides	C34H69NC	0.014086	0.02239	0.01088	0.017652	0.013853	0.018167	0.016312	0.018154	0.009125	0.016599	0.035782	0.022552	0.022138	0.011985	0.027914	0.016199	0.016859	0.032023	0.035147	0.017912	0.013851	0.016495	0.023027	0.0094	0.010218	0.009831	0.008179	0.01602	0.009928	0.01263
569.50143	Cer t18:1-16:0	Ceramides	C34H67NC	0.016822	0.030319	0.013555	0.025452	0.019778	0.040583	0.040075	0.038195	0.02139	0.03668	0.059054	0.040664	0.037314	0.02175	0.050632	0.036605	0.02398	0.042126	0.053371	0.050463	0.037508	0.047185	0.062238	0.027243	0.029093	0.040209	0.030385	0.056896	0.035431	0.038268
571.51723	Cer t18:0h16:0	Ceramides	C34H69NC	0.014668	0.020908	0.009811	0.020602	0.013297	0.025637	0.027524	0.025651	0.012565	0.025845	0.043696	0.03321	0.031722	0.014362	0.035456	0.010187	0.010122	0.018942	0.020246	0.014174	0.009968	0.013627	0.025369	0.009002	0.009608	0.010232	0.007918	0.018564	0.009169	0.010303
581.53789	Cer d18:0h18:1	Ceramides	C36H71NC	0.003453	0.008579	0.003194	0.006225	0.003646	9.79E-05	0.006247	0.005465	0.002103	0.006611	0.016113	0.005289	0.005211	0.00424	0.007121	0.003378	0.00295	0.005354	0.007975	0.005765	0.003922	0.004847	0.007715	0.002714	0.003481	0.003072	0.00245	0.005463	0.004984	0.003872
609.56915	Cer d18:1h20:0	Ceramides	C38H75NC	0.023798	0.018118	0.024094	0.023024	0.012075	0.025378	0.024192	0.031211	0.010544	0.028394	0.040531	0.031886	0.030271	0.017995	0.026603	0.017458	0.012324	0.037077	0.031934	0.019303	0.021204	0.028561	0.027215	0.0197	0.023261	0.02975	0.0168	0.029547	0.018367	0.015469
625.56414	Cer t18:1h20:0	Ceramides	C38H75NC	0.006501	0.008511	0.004374	0.007401	0.00542	0.012531	0.012254	0.010913	0.006068	0.01011	0.020656	0.012693	0.012279	0.007885	0.015648	0.011954	0.00523	0.012881	0.013456	0.01074	0.008515	0.012422	0.015666	0.008732	0.006703	0.009069	0.005857	0.014595	0.009351	0.010897
651.57974	Cer t18:2h22:0	Ceramides	C40H77NC	0.003858	0.007504	0.004225	0.006645	0.006103	0.013182	0.01036	0.010257	0.005125	0.013075	0.020606	0.011556	0.015412	0.008061	0.014441	0.006784	0.005973	0.011341	0.01173	0.012786	0.007775	0.009667	0.012994	0.004777	0.006476	0.008556	0.006249	0.012437	0.007538	0.009629
651.65241	Cer d18:0h24:0	Ceramides	C42H85NC	0.006504	0.008894	0.004114	0.007788	0.005708	0.009165	0.005944	0.009384	0.005003	0.006667	0.011991	0.008852	0.009542	0.005616	0.010908	0.008869	0.005535	0.009349	0.009715	0.005885	0.007382	0.011855	0.00971	0.006503	0.004611	0.008137	0.004296	0.009472	0.006937	0.007383
653.5954	Cer t18:1h22:0	Ceramides	C40H79NC	0.094807	0.12877	0.071055	0.108084	0.079233	0.175104	0.170666	0.154345	0.081754	0.156538	0.303717	0.185168	0.186522	0.101746	0.206652	0.11974	0.080837	0.177808	0.173447	0.149876	0.16729	0.198765	0.241037	0.104505	0.1093	0.148091	0.107256	0.193443	0.129863	0.132525
655.61099	Cer t18:0h22:0	Ceramides	C40H81NC	0.018789	0.034689	0.010464	0.023027	0.022391	0.035354	0.032686	0.038809	0.020484	0.035826	0.078937	0.044891	0.046974	0.031979	0.05959	0.019954	0.012829	0.030271	0.035143	0.023927	0.024029	0.021559	0.031478	0.012999	0.015042	0.01176	0.010556	0.020903	0.013613	0.013441
663.61587	Cer d18:2h24:0	Ceramides	C42H81NC	0.13322	0.185592	0.124968	0.166788	0.11696	0.155317	0.168951	0.165402	0.083361	0.168162	0.299696	0.204504	0.185127	0.129228	0.221514	0.127208	0.102813	0.150495	0.173625	0.151593	0.155637	0.164265	0.191512	0.105594	0.110238	0.160261	0.106074	0.205603	0.146021	0.14353
665.59501	Cer t18:2h23:0	Ceramides	C41H79NC	0.026666	0.027797	0.028643	0.026055	0.017919	0.045787	0.043932	0.044197	0.020588	0.035781	0.058848	0.050927	0.056747	0.029403	0.044954	0.029685	0.024748	0.043144	0.04258	0.03902	0.042439	0.040598	0.055225	0.03003	0.03195	0.049547	0.032971	0.057241	0.035564	0.034662
665.63156	Cer d18:1h24:0	Ceramides	C42H83NC	0.123625	0.215505	0.083747	0.161865	0.135065	0.201677	0.167833	0.193074	0.100021	0.179298	0.325515	0.197664	0.183558	0.12826	0.257201	0.213181	0.119029	0.18615	0.229284	0.227287	0.169288	0.220236	0.306107	0.135296	0.133001	0.161028	0.137245	0.259084	0.170527	0.182324
667.61087	Cer t18:1h23:0	Ceramides	C41H81NC	0.036262	0.066264	0.018782	0.04396	0.035235	0.063623	0.06613	0.06732	0.042053	0.066554	0.157808	0.085991	0.088575	0.057213	0.110798	0.059674	0.035349	0.069435	0.081307	0.076565	0.07476	0.06941	0.102603	0.047412	0.046027	0.049875	0.037548	0.078879	0.049486	0.050418
669.62645	Cer t18:0h23:0	Ceramides	C41H83NC	0.005531	0.010344	0.000206	0.005902	0.010716	0.01275	0.011914	0.01145	0.006051	0.008714	0.019747	0.009453	0.011882	0.005536	0.01685	0.010041	0.00466	0.013471	0.01266	0.008716	0.009629	0.010446	0.010237	0.006828	6.84E-05	0.007362	0.007256	0.013442	0.009224	0.008446
675.57964	Cer t18:1h24:3	Ceramides	C42H77NC	0.001476	0.002091	0.000206	0.002015	0.001573	0.003835	0.002708	0.003128	0.001707	0.002863	0.006735	0.002499	0.002753	0.001024	0.002728	0.003274	0.002189	0.003518	0.004824	0.005701	0.002526	0.003824	0.005584	0.002682	0.002404	0.003892	0.003334	0.007218	0.004108	0.004312
677.63128	Cer d18:2h25:0	Ceramides	C43H83NC	0.010994	0.021597	0.010418	0.018231	0.01328	0.02365	0.022106	0.022956	0.009714	0.020814	0.043278	0.029053	0.028256	0.015881	0.031551	0.018909	0.016914	0.022626	0.029971	0.02312	0.021152	0.022498	0.026233	0.014807	0.016123	0.018967	0.015593	0.024667	0.018014	0.016706
679.6113	Cer t18.2h24.0	Ceramides	C42H81NC	0.297559	0.448978	0.214917	0.391602	0.284236	0.622348	0.597585	0.620747	0.288554	0.53046	0.918271	0.599866	0.574707	0.32356	0.695576	0.405294	0.258769	0.434136	0.692981	0.624226	0.50263	0.58977	0.779096	0.323725	0.362344	0.469112	0.34987	0.680623	0.453746	0.49814
681.62716	Cer t18:1h24:0	Ceramides	C42H83NC	0.145792	0.238379	0.094248	0.195045	0.154099	0.293974	0.273802	0.286228	0.157978	0.260473	0.457062	0.285878	0.295474	0.17007	0.358141	0.258408	0.147052	0.277034	0.32914	0.315867	0.274019	0.33764	0.498637	0.197504	0.200975	0.251016	0.194644	0.383882	0.247896	0.247222
683.64133	Cer t18:0h24:0	Ceramides	C42H85NC	0.026347	0.05613	0.020469	0.043356	0.053867	0.059029	0.054869	0.061047	0.034957	0.05812	0.112616	0.064962	0.063466	0.035529	0.085376	0.035315	0.02356	0.052628	0.066456	0.043041	0.040412	0.046235	0.071887	0.028054	0.026244	0.029459	0.029528	0.057726	0.034148	0.03308
691.64709	Cer d18:2h26:0	Ceramides	C44H85NC	0.043521	0.089175	0.027198	0.055778	0.044765	0.073313	0.071042	0.074367	0.037582	0.077401	0.099082	0.069311	0.067529	0.045569	0.086546	0.081611	0.041641	0.075448	0.093547	0.083606	0.063553	0.085943	0.108868	0.050163	0.066545	0.070945	0.056889	0.111298	0.063353	0.070216
693.62651	Cer t18:1h25:1	Ceramides	C43H83NC	0.063313	0.079039	0.062548	0.063802	0.048133	0.103065	0.120117	0.106091	0.05559	0.101236	0.154586	0.110942	0.115742	0.058544	0.114197	0.062147	0.044054	0.086196	0.099234	0.087939	0.08709	0.122098	0.128034	0.06391	0.060462	0.091301	0.061029	0.11324	0.076645	0.077245
693.6637	Cer d18:1h26:0	Ceramides	C44H87NC	0.048517	0.083802	0.042573	0.075438	0.065997	0.088732	0.085999	0.097167	0.056686	0.083713	0.128046	0.084104	0.070422	0.046518	0.109367	0.10494	0.062388	0.098145	0.128266	0.101314	0.087047	0.098659	0.198874	0.076069	0.060722	0.088723	0.085339	0.155785	0.101347	0.086693
695.64215	Cer t18:1h25:0	Ceramides	C43H85NC	0.011266	0.022346	0.007881	0.0162	0.013593	0.026724	0.023758	0.026837	0.015739	0.022842	0.051097	0.025798	0.024821	0.016706	0.03479	0.022606	0.014198	0.019569	0.030374	0.025214	0.021895	0.02712	0.040329	0.016242	0.016715	0.01676	0.01568	0.032708	0.021777	0.020417
695.67857	Cer d18:0h26:0	Ceramides	C44H89NC	0.095973	0.160911	0.071774	0.127169	0.127569	0.135601	0.123119	0.157167	0.094976	0.130334	0.232216	0.164357	0.126937	0.083835	0.199868	0.103248	0.067207	0.11917	0.100648	0.091986	0.078273	0.097748	0.163403	0.066877	0.050501	0.074429	0.066289	0.116903	0.082506	0.074336
697.65737	Cer t18:0h25:0	Ceramides	C43H87NC	0.004198	0.008174	0.003078	0.007431	0.011799	0.007724	0.007728	0.009211	0.005781	0.009719	0.011318	0.00957	0.010185	0.00437	0.012209	0.007042	0.005741	0.011481	0.013317	0.005416	0.008031	0.010564	0.010707	0.006041	0.004799	0.007095	0.007225	0.011177	0.009827	0.005998
707.64213	Cer t18:1h26:1	Ceramides	C44H85NC	0.07154	0.148741	0.055515	0.10805	0.089144	0.181761	0.185307	0.199999	0.102683	0.200531	0.303673	0.173941	0.174552	0.107658	0.234797	0.141419	0.088705	0.157708	0.217427	0.211543	0.145619	0.173651	0.271036	0.116779	0.117834	0.144844	0.116877	0.232637	0.147861	0.14326
709.65925	Cer t18:1h26:0	Glycosylcei	C44H87NC	0.056548	0.097948	0.037565	0.089331	0.076543	0.144858	0.13968	0.156655	0.088104	0.131806	0.228591	0.141393	0.122148	0.06967	0.167113	0.11368	0.071872	0.117222	0.139683	0.127566	0.113718	0.1386	0.215982	0.084684	0.076201	0.097945	0.08855	0.165462	0.111528	0.098645
711.67423	Cer t18:0h26:0	Ceramides	C44H89NC	0.017372	0.031352	0.014525	0.030458	0.033791	0.036387	0.03924	0.045119	0.028306	0.03704	0.085322	0.053622	0.042793	0.02351	0.069508	0.020226	0.012902	0.020823	0.026326	0.020195	0.021337	0.020883	0.034676	0.015402	0.012519	0.016077	0.012831	0.027193	0.019011	0.017274
646.56836	CE 18:3	Cholestrol	C45H74O2	0.002894	0.004255	0.002103	0.003838	0.00322	0.003828	0.003094	0.002562	0.00138	0.003388	0.00853	0.005194	0.005094	0.003409	0.007098	0.004506	0.003294	0.004835	0.005862	0.004254	0.005922	0.006154	0.007082	0.003866	0.003612	0.004988	0.002355	0.005799	0.003149	0.003801
648.58405	CE 18:2	Cholestrol	C45H76O2	0.005647	0.004748	0.001683	0.003365	0.002653	0.005308	0.004367	0.004325	0.002582	0.003857	0.005989	0.005448	0.004136	0.003486	0.005918	0.008211	0.004083	0.006875	0.005699	0.004116	0.004622	0.007335	0.006972	0.00498	0.003419	0.006411	0.001994	0.005927	0.005114	0.005021
650.59942	CE 18:1	Cholestrol	C45H78O2	0.007225	0.004034	0.00236	0.003365	0.002065	0.006161	0.006127	0.005998	0.002639	0.003957	0.00444	0.004651	0.004444	0.004103	0.005768	0.00795	0.003899	0.006203	0.005161	0.003503	0.005783	0.008118	0.005893	0.006277	0.003888	0.007317	0.002068	0.008164	0.004918	0.006494
536.44391	DG 14:1-16:1	Diacylglyce	C33H60O5	0.003514	6.68E-05	0.000206	5.89E-05	3.95E-05	9.79E-05	0.003106	0.002617	4.29E-05	7.84E-05	0.000116	8.25E-05	9.09E-05	4.85E-05	8.68E-05	0.004441	0.001871	0.002242	0.002841	8.81E-05	0.002403	0.000125	0.000108	0.003065	0.001628	0.000134	6.87E-05	0.003192	0.002175	9.26E-05
538.45928	DG 14:0-16:1	Diacylglyce	C33H62O5	0.004945	0.002936	0.001523	0.003476	0.001004	9.79E-05	0.008015	0.004924	0.002255	0.00411	0.004039	0.002209	0.004512	0.003289	0.005939	0.007137	0.004053	0.006547	0.006835	0.003969	0.005513	0.006102	0.005077	0.005737	0.003494	0.003553	0.001905	0.006539	0.004709	0.004381
540.47521	DG 14:0-16:0	Diacylglyce	C33H64O5	0.005483	0.004764	0.002991	0.003819	0.001858	0.006253	0.008098	0.006488	0.004313	0.00518	0.006198	0.005542	0.005722	0.004781	0.00791	0.008964	0.004272	0.007768	0.010737	0.006402	0.005939	0.009125	0.007739	0.007064	0.004523	0.005829	0.002603	0.009254	0.006216	0.007123
552.47457	DG 15:0-16:1	Diacylglyce	C34H64O5	0.00632	6.68E-05	0.001549	0.002991	3.95E-05	9.79E-05	0.006784	0.004677	0.002709	0.003552	0.000116	0.004368	9.09E-05	0.003438	8.68E-05	0.0081	0.003037	0.007191	0.006933	8.81E-05	0.006793	0.009496	0.000108	0.006106	0.004448	0.000134	6.87E-05	0.007739	0.00556	9.26E-05
554.49037	DG 15:0-16:0	Diacylglyce	C34H66O5	0.004709	0.004071	0.000206	0.003505	0.001759	0.0051	0.006689	0.005538	0.002229	0.004327	0.004723	0.003903	0.004875	0.00346	0.005235	0.006321	0.003651	0.004735	0.006653	0.004572	0.003774	0.006738	0.004622	0.005124	0.003207	0.004051	0.002307	0.007794	0.005814	0.004451
562.45964	DG 16:0-16:3	Diacylglyce	C35H62O5	0.002565	0.004876	0.000206	0.003564	0.002447	0.006138	0.005344	0.005808	0.003464	0.003926	0.007075	0.007182	0.005215	0.004068	0.009056	0.006839	0.006099	0.008497	0.011905	0.008709	0.00517	0.007612	0.008699	0.005331	0.004963	0.012427	0.00786	0.01218	0.007101	0.009574
564.47524	DG 16:1-16:1	Diacylglyce	C35H64O5	0.006597	0.003359	0.002312	0.004589	0.001855	0.005941	0.008668	0.00588	0.002256	0.004234	0.006997	0.004478	0.00631	0.003334	0.009494	0.009157	0.004642	0.006551	0.010512	0.005661	0.006579	0.007576	0.007312	0.006901	0.00385	0.006674	0.001679	0.009543	0.006524	0.006343

566.49079	DG 16:0-16:1	Diacylglyce	C35H66O5	0.018543	0.010533	0.006006	0.010784	0.00572	0.016173	0.02232	0.018599	0.009531	0.01318	0.01313	0.015437	0.017421	0.012192	0.022137	0.029107	0.012017	0.022828	0.023907	0.015434	0.020431	0.028373	0.019441	0.022192	0.011183	0.013657	0.007794	0.026184	0.020166	0.018453
568.50603	DG 16:0-16:0	Diacylglyce	C35H68O5	0.027348	0.021705	0.014275	0.017421	0.014383	0.026028	0.035078	0.024338	0.025026	0.022223	0.049979	0.025381	0.034137	0.019233	0.031726	0.036056	0.019198	0.02853	0.062068	0.03725	0.030796	0.036719	0.051934	0.025978	0.020344	0.029535	0.015078	0.055119	0.042244	0.0465
582.52147	DG 16:0-17:0	Diacylglyce	C36H70O5	0.004422	6.68E-05	0.002728	5.89E-05	3.95E-05	0.003627	0.008175	0.003824	0.002754	7.84E-05	0.000116	8.25E-05	9.09E-05	4.85E-05	8.68E-05	0.007675	5.37E-05	0.000102	1E-04	8.81E-05	0.000117	0.005931	0.000108	0.004544	6.84E-05	0.004058	6.87E-05	0.008004	0.005714	0.00413
584.44385	DG 16:3-18:3	Diacylglyce	C37H60O5	0.006467	0.008742	0.009241	0.010114	0.009111	0.014444	0.010891	0.009707	0.008012	0.009735	0.01984	0.011393	0.01262	0.007148	0.018213	0.010223	0.009537	0.010395	0.022066	0.014543	0.007341	0.007542	0.011096	0.00584	0.004516	0.00987	0.008801	0.015776	0.014263	0.024207
586.45927	DG 16:3-18:2	Diacylglyce	C37H62O5	0.001453	0.002978	0.001247	0.002099	0.002698	0.004248	0.00375	0.003831	0.002402	0.003143	0.007158	0.003991	0.005898	0.002707	0.008597	0.005634	0.003393	0.005333	0.01002	0.006975	0.004762	0.005078	0.006676	0.002995	0.003556	0.0049	0.004485	0.008835	0.005302	0.007736
588.47507	DG 16:1-18:3	Diacylglyce	C37H64O5	0.006536	0.011527	0.006103	0.008677	0.007961	0.014223	0.013286	0.010974	0.007533	0.011823	0.022637	0.018288	0.018829	0.012392	0.033811	0.016497	0.013156	0.020293	0.030931	0.024908	0.013236	0.019929	0.034539	0.013138	0.014139	0.018028	0.01933	0.026803	0.016863	0.021576
590.49035	DG 16:1-18:2	Diacylglyce	C37H66O5	0.006043	0.003789	0.000206	0.004259	0.003003	0.004638	0.004911	0.005554	0.002095	0.004046	0.010769	0.004973	0.009265	0.002646	0.005077	0.007193	0.005532	0.010319	0.015385	0.007075	0.005717	0.006548	0.010102	0.005981	0.006158	0.009713	0.0049	0.01803	0.011003	0.009779
590.49043	DG 16:0-18:3	Diacylglyce	C37H66O5	0.158579	0.237725	0.145683	0.235092	0.180814	0.363083	0.363371	0.352826	0.204875	0.321664	0.548184	0.392293	0.383803	0.236406	0.54541	0.41459	0.321744	0.498997	0.769503	0.571191	0.36877	0.482239	0.661691	0.339127	0.317269	0.606919	0.380516	0.760778	0.447705	0.627053
592.50625	DG 16:1-18:1	Diacylglyce	C37H68O5	0.191132	0.273215	0.164417	0.259107	0.191586	0.39117	0.401676	0.38737	0.210956	0.332549	0.596834	0.458234	0.420397	0.26112	0.603434	0.431884	0.320604	0.499038	0.70313	0.541565	0.354103	0.456388	0.616848	0.313898	0.306938	0.552975	0.383295	0.808178	0.502238	0.541161
592.50635	DG 16:0-18:2	Diacylglyce	C37H68O5	0.136176	0.127377	0.193196	0.135798	0.086138	0.212466	0.235162	0.225717	0.117197	0.201486	0.256324	0.184918	0.168945	0.096819	0.203838	0.161653	0.111653	0.196954	0.199221	0.155305	0.175325	0.19272	0.213223	0.136942	0.141279	0.298299	0.156327	0.317563	0.221203	0.190538
594.52194	DG 16:0-18:1	Diacylglyce	C37H70O5	0.038464	0.036004	0.024012	0.033351	0.023469	0.040909	0.047536	0.048202	0.032327	0.057719	0.050688	0.048143	0.050109	0.026828	0.059603	0.065754	0.040302	0.064257	0.101648	0.066545	0.053065	0.070283	0.111443	0.052288	0.032458	0.058458	0.034918	0.109926	0.086642	0.084965
596.53746	DG 16:0-18:0	Diacylglyce	C37H72O5	0.042901	0.042702	0.015688	0.031113	0.021453	0.062804	0.046365	0.041499	0.025343	0.041629	0.059557	0.041364	0.036111	0.021708	0.04011	0.051394	0.045806	0.071162	0.076605	0.05527	0.065628	0.072156	0.069964	0.044897	0.056353	0.078277	0.051056	0.074662	0.05034	0.054392
604.50606	DG 17:0-18:3	Diacylglyce	C38H68O5	0.002022	0.002517	0.001523	0.002236	0.001362	0.003233	0.003439	0.003278	0.001787	0.002867	0.004922	0.004546	0.003531	0.00184	0.00492	0.004173	0.003343	0.004987	0.007707	0.005475	0.002575	0.003936	0.006331	0.003441	0.002778	0.004326	0.003102	0.007301	0.004548	0.005864
612.47497	DG 18:3-18:3	Diacylglyce	C39H64O5	0.081813	0.08439	0.09817	0.093448	0.065005	0.171158	0.137837	0.15053	0.080343	0.123209	0.208031	0.140302	0.152928	0.075	0.170291	0.129563	0.107392	0.171997	0.264418	0.160865	0.144316	0.135628	0.167934	0.099276	0.103808	0.190133	0.126141	0.229199	0.140867	0.17834
614.49064	DG 18:2-18:3	Diacylglyce	C39H66O5	0.037203	0.056603	0.031578	0.05958	0.044701	0.082984	0.078889	0.08208	0.052043	0.076159	0.142784	0.092479	0.088806	0.057785	0.14528	0.093009	0.067732	0.094789	0.152396	0.116112	0.066375	0.076709	0.12905	0.058512	0.058201	0.108655	0.086815	0.155036	0.092226	0.105782
616.50625	DG 18:2-18:2	Diacylglyce	C39H68O5	0.05014	0.047681	0.091059	0.046402	0.036927	0.066401	0.075886	0.074889	0.036005	0.0636	0.083169	0.076242	0.083181	0.043765	0.092338	0.054804	0.039683	0.069457	0.102973	0.064795	0.069323	0.061834	0.097097	0.037503	0.045967	0.085016	0.045304	0.095277	0.05199	0.074943
618.52191	DG 18:1-18:2	Diacylglyce	C39H70O5	0.020601	0.028317	0.018402	0.025437	0.020223	0.030106	0.031342	0.031247	0.021062	0.029824	0.048948	0.035782	0.034097	0.022713	0.049797	0.034583	0.025856	0.038019	0.063276	0.048606	0.030398	0.036097	0.076587	0.027224	0.023196	0.03584	0.027483	0.062414	0.038038	0.040161
620.53744	DG 18:1-18:1	Diacylglyce	C39H72O5	0.021595	0.014112	0.015087	0.011249	0.0118	0.019469	0.030517	0.033141	0.010925	0.034874	0.024083	0.016897	0.026652	0.007115	0.021355	0.029417	0.010448	0.018217	0.053596	0.036519	0.016657	0.019858	0.049801	0.014369	0.017299	0.024102	0.011102	0.073781	0.044401	0.054249
622.55259	DG 18:0-18:1	Diacylglyce	C39H74O5	0.008303	0.007134	0.004215	0.005498	0.00382	0.009296	0.008771	0.009006	0.006338	0.015581	0.010485	0.009684	0.007905	0.00619	0.012522	0.012137	0.007187	0.010225	0.02179	0.016921	0.00901	0.013591	0.018545	0.00863	0.006012	0.010288	0.006011	0.025028	0.017051	0.018635
624.5691	DG 18:0-18:0	Diacylglyce	C39H76O5	0.027572	0.026201	0.007731	0.019095	0.006733	0.048242	0.027357	0.023665	0.006655	0.022243	0.014757	0.021399	0.01041	0.006412	0.0109	0.032469	0.033426	0.060232	0.025989	0.017197	0.046743	0.042563	0.018544	0.028111	0.038862	0.046959	0.030176	0.023263	0.013792	0.01442
652.59915	DG 14:0-24:0	Diacylglyce	C41H80O5	0.004689	0.00443	0.002985	0.003352	0.001744	0.006849	0.004876	0.004658	0.002204	0.004853	0.004408	0.004212	0.004253	0.002452	0.005363	0.007541	0.005664	0.009952	0.006867	0.005279	0.006554	0.007646	0.00555	0.005672	0.006374	0.006546	0.004166	0.006299	0.00446	0.004875
668.53968	DG 16:0-24:6	Diacylglyce	C43H72O5	0.005297	0.00684	0.000206	0.004156	0.007946	0.005935	0.001888	0.001909	0.008658	0.003816	0.029187	0.005012	0.021364	0.013531	0.024174	0.010213	0.0058	0.003699	0.037774	0.032124	0.008924	0.008075	0.033676	0.006038	0.007044	0.011325	0.00424	0.034512	0.028733	0.03677
680.63113	DG 16:0-24:0	Diacylglyce	C43H84O5	0.004773	0.004697	0.004425	0.004653	0.002267	0.006258	0.005601	0.006273	0.003163	0.004773	0.006564	0.004955	0.003762	0.002556	0.00544	0.007553	0.004381	0.007832	0.006758	0.004902	0.006278	0.007762	0.006238	0.005577	0.004462	0.007579	0.003858	0.008633	0.006003	0.005321
694.64628	DG 16:0-25:0	Diacylglyce	C44H86O5	0.002622	0.002681	0.002565	0.00255	0.001333	0.003372	0.003587	0.003342	0.00187	0.003443	0.003754	0.003023	0.00349	0.002092	0.003726	0.005368	0.002464	0.004047	0.004417	0.00321	0.003706	0.004319	0.00351	0.003661	0.002968	0.003561	0.001689	0.005457	0.004132	0.003754
704.63117	DG 18:2-24:0	Diacylglyce	C45H84O5	0.002239	0.003403	0.000206	0.00287	0.002642	0.004676	0.004028	0.00412	0.002362	0.00453	0.006584	0.004759	0.005262	0.002707	0.007088	0.006485	0.005135	0.007874	0.011787	0.008056	0.005815	0.006944	0.011398	0.004741	0.004426	0.005948	0.004952	0.008099	0.006186	0.006794
724.60225	DG 18:0-26:6	Diacylglyce	C47H80O5	0.014429	0.01387	0.000206	0.007841	0.009944	0.018924	0.005346	0.004626	0.013383	0.010923	0.046145	0.007539	0.031372	0.017083	0.030705	0.017997	0.014164	0.016063	0.046529	0.041053	0.022884	0.019822	0.04087	0.013811	0.018537	0.022651	0.013316	0.048145	0.0416	0.042356
699.56413	Glc-O-t18:1h16:0	Glycosylcer	C40H77NC	0.005185	0.008689	0.004782	0.007183	0.006311	0.009227	0.008565	0.007761	0.004692	0.007453	0.015686	0.01001	0.007759	0.008252	0.012378	0.009037	0.005843	0.011971	0.01495	0.011054	0.008687	0.009739	0.014946	0.006524	0.005853	0.011024	0.006825	0.013542	0.008393	0.008641
715.55953	Glc d18:1h16:0	Glycosylcer	C40H77NC	0.027872	0.036366	0.032997	0.03081	0.021388	0.043448	0.041548	0.040665	0.022011	0.038739	0.059665	0.04455	0.04072	0.023168	0.050151	0.038741	0.028001	0.052539	0.055583	0.045635	0.055677	0.057066	0.063747	0.035445	0.034454	0.061612	0.034923	0.059076	0.043185	0.046257
731.55404	Glc t18:1h16:0	Glycosylcer	C40H77NC	0.037914	0.049953	0.026347	0.042617	0.030587	0.066444	0.070032	0.065152	0.038125	0.066204	0.084316	0.064498	0.055417	0.03297	0.074471	0.106422	0.068143	0.109173	0.15191	0.119679	0.09231	0.106709	0.147188	0.064557	0.071633	0.129579	0.09128	0.154213	0.093101	0.110535
787.61636	Glc t18:1h20:0	Glycosylcer	C44H85NC	0.008554	0.013474	0.00846	0.011355	0.007916	0.020046	0.015508	0.015359	0.008323	0.017279	0.039282	0.017004	0.01654	0.01116	0.020486	0.020494	0.012993	0.023424	0.027945	0.021757	0.017434	0.022567	0.025925	0.012648	0.012911	0.020438	0.014776	0.028078	0.01644	0.021141
799.65293	Glc d18:1h22:0	Glycosylcer	C46H89NC	0.001877	0.003613	0.000206	0.003411	0.002891	0.001173	0.001607	0.001722	0.000878	0.001776	0.003672	0.005552	0.005655	0.003877	0.007811	0.005256	0.003458	0.00587	0.007179	0.001641	0.004682	0.005512	0.009237	0.003583	0.004059	0.007599	0.003805	0.007478	0.004431	0.005326
815.6479	Glc t18:1h22:0	Glycosylcer	C46H89NC	0.05355	0.074872	0.049516	0.065615	0.050523	0.104929	0.098096	0.091624	0.046746	0.085008	0.17859	0.110998	0.106646	0.065815	0.133776	0.118978	0.082214	0.150623	0.173978	0.132207	0.12312	0.131843	0.179932	0.085132	0.084622	0.144986	0.101817	0.161088	0.113899	0.123769
825.66826	Glc d18:2h24:0	Glycosylcer	C48H91NC	0.016309	0.020606	0.014083	0.019516	0.013786	0.029342	0.024546	0.0233	0.011747	0.021763	0.041281	0.026433	0.025991	0.01524	0.028258	0.02298	0.014902	0.030344	0.029511	0.026606	0.025547	0.028933	0.037023	0.016468	0.017425	0.033891	0.020474	0.030511	0.024008	0.028234
839.6484	Glc t18:3h24:0	Glycosylcer	C48H89NC	0.004153	0.005179	0.002808	0.005209	0.003225	0.008644	0.007693	0.009074	0.004329	0.006883	0.016119	0.007223	0.007301	0.004798	0.010464	0.01085	0.00661	0.012502	0.012326	0.012024	0.008713	0.012496	0.015907	0.006734	0.00743	0.012374	0.009319	0.015934	0.010731	0.011192
841.6634	Glc t18:2h24:0	Glycosylcer	C48H91NC	0.190339	0.291457	0.139651	0.292852	0.198113	0.406464	0.398087	0.38258	0.193872	0.333588	0.551648	0.366834	0.340789	0.214768	0.437498	0.388422	0.286456	0.464433	0.664804	0.520446	0.396139	0.452351	0.66493	0.311972	0.304453	0.538267	0.35203	0.666369	0.422179	0.450152
843.67885	Glc t18:1h24:0	Glycosylcer	C48H93NC	0.286779	0.385397	0.253802	0.31386	0.232498	0.391154	0.460491	0.464111	0.235443	0.419891	0.605005	0.412929	0.360897	0.226746	0.447413	0.572159	0.400657	0.683221	0.829751	0.592507	0.654339	0.763346	0.885126	0.411245	0.476323	0.742619	0.501787	0.88327	0.638683	0.527614
853.69978	Glc d18:2h26:0	Glycosylcer	C50H95NC	0.001992	0.002606	0.000206	0.001874	0.00151	9.79E-05	0.002526	9.08E-05	4.29E-05	0.001856	0.000116	0.002847	0.002528	0.001131	0.003325	0.002096	0.001993	0.003483	0.00396	0.003942	0.003175	0.000125	0.003534	0.00007	0.002232	0.003633	0.001972	0.004287	0.00269	0.00243
857.69419	Glc t18:1h25:0	Glycosylcer	C49H95NC	0.004224	0.006396	0.002717	0.005387	0.004497	0.010612	0.010177	0.011406	0.005096	0.011088	0.015371	0.009257	0.007923	0.003755	0.009094	0.010313	0.00732	0.01427	0.01472	0.011311	0.011763	0.011937	0.016455	0.008664	0.006367	0.013013	0.008313	0.012524	0.008658	0.009576
869.69429	Glc t18:2h26:0	Glycosylcer	C50H95NC	0.033228	0.039114	0.027795	0.03865	0.026323	0.069427	0.066057	0.064752	0.034256	0.061968	0.071955	0.060825	0.049813	0.02722	0.05824	0.059588	0.041725	0.073131	0.098256	0.069306	0.071876	0.089702	0.099329	0.044352	0.043983	0.081502	0.057667	0.092121	0.066644	0.07432
871.7103	Glc t18:1h26:0	Glycosylcer	C50H97NC	0.063003	0.115506	0.031704	0.067699	0.062089	0.103722	0.110797	0.111198	0.064026	0.107706	0.150785	0.092646	0.086535	0.061187	0.120254	0.207424	0.095384	0.191276	0.207968	0.153032	0.131207	0.19796	0.213642	0.104993	0.143502	0.171244	0.127539	0.211036	0.135746	0.144173
495.33226	LPC 16:0	Lysophospl	C24H50NC	0.004407	0.003633	0.003344	0.002015	0.001249	0.002029	0.00336	0.003968	0.003082	0.002408	0.000116	0.002691	9.09E-05	0.001085	8.68E-05	0.005567	0.002364	0.000102	0.007519	8.81E-05	0.000117	0.000125	0.000108	0.00007	6.84E-05	0.000134	0.004249	0.000117	0.006935	0.006826
517.31684	LPC 18:3	Lysophospl	C26H48NC	0.003983	0.003569	0.003742	0.003536	0.001719	0.004775	0.004752	0.005832	0.005061	0.00496	0.0059	0.005112	0.005001	0.0028	0.005646	0.005127	0.002746	0.00368	0.007332	0.005772	0.004742	0.003932	0.005466	0.002891	0.00283	0.003826	0.009944	0.014172	0.004342	0.003296
519.33223	LPC 18:2	Lysophospl	C26H50NC	0.0056	0.005151	0.006268	0.004664	0.002386	0.004989	0.006538	0.007029	0.006426	0.006689	0.008087	0.006998	0.00595	0.003167	0.007471	0.008471	0.003617	0.004812	0.009321	0.008438	0.007884	0.007994	0.009477	0.00524	0.004474	0.008447	0.01334	0.023791	0.010419	0.00608
521.34783	LPC 18:1	Lysophospl	C26H52NC	0.001941	0.001552	0.001514	0.001584	0.000986	9.79E-05	9.27E-05	0.00173	0.00104	0.001015	0.000116	0.001346	9.09E-05	0.000648	8.68E-05	0.003147	0.001603	0.000102	0.003903	8.81E-05	0.002659	0.00311	0.004363	0.002211	0.001713	0.000134	0.004822	0.007931	0.003515	9.26E-05

720.4932	PG 16:0-16:1	Phosphatid	C38H73O1	0.001352	6.68E-05	0.00218	0.002759	0.003435	9.79E-05	9.27E-05	0.002946	0.003425	0.002338	0.000116	8.25E-05	9.09E-05	0.001283	8.68E-05	0.004872	0.002306	0.000102	1E-04	8.81E-05	0.000117	0.003358	0.000108	0.002714	0.002273	0.00437	0.003661	0.006052	0.004384	0.002401
722.5105	PG 16:0-16:0	Phosphatid	C38H75O1	0.002923	0.003113	0.002177	0.002723	0.002863	0.002735	9.27E-05	0.002735	0.003303	0.002761	0.002621	0.002207	0.001223	0.001996	0.001806	0.00483	0.002698	0.002277	0.003236	0.002027	0.002763	0.000125	0.002689	0.001818	0.002245	0.003481	0.00248	0.003611	0.003047	0.003332
742.47756	PG 16:1-18:3	Phosphatid	C40H71O1	0.054584	0.062202	0.062327	0.075195	0.082584	0.054787	0.076626	0.093632	0.183351	0.086586	0.085094	0.079402	0.028704	0.036292	0.070365	0.16843	0.067012	0.037885	0.080793	0.076776	0.064437	0.10448	0.075801	0.074632	0.068679	0.118016	0.116978	0.155378	0.109496	0.121143
744.49337	PG 16:1-18:2	Phosphatid	C40H73O1	0.00593	0.007037	0.008416	0.00966	0.009154	0.005694	0.007663	0.008725	0.015885	0.009379	0.009621	0.009999	0.003849	0.006172	0.007963	0.028453	0.011187	0.006982	0.011351	0.01126	0.009726	0.014619	0.011583	0.011186	0.012344	0.021747	0.019411	0.024622	0.019499	0.017022
744.49363	PG 16:0-18:3	Phosphatid	C40H73O1	0.022504	0.020004	0.026685	0.029701	0.028976	0.0247	0.029314	0.037102	0.080714	0.034955	0.047164	0.041326	0.014028	0.021207	0.030325	0.054258	0.025443	0.012694	0.030938	0.023486	0.018977	0.030486	0.0243	0.021448	0.02145	0.031115	0.025619	0.034831	0.028356	0.03059
746.50969	PG 16:0-18:2	Phosphatid	C40H75O1	0.004521	0.004136	0.006352	0.007125	0.007416	0.003311	0.005097	0.007089	0.012029	0.007155	0.008751	0.008472	0.002952	0.005559	0.00495	0.016242	0.008	0.003906	0.006582	0.005168	0.005346	0.008348	0.005465	0.006823	0.007219	0.012026	0.010307	0.012813	0.010741	0.008525
748.52477	PG 16:0-18:1	Phosphatid	C40H77O1	0.0058	0.005311	0.008177	0.010251	0.010706	0.004983	0.007042	0.010026	0.016722	0.011149	0.010579	0.013042	0.005703	0.01117	0.008973	0.024468	0.013116	0.006305	0.009378	0.007712	0.006764	0.012921	0.01228	0.011619	0.011707	0.015484	0.013203	0.01979	0.015571	0.010666
834.52738	PiI 18:2-16:0	Phosphatid	C43H79O1	0.001366	6.68E-05	0.001512	5.89E-05	0.001384	0.001983	0.001341	0.001417	4.29E-05	0.001041	0.002437	0.001111	0.000774	0.000714	0.001502	7.84E-05	0.001583	0.001087	0.004954	0.002292	0.00161	0.000125	0.002699	0.00007	6.84E-05	0.000134	6.87E-05	0.003161	0.001994	9.26E-05
749.50235	PC 16:3-18:3	Phosphoch	C42H72NC	0.001762	0.002987	0.001788	0.002381	0.001908	0.00463	0.005694	0.005316	0.003539	0.004758	0.005514	0.004633	0.003382	0.002039	0.006616	0.005774	0.003293	0.003902	0.006343	0.006421	0.0049	0.005795	0.006489	0.00304	0.002215	0.007622	0.004447	0.007507	0.00392	0.007068
755.54582	PC 16:0-18:3	Phosphoch	C42H78NC	0.072179	0.096718	0.071798	0.081434	0.054174	0.122741	0.150275	0.142084	0.123325	0.124688	0.135518	0.118916	0.053261	0.042443	0.13276	0.18254	0.097834	0.105886	0.140974	0.117356	0.106312	0.12331	0.119715	0.067638	0.053933	0.222769	0.132917	0.188909	0.129506	0.19413
757.56143	PC 16:0-18:2	Phosphoch	C42H80NC	0.107913	0.13105	0.115509	0.101928	0.07354	0.141683	0.169158	0.152283	0.151101	0.147666	0.151468	0.152112	0.070364	0.058656	0.148857	0.206587	0.097223	0.116385	0.128173	0.116481	0.139212	0.138603	0.15786	0.083158	0.071592	0.244979	0.153252	0.221622	0.163104	0.198859
759.57703	PC 16:0-18:1	Phosphoch	C42H82NC	0.049998	0.049637	0.044048	0.040503	0.027948	0.028129	0.039806	0.034916	0.03397	0.037928	0.041166	0.039545	0.016515	0.014101	0.036915	0.066174	0.039771	0.039984	0.045195	0.032219	0.048821	0.041423	0.072459	0.030131	0.025817	0.071184	0.04704	0.059051	0.047389	0.054835
767.55385	PC 17:1-18:3	Phosphoch	C43H78NC	0.022844	0.045965	0.023197	0.038349	0.029825	0.061227	0.054465	0.052844	0.031497	0.054248	0.105048	0.056249	0.061481	0.054531	0.107962	0.090894	0.047766	0.092867	0.111959	0.138367	0.071411	0.091087	0.218774	0.052469	0.051079	0.091384	0.058474	0.126655	0.062057	0.087791
769.56659	PC 17:1-18:2	Phosphoch	C43H80NC	0.004289	0.005412	0.003163	5.89E-05	0.004076	0.008119	0.008994	0.00839	0.0054	7.84E-05	0.000116	0.007455	0.006673	0.004862	0.011518	0.008737	0.004407	0.006649	0.007617	0.005644	0.006653	0.006358	0.012586	0.004305	0.003581	0.010265	6.87E-05	0.010248	0.0066	0.011376
777.53029	PC 18:3-18:3	Phosphoch	C44H76NC	0.05605	0.08108	0.061785	0.069154	0.047123	0.127563	0.146103	0.148803	0.105673	0.126186	0.113748	0.09641	0.064398	0.048312	0.131345	0.154256	0.084282	0.093448	0.143529	0.13882	0.104264	0.11812	0.121178	0.061013	0.04675	0.190795	0.115951	0.186856	0.114308	0.219931
779.54366	PC 18:0-18:5	Phosphoch	C44H78NC	0.017029	0.018883	0.017733	0.014883	0.010706	0.020969	0.027526	0.02495	0.024777	0.022025	0.021449	0.024023	0.009411	0.007123	0.021206	0.030848	0.013651	0.015749	0.01819	0.016137	0.020877	0.020302	0.021078	0.011874	0.010548	0.041931	0.024957	0.032296	0.026072	0.030864
779.54584	PC 18:2-18:3	Phosphoch	C44H78NC	0.074435	0.110862	0.080771	0.091399	0.063484	0.151863	0.171104	0.16654	0.147897	0.152525	0.132673	0.117224	0.060754	0.05305	0.146404	0.199658	0.093548	0.107445	0.144239	0.147506	0.120835	0.133339	0.145255	0.068449	0.055334	0.240229	0.133025	0.213604	0.133815	0.223989
781.56135	PC 18:2-18:2	Phosphoch	C44H80NC	0.035651	0.037637	0.040589	0.035652	0.020969	0.050492	0.057852	0.054283	0.038589	0.049569	0.039071	0.041456	0.022784	0.017151	0.043025	0.051435	0.027446	0.038972	0.044377	0.041858	0.051829	0.042827	0.047311	0.027243	0.02613	0.089049	0.049199	0.069096	0.051406	0.062609
783.57747	PC 18:1-18:2	Phosphoch	C44H82NC	0.024466	0.027799	0.02138	0.023813	0.015572	0.024603	0.027961	0.027163	0.022604	0.023567	0.024238	0.022639	0.010599	0.009221	0.02217	0.034218	0.017873	0.021109	0.025269	0.020146	0.025038	0.025033	0.039704	0.015171	0.013268	0.042546	0.026132	0.04046	0.027924	0.034353
785.59286	PC 18:1-18:1	Phosphoch	C44H84NC	0.026385	0.032177	0.013331	0.027004	0.018047	0.030849	0.039047	0.033534	0.032134	0.030514	0.035905	0.036172	0.014723	0.012798	0.03467	0.038947	0.01756	0.024589	0.029606	0.022948	0.029598	0.030527	0.040749	0.020656	0.017807	0.04782	0.033669	0.056296	0.036289	0.041269
687.48343	PE 16:1-16:1	Phosphoetl	C37H70NC	0.002748	0.001132	0.027575	0.016338	0.029674	0.001079	9.27E-05	0.018018	4.29E-05	0.03333	0.000116	8.25E-05	0.009323	0.023726	0.002222	7.84E-05	0.012104	0.00585	0.003139	0.003184	0.000117	0.004255	0.003671	0.00007	0.018225	0.000134	6.87E-05	0.000117	9.28E-05	9.26E-05
699.48312	PE 15:0-18:3	Phosphoetl	C38H70NC	0.002393	0.004175	0.002644	0.003258	0.003285	0.00234	0.004	0.003658	0.003848	0.001998	0.00449	0.002893	0.002729	0.003126	0.005691	0.010354	0.006859	0.004871	0.013035	0.008619	0.004518	0.008947	0.01005	0.004536	0.008005	0.005572	0.011104	0.015113	0.014425	0.003832
711.48317	PE 14:0-20:4	Phosphoetl	C39H70NC	0.00377	0.001582	0.015425	0.013361	0.021624	0.001642	0.001807	0.012724	0.001901	0.021312	0.000116	0.000785	0.008652	0.016205	0.003115	0.001041	0.0113	0.004507	0.003693	0.004332	0.003533	0.00482	0.00429	0.00007	0.014339	0.004788	0.001436	0.000117	0.001794	9.26E-05
713.49895	PE 16:0-18:3	Phosphoetl	C39H72NC	0.028906	0.037301	0.040642	0.038721	0.040596	0.025893	0.035761	0.062654	0.048211	0.048137	0.038114	0.04235	0.026593	0.027112	0.03762	0.049393	0.038895	0.040559	0.037424	0.031932	0.031588	0.033551	0.046057	0.026495	0.029271	0.03803	0.032761	0.032969	0.035595	0.034547
715.51514	PE 16:0-18:2	Phosphoetl	C39H74NC	0.013256	0.017053	0.017421	0.016252	0.020495	0.011674	0.015147	0.024549	0.020722	0.022722	0.019136	0.017574	0.009011	0.012367	0.016123	0.02071	0.017513	0.014885	0.014153	0.013762	0.012141	0.01079	0.016959	0.010846	0.010712	0.017878	0.01424	0.015112	0.01509	0.013902
739.51479	PE 18:2-18:2	Phosphoetl	C41H74NC	0.016676	0.022005	0.02209	0.017102	0.017591	0.016654	0.018125	0.032334	0.022216	0.019747	0.016936	0.020094	0.009347	0.008613	0.018082	0.020463	0.01585	0.013247	0.017248	0.012361	0.019358	0.01416	0.024769	0.011149	0.007843	0.023059	0.013702	0.007519	0.017898	0.011957
741.53284	PE 18:1-18:2	Phosphoetl	C41H76NC	7.85E-05	0.005722	0.004041	0.005515	0.005103	9.79E-05	0.00318	0.005289	0.004003	0.004175	0.000116	8.25E-05	0.002807	0.001879	0.003975	7.84E-05	0.004519	0.003894	0.005379	0.004893	0.000117	0.000125	0.007499	0.002953	0.003053	0.000134	0.003699	0.000117	9.28E-05	9.26E-05
813.6261	PE 18:2-23:0	Phosphoetl	C46H88NC	0.002762	0.004015	0.002838	0.003242	0.00224	0.008485	0.00463	0.004042	0.002238	0.005014	0.004281	0.004645	0.003673	0.002578	0.004213	0.003495	0.002807	0.006244	0.004805	0.004755	0.004734	0.004166	0.004861	0.002142	0.003	0.00473	0.003142	0.00487	0.003963	0.003935
827.63948	PE 18:2-24:0	Phosphoetl	C47H90NC	0.001162	0.001991	0.003091	0.003487	0.004315	0.00119	0.00181	0.002947	0.002714	0.004256	0.001803	0.001786	0.001223	0.002371	0.001549	0.001989	0.003378	0.002532	0.002014	0.001943	0.001657	0.001788	0.002973	0.0013	0.003013	0.00176	0.001581	0.001788	0.001981	0.001865
245.23581	Sphinganine d14:0	Sphingosin	C14H31NC	0.222503	0.106944	0.140643	0.032951	0.029416	0.256799	0.417307	0.153101	4.29E-05	7.84E-05	0.811091	0.04578	0.563695	0.004181	0.432502	0.022999	0.229228	0.309369	1E-04	8.81E-05	0.028044	0.261359	0.274176	0.54127	0.029507	0.024339	0.194935	0.000117	9.28E-05	9.26E-05
259.25114	Sphinganine d15:0	Sphingosin	C15H33NC	0.003006	0.0014	0.003964	0.000476	0.000807	0.003844	0.00486	0.003583	4.29E-05	7.84E-05	0.019146	0.001292	0.01057	4.85E-05	0.005975	0.00098	0.003778	0.003453	1E-04	8.81E-05	0.000117	0.001531	0.003635	0.008954	6.84E-05	0.000134	0.003903	0.000117	9.28E-05	9.26E-05
261.23031	Sphingosine t14:0	Sphingosin	C14H31NC	0.074144	0.036658	0.027493	0.011294	0.01072	0.036254	0.059846	0.088147	4.29E-05	7.84E-05	0.277799	0.005728	0.182378	0.00086	0.066351	0.001981	0.033586	0.053789	1E-04	8.81E-05	0.006132	0.056351	0.068356	0.114679	0.017203	0.006592	0.11147	0.000117	9.28E-05	9.26E-05
271.25117	Sphinganine d16:1	Sphingosin	C16H33NC	0.008884	0.009052	0.00253	5.89E-05	0.002552	0.018449	0.021838	0.019991	4.29E-05	7.84E-05	0.030754	8.25E-05	0.022479	4.85E-05	0.020566	7.84E-05	0.016757	0.01068	1E-04	8.81E-05	0.000117	0.000125	0.019385	0.018947	6.84E-05	0.000134	0.014144	0.000117	9.28E-05	9.26E-05
273.26687	Sphinganine d16:0	Sphingosin	C16H35NC	0.118865	0.082232	0.252674	0.015431	0.082281	0.250276	0.150103	0.459005	0.001133	7.84E-05	1.903019	0.159512	0.724001	0.015372	0.372921	0.119445	0.161505	0.107173	0.005289	0.001984	0.14394	0.034874	0.139631	0.618073	0.027771	0.01203	0.217932	0.005956	0.002399	0.004445
289.26149	Sphingosine t16:0	Sphingosin	C16H35NC	0.011202	0.003232	0.015429	0.001142	0.008166	0.010836	0.009406	0.092456	4.29E-05	7.84E-05	0.14574	0.008166	0.06219	0.001353	0.010758	0.004247	0.009348	0.00817	1E-04	8.81E-05	0.006197	0.004688	0.012968	0.043993	0.004716	0.005771	0.04989	0.000117	9.28E-05	9.26E-05
299.28142	Sphingosine d18:1	Sphingosin	C18H37NC	0.002147	6.68E-05	0.000206	5.89E-05	3.95E-05	9.79E-05	9.27E-05	9.08E-05	4.29E-05	7.84E-05	0.000116	8.25E-05	9.09E-05	4.85E-05	8.68E-05	0.002451	0.002146	0.003142	0.002984	8.81E-05	0.000117	0.000125	0.000108	0.002712	6.84E-05	0.000134	6.87E-05	0.000117	9.28E-05	9.26E-05
301.298	Sphinganine d18:0	Sphingosin	C18H39NC	0.001494	0.002737	0.009171	5.89E-05	0.003765	0.003304	9.27E-05	0.021575	4.29E-05	7.84E-05	0.109497	0.012828	0.017924	4.85E-05	0.021631	0.005112	0.001277	0.000102	1E-04	8.81E-05	0.012188	0.000125	0.000108	0.008978	6.84E-05	0.000134	0.003238	0.000117	9.28E-05	9.26E-05
317.2932	Sphingosine t18:0	Sphingosin	C18H39NC	0.003363	6.68E-05	0.001546	5.89E-05	0.001282	0.001389	9.27E-05	0.010663	4.29E-05	7.84E-05	0.025028	0.002499	0.004886	4.85E-05	8.68E-05	0.002256	0.00207	0.000102	1E-04	8.81E-05	0.003019	0.000125	0.000108	0.00007	6.84E-05	0.003618	6.87E-05	0.000117	9.28E-05	9.26E-05
788.47478	SQDG 16:0-16:3	Sulfoquino	C41H72O1	7.85E-05	0.001985	0.000206	0.000862	0.000943	0.002004	0.001852	0.001372	0.001927	0.001544	0.002092	0.001236	0.0011	4.85E-05	0.001666	0.003031	0.001052	0.001511	0.002693	0.001628	0.00138	0.003259	0.002843	0.000913	0.000576	0.001895	0.002919	0.004876	0.002802	0.002112
792.50492	SQDG 16:0-16:1	Sulfoquino	C41H76O1	7.85E-05	0.002248	0.000206	5.89E-05	3.95E-05	9.79E-05	9.27E-05	9.08E-05	0.002075	7.84E-05	0.000116	8.25E-05	9.09E-05	4.85E-05	8.68E-05	0.004913	5.37E-05	0.000102	0.002757	8.81E-05	0.000117	0.005294	0.000108	0.00007	6.84E-05	0.000134	0.003577	0.004731	0.004911	9.26E-05
794.52025	SQDG 16:0-16:0	Sulfoquino	C41H78O1	0.004919	0.010574	0.005925	0.005021	0.00492	0.005834	0.004408	0.003475	0.008699	0.004469	0.006613	0.004434	0.001985	0.001779	0.004888	0.015274	0.005714	0.006666	0.008624	0.006097	0.006654	0.015861	0.009153	0.005462	0.003745	0.009072	0.019458	0.02368	0.019798	0.008706
816.50489	SQDG 16:0-18:3	Sulfoquino	C43H76O1	0.051914	0.128201	0.058806	0.059886	0.058505	0.103346	0.0881	0.077182	0.115352	0.071201	0.1296	0.080362	0.053808	0.038858	0.10204	0.21813	0.064556	0.085394	0.133999	0.093615	0.091546	0.226145	0.161651	0.059975	0.049176	0.079226	0.168612	0.248098	0.176568	0.110169
818.52068	SQDG 16:0-18:2	Sulfoquino	C43H78O1	0.007677	0.018364	0.008376	0.008953	0.009266	0.015437	0.012057	0.00956	0.016989	0.009985	0.020434	0.013226	0.009766	0.006028	0.016096	0.041768	0.014258	0.021816	0.028514	0.020671	0.014925	0.03822	0.02775	0.011304	0.010395	0.017784	0.035368	0.050922	0.036916	0.017588
820.53617	SQDG 16:0-18:1	Sulfoquino	C43H80O1	0.003928	0.00873	0.004362	0.004613	0.00387	0.006861	0.004717	0.003519	0.008449	0.004217	0.008064	0.006215	0.003286	0.002867	0.006223	0.020176	0.005731	0.008342	0.010959	0.006194	0.007245	0.017029	0.016301	0.005945	0.004608	0.008661	0.019016	0.026903	0.020678	0.009144

838.48924	SQDG 18:3-18:3	Sulfoquino	C45H74O1	0.01408	0.028827	0.016551	0.015222	0.014812	0.034739	0.0263	0.025157	0.025715	0.0243	0.048043	0.024952	0.02135	0.014643	0.039889	0.05733	0.019628	0.029196	0.05336	0.04121	0.035556	0.069616	0.056461	0.020686	0.01648	0.025528	0.041597	0.062945	0.04709	0.044403
840.50472	SQDG 18:2-18:3	Sulfoquino	C45H76O1	7.85E-05	0.010741	0.000206	5.89E-05	3.95E-05	9.79E-05	9.27E-05	9.08E-05	0.008569	7.84E-05	0.015268	8.25E-05	9.09E-05	4.85E-05	8.68E-05	0.021832	0.008434	0.017533	0.019785	0.013405	0.000117	0.026798	0.01835	0.00007	0.004214	0.000134	0.015736	0.022814	0.01627	0.014835
842.52053	SQDG 18:1-18:3	Sulfoquino	C45H78O1	0.00198	0.004579	0.002507	0.002559	0.002721	0.003739	0.0031	0.002294	0.003741	0.002757	0.005574	0.00358	0.002155	0.001544	0.004338	0.008157	0.002618	0.003756	0.004528	0.003255	0.003386	0.006907	0.00535	0.002053	0.001718	0.002823	0.005636	0.008842	0.00571	0.00405
772.65725	TG 14:1-16:1-16:1	Triacylglyce	C49H88O6	0.01429	0.0091	0.005687	0.00794	0.004454	0.013573	0.018643	0.014137	0.008223	0.011077	0.013607	0.011654	0.0146	0.011676	0.019065	0.029045	0.012082	0.01885	0.02193	0.014375	0.018571	0.023973	0.019831	0.02126	0.011473	0.01514	0.00512	0.024406	0.017454	0.014665
774.67294	TG 14:1-16:0-16:1	Triacylglyce	C49H90O6	0.061848	0.030371	0.022551	0.033155	0.017182	0.051692	0.075357	0.064572	0.031031	0.045934	0.051481	0.047107	0.062419	0.04537	0.079657	0.11466	0.051106	0.074191	0.095956	0.058645	0.080754	0.10107	0.082341	0.09392	0.047905	0.056262	0.022018	0.106613	0.074044	0.063922
776.68856	TG 14:0-16:0-16:1	Triacylglyce	C49H92O6	0.113205	0.058737	0.039813	0.056762	0.030496	0.106214	0.142197	0.113451	0.060769	0.085309	0.085022	0.078386	0.101109	0.074136	0.133518	0.221607	0.089882	0.138949	0.157945	0.094689	0.14277	0.172421	0.125313	0.17624	0.08171	0.104286	0.037276	0.183858	0.132331	0.115551
778.70401	TG 14:0-16:0-16:0	Triacylglyce	C49H94O6	0.09571	0.068877	0.051921	0.055371	0.031129	0.102441	0.103497	0.105186	0.047212	0.079801	0.075819	0.071314	0.073786	0.056926	0.09857	0.162111	0.066591	0.107531	0.121575	0.075259	0.106254	0.126619	0.088953	0.125136	0.06543	0.090851	0.037265	0.151239	0.11198	0.10291
786.67312	TG 15:1-16:1-16:1	Triacylglyce	C50H90O6	0.018124	0.012375	0.004948	0.014819	0.007208	0.019978	0.02496	0.020811	0.010877	0.015643	0.017903	0.011687	0.027893	0.017539	0.026437	0.038231	0.013401	0.030376	0.034446	0.016546	0.035211	0.032523	0.031095	0.034458	0.015418	0.018375	0.007131	0.040562	0.024704	0.015753
788.68856	TG 15:1-16:0-16:1	Triacylglyce	C50H92O6	0.05471	0.029645	0.020059	0.030588	0.015468	0.055545	0.067506	0.058778	0.028018	0.042365	0.045079	0.041215	0.05723	0.040387	0.073317	0.106682	0.045461	0.074326	0.084902	0.052691	0.071132	0.090068	0.074533	0.083293	0.041549	0.051969	0.019788	0.093587	0.066274	0.056463
790.70415	TG 15:0-16:0-16:1	Triacylglyce	C50H94O6	0.102994	0.060409	0.034564	0.057094	0.028521	0.098564	0.117204	0.101441	0.04925	0.072118	0.074025	0.071474	0.091095	0.066429	0.112627	0.191994	0.07542	0.128726	0.14767	0.084554	0.118405	0.158004	0.116474	0.147876	0.069856	0.087406	0.031576	0.157932	0.111572	0.100948
792.71956	TG 15:0-16:0-16:0	Triacylglyce	C50H96O6	0.084464	0.058677	0.040046	0.045771	0.026139	0.088443	0.083841	0.082347	0.038396	0.063967	0.066065	0.059351	0.062287	0.048993	0.082171	0.151538	0.057143	0.097091	0.109457	0.064027	0.088073	0.118033	0.079964	0.103455	0.054845	0.077892	0.03121	0.123521	0.091913	0.091495
796.65676	TG 16:1-16:1-16:3	Triacylglyce	C51H88O6	0.001257	6.68E-05	0.000206	5.89E-05	3.95E-05	9.79E-05	9.27E-05	9.08E-05	0.000692	7.84E-05	0.000116	8.25E-05	0.005282	0.000508	0.001195	0.001762	5.37E-05	0.000102	0.001122	8.81E-05	0.000117	0.001385	0.000108	0.00007	0.000546	0.000134	6.87E-05	0.000117	9.28E-05	0.000925
798.67278	TG 14:1-16:1-18:2	Triacylglyce	C51H90O6	0.003571	0.002187	0.001569	0.002446	0.001253	0.003571	0.005231	0.004507	0.002341	0.003231	0.003958	0.003098	0.004949	0.003188	0.005886	0.008205	0.003178	0.004871	0.006755	0.00422	0.00481	0.006712	0.005444	0.005682	0.00289	0.00414	0.001615	0.007009	0.005026	0.003702
800.68845	TG 14:1-16:1-18:1	Triacylglyce	C51H92O6	0.031014	0.015158	0.014153	0.015759	0.008645	0.024853	0.037041	0.030942	0.015705	0.023519	0.02591	0.021045	0.030396	0.020967	0.035629	0.052995	0.02459	0.038711	0.043728	0.027713	0.03771	0.046764	0.038913	0.045142	0.023526	0.028257	0.01035	0.047786	0.03442	0.028679
802.70428	TG 16:0-16:1-16:1	Triacylglyce	C51H94O6	0.143241	0.073409	0.048917	0.079863	0.042756	0.137037	0.18983	0.140027	0.080085	0.110696	0.110162	0.105041	0.142536	0.098617	0.174852	0.286107	0.116688	0.183278	0.196121	0.125759	0.191921	0.240532	0.177161	0.229706	0.11125	0.135546	0.046263	0.236572	0.171328	0.151362
804.71983	TG 16:0-16:0-16:1	Triacylglyce	C51H96O6	0.188291	0.112407	0.060787	0.102851	0.054408	0.197175	0.212311	0.194584	0.094922	0.143327	0.145082	0.128416	0.164524	0.124502	0.212427	0.365227	0.138503	0.229747	0.253409	0.142103	0.227174	0.304838	0.206979	0.281059	0.132218	0.178316	0.060343	0.290071	0.213389	0.18973
806.73513	TG 16:0-16:0-16:0	Triacylglyce	C51H98O6	0.039218	0.026088	0.040867	0.025673	0.012486	0.03391	0.039126	0.044509	0.020337	0.031714	0.037045	0.028202	0.028698	0.019074	0.034988	0.043559	0.019567	0.032873	0.038347	0.027096	0.040632	0.039716	0.034282	0.038328	0.026193	0.038666	0.014796	0.051494	0.039831	0.034861
806.73518	TG 14:0-16:0-18:0	Triacylglyce	C51H98O6	0.136307	0.103323	0.072714	0.080989	0.051687	0.157493	0.133458	0.142676	0.067085	0.112883	0.112393	0.097696	0.101885	0.075745	0.119898	0.253446	0.088267	0.159508	0.167964	0.108471	0.1497	0.205668	0.132436	0.161172	0.094861	0.139468	0.048167	0.205826	0.143283	0.14973
814.7041	TG 15:0-16:1-18:2	Triacylglyce	C52H94O6	0.025868	0.013348	0.010229	0.01509	0.007892	0.025544	0.032334	0.028822	0.013519	0.02111	0.020617	0.019853	0.02591	0.019347	0.033796	0.049685	0.022048	0.034101	0.040185	0.024004	0.035374	0.04348	0.034542	0.038556	0.020693	0.025057	0.009456	0.044244	0.03034	0.026337
816.71963	TG 15:0-16:1-18:1	Triacylglyce	C52H96O6	0.085738	0.045635	0.026624	0.046934	0.025761	0.080826	0.093325	0.087388	0.043587	0.06063	0.063113	0.063984	0.079973	0.05609	0.100092	0.169425	0.066507	0.109469	0.123501	0.075958	0.106208	0.141547	0.10689	0.124479	0.063795	0.079843	0.027433	0.136703	0.096205	0.082132
818.73536	TG 16:0-16:1-17:0	Triacylglyce	C52H98O6	0.10732	0.060236	0.033716	0.053332	0.032456	0.108867	0.113204	0.103226	0.049459	0.07394	0.075449	0.074063	0.08887	0.069221	0.117972	0.212673	0.079292	0.127211	0.139357	0.081422	0.114832	0.17127	0.1234	0.150237	0.071153	0.091158	0.032776	0.159154	0.111819	0.108764
820.75089	TG 16:0-16:0-17:0	Triacylglyce	C52H100O	0.062336	0.044078	0.031104	0.035812	0.02023	0.063907	0.055962	0.056737	0.026551	0.049178	0.04586	0.040806	0.040754	0.03591	0.052486	0.11105	0.039494	0.069472	0.073131	0.044205	0.062217	0.088128	0.054685	0.06815	0.039998	0.05472	0.021966	0.079939	0.062567	0.058759
822.67287	TG 16:0-16:3-18:3	Triacylglyce	C53H90O6	7.85E-05	0.002412	0.001756	0.003742	0.00189	0.005191	0.004166	0.005468	0.003399	0.002917	0.009966	0.008389	0.009305	0.003726	0.00923	0.005959	0.004259	0.008634	0.011672	0.005843	0.00706	0.006471	0.006823	0.003603	0.003727	0.00648	0.005376	0.01021	0.007277	0.007865
826.70399	TG 16:0-16:1-18:3	Triacylglyce	C53H94O6	0.011499	0.007234	0.005301	0.007375	0.004365	0.011294	0.015667	0.014519	0.007761	0.011108	0.01556	0.012456	0.01434	0.00959	0.017086	0.021981	0.01064	0.017413	0.023722	0.013114	0.015477	0.021645	0.018221	0.018484	0.010645	0.014392	0.006895	0.025612	0.015844	0.015438
828.71972	TG 16:0-16:1-18:2	Triacylglyce	C53H96O6	0.071092	0.036568	0.025578	0.040692	0.022908	0.068546	0.09366	0.071384	0.043869	0.057332	0.06948	0.063963	0.076577	0.04861	0.095193	0.138758	0.062114	0.103363	0.111692	0.063815	0.094137	0.120063	0.088935	0.111727	0.056505	0.075539	0.029067	0.129699	0.088556	0.077631
830.73549	TG 16:0-16:1-18:1	Triacylglyce	C53H98O6	0.149704	0.085812	0.048865	0.086207	0.049482	0.171986	0.181835	0.170015	0.084786	0.129247	0.134621	0.125586	0.150839	0.109953	0.197186	0.307904	0.122486	0.205879	0.220724	0.13181	0.192408	0.277727	0.183581	0.235614	0.110387	0.161801	0.056058	0.265887	0.17968	0.164795
832.75104	TG 16:0-16:0-18:1	Triacylglyce	C53H100O	0.15074	0.095688	0.044397	0.080122	0.047441	0.165305	0.148717	0.152635	0.07976	0.128896	0.107753	0.107967	0.114115	0.090554	0.144563	0.299595	0.102298	0.177323	0.197003	0.126481	0.17692	0.237167	0.162334	0.193516	0.097993	0.133653	0.046131	0.244392	0.177482	0.161577
834.76647	TG 16:0-16:0-18:0	Triacylglyce	C53H102O	0.085665	0.06769	0.033742	0.050737	0.030204	0.098242	0.069245	0.069775	0.03731	0.067475	0.062512	0.057469	0.053839	0.042791	0.064349	0.128563	0.055052	0.094078	0.102293	0.059442	0.084207	0.135332	0.077743	0.076624	0.054646	0.100683	0.033554	0.115827	0.091236	0.083215
836.6796	TG 15:0-18:3-18:3	Triacylglyce	C54H92O6	7.85E-05	6.68E-05	0.001086	0.001561	0.001064	0.003351	0.003889	0.003244	0.001139	0.002785	0.000116	0.0024	0.002721	0.001839	0.004161	0.00319	0.002844	0.003958	0.005506	0.002233	0.001828	0.000125	0.00231	0.002993	6.84E-05	0.000134	6.87E-05	0.000117	0.003132	0.003206
840.71944	TG 15:1-18:1-18:2	Triacylglyce	C54H96O6	0.006562	0.0036	0.002126	0.003594	0.00227	0.007024	0.008338	0.00784	0.004031	0.005851	0.007749	0.006693	0.00765	0.005516	0.008933	0.013607	0.005627	0.009881	0.013177	0.007327	0.009038	0.01191	0.011602	0.010405	0.005313	0.00748	0.003065	0.012569	0.009393	0.008194
842.73512	TG 16:0-17:1-18:2	Triacylglyce	C54H98O6	0.027552	0.01423	0.009612	0.015182	0.00843	0.025429	0.032139	0.027953	0.014302	0.020962	0.023416	0.021714	0.02596	0.018727	0.035096	0.053458	0.021654	0.036045	0.042856	0.024247	0.034339	0.044891	0.037042	0.040502	0.020207	0.025785	0.00993	0.045841	0.030894	0.028336
844.65715	TG 16:3-18:3-18:3	Triacylglyce	C55H88O6	0.004345	0.004045	0.006115	0.005943	0.004146	0.007963	0.007726	0.006394	0.00673	0.007567	0.013292	0.008986	0.010388	0.005224	0.01508	0.012003	0.011291	0.015849	0.025678	0.012584	0.024871	0.014245	0.010965	0.009255	0.007134	0.012912	0.012814	0.020657	0.016097	0.017053
844.75082	TG 16:1-17:0-18:1	Triacylglyce	C54H100O	0.050589	0.031583	0.016923	0.028362	0.016773	0.056986	0.060129	0.05616	0.025887	0.039291	0.040689	0.037402	0.04483	0.036003	0.063635	0.107559	0.042344	0.070681	0.071494	0.044642	0.061334	0.09246	0.064201	0.077548	0.039343	0.050864	0.017718	0.086566	0.06256	0.058812
846.76645	TG 16:0-17:0-18:1	Triacylglyce	C54H102O	0.049561	0.030065	0.016544	0.025814	0.014669	0.0538	0.048264	0.043825	0.021787	0.036973	0.033723	0.029929	0.034506	0.030229	0.047069	0.099672	0.032936	0.055321	0.058802	0.036083	0.048726	0.078097	0.048044	0.059955	0.031967	0.043343	0.015387	0.065287	0.049448	0.043744
848.68839	TG 16:1-18:3-18:3	Triacylglyce	C55H92O6	0.00945	0.011461	0.007804	0.012051	0.008252	0.019164	0.018114	0.016679	0.010824	0.016214	0.024385	0.019808	0.019313	0.010898	0.02776	0.027274	0.01898	0.029113	0.041373	0.030587	0.026596	0.028151	0.023602	0.016198	0.016204	0.032078	0.024329	0.041709	0.026268	0.029673
848.78207	TG 16:0-17:0-18:0	Triacylglyce	C54H104O	0.022718	0.018354	0.010571	0.015151	0.008574	0.025882	0.021608	0.019224	0.009716	0.018574	0.016739	0.015402	0.014282	0.01253	0.018621	0.038989	0.015661	0.028737	0.027751	0.014811	0.023135	0.034702	0.020225	0.024105	0.013709	0.024976	0.008544	0.031359	0.022182	0.023287
850.70385	TG 16:0-18:3-18:3	Triacylglyce	C55H94O6	0.019562	0.023531	0.022813	0.027595	0.019654	0.054073	0.047372	0.046084	0.029461	0.040029	0.080117	0.058172	0.061773	0.031531	0.072519	0.05236	0.040712	0.062284	0.096817	0.05392	0.051518	0.058815	0.043328	0.036008	0.035849	0.061871	0.050065	0.086785	0.060784	0.067663
852.71956	TG 16:0-18:2-18:3	Triacylglyce	C55H96O6	0.045522	0.054238	0.037739	0.062622	0.043065	0.106137	0.103774	0.09762	0.062508	0.096282	0.173384	0.127539	0.136795	0.074691	0.172309	0.116614	0.090415	0.13467	0.197504	0.111255	0.095528	0.11214	0.090791	0.077643	0.071215	0.129779	0.092772	0.180309	0.121418	0.134646
854.73521	TG 16:0-18:2-18:2	Triacylglyce	C55H98O6	0.060522	0.058892	0.033017	0.048987	0.040083	0.089307	0.103684	0.094292	0.059338	0.088902	0.125057	0.101543	0.116998	0.069973	0.13661	0.118238	0.06898	0.105791	0.177588	0.100623	0.090602	0.108603	0.182089	0.09532	0.05953	0.087878	0.054757	0.163748	0.107559	0.112333
856.75081	TG 16:0-18:1-18:2	Triacylglyce	C55H100O	0.055918	0.039538	0.025593	0.031184	0.023225	0.056504	0.065826	0.065429	0.037339	0.05933	0.053273	0.057946	0.055408	0.038816	0.065564	0.088608	0.043409	0.071029	0.092304	0.059462	0.063284	0.080009	0.071375	0.071028	0.039712	0.053213	0.025206	0.108205	0.073721	0.06465
858.76667	TG 16:0-18:1-18:1	Triacylglyce	C55H102O	0.108719	0.064646	0.033915	0.053877	0.036076	0.113713	0.105192	0.110086	0.071867	0.117595	0.083898	0.088699	0.082087	0.07065	0.104068	0.193435	0.070003	0.133776	0.182328	0.114491	0.11692	0.158481	0.120661	0.126414	0.068813	0.088948	0.038863	0.23126	0.149079	0.129593
860.78212	TG 16:0-18:0-18:1	Triacylglyce	C55H104O	0.070461	0.046417	0.021316	0.035498	0.021437	0.072917	0.064187	0.05732	0.039704	0.076617	0.042503	0.044463	0.046503	0.037498	0.059424	0.114465	0.041475	0.073709	0.095382	0.064721	0.066505	0.10379	0.06261	0.07394	0.038156	0.063077	0.021749	0.115099	0.092543	0.077865
862.79785	TG 16:0-18:0-18:0	Triacylglyce	C55H106O	0.060053	0.052502	0.019633	0.047507	0.020231	0.081987	0.047589	0.048203	0.023561	0.052367	0.039048	0.038661	0.027518	0.024398	0.035096	0.0885	0.04907	0.078266	0.060883	0.035434	0.079477	0.107718	0.049781	0.050361	0.04759	0.079924	0.036087	0.066544	0.054837	0.048461
870.76643	TG 17:1-18:1-18:1	Triacylglyce	C56H102O	0.013937	0.009416	0.004216	0.006843	0.004488	0.014109	0.016621	0.014716	0.007291	0.01146	0.011729	0.012092	0.012827	0.010317	0.016157	0.028499	0.011087	0.01835	0.020719	0.013027	0.017245	0.024432	0.018211	0.019562	0.00986	0.014415	0.004974	0.023091	0.017386	0.015566

872.68819	TG 14:3-18:3-22:3	Triacyglyce	C57H92O6	0.044768	0.059689	0.042332	0.070004	0.049198	0.103356	0.093437	0.09764	0.058797	0.082064	0.150572	0.102989	0.11666	0.054249	0.150117	0.158634	0.117908	0.148338	0.241723	0.157898	0.132045	0.161865	0.115394	0.091017	0.097509	0.184376	0.147543	0.247117	0.1638	0.172018
874.70406	TG 18:2-18:3-18:3	Triacyglyce	C57H94O6	0.131475	0.180288	0.117224	0.199846	0.148111	0.316399	0.282198	0.265729	0.160035	0.246409	0.417301	0.308343	0.34377	0.164907	0.453961	0.369988	0.26866	0.377312	0.514677	0.381043	0.325678	0.384411	0.274521	0.207464	0.222681	0.454724	0.353633	0.583096	0.361634	0.437514
876.71936	TG 18:2-18:2-18:3	Triacyglyce	C57H96O6	0.035805	0.042996	0.046911	0.045216	0.029195	0.063271	0.062629	0.058147	0.032779	0.057058	0.076729	0.060145	0.069623	0.032811	0.078048	0.054092	0.039137	0.0589	0.082154	0.0573	0.055944	0.061614	0.047738	0.036806	0.039191	0.077655	0.054081	0.092194	0.064715	0.06347
876.71963	TG 18:1-18:2-18:4	Triacyglyce	C57H96O6	0.066244	0.093231	0.058534	0.100696	0.069753	0.139502	0.134039	0.131793	0.081723	0.126102	0.20737	0.166767	0.168078	0.089012	0.243788	0.16011	0.118584	0.153975	0.259758	0.171911	0.14341	0.152794	0.134129	0.093206	0.098663	0.188518	0.140147	0.277057	0.171456	0.185408
878.73496	TG 18:2-18:2-18:2	Triacyglyce	C57H98O6	0.023735	0.03326	0.025798	0.022917	0.017797	0.029817	0.028924	0.031846	0.018372	0.037812	0.04612	0.033823	0.04124	0.020808	0.044097	0.027476	0.020236	0.03253	0.053102	0.038717	0.029882	0.026944	0.107156	0.024481	0.019338	0.029532	0.024382	0.048949	0.033944	0.033214
878.73508	TG 18:0-18:3-18:3	Triacyglyce	C57H98O6	0.047195	0.070659	0.033883	0.05138	0.042596	0.058937	0.0689	0.071627	0.044465	0.082766	0.108878	0.082742	0.106178	0.053455	0.119894	0.078961	0.05853	0.080923	0.173883	0.112179	0.073539	0.07116	0.350028	0.069121	0.050545	0.070187	0.060051	0.145551	0.09507	0.089122
880.75054	TG 18:1-18:2-18:2	Triacyglyce	C57H100O	0.049097	0.067911	0.022172	0.03158	0.034452	0.050637	0.056972	0.059838	0.037879	0.070579	0.080383	0.064802	0.085149	0.043256	0.080176	0.07826	0.042695	0.070371	0.17445	0.109123	0.059215	0.062301	0.134429	0.067065	0.039473	0.042622	0.031491	0.123132	0.081309	0.070303
882.76616	TG 18:1-18:1-18:2	Triacyglyce	C57H102O	0.053082	0.056316	0.020444	0.028878	0.031469	0.05553	0.055871	0.060624	0.03519	0.066922	0.06214	0.062528	0.065981	0.037268	0.068545	0.087528	0.041732	0.072431	0.13191	0.085715	0.054309	0.0615	0.077785	0.061867	0.039488	0.037406	0.026601	0.114659	0.073734	0.062404
884.78168	TG 18:1-18:1-18:1	Triacyglyce	C57H104O	0.024183	0.020118	0.018183	0.011822	0.010279	0.022542	0.024454	0.026845	0.016337	0.02317	0.019789	0.019404	0.018502	0.01315	0.023602	0.026101	0.012394	0.025509	0.038305	0.026998	0.019398	0.023098	0.023932	0.019858	0.014234	0.016765	0.010421	0.044818	0.027549	0.026486
884.78201	TG 18:0-18:1-18:2	Triacyglyce	C57H104O	0.072175	0.066003	0.025274	0.031268	0.036146	0.073662	0.070556	0.073189	0.047984	0.075674	0.056679	0.060792	0.056031	0.044364	0.076893	0.119708	0.046878	0.099008	0.164109	0.102525	0.059231	0.091803	0.07609	0.074084	0.045929	0.050114	0.031053	0.173229	0.090382	0.090331
886.79734	TG 18:0-18:1-18:1	Triacyglyce	C57H106O	0.037568	0.032483	0.01268	0.02074	0.017679	0.038195	0.035923	0.035258	0.020804	0.0405	0.030723	0.029868	0.030058	0.024584	0.034249	0.062828	0.024708	0.047206	0.066491	0.040964	0.035379	0.052694	0.0382	0.042582	0.023082	0.03328	0.014544	0.072704	0.044448	0.041379
890.81661	TG 16:0-18:0-20:0	Triacyglyce	C57H110O	0.013041	0.010842	0.007272	0.009221	0.004751	0.014944	0.013312	0.01335	0.006657	0.011966	0.011979	0.010476	0.009046	0.007472	0.011161	0.019913	0.010452	0.014816	0.016744	0.010773	0.019362	0.020084	0.013405	0.01378	0.010836	0.018059	0.007959	0.02027	0.015583	0.014071
890.82881	TG 14:0-16:0-24:0	Triacyglyce	C57H110O	0.054207	0.044427	0.015703	0.083341	0.015605	0.078471	0.045343	0.035728	0.013177	0.047204	0.020681	0.032544	0.016148	0.014769	0.021166	0.072014	0.047283	0.084857	0.047461	0.023724	0.071696	0.096303	0.035301	0.044323	0.042687	0.071194	0.039609	0.050468	0.029595	0.033137
898.79698	TG 18:1-18:1-19:1	Triacyglyce	C58H106O	0.004583	0.003427	0.001224	0.002527	0.001528	0.00569	0.005092	0.005297	0.002516	0.003734	0.003663	0.003565	0.004164	0.003085	0.003929	0.011435	0.003471	0.006725	0.006328	0.004805	0.005004	0.008124	0.006078	0.005996	0.003531	0.004672	0.002	0.008573	0.005321	0.004129
900.81273	TG 15:1-16:1-24:0	Triacyglyce	C58H108O	0.009805	0.007189	0.00336	0.00545	0.003466	0.010859	0.01082	0.008823	0.004361	0.008366	0.00742	0.006466	0.007224	0.006849	0.010176	0.019201	0.007318	0.01379	0.013278	0.007218	0.012421	0.015585	0.011221	0.013202	0.006198	0.009988	0.003553	0.015144	0.009609	0.009844
904.74945	TG 18:2-18:3-20:2	Triacyglyce	C59H100O	0.006284	0.041839	0.003838	0.007563	0.012117	0.005446	0.005897	0.005971	0.003545	0.005483	0.008963	0.01046	0.009033	0.015929	0.009706	0.00921	0.015282	0.009393	0.042146	0.012522	0.007022	0.006904	0.008251	0.004766	0.004481	0.008871	0.006955	0.010819	0.007087	0.00905
904.8441	TG 15:0-16:0-24:0	Triacyglyce	C58H112O	0.017209	0.013285	0.007546	0.012685	0.007155	0.017743	0.016389	0.01626	0.006934	0.013919	0.011256	0.011989	0.010332	0.010079	0.013138	0.029404	0.012858	0.018744	0.022172	0.01194	0.018649	0.025258	0.016455	0.018588	0.009943	0.019779	0.006813	0.022083	0.015991	0.016465
906.7652	TG 18:3-18:3-20:0	Triacyglyce	C59H102O	0.011403	0.064324	0.004624	0.010118	0.025502	0.006499	0.005908	9.08E-05	0.003601	0.005747	0.00979	0.012926	0.012989	0.026046	0.011659	0.014474	0.024734	0.015657	0.074927	0.021697	0.007993	0.006994	0.015114	0.005035	0.004505	0.007164	0.0109	0.010387	0.006184	0.015004
908.77978	TG 18:2-18:2-20:1	Triacyglyce	C59H104O	0.013958	0.063719	0.004829	0.010881	0.02969	0.006761	0.00676	0.006938	0.003863	0.006247	0.009259	0.013957	0.015524	0.02763	0.010728	0.01472	0.028233	0.015319	0.077042	0.023272	0.007726	0.007541	0.009684	0.006588	0.00436	0.007	0.009787	0.012255	0.007049	0.015387
910.7971	TG 18:1-18:2-20:1	Triacyglyce	C59H106O	0.009947	0.037382	0.003444	0.007651	0.019844	0.008875	0.007773	0.008014	0.004329	0.00751	0.009527	0.0105	0.010824	0.016593	0.010285	0.015887	0.018295	0.014116	0.057421	0.015758	0.009632	0.010537	0.009938	0.008426	0.004477	0.007228	0.006721	0.010932	0.007819	0.012369
912.81262	TG 18:1-18:1-20:1	Triacyglyce	C59H108O	0.010441	0.032205	0.003543	0.007789	0.018132	0.010656	0.008931	0.008319	0.004198	0.007931	0.007044	0.009778	0.009342	0.014001	0.009566	0.018623	0.015168	0.013428	0.048351	0.015157	0.010661	0.014921	0.01112	0.010048	0.005643	0.008671	0.005481	0.014691	0.010001	0.013839
914.81643	TG 18:0-18:2-20:0	Triacyglyce	C59H110O	0.007503	0.010937	0.004466	0.004577	0.006275	0.007329	0.007735	0.00662	0.004169	0.007636	0.006189	0.006751	0.007741	0.007582	0.007557	0.010722	0.006466	0.00909	0.020949	0.010354	0.008359	0.010118	0.007662	0.009181	0.005138	0.008097	0.003559	0.013827	0.009456	0.009028
914.82844	TG 16:1-18:1-22:0	Triacyglyce	C59H110O	0.020561	0.019368	0.005818	0.011438	0.011358	0.019174	0.018036	0.017243	0.008714	0.015874	0.014026	0.014001	0.014466	0.015117	0.018965	0.038097	0.014821	0.0246	0.03388	0.015934	0.02372	0.030669	0.020653	0.023429	0.012302	0.019488	0.006476	0.02702	0.018854	0.018838
916.84401	TG 16:0-16:1-24:0	Triacyglyce	C59H112O	0.032781	0.023384	0.008849	0.020711	0.011036	0.031746	0.031167	0.025637	0.012183	0.026842	0.017288	0.022731	0.018538	0.020747	0.027307	0.063125	0.022105	0.042282	0.045675	0.021517	0.035664	0.04803	0.030121	0.038183	0.019068	0.034316	0.010643	0.045695	0.03189	0.029429
918.85943	TG 16:0-16:0-24:0	Triacyglyce	C59H114O	0.017653	0.014869	0.006676	0.018408	0.007884	0.018717	0.016163	0.014412	0.006299	0.015001	0.010537	0.012012	0.009022	0.009269	0.012619	0.027376	0.01376	0.019596	0.023708	0.011581	0.019197	0.029734	0.018772	0.018103	0.009191	0.021884	0.007671	0.025065	0.017353	0.016972
928.84366	TG 16:1-17:1-24:0	Triacyglyce	C60H112O	0.012178	0.008773	0.003801	0.006097	0.004182	0.011949	0.012625	0.010356	0.004788	0.010084	0.007947	0.008015	0.008505	0.007811	0.011207	0.021107	0.008634	0.014795	0.016018	0.008523	0.014153	0.017732	0.012046	0.014707	0.007292	0.013049	0.004444	0.015637	0.011408	0.009806
930.85941	TG 16:1-18:0-23:0	Triacyglyce	C60H114O	0.022052	0.016147	0.007399	0.014039	0.007808	0.023064	0.023549	0.020669	0.008929	0.017963	0.014342	0.016271	0.014685	0.014948	0.020892	0.040606	0.017349	0.026871	0.032345	0.013926	0.026472	0.032753	0.023124	0.026739	0.014316	0.023827	0.007672	0.031538	0.021604	0.020174
932.78096	TG 18:3-18:3-22:1	Triacyglyce	C61H104O	0.001065	0.008703	0.000206	0.001558	0.002258	9.79E-05	9.27E-05	9.08E-05	4.29E-05	7.84E-05	0.001618	0.00195	9.09E-05	0.003561	8.68E-05	7.84E-05	0.002918	0.000102	0.007662	0.003295	0.000117	0.001324	0.000108	0.00007	6.84E-05	0.001794	0.001636	0.000117	9.28E-05	0.001797
932.87488	TG 15:0-16:0-26:0	Triacyglyce	C60H116O	0.01137	0.009208	0.004892	0.008375	0.004819	0.011967	0.010589	0.009942	0.004773	0.010225	0.007759	0.008131	0.007073	0.006471	0.009242	0.020569	0.009204	0.014214	0.016715	0.009542	0.013205	0.019051	0.01245	0.012481	0.00693	0.014694	0.005423	0.017913	0.011471	0.011582
934.79564	TG 18:2-18:3-22:1	Triacyglyce	C61H106O	0.004036	0.019401	0.001212	0.002737	0.006158	0.002536	0.002	9.08E-05	4.29E-05	0.001432	0.002841	0.003745	0.004066	0.007468	0.002966	0.004219	0.00673	0.004903	0.01808	0.005935	0.002499	0.002112	0.000108	0.00138	0.00172	0.00292	0.002717	0.005587	0.002873	0.003689
936.81079	TG 18:3-20:1-20:1	Triacyglyce	C61H108O	0.006291	0.029098	0.000206	0.004842	0.010051	9.79E-05	0.002646	0.003157	0.001513	0.002688	0.003506	0.005547	0.00499	0.011311	0.004612	0.006623	0.01067	0.006833	0.032517	0.008888	0.003566	0.004358	0.004093	0.00007	6.84E-05	0.000134	0.003975	0.005064	0.002769	0.006834
938.82801	TG 18:2-20:1-20:1	Triacyglyce	C61H110O	7.85E-05	0.026871	0.001677	0.004442	0.011315	0.005226	0.004937	0.004074	0.002629	0.005206	0.005274	0.005935	0.006979	4.85E-05	0.005245	0.007278	0.011948	0.00842	1E-04	8.81E-05	0.004791	0.006596	0.006643	0.004727	0.002681	0.004344	0.003854	0.00785	0.004886	9.26E-05
940.84327	TG 18:2-20:0-20:1	Triacyglyce	C61H112O	0.007888	0.018215	0.002734	0.004808	0.008233	0.007412	0.007454	0.006727	0.003158	0.006811	0.005881	0.006394	0.006925	0.008011	0.007295	0.012955	0.007553	0.009618	0.024213	0.008617	0.008576	0.010712	0.008619	0.00839	0.004159	0.00684	0.003755	0.010544	0.007347	0.008696
942.85891	TG 16:1-18:1-24:0	Triacyglyce	C61H114O	0.016735	0.014005	0.004715	0.010834	0.006907	0.01682	0.017428	0.013668	0.007389	0.014464	0.011936	0.0135	0.01172	0.011615	0.014419	0.031501	0.012761	0.023146	0.02956	0.012581	0.019644	0.024472	0.017378	0.021572	0.010621	0.019276	0.006489	0.025156	0.019279	0.016306
944.8746	TG 16:0-18:1-24:0	Triacyglyce	C61H116O	0.020238	0.014533	0.005752	0.013356	0.007644	0.019248	0.018618	0.014932	0.007769	0.015737	0.011707	0.01359	0.011349	0.012958	0.01754	0.034238	0.01461	0.022701	0.02816	0.013461	0.021307	0.029697	0.020992	0.023227	0.011076	0.022888	0.006738	0.029009	0.019844	0.019837
946.89025	TG 16:0-18:0-24:0	Triacyglyce	C61H118O	0.006876	0.005816	0.002711	0.005611	0.003291	0.007075	0.006628	0.006606	0.002627	0.006394	0.004034	0.004684	0.003349	0.003266	0.004996	0.010975	0.005247	0.008681	0.0096	0.004825	0.007509	0.011622	0.007953	0.006525	0.003752	0.009206	0.003156	0.010668	0.007211	0.006686
956.87438	TG 16:1-18:1-25:0	Triacyglyce	C62H116O	0.009199	0.005941	0.003593	0.006735	0.003277	0.009372	0.009997	0.008859	0.00381	0.008046	0.005787	0.007172	0.006886	0.006385	0.008944	0.016357	0.00712	0.010818	0.012566	0.006819	0.011239	0.014736	0.009865	0.011337	0.006047	0.010632	0.003243	0.012831	0.008612	0.008169
958.88993	TG 16:0-18:1-25:0	Triacyglyce	C62H118O	0.011181	0.007678	0.00351	0.006678	0.003587	0.010302	0.01002	0.00952	0.004095	0.008412	0.005866	0.007368	0.006707	0.006185	0.009388	0.018529	0.007536	0.012157	0.014116	0.007821	0.011577	0.015041	0.010744	0.012277	0.005977	0.011954	0.00374	0.014902	0.009812	0.009653
960.90597	TG 16:0-17:0-26:0	Triacyglyce	C62H120O	0.003505	0.003151	0.001577	0.002949	0.001786	0.003945	0.003037	0.003416	0.00145	0.003818	0.002273	0.002397	0.001927	0.002062	0.003167	0.005486	0.00275	0.00395	0.005319	0.00218	0.00406	0.005419	0.00439	0.003497	0.00206	0.004423	0.001621	0.005775	0.003213	0.003999
964.84008	TG 18:3-20:1-22:1	Triacyglyce	C63H112O	0.001754	0.005916	0.000206	0.001587	0.002085	0.002442	0.001909	9.08E-05	0.000914	0.001913	0.001909	0.001931	0.002079	0.002877	0.001978	0.002996	0.002702	0.003036	0.008043	0.002531	0.002383	0.002714	0.000108	0.001561	0.001272	0.002262	0.001578	0.003097	0.001883	0.002683
966.85849	TG 18:2-20:1-22:1	Triacyglyce	C63H114O	0.002726	0.005298	0.000206	0.001925	0.002059	0.002836	0.002635	0.002385	0.001352	0.002878	0.003048	0.002775	0.002872	0.002662	0.003201	0.004557	0.002752	0.003896	0.006444	0.003661	0.003126	0.004347	0.003281	0.002922	0.001664	0.002707	0.001537	0.004643	0.002853	0.003275
968.87382	TG 18:1-18:2-24:0	Triacyglyce	C63H116O	0.004742	0.005043	0.001433	0.002741	0.002226	0.004564	0.005086	0.004105	0.001886	0.004041	0.00354	0.003838	0.00381	0.003748	0.00443	0.008038	0.004242	0.005494	0.00895	0.004245	0.005549	0.007213	0.005487	0.005206	0.002818	0.005211	0.001902	0.006189	0.004527	0.004738
970.88959	TG 16:1-18:1-26:0	Triacyglyce	C63H118O	0.008726	0.007058	0.00243	0.005612	0.003687	0.009194	0.007936	0.007456	0.003441	0.00786	0.005792	0.006949	0.005841	0.006354	0.008322	0.015414	0.006638	0.010503	0.012995	0.006727	0.010857	0.01286	0.010034	0.011268	0.005587	0.010093	0.003372	0.013113	0.00924	0.008851
972.90479	TG 16:0-18:1-26:0	Triacyglyce	C63H120O	0.006613	0.005248	0.002082	0.004189	0.002475	0.00625	0.006085	0.006225	0.002458	0.005327	0.003232	0.004572	0.003463	0.003834	0.005348	0.011074	0.004698	0.007616	0.009111	0.004589	0.007278	0.009908	0.006979	0.007487	0.003607	0.008396	0.002451	0.009459	0.006991	0.006115

974.91315	TG 16:0-18:0-26:0	Triacyglyce	C63H122O	0.002719	0.001469	0.000206	0.001861	0.001046	0.002027	0.002642	0.002739	0.00129	0.002151	0.001801	0.002245	0.001883	0.001632	0.002602	0.002723	0.001751	0.002649	0.004342	0.002122	0.002787	0.003838	0.003233	0.002989	0.001702	0.003536	6.87E-05	0.004012	0.002911	0.002839
982.88938	TG 18:1-18:2-25:0	Triacyglyce	C64H118O	0.002538	0.001474	0.000206	0.001152	0.000808	0.002012	0.00194	0.002004	0.000902	0.001733	0.001377	0.001793	0.0016	0.001438	0.001851	0.003964	0.00174	0.002766	0.003232	0.001391	0.002468	0.003533	0.002631	0.002665	0.001454	0.002422	0.000681	0.003121	0.001912	0.002033

Mass (M)	Name	Compound	FORMULA (	Ler-R1	Ler-R2	Ler-R3	Ler-R4	Ler-R5	Average	Stdev	arc3-R1	arc3-R2	arc3-R3	arc3-R4	arc3-R5	Average	Stdev	t-test	arc5-R1	arc5-R2	arc5-R3	arc5-R4	arc5-R5	Average	Stdev	t-test	Col-0-R1	Col-0-R2	Col-0-R3	Col-0-R4	Col-0-R5	Average	Stdev	kac1/2-R1	kac1/2-R2	kac1/2-R3	kac1/2-R4	kac1/2-R5	Average	Stdev	t-test	chup1-R1	chup1-R2	chup1-R3	chup1-R4	chup1-R5	Average	Stdev	t-test
774.45519	Arabidopside A	Arabidopsi	C43H66O1	0.048612	0.040292	0.055173	0.043099	0.023144	0.042064	0.012009	0.060663	0.083485	0.064956	0.050341	0.059942	0.063877	0.012193	0.021478	0.080593	0.127768	0.081635	0.052593	0.081614	0.084841	0.027025	0.011977	0.068703	0.044463	0.098902	0.086402	0.052251	0.070144	0.022766	0.241174	0.194889	0.24499	0.158533	0.166467	0.201211	0.040565	0.000233	0.293636	0.145723	0.286653	0.177236	0.131633	0.206976	0.077736	0.005409
802.4869	Arabidopside B	Arabidopsi	C45H70O1	0.000393	0.001952	0.000206	0.001023	0.000544	0.000824	0.000699	0.00159	0.001868	9.08E-05	4.29E-05	7.84E-05	0.000734	0.000914	0.866125	0.000116	8.25E-05	0.002344	0.001643	0.00295	0.001427	0.001297	0.386426	0.001383	0.000824	0.000937	0.002338	0.000557	0.001208	0.000698	0.005172	0.006581	0.008372	0.006007	0.006729	0.006572	0.001177	2.25E-05	0.00554	0.005052	0.007936	0.004376	9.26E-05	0.004599	0.002854	0.032568
537.51165	Cer d18:1h16:0	Ceramides	C34H67NC	0.0171	0.02473	0.010477	0.016681	0.01471	0.01674	0.00518	0.016166	0.013319	0.015327	0.009384	0.014932	0.013826	0.00269	0.296634	0.0303	0.021799	0.019586	0.015072	0.027344	0.02282	0.006082	0.127176	0.013503	0.010603	0.023145	0.025184	0.012549	0.016997	0.006665	0.009849	0.016031	0.021525	0.00895	0.010955	0.013462	0.005275	0.379655	0.011757	0.006867	0.018788	0.011495	0.009959	0.011773	0.004377	0.181162
553.50719	Cer d16:0h18:1	Ceramides	C34H67NC	0.018172	0.030759	0.012253	0.023707	0.019399	0.020858	0.006884	0.027876	0.026095	0.026094	0.014826	0.027314	0.024441	0.005431	0.387567	0.040353	0.033297	0.029638	0.020177	0.038546	0.032402	0.008045	0.040688	0.030676	0.02455	0.040721	0.056357	0.041138	0.038688	0.012108	0.027555	0.03707	0.051402	0.022257	0.024166	0.03249	0.01201	0.439897	0.029969	0.024382	0.043523	0.028168	0.028993	0.031007	0.00731	0.259208
555.52274	Cer d16:0h18:0	Ceramides	C34H69NC	0.014086	0.02239	0.01088	0.017652	0.013853	0.015772	0.00441	0.018167	0.016312	0.018154	0.009125	0.016599	0.015672	0.003759	0.969978	0.035782	0.022552	0.022138	0.011985	0.027914	0.024074	0.008718	0.093968	0.016199	0.016859	0.032023	0.035147	0.017912	0.023628	0.009177	0.013851	0.016495	0.023027	0.0094	0.010218	0.014598	0.005509	0.095951	0.009831	0.008179	0.01602	0.009928	0.01263	0.011318	0.003075	0.021669
569.50143	Cer t18:1-16:0	Ceramides	C34H67NC	0.016822	0.030319	0.013555	0.025452	0.019778	0.021185	0.006724	0.040583	0.040075	0.038195	0.02139	0.03668	0.035385	0.007976	0.015977	0.059054	0.040664	0.037314	0.02175	0.050632	0.041883	0.014134	0.018234	0.036605	0.02398	0.042126	0.053371	0.050463	0.041309	0.011752	0.037508	0.047185	0.062238	0.027243	0.029093	0.040653	0.014422	0.939138	0.040209	0.030385	0.056896	0.035431	0.038268	0.040238	0.01002	0.880591
571.51723	Cer t18:0h16:0	Ceramides	C34H69NC	0.014668	0.020908	0.009811	0.020602	0.013297	0.015857	0.00481	0.025637	0.027524	0.025651	0.012565	0.025845	0.023445	0.006133	0.061179	0.043696	0.03321	0.031722	0.014362	0.035456	0.031689	0.010734	0.016826	0.010187	0.010122	0.018942	0.020246	0.014174	0.014734	0.004753	0.009968	0.013627	0.025369	0.009002	0.009608	0.013515	0.006869	0.752522	0.010232	0.007918	0.018564	0.009169	0.010303	0.011237	0.004209	0.253058
581.53789	Cer d18:0h18:1	Ceramides	C36H71NC	0.003453	0.008579	0.003194	0.006225	0.003646	0.005019	0.002334	9.79E-05	0.006247	0.005465	0.002103	0.006611	0.004105	0.002863	0.594945	0.016113	0.005289	0.005211	0.00424	0.007121	0.007595	0.004874	0.317691	0.003378	0.00295	0.005354	0.007975	0.005765	0.005084	0.002022	0.003922	0.004847	0.007715	0.002714	0.003481	0.004536	0.001937	0.67307	0.003072	0.00245	0.005463	0.004984	0.003872	0.003968	0.001263	0.325881
609.56915	Cer d18:1h20:0	Ceramides	C38H75NC	0.023798	0.018118	0.024094	0.023024	0.012075	0.020222	0.005159	0.025378	0.024192	0.031211	0.010544	0.028394	0.023944	0.007973	0.406374	0.040531	0.031886	0.030271	0.017995	0.026603	0.029457	0.008196	0.065549	0.017458	0.012324	0.037077	0.031934	0.019303	0.023619	0.010421	0.021204	0.028561	0.027215	0.0197	0.023261	0.023988	0.003808	0.94252	0.02975	0.0168	0.029547	0.018367	0.015469	0.021986	0.007069	0.779242
625.56414	Cer t18:1h20:0	Ceramides	C38H75NC	0.006501	0.008511	0.004374	0.007401	0.00542	0.006441	0.001622	0.012531	0.012254	0.010913	0.006068	0.01011	0.010375	0.002603	0.020886	0.020656	0.012693	0.012279	0.007885	0.015648	0.013832	0.004715	0.010632	0.011954	0.00523	0.012881	0.013456	0.01074	0.010852	0.003306	0.008515	0.012422	0.015666	0.008732	0.006703	0.010408	0.003599	0.843819	0.009069	0.005857	0.014595	0.009351	0.010897	0.009954	0.003177	0.672749
651.57974	Cer t18:2h22:0	Ceramides	C40H77NC	0.003858	0.007504	0.004225	0.006645	0.006103	0.005667	0.001571	0.013182	0.01036	0.010257	0.005125	0.013075	0.0104	0.003269	0.019353	0.020606	0.011556	0.015412	0.008061	0.014441	0.014015	0.004665	0.005299	0.006784	0.005973	0.011341	0.01173	0.012786	0.009723	0.003112	0.007775	0.009667	0.012994	0.004777	0.006476	0.008338	0.003159	0.504699	0.008556	0.006249	0.012437	0.007538	0.009629	0.008882	0.002348	0.642511
651.65241	Cer d18:0h24:0	Ceramides	C42H85NC	0.006504	0.008894	0.004114	0.007788	0.005708	0.006602	0.001848	0.009165	0.005944	0.009384	0.005003	0.006667	0.007233	0.001957	0.614291	0.011991	0.008852	0.009542	0.005616	0.010908	0.009382	0.00243	0.076108	0.008869	0.005535	0.009349	0.009715	0.005885	0.007871	0.001999	0.007382	0.011855	0.00971	0.006503	0.004611	0.008012	0.002824	0.929489	0.008137	0.004296	0.009472	0.006937	0.007383	0.007245	0.001908	0.626312
653.5954	Cer t18:1h22:0	Ceramides	C40H79NC	0.094807	0.12877	0.071055	0.108084	0.079233	0.09639	0.023043	0.175104	0.170666	0.154345	0.081754	0.156538	0.147681	0.037913	0.032359	0.303717	0.185168	0.186522	0.101746	0.206652	0.196761	0.072119	0.018025	0.11974	0.080837	0.177808	0.173447	0.149876	0.140342	0.040484	0.16729	0.198765	0.241037	0.104505	0.1093	0.164179	0.058493	0.475125	0.148091	0.107256	0.193443	0.129863	0.132525	0.142236	0.032122	0.936694
655.61099	Cer t18:0h22:0	Ceramides	C40H81NC	0.018789	0.034689	0.010464	0.023027	0.022391	0.021872	0.00874	0.035354	0.032686	0.038809	0.020484	0.035826	0.032632	0.00713	0.065472	0.078937	0.044891	0.046974	0.031979	0.05959	0.052474	0.017739	0.008564	0.019954	0.012829	0.030271	0.035143	0.023927	0.024425	0.008714	0.024029	0.021559	0.031478	0.012999	0.015042	0.021021	0.007396	0.52424	0.01176	0.010556	0.020903	0.013613	0.013441	0.014054	0.004031	0.042168
663.61587	Cer d18:2h24:0	Ceramides	C42H81NC	0.13322	0.185592	0.124968	0.166788	0.11696	0.145505	0.029357	0.155317	0.168951	0.165402	0.083361	0.168162	0.148239	0.036673	0.899688	0.299696	0.204504	0.185127	0.129228	0.221514	0.208014	0.06191	0.075679	0.127208	0.102813	0.150495	0.173625	0.151593	0.141147	0.026996	0.155637	0.164265	0.191512	0.105594	0.110238	0.145449	0.036769	0.838234	0.160261	0.106074	0.205603	0.146021	0.14353	0.152298	0.035909	0.594057
665.59501	Cer t18:2h23:0	Ceramides	C41H79NC	0.026666	0.027797	0.028643	0.026055	0.017919	0.025416	0.004308	0.045787	0.043932	0.044197	0.020588	0.035781	0.038057	0.010516	0.037681	0.058848	0.050927	0.056747	0.029403	0.044954	0.048176	0.011808	0.00369	0.029685	0.024748	0.043144	0.04258	0.03902	0.035836	0.008213	0.042439	0.040598	0.055225	0.03003	0.03195	0.040048	0.010029	0.488109	0.049547	0.032971	0.057241	0.035564	0.034662	0.041997	0.010794	0.339454
665.63156	Cer d18:1h24:0	Ceramides	C42H83NC	0.123625	0.215505	0.083747	0.161865	0.135065	0.143962	0.048881	0.201677	0.167833	0.193074	0.100021	0.179298	0.168381	0.040345	0.414038	0.325515	0.197664	0.183558	0.12826	0.257201	0.21844	0.075413	0.100992	0.213181	0.119029	0.18615	0.229284	0.227287	0.194986	0.045821	0.169288	0.220236	0.306107	0.135296	0.133001	0.192786	0.072503	0.955656	0.161028	0.137245	0.259084	0.170527	0.182324	0.182041	0.046142	0.66803
667.61087	Cer t18:1h23:0	Ceramides	C41H81NC	0.036262	0.066264	0.018782	0.04396	0.035235	0.0401	0.017265	0.063623	0.06613	0.06732	0.042053	0.066554	0.061136	0.010758	0.049511	0.157808	0.085991	0.088575	0.057213	0.110798	0.100077	0.03747	0.011688	0.059674	0.035349	0.069435	0.081307	0.076565	0.064466	0.018203	0.07476	0.06941	0.102603	0.047412	0.046027	0.068043	0.023193	0.793063	0.049875	0.037548	0.078879	0.049486	0.050418	0.053241	0.015305	0.322081
669.62645	Cer t18:0h23:0	Ceramides	C41H83NC	0.005531	0.010344	0.000206	0.005902	0.010716	0.00654	0.004285	0.01275	0.011914	0.01145	0.006051	0.008714	0.010176	0.002758	0.149278	0.019747	0.009453	0.011882	0.005536	0.01685	0.012694	0.005688	0.089416	0.010041	0.00466	0.013471	0.01266	0.008716	0.00991	0.003509	0.009629	0.010446	0.010237	0.006828	6.84E-05	0.007442	0.004369	0.353555	0.007362	0.007256	0.013442	0.009224	0.008446	0.009146	0.002535	0.703541
675.57964	Cer t18:1h24:3	Ceramides	C42H77NC	0.001476	0.002091	0.000206	0.002015	0.001573	0.001472	0.000757	0.003835	0.002708	0.003128	0.001707	0.002863	0.002848	0.000771	0.021529	0.006735	0.002499	0.002753	0.001024	0.002728	0.003148	0.002129	0.135953	0.003274	0.002189	0.003518	0.004824	0.005701	0.003901	0.001375	0.002526	0.003824	0.005584	0.002682	0.002404	0.003404	0.001344	0.579192	0.003892	0.003334	0.007218	0.004108	0.004312	0.004573	0.001523	0.484968
677.63128	Cer d18:2h25:0	Ceramides	C43H83NC	0.010994	0.021597	0.010418	0.018231	0.01328	0.014904	0.004846	0.02365	0.022106	0.022956	0.009714	0.020814	0.019848	0.005763	0.180236	0.043278	0.029053	0.028256	0.015881	0.031551	0.029604	0.009762	0.016662	0.018909	0.016914	0.022626	0.029971	0.02312	0.022308	0.005004	0.021152	0.022498	0.026233	0.014807	0.016123	0.020163	0.004698	0.504432	0.018967	0.015593	0.024667	0.018014	0.016706	0.018789	0.003526	0.234637
679.6113	Cer t18.2h24.0	Ceramides	C42H81NC	0.297559	0.448978	0.214917	0.391602	0.284236	0.327458	0.092614	0.622348	0.597585	0.620747	0.288554	0.53046	0.531939	0.141075	0.026683	0.918271	0.599866	0.574707	0.32356	0.695576	0.622396	0.215048	0.02261	0.405294	0.258769	0.434136	0.692981	0.624226	0.483081	0.175173	0.50263	0.58977	0.779096	0.323725	0.362344	0.511513	0.183929	0.808665	0.469112	0.34987	0.680623	0.453746	0.49814	0.490298	0.120198	0.941316
681.62716	Cer t18:1h24:0	Ceramides	C42H83NC	0.145792	0.238379	0.094248	0.195045	0.154099	0.165513	0.054267	0.293974	0.273802	0.286228	0.157978	0.260473	0.254491	0.055429	0.033391	0.457062	0.285878	0.295474	0.17007	0.358141	0.313325	0.105194	0.023473	0.258408	0.147052	0.277034	0.32914	0.315867	0.2655	0.072111	0.274019	0.33764	0.498637	0.197504	0.200975	0.301755	0.124343	0.588213	0.251016	0.194644	0.383882	0.247896	0.247222	0.264932	0.070511	0.990258
683.64133	Cer t18:0h24:0	Ceramides	C42H85NC	0.026347	0.05613	0.020469	0.043356	0.053867	0.040034	0.016059	0.059029	0.054869	0.061047	0.034957	0.05812	0.053604	0.01066	0.154073	0.112616	0.064962	0.063466	0.035529	0.085376	0.07239	0.028637	0.058664	0.035315	0.02356	0.052628	0.066456	0.043041	0.0442	0.016376	0.040412	0.046235	0.071887	0.028054	0.026244	0.042566	0.018404	0.88577	0.029459	0.029528	0.057726	0.034148	0.03308	0.036788	0.011891	0.436506
691.64709	Cer d18:2h26:0	Ceramides	C44H85NC	0.043521	0.089175	0.027198	0.055778	0.044765	0.052087	0.023106	0.073313	0.071042	0.074367	0.037582	0.077401	0.066741	0.01646	0.281419	0.099082	0.069311	0.067529	0.045569	0.086546	0.073608	0.020359	0.156776	0.081611	0.041641	0.075448	0.093547	0.083606	0.075171	0.019841	0.063553	0.085943	0.108868	0.050163	0.066545	0.075014	0.022838	0.991063	0.070945	0.056889	0.111298	0.063353	0.070216	0.07454	0.021328	0.962579
693.62651	Cer t18:1h25:1	Ceramides	C43H83NC	0.063313	0.079039	0.062548	0.063802	0.048133	0.063367	0.010938	0.103065	0.120117	0.106091	0.05559	0.101236	0.09722	0.024425	0.022201	0.154586	0.110942	0.115742	0.058544	0.114197	0.110802	0.034219	0.018355	0.062147	0.044054	0.086196	0.099234	0.087939	0.075914	0.022354	0.08709	0.122098	0.128034	0.06391	0.060462	0.092319	0.031669	0.3717	0.091301	0.061029	0.11324	0.076645	0.077245	0.083892	0.019593	0.565025
693.6637	Cer d18:1h26:0	Ceramides	C44H87NC	0.048517	0.083802	0.042573	0.075438	0.065997	0.063266	0.017486	0.088732	0.085999	0.097167	0.056686	0.083713	0.082459	0.015281	0.101764	0.128046	0.084104	0.070422	0.046518	0.109367	0.087691	0.032034	0.172875	0.10494	0.062388	0.098145	0.128266	0.101314	0.099011	0.023655	0.087047	0.098659	0.198874	0.076069	0.060722	0.104274	0.054703	0.848375	0.088723	0.085339	0.155785	0.101347	0.086693	0.103578	0.02987	0.795466
695.64215	Cer t18:1h25:0	Ceramides	C43H85NC	0.011266	0.022346	0.007881	0.0162	0.013593	0.014257	0.00546	0.026724	0.023758	0.026837	0.015739	0.022842	0.02318	0.004521	0.022675	0.051097	0.025798	0.024821	0.016706	0.03479	0.030642	0.013107	0.032592	0.022606	0.014198	0.019569	0.030374	0.025214	0.022392	0.006059	0.021895	0.02712	0.040329	0.016242	0.016715	0.02446	0.009912	0.701012	0.01676	0.01568	0.032708	0.021777	0.020417	0.021468	0.006768	0.825805
695.67857	Cer d18:0h26:0	Ceramides	C44H89NC	0.095973	0.160911	0.071774	0.127169	0.127569	0.116679	0.034023	0.135601	0.123119	0.157167	0.094976	0.130334	0.128239	0.022517	0.544037	0.232216	0.164357	0.126937	0.083835	0.199868	0.161443	0.058539	0.17758	0.103248	0.067207	0.11917	0.100648	0.091986	0.096452	0.019073	0.078273	0.097748	0.163403	0.066877	0.050501	0.09136	0.043793	0.817609	0.074429	0.066289	0.116903	0.082506	0.074336	0.082893	0.019858	0.30286
697.65737	Cer t18:0h25:0	Ceramides	C43H87NC	0.004198	0.008174	0.003078	0.007431	0.011799	0.006936	0.003457	0.007724	0.007728	0.009211	0.005781	0.009719	0.008033	0.00154	0.535229	0.011318	0.00957	0.010185	0.00437	0.012209	0.009531	0.003059	0.2443	0.007042	0.005741	0.011481	0.013317	0.005416	0.008599	0.003581	0.008031	0.010564	0.010707	0.006041	0.004799	0.008029	0.002645	0.781695	0.007095	0.007225	0.011177	0.009827	0.005998	0.008264	0.002151	0.862223
707.64213	Cer t18:1h26:1	Ceramides	C44H85NC	0.07154	0.148741	0.055515	0.10805	0.089144	0.094598	0.03606	0.181761	0.185307	0.199999	0.102683	0.200531	0.174056	0.040786	0.011466	0.303673	0.173941	0.174552	0.107658	0.234797	0.198924	0.073836	0.021847	0.141419	0.088705	0.157708	0.217427	0.211543	0.163361	0.053225	0.145619	0.173651	0.271036	0.116779	0.117834	0.164984	0.063726	0.966204	0.144844	0.116877	0.232637	0.147861	0.14326	0.157096	0.04402	0.844339
709.65925	Cer t18:1h26:0	Glycosylcer	C44H87NC	0.056548	0.097948	0.037565	0.089331	0.076543	0.071587	0.024587	0.144858	0.13968	0.156655	0.088104	0.131806	0.132221	0.026262	0.005475	0.228591	0.141393	0.122148	0.06967	0.167113	0.145783	0.058488	0.030891	0.11368	0.071872	0.117222	0.139683	0.127566	0.114005	0.025637	0.113718	0.1386	0.215982	0.084684	0.076201	0.125837	0.05611	0.679314	0.097945	0.08855	0.165462	0.111528	0.098645	0.112426	0.030754	0.93191
711.67423	Cer t18:0h26:0	Ceramides	C44H89NC	0.017372	0.031352	0.014525	0.030458	0.033791	0.0255	0.008861	0.036387	0.03924	0.045119	0.028306	0.03704	0.037218	0.006055	0.040467	0.085322	0.053622	0.042793	0.02351	0.069508	0.054951	0.023835	0.032123	0.020226	0.012902	0.020823	0.026326	0.020195	0.020094	0.004773	0.021337	0.020883	0.034676	0.015402	0.012519	0.020963	0.008521	0.847201	0.016077	0.012831	0.027193	0.019011	0.017274	0.018477	0.00537	0.628352
646.56836	CE 18:3	Cholestrol	C45H74O2	0.002894	0.004255	0.002103	0.003838	0.00322	0.003262	0.000837	0.003828	0.003094	0.002562	0.00138	0.003388	0.00285	0.000942	0.485884	0.00853	0.005194	0.005094	0.003409	0.007098	0.005865	0.001981	0.026797	0.004506	0.003294	0.004835	0.005862	0.004254	0.00455	0.000931	0.005922	0.006154	0.007082	0.003866	0.003612	0.005327	0.001516	0.357393	0.004988	0.002355	0.005799	0.003149	0.003801	0.004019	0.001386	0.496615
648.58405	CE 18:2	Cholestrol	C45H76O2	0.005647	0.004748	0.001683	0.003365	0.002653	0.003619	0.001592	0.005308	0.004367	0.004325	0.002582	0.003857	0.004088	0.000993	0.591607	0.005989	0.005448	0.004136	0.003486	0.005918	0.004995	0.001125	0.152974	0.008211	0.004083	0.006875	0.005699	0.004116	0.005797	0.001786	0.004622	0.007335	0.006972	0.00498	0.003419	0.005466	0.001651	0.768613	0.006411	0.001994	0.005927	0.005114	0.005021	0.004893	0.001721	0.438865
650.59942	CE 18:1	Cholestrol	C45H78O2	0.007225	0.004034	0.00236	0.003365	0.002065	0.00381	0.002065	0.006161	0.006127	0.005998	0.002639	0.003957	0.004976	0.001603	0.347518	0.00444	0.004651	0.004444	0.004103	0.005768	0.004681	0.000639	0.393718	0.00795	0.003899	0.006203	0.005161	0.003503	0.005343	0.001805	0.005783	0.008118	0.005893	0.006277	0.003888	0.005992	0.001507	0.554649	0.007317	0.002068	0.008164	0.004918	0.006494	0.005792	0.002402	0.746985
536.44391	DG 14:1-16:1	Diacylglyce	C33H60O5	0.003514	6.68E-05	0.000206	5.89E-05	3.95E-05	0.000777	0.001531	9.79E-05	0.003106	0.002617	4.29E-05	7.84E-05	0.001188	0.001537	0.682896	0.000116	8.25E-05	9.09E-05	4.85E-05	8.68E-05	0.000085	2.43E-05	0.341861	0.004441	0.001871	0.002242	0.002841	8.81E-05	0.002297	0.001578	0.002403	0.000125	0.000108	0.003065	0.001628	0.001466	0.001333	0.394537	0.000134	6.87E-05	0.003192	0.002175	9.26E-05	0.001132	0.001461	0.260572
538.45928	DG 14:0-16:1	Diacylglyce	C33H62O5	0.004945	0.002936	0.001523	0.003476	0.001004	0.002777	0.001576	9.79E-05	0.008015	0.004924	0.002255	0.00411	0.003881	0.002966	0.483324	0.004039	0.002209	0.004512	0.003289	0.005939	0.003998	0.001391	0.230097	0.007137	0.004053	0.006547	0.006835	0.003969	0.005708	0.001564	0.005513	0.006102	0.005077	0.005737	0.003494	0.005184	0.001015	0.547417	0.003553	0.001905	0.006539	0.004709	0.004381	0.004217	0.001692	0.185923
540.47521	DG 14:0-16:0	Diacylglyce	C33H64O5	0.005483	0.004764	0.002991	0.003819	0.001858	0.003783	0.001431	0.006253	0.008098	0.006488	0.004313	0.00518	0.006066	0.001432	0.035677	0.006198	0.005542	0.005722	0.004781	0.00791	0.006031	0.001168	0.026197	0.008964	0.004272	0.007768	0.010737	0.006402	0.007628	0.002461	0.005939	0.009125	0.007739	0.007064	0.004523	0.006878	0.00175	0.593605	0.005829	0.002603	0.009254	0.006216	0.007123	0.006205	0.002411	0.38265
552.47457	DG 15:0-16:1	Diacylglyce	C34H64O5	0.00632	6.68E-05	0.001549	0.002991	3.95E-05	0.002193	0.00261	9.79E-05	0.006784	0.004677	0.002709	0.003552	0.003564	0.002467	0.418185	0.000116	0.004368	9.09E-05	0.003438	8.68E-05	0.00162	0.00211	0.712568	0.0081	0.003037	0.007191	0.006933	8.81E-05	0.00507	0.003395	0.006793	0.009496	0.000108	0.006106	0.004448	0.00539	0.003469	0.886288	0.000134	6.87E-05	0.007739	0.00556	9.26E-05	0.002719	0.00367	0.323715
554.49037	DG 15:0-16:0	Diacylglyce	C34H66O5	0.004709	0.004071	0.000206	0.003505	0.001759	0.00285	0.001841	0.0051	0.006689	0.005538	0.002229	0.004327	0.004777	0.001661	0.120482	0.004723	0.003903	0.004875	0.00346	0.005235	0.004439	0.000733	0.110706	0.006321	0.003651	0.004735	0.006653	0.004572	0.005186	0.001263	0.003774	0.006738	0.004622	0.005124	0.003207	0.004693	0.001363	0.569254	0.004051	0.002307	0.007794	0.005814	0.004451	0.004884	0.002053	0.785934
562.45964	DG 16:0-16:3	Diacylglyce	C35H62O5	0.002565	0.004876	0.000206	0.003564	0.002447	0.002732	0.001716	0.006138	0.005344	0.005808	0.003464	0.003926	0.004936	0.001179	0.0454	0.007075	0.007182	0.005215	0.004068	0.009056	0.006519	0.00193	0.011189	0.006839	0.006099	0.008497	0.011905	0.008709	0.00841	0.002243	0.00517	0.007612	0.008699	0.005331	0.004963	0.006355	0.001693	0.140665	0.012427	0.00786	0.01218	0.007101	0.009574	0.009828	0.002432	0.365709
564.47524	DG 16:1-16:1	Diacylglyce	C35H64O5	0.006597	0.003359	0.002312	0.004589	0.001855	0.003742	0.001912	0.005941	0.008668	0.00588	0.002256	0.004234	0.005396	0.002369	0.259177	0.006997	0.004478	0.00631	0.003334	0.009494	0.006123	0.002379	0.119373	0.009157	0.004642	0.006551	0.010512	0.005661	0.007305	0.002453	0.006579	0.007576	0.007312	0.006901	0.00385	0.006444	0.001499	0.521841	0.006674	0.001679	0.009543	0.006524	0.006343	0.006153	0.002827	0.510699
566.49079	DG 16:0-16:1	Diacylglyce	C35H66O5	0.018543	0.010533	0.006006	0.010784	0.00572	0.010317	0.005188	0.016173	0.02232	0.018599	0.009531	0.01318	0.015961	0.004912	0.115343	0.01313	0.015437	0.017421	0.012192	0.022137	0.016063	0.003959	0.084491	0.029107	0.012017	0.022828	0.023907	0.015434	0.020659	0.006867	0.020431	0.028373	0.019441	0.022192	0.011183	0.020324	0.006177	0.937424	0.013657	0.007794	0.026184	0.020166	0.018453	0.017251	0.00693	0.457225
568.50603	DG 16:0-16:0	Diacylglyce	C35H68O5	0.027348	0.021705	0.014275	0.017421	0.014383	0.019026	0.005548	0.026028	0.035078	0.024338	0.025026	0.022223	0.026539	0.004973	0.054188	0.049979	0.025381	0.034137	0.019233	0.031726	0.032091	0.011561	0.052229	0.036056	0.019198	0.02853	0.062068	0.03725	0.03662	0.015948	0.030796	0.036719	0.051934	0.025978	0.020344	0.033154	0.012109	0.708796	0.029535	0.015078	0.055119	0.042244	0.0465	0.037695	0.015652	0.916999
582.52147	DG 16:0-17:0	Diacylglyce	C36H70O5	0.004422	6.68E-05	0.002728	5.89E-05	3.95E-05	0.001463	0.002019	0.003627	0.008175	0.003824	0.002754	7.84E-05	0.003692	0.002918	0.197822	0.000116	8.25E-05	9.09E-05	4.85E-05	8.68E-05	0.000085	2.43E-05	0.165487	0.007675	5.37E-05	0.000102	1E-04	8.81E-05	0.001604	0.003394	0.000117	0.005931	0.000108	0.004544	6.84E-05	0.002154	0.002858	0.788616	0.004058	6.87E-05	0.008004	0.005714	0.00413	0.004395	0.002901	0.199679

584.44385	DG 16:3-18:3	Diacylglyce	C37H60O5	0.006467	0.008742	0.009241	0.010114	0.009111	0.008735	0.001364	0.014444	0.010891	0.009707	0.008012	0.009735	0.010558	0.002403	0.178351	0.01984	0.011393	0.01262	0.007148	0.018213	0.013843	0.005181	0.065611	0.010223	0.009537	0.010395	0.022066	0.014543	0.013353	0.005255	0.007341	0.007542	0.011096	0.00584	0.004516	0.007267	0.002467	0.047092	0.00987	0.008801	0.015776	0.014263	0.024207	0.014584	0.00612	0.741807
586.45927	DG 16:3-18:2	Diacylglyce	C37H62O5	0.001453	0.002978	0.001247	0.002099	0.002698	0.002095	0.000754	0.004248	0.00375	0.003831	0.002402	0.003143	0.003475	0.000718	0.018038	0.007158	0.003991	0.005898	0.002707	0.008597	0.00567	0.002368	0.012303	0.005634	0.003393	0.005333	0.01002	0.006975	0.006271	0.002456	0.004762	0.005078	0.006676	0.002995	0.003556	0.004613	0.001434	0.228718	0.0049	0.004485	0.008835	0.005302	0.007736	0.006251	0.001919	0.989091
588.47507	DG 16:1-18:3	Diacylglyce	C37H64O5	0.006536	0.011527	0.006103	0.008677	0.007961	0.008161	0.002151	0.014223	0.013286	0.010974	0.007533	0.011823	0.011568	0.002584	0.053232	0.022637	0.018288	0.018829	0.012392	0.033811	0.021192	0.00795	0.007643	0.016497	0.013156	0.020293	0.030931	0.024908	0.021157	0.007001	0.013236	0.019929	0.034539	0.013138	0.014139	0.018996	0.009132	0.685639	0.018028	0.01933	0.026803	0.016863	0.021576	0.02052	0.003924	0.863524
590.49035	DG 16:1-18:2	Diacylglyce	C37H66O5	0.006043	0.003789	0.000206	0.004259	0.003003	0.00346	0.002134	0.004638	0.004911	0.005554	0.002095	0.004046	0.004249	0.001321	0.502218	0.010769	0.004973	0.009265	0.002646	0.005077	0.006546	0.003357	0.12101	0.007193	0.005532	0.010319	0.015385	0.007075	0.009101	0.00392	0.005717	0.006548	0.010102	0.005981	0.006158	0.006901	0.001815	0.287706	0.009713	0.0049	0.01803	0.011003	0.009779	0.010685	0.004724	0.579836
590.49043	DG 16:0-18:3	Diacylglyce	C37H66O5	0.158579	0.237725	0.145683	0.235092	0.180814	0.191579	0.04282	0.363083	0.363371	0.352826	0.204875	0.321664	0.321164	0.067202	0.006624	0.548184	0.392293	0.383803	0.236406	0.54541	0.421219	0.130323	0.005679	0.41459	0.321744	0.498997	0.769503	0.571191	0.515205	0.170011	0.36877	0.482239	0.661691	0.339127	0.317269	0.433819	0.142348	0.435575	0.606919	0.380516	0.760778	0.447705	0.627053	0.564594	0.151434	0.640633
592.50625	DG 16:1-18:1	Diacylglyce	C37H68O5	0.191132	0.273215	0.164417	0.259107	0.191586	0.215891	0.047452	0.39117	0.401676	0.38737	0.210956	0.332549	0.344744	0.07947	0.014383	0.596834	0.458234	0.420397	0.26112	0.603434	0.468004	0.141511	0.005411	0.431884	0.320604	0.499038	0.70313	0.541565	0.499244	0.141254	0.354103	0.456388	0.616848	0.313898	0.306938	0.409635	0.130306	0.327596	0.552975	0.383295	0.808178	0.502238	0.541161	0.55757	0.155345	0.551784
592.50635	DG 16:0-18:2	Diacylglyce	C37H68O5	0.136176	0.127377	0.193196	0.135798	0.086138	0.135737	0.038183	0.212466	0.235162	0.225717	0.117197	0.201486	0.198406	0.047167	0.049754	0.256324	0.184918	0.168945	0.096819	0.203838	0.182169	0.057956	0.173029	0.161653	0.111653	0.196954	0.199221	0.155305	0.164957	0.035858	0.175325	0.19272	0.213223	0.136942	0.141279	0.171898	0.032835	0.757753	0.298299	0.156327	0.317563	0.221203	0.190538	0.236786	0.069217	0.073318
594.52194	DG 16:0-18:1	Diacylglyce	C37H70O5	0.038464	0.036004	0.024012	0.033351	0.023469	0.03106	0.006925	0.040909	0.047536	0.048202	0.032327	0.057719	0.045339	0.009424	0.025838	0.050688	0.048143	0.050109	0.026828	0.059603	0.047074	0.012148	0.0336	0.065754	0.040302	0.064257	0.101648	0.066545	0.067701	0.02191	0.053065	0.070283	0.111443	0.052288	0.032458	0.063908	0.029757	0.82419	0.058458	0.034918	0.109926	0.086642	0.084965	0.074982	0.028874	0.665249
596.53746	DG 16:0-18:0	Diacylglyce	C37H72O5	0.042901	0.042702	0.015688	0.031113	0.021453	0.030772	0.012288	0.062804	0.046365	0.041499	0.025343	0.041629	0.043528	0.013398	0.155283	0.059557	0.041364	0.036111	0.021708	0.04011	0.03977	0.013544	0.303232	0.051394	0.045806	0.071162	0.076605	0.05527	0.060047	0.013212	0.065628	0.072156	0.069964	0.044897	0.056353	0.061799	0.011224	0.82688	0.078277	0.051056	0.074662	0.05034	0.054392	0.061745	0.013588	0.846226
604.50606	DG 17:0-18:3	Diacylglyce	C38H68O5	0.002022	0.002517	0.001523	0.002236	0.001362	0.001932	0.000483	0.003233	0.003439	0.003278	0.001787	0.002867	0.002921	0.000668	0.027807	0.004922	0.004546	0.003531	0.00184	0.00492	0.003952	0.00131	0.011994	0.004173	0.003343	0.004987	0.007707	0.005475	0.005137	0.00165	0.002575	0.003936	0.006331	0.003441	0.002778	0.003812	0.001508	0.221583	0.004326	0.003102	0.007301	0.004548	0.005864	0.005028	0.001604	0.918232
612.47497	DG 18:3-18:3	Diacylglyce	C39H64O5	0.081813	0.08439	0.09817	0.093448	0.065005	0.084565	0.012789	0.171158	0.137837	0.15053	0.080343	0.123209	0.132616	0.034112	0.018446	0.208031	0.140302	0.152928	0.075	0.170291	0.149311	0.048746	0.02074	0.129563	0.107392	0.171997	0.264418	0.160865	0.166847	0.060223	0.144316	0.135628	0.167934	0.099276	0.103808	0.130193	0.028746	0.254271	0.190133	0.126141	0.229199	0.140867	0.17834	0.172936	0.040953	0.856357
614.49064	DG 18:2-18:3	Diacylglyce	C39H66O5	0.037203	0.056603	0.031578	0.05958	0.044701	0.045933	0.012082	0.082984	0.078889	0.08208	0.052043	0.076159	0.074431	0.012804	0.006785	0.142784	0.092479	0.088806	0.057785	0.14528	0.105427	0.037741	0.009975	0.093009	0.067732	0.094789	0.152396	0.116112	0.104808	0.031649	0.066375	0.076709	0.12905	0.058512	0.058201	0.077769	0.029641	0.20073	0.108655	0.086815	0.155036	0.092226	0.105782	0.109703	0.02693	0.798896
616.50625	DG 18:2-18:2	Diacylglyce	C39H68O5	0.05014	0.047681	0.091059	0.046402	0.036927	0.054442	0.021074	0.066401	0.075886	0.074889	0.036005	0.0636	0.063356	0.016182	0.474606	0.083169	0.076242	0.083181	0.043765	0.092338	0.075739	0.018766	0.129961	0.054804	0.039683	0.069457	0.102973	0.064795	0.066342	0.02344	0.069323	0.061834	0.097097	0.037503	0.045967	0.062345	0.023141	0.792963	0.085016	0.045304	0.095277	0.05199	0.074943	0.070506	0.021341	0.776466
618.52191	DG 18:1-18:2	Diacylglyce	C39H70O5	0.020601	0.028317	0.018402	0.025437	0.020223	0.022596	0.004123	0.030106	0.031342	0.031247	0.021062	0.029824	0.028716	0.004332	0.051394	0.048948	0.035782	0.034097	0.022713	0.049797	0.038267	0.01132	0.019626	0.034583	0.025856	0.038019	0.063276	0.048606	0.042068	0.014386	0.030398	0.036097	0.076587	0.027224	0.023196	0.0387	0.021698	0.779736	0.03584	0.027483	0.062414	0.038038	0.040161	0.040787	0.013011	0.886262
620.53744	DG 18:1-18:1	Diacylglyce	C39H72O5	0.021595	0.014112	0.015087	0.011249	0.0118	0.014768	0.004133	0.019469	0.030517	0.033141	0.010925	0.034874	0.025785	0.010245	0.056304	0.024083	0.016897	0.026652	0.007115	0.021355	0.019221	0.007671	0.286284	0.029417	0.010448	0.018217	0.053596	0.036519	0.029639	0.016734	0.016657	0.019858	0.049801	0.014369	0.017299	0.023597	0.014779	0.56181	0.024102	0.011102	0.073781	0.044401	0.054249	0.041527	0.024697	0.398918
622.55259	DG 18:0-18:1	Diacylglyce	C39H74O5	0.008303	0.007134	0.004215	0.005498	0.00382	0.005794	0.001909	0.009296	0.008771	0.009006	0.006338	0.015581	0.009798	0.003441	0.052411	0.010485	0.009684	0.007905	0.00619	0.012522	0.009357	0.002425	0.032536	0.012137	0.007187	0.010225	0.02179	0.016921	0.013652	0.005761	0.00901	0.013591	0.018545	0.00863	0.006012	0.011157	0.004949	0.483654	0.010288	0.006011	0.025028	0.017051	0.018635	0.015403	0.007419	0.687802
624.5691	DG 18:0-18:0	Diacylglyce	C39H76O5	0.027572	0.026201	0.007731	0.019095	0.006733	0.017467	0.009888	0.048242	0.027357	0.023665	0.006655	0.022243	0.025633	0.014914	0.337375	0.014757	0.021399	0.01041	0.006412	0.0109	0.012776	0.005655	0.38404	0.032469	0.033426	0.060232	0.025989	0.017197	0.033863	0.016103	0.046743	0.042563	0.018544	0.028111	0.038862	0.034964	0.011492	0.903969	0.046959	0.030176	0.023263	0.013792	0.01442	0.025722	0.013665	0.413831
652.59915	DG 14:0-24:0	Diacylglyce	C41H80O5	0.004689	0.00443	0.002985	0.003352	0.001744	0.00344	0.001186	0.006849	0.004876	0.004658	0.002204	0.004853	0.004688	0.001651	0.207158	0.004408	0.004212	0.004253	0.002452	0.005363	0.004138	0.001053	0.354122	0.007541	0.005664	0.009952	0.006867	0.005279	0.007061	0.001854	0.006554	0.007646	0.00555	0.005672	0.006374	0.006359	0.00084	0.463148	0.006546	0.004166	0.006299	0.00446	0.004875	0.005269	0.001086	0.099345
668.53968	DG 16:0-24:6	Diacylglyce	C43H72O5	0.005297	0.00684	0.000206	0.004156	0.007946	0.004889	0.002991	0.005935	0.001888	0.001909	0.008658	0.003816	0.004441	0.002887	0.815598	0.029187	0.005012	0.021364	0.013531	0.024174	0.018654	0.009502	0.014894	0.010213	0.0058	0.003699	0.037774	0.032124	0.017922	0.015847	0.008924	0.008075	0.033676	0.006038	0.007044	0.012751	0.011747	0.573949	0.011325	0.00424	0.034512	0.028733	0.03677	0.023116	0.014519	0.603642
680.63113	DG 16:0-24:0	Diacylglyce	C43H84O5	0.004773	0.004697	0.004425	0.004653	0.002267	0.004163	0.001068	0.006258	0.005601	0.006273	0.003163	0.004773	0.005213	0.0013	0.200208	0.006564	0.004955	0.003762	0.002556	0.00544	0.004655	0.001545	0.573971	0.007553	0.004381	0.007832	0.006758	0.004902	0.006285	0.001562	0.006278	0.007762	0.006238	0.005577	0.004462	0.006063	0.0012	0.807443	0.007579	0.003858	0.008633	0.006003	0.005321	0.006279	0.001877	0.99542
694.64628	DG 16:0-25:0	Diacylglyce	C44H86O5	0.002622	0.002681	0.002565	0.00255	0.001333	0.00235	0.000571	0.003372	0.003587	0.003342	0.00187	0.003443	0.003123	0.000707	0.093767	0.003754	0.003023	0.00349	0.002092	0.003726	0.003217	0.000694	0.063144	0.005368	0.002464	0.004047	0.004417	0.00321	0.003901	0.001116	0.003706	0.004319	0.00351	0.003661	0.002968	0.003633	0.000483	0.635316	0.003561	0.001689	0.005457	0.004132	0.003754	0.003718	0.001354	0.821745
704.63117	DG 18:2-24:0	Diacylglyce	C45H84O5	0.002239	0.003403	0.000206	0.00287	0.002642	0.002272	0.001229	0.004676	0.004028	0.00412	0.002362	0.00453	0.003943	0.000925	0.041183	0.006584	0.004759	0.005262	0.002707	0.007088	0.00528	0.001722	0.013005	0.006485	0.005135	0.007874	0.011787	0.008056	0.007867	0.002489	0.005815	0.006944	0.011398	0.004741	0.004426	0.006665	0.002825	0.495376	0.005948	0.004952	0.008099	0.006186	0.006794	0.006396	0.001161	0.26506
724.60225	DG 18:0-26:6	Diacylglyce	C47H80O5	0.014429	0.01387	0.000206	0.007841	0.009944	0.009258	0.005754	0.018924	0.005346	0.004626	0.013383	0.010923	0.01064	0.005925	0.71793	0.046145	0.007539	0.031372	0.017083	0.030705	0.026569	0.014794	0.040661	0.017997	0.014164	0.016063	0.046529	0.041053	0.027161	0.015364	0.022884	0.019822	0.04087	0.013811	0.018537	0.023185	0.010412	0.644709	0.022651	0.013316	0.048145	0.0416	0.042356	0.033614	0.014862	0.518715
699.56413	Glc-O-t18:1h16:0	Glycosylcer	C40H77NC	0.005185	0.008689	0.004782	0.007183	0.006311	0.00643	0.001578	0.009227	0.008565	0.007761	0.004692	0.007453	0.007539	0.001736	0.321192	0.015686	0.01001	0.007759	0.008252	0.012378	0.010817	0.003268	0.026951	0.009037	0.005843	0.011971	0.01495	0.011054	0.010571	0.003394	0.008687	0.009739	0.014946	0.006524	0.005853	0.00915	0.003603	0.538839	0.011024	0.006825	0.013542	0.008393	0.008641	0.009685	0.002627	0.656671
715.55953	Glc d18:1h16:0	Glycosylcer	C40H77NC	0.027872	0.036366	0.032997	0.03081	0.021388	0.029887	0.005674	0.043448	0.041548	0.040665	0.022011	0.038739	0.037282	0.008703	0.150117	0.059665	0.04455	0.04072	0.023168	0.050151	0.043651	0.013488	0.068575	0.038741	0.028001	0.052539	0.055583	0.045635	0.0441	0.011105	0.055677	0.057066	0.063747	0.035445	0.034454	0.049278	0.013436	0.525186	0.061612	0.034923	0.059076	0.043185	0.046257	0.049011	0.011181	0.505625
731.55404	Glc t18:1h16:0	Glycosylcer	C40H77NC	0.037914	0.049953	0.026347	0.042617	0.030587	0.037483	0.009402	0.066444	0.070032	0.065152	0.038125	0.066204	0.061191	0.013025	0.010859	0.084316	0.064498	0.055417	0.03297	0.074471	0.062334	0.019655	0.034155	0.106422	0.068143	0.109173	0.15191	0.119679	0.111065	0.030041	0.09231	0.106709	0.147188	0.064557	0.071633	0.096479	0.0329	0.485022	0.129579	0.09128	0.154213	0.093101	0.110535	0.115741	0.026503	0.800675
787.61636	Glc t18:1h20:0	Glycosylcer	C44H85NC	0.008554	0.013474	0.00846	0.011355	0.007916	0.009952	0.002382	0.020046	0.015508	0.015359	0.008323	0.017279	0.015303	0.004335	0.041916	0.039282	0.017004	0.01654	0.01116	0.020486	0.020895	0.010807	0.057979	0.020494	0.012993	0.023424	0.027945	0.021757	0.021323	0.005443	0.017434	0.022567	0.025925	0.012648	0.012911	0.018297	0.005875	0.422868	0.020438	0.014776	0.028078	0.01644	0.021141	0.020175	0.005161	0.741065
799.65293	Glc d18:1h22:0	Glycosylcer	C46H89NC	0.001877	0.003613	0.000206	0.003411	0.002891	0.0024	0.001398	0.001173	0.001607	0.001722	0.000878	0.001776	0.001431	0.000389	0.174083	0.003672	0.005552	0.005655	0.003877	0.007811	0.005313	0.001671	0.017335	0.005256	0.003458	0.00587	0.007179	0.001641	0.004681	0.002164	0.004682	0.005512	0.009237	0.003583	0.004059	0.005415	0.002255	0.61386	0.007599	0.003805	0.007478	0.004431	0.005326	0.005728	0.00174	0.423583
815.6479	Glc t18:1h22:0	Glycosylcer	C46H89NC	0.05355	0.074872	0.049516	0.065615	0.050523	0.058815	0.011034	0.104929	0.098096	0.091624	0.046746	0.085008	0.08528	0.022779	0.047559	0.17859	0.110998	0.106646	0.065815	0.133776	0.119165	0.041275	0.013425	0.118978	0.082214	0.150623	0.173978	0.132207	0.1316	0.034483	0.12312	0.131843	0.179932	0.085132	0.084622	0.12093	0.039385	0.66066	0.144986	0.101817	0.161088	0.113899	0.123769	0.129112	0.023872	0.897746
825.66826	Glc d18:2h24:0	Glycosylcer	C48H91NC	0.016309	0.020606	0.014083	0.019516	0.013786	0.01686	0.003105	0.029342	0.024546	0.0233	0.011747	0.021763	0.022139	0.006464	0.138352	0.041281	0.026433	0.025991	0.01524	0.028258	0.027441	0.009276	0.041941	0.02298	0.014902	0.030344	0.029511	0.026606	0.024868	0.006274	0.025547	0.028933	0.037023	0.016468	0.017425	0.025079	0.008521	0.965586	0.033891	0.020474	0.030511	0.024008	0.028234	0.027424	0.005291	0.506048
839.6484	Glc t18:3h24:0	Glycosylcer	C48H89NC	0.004153	0.005179	0.002808	0.005209	0.003225	0.004115	0.001099	0.008644	0.007693	0.009074	0.004329	0.006883	0.007324	0.001878	0.010881	0.016119	0.007223	0.007301	0.004798	0.010464	0.009181	0.004369	0.036108	0.01085	0.00661	0.012502	0.012326	0.012024	0.010862	0.002463	0.008713	0.012496	0.015907	0.006734	0.00743	0.010256	0.003864	0.774784	0.012374	0.009319	0.015934	0.010731	0.011192	0.01191	0.002502	0.523392
841.6634	Glc t18:2h24:0	Glycosylcer	C48H91NC	0.190339	0.291457	0.139651	0.292852	0.198113	0.222482	0.067449	0.406464	0.398087	0.38258	0.193872	0.333588	0.342918	0.087973	0.041244	0.551648	0.366834	0.340789	0.214768	0.437498	0.382307	0.124253	0.035376	0.388422	0.286456	0.464433	0.664804	0.520446	0.464912	0.142072	0.396139	0.452351	0.66493	0.311972	0.304453	0.425969	0.147012	0.681381	0.538267	0.35203	0.666369	0.422179	0.450152	0.4858	0.121007	0.808687
843.67885	Glc t18:1h24:0	Glycosylcer	C48H93NC	0.286779	0.385397	0.253802	0.31386	0.232498	0.294467	0.059576	0.391154	0.460491	0.464111	0.235443	0.419891	0.394218	0.093748	0.079516	0.605005	0.412929	0.360897	0.226746	0.447413	0.410598	0.137319	0.120995	0.572159	0.400657	0.683221	0.829751	0.592507	0.615659	0.157364	0.654339	0.763346	0.885126	0.411245	0.476323	0.638076	0.196601	0.847189	0.742619	0.501787	0.88327	0.638683	0.527614	0.658795	0.157861	0.676641
853.69978	Glc d18:2h26:0	Glycosylcer	C50H95NC	0.001992	0.002606	0.000206	0.001874	0.00151	0.001638	0.000892	9.79E-05	0.002526	9.08E-05	4.29E-05	0.001856	0.000923	0.001182	0.311893	0.000116	0.002847	0.002528	0.001131	0.003325	0.001989	0.001328	0.636206	0.002096	0.001993	0.003483	0.00396	0.003942	0.003095	0.000978	0.003175	0.000125	0.003534	0.00007	0.002232	0.001827	0.001649	0.177632	0.003633	0.001972	0.004287	0.00269	0.00243	0.003002	0.00094	0.882789
857.69419	Glc t18:1h25:0	Glycosylcer	C49H95NC	0.004224	0.006396	0.002717	0.005387	0.004497	0.004644	0.001372	0.010612	0.010177	0.011406	0.005096	0.011088	0.009676	0.002602	0.005058	0.015371	0.009257	0.007923	0.003755	0.009094	0.00908	0.004162	0.053459	0.010313	0.00732	0.01427	0.01472	0.011311	0.011587	0.003038	0.011763	0.011937	0.016455	0.008664	0.006367	0.011037	0.003812	0.807295	0.013013	0.008313	0.012524	0.008658	0.009576	0.010417	0.002202	0.505411
869.69429	Glc t18:2h26:0	Glycosylcer	C50H95NC	0.033228	0.039114	0.027795	0.03865	0.026323	0.033022	0.005938	0.069427	0.066057	0.064752	0.034256	0.061968	0.059292	0.01425	0.005199	0.071955	0.060825	0.049813	0.02722	0.05824	0.053611	0.01674	0.032018	0.059588	0.041725	0.073131	0.098256	0.069306	0.068401	0.020638	0.071876	0.089702	0.099329	0.044352	0.043983	0.069848	0.025429	0.923722	0.081502	0.057667	0.092121	0.066644	0.07432	0.074451	0.013272	0.596483
871.7103	Glc t18:1h26:0	Glycosylcer	C50H97NC	0.063003	0.115506	0.031704	0.067699	0.062089	0.068	0.030142	0.103722	0.110797	0.111198	0.064026	0.107706	0.09949	0.02005	0.087664	0.150785	0.092646	0.086535	0.061187	0.120254	0.102281	0.034297	0.131704	0.207424	0.095384	0.191276	0.207968	0.153032	0.171017	0.047815	0.131207	0.19796	0.213642	0.104993	0.143502	0.158261	0.045908	0.678331	0.171244	0.127539	0.211036	0.135746	0.144173	0.157947	0.033921	0.631552
495.33226	LPC 16:0	Lysophospl	C24H50NC	0.004407	0.003633	0.003344	0.002015	0.001249	0.002929	0.001276	0.002029	0.00336	0.003968	0.003082	0.002408	0.00297	0.000768	0.953453	0.000116	0.002691	9.09E-05	0.001085	8.68E-05	0.000814	0.001133	0.024221	0.005567	0.002364	0.000102	0.007519	8.81E-05	0.003128	0.003325	0.000117	0.000125	0.000108	0.00007	6.84E-05	9.76E-05	2.66E-05	0.075884	0.000134	0.004249	0.000117	0.006935	0.006826	0.003652	0.003394	0.811451
517.31684	LPC 18:3	Lysophospl	C26H48NC	0.003983	0.003569	0.003742	0.003536	0.001719	0.00331	0.000907	0.004775	0.004752	0.005832	0.005061	0.00496	0.005076	0.000442	0.004452	0.0059	0.005112	0.005001	0.0028	0.005646	0.004892	0.001227	0.04903	0.005127	0.002746	0.00368	0.007332	0.005772	0.004931	0.001792	0.004742	0.003932	0.005466	0.002891	0.00283	0.003972	0.001151	0.343436	0.003826	0.009944	0.014172	0.004342	0.003296	0.007116	0.004767	0.365591
519.33223	LPC 18:2	Lysophospl	C26H50NC	0.0056	0.005151	0.006268	0.004664	0.002386	0.004814	0.00148	0.004989	0.006538	0.007029	0.006426	0.006689	0.006334	0.000786	0.076952	0.008087	0.006998	0.00595	0.003167	0.007471	0.006335	0.001935	0.200247	0.008471	0.003617	0.004812	0.009321	0.008438	0.006932	0.002541	0.007884	0.007994	0.009477	0.00524	0.004474	0.007014	0.002084	0.956898	0.008447	0.01334	0.023791	0.010419	0.00608	0.012415	0.006894	0.133717
521.34783	LPC 18:1	Lysophospl	C26H52NC	0.001941	0.001552	0.001514	0.001584	0.000986	0.001515	0.000342	9.79E-05	9.27E-05	0.00173	0.00104	0.001015	0.000795	0.0007	0.072632	0.000116	0.001346	9.09E-05	0.000648	8.68E-05	0.000458	0.000551	0.006517	0.003147	0.001603	0.000102	0.003903	8.81E-05	0.001769	0.001738	0.002659	0.00311	0.004363	0.002211	0.001713	0.002811	0.001011	0.279769	0.000134	0.004822	0.007931	0.003515	9.26E-05	0.003299	0.003321	0.388017
720.4932	PG 16:0-16:1	Phosphatid	C38H73O1	0.001352	6.68E-05	0.00218	0.002759	0.003435	0.001959	0.001306	9.79E-05	9.27E-05	0.002946	0.003425	0.002338	0.00178	0.001585	0.85049	0.000116	8.25E-05	9.09E-05	0.001283	8.68E-05	0.000332	0.000532	0.032616	0.004872	0.002306	0.000102	1E-04	8.81E-05	0.001494	0.002117	0.000117	0.003358	0.000108	0.002714	0.002273	0.001714	0.001512	0.854412	0.00437	0.003661	0.006052	0.004384	0.002401	0.004174	0.001324	0.043195
722.5105	PG 16:0-16:0	Phosphatid	C38H75O1	0.002923	0.003113	0.002177	0.002723	0.002863	0.00276	0.000355	0.002735	9.27E-05	0.002735	0.003303	0.002761	0.002325	0.001271	0.482926	0.002621	0.002207	0.001223	0.001996	0.001806	0.001971	0.000516	0.022645	0.00483	0.002698	0.002277	0.003236	0.002027	0.003014	0.001114	0.002763	0.000125	0.002689	0.001818	0.002245	0.001928	0.001077	0.155841	0.003481	0.00248	0.003611	0.003047	0.003332	0.00319	0.000449	0.75075
742.47756	PG 16:1-18:3	Phosphatid	C40H71O1	0.054584	0.062202	0.062327	0.075195	0.082584	0.067379	0.011273	0.054787	0.076626	0.093632	0.183351	0.086586	0.098996	0.04938	0.200281	0.085094	0.079402	0.028704	0.036292	0.070365	0.059971	0.025764	0.572133	0.16843	0.067012	0.037885	0.080793	0.076776	0.086179	0.048945	0.064437	0.10448	0.075801	0.074632	0.068679	0.077606	0.015711	0.718877	0.118016	0.116978	0.155378	0.109496	0.121143	0.124202	0.017944	0.141545
744.49337	PG 16:1-18:2	Phosphatid	C40H73O1	0.00593	0.007037	0.008416	0.00966	0.009154	0.008039	0.001538	0.005694	0.007663	0.008725	0.015885	0.009379	0.009469	0.003848	0.462585	0.009621	0.009999	0.003849	0.006172	0.007963	0.007521	0.002552	0.707258	0.028453	0.011187	0.006982	0.011351	0.01126	0.013847	0.008373	0.009726	0.014619	0.011583	0.011186	0.012344	0.011892	0.001798	0.623493	0.021747	0.019411	0.024622	0.019499	0.017022	0.02046	0.002865	0.133269
744.49363	PG 16:0-18:3	Phosphatid	C40H73O1	0.022504	0.020004	0.026685	0.029701	0.028976	0.025574	0.004192	0.0247	0.029314	0.037102	0.080714	0.034955	0.041357	0.022531	0.162135	0.047164	0.041326	0.014028	0.021207	0.030325	0.03081	0.013712	0.437828	0.054258	0.025443	0.012694	0.030938	0.023486	0.029364	0.015411	0.018977	0.030486	0.0243	0.021448	0.02145	0.023332	0.004421	0.424615	0.031115	0.025619	0.034831	0.028356	0.03059	0.030102	0.00342	0.919299
746.50969	PG 16:0-18:2	Phosphatid	C40H75O1	0.004521	0.004136	0.006352	0.007125	0.007416	0.00591	0.001501	0.003311	0.005097	0.007089	0.012029	0.007155	0.006936	0.003261	0.54046	0.008751	0.008472	0.002952	0.005559	0.00495	0.006137	0.002459	0.864606	0.016242	0.008	0.003906	0.006582	0.005168	0.00798	0.004866	0.005346	0.008348	0.005465	0.006823	0.007219	0.00664	0.001259	0.567681	0.012026	0.010307	0.012813	0.010741	0.008525	0.010883	0.001654	0.242181
748.52477	PG 16:0-18:1	Phosphatid	C40H77O1	0.0058	0.005311	0.008177	0.010251	0.010706	0.008049	0.002474	0.004983	0.007042	0.010026	0.016722	0.011149	0.009984	0.004484	0.422635	0.010579	0.013042	0.005703	0.01117	0.008973	0.009893	0.002757	0.297935	0.024468	0.013116	0.006305	0.009378	0.007712	0.012196	0.007318	0.006764	0.012921	0.01228	0.011619	0.011707	0.011058	0.002457	0.750229	0.015484	0.013203	0.01979	0.015571	0.010666	0.014943	0.003373	0.467767

834.52738	Pil 18:2-16:0	Phosphatid	C43H79O1	0.001366	6.68E-05	0.001512	5.89E-05	0.001384	0.000878	0.000746	0.001983	0.001341	0.001417	4.29E-05	0.001041	0.001165	0.000714	0.550878	0.002437	0.001111	0.000774	0.000714	0.001502	0.001308	0.000705	0.376151	7.84E-05	0.001583	0.001087	0.004954	0.002292	0.001999	0.001838	0.00161	0.000125	0.002699	0.00007	6.84E-05	0.000914	0.001196	0.300901	0.000134	6.87E-05	0.003161	0.001994	9.26E-05	0.00109	0.001419	0.407008
749.50235	PC 16:3-18:3	Phosphoch	C42H72NC	0.001762	0.002987	0.001788	0.002381	0.001908	0.002165	0.000523	0.00463	0.005694	0.005316	0.003539	0.004758	0.004787	0.000819	0.000312	0.005514	0.004633	0.003382	0.002039	0.006616	0.004437	0.00179	0.026064	0.005774	0.003293	0.003902	0.006343	0.006421	0.005147	0.001452	0.0049	0.005795	0.006489	0.00304	0.002215	0.004488	0.001813	0.543458	0.007622	0.004447	0.007507	0.00392	0.007068	0.006113	0.001783	0.375001
755.54582	PC 16:0-18:3	Phosphoch	C42H78NC	0.072179	0.096718	0.071798	0.081434	0.054174	0.07526	0.015529	0.122741	0.150275	0.142084	0.123325	0.124688	0.132623	0.01273	0.000212	0.135518	0.118916	0.053261	0.042443	0.13276	0.09658	0.045087	0.34674	0.18254	0.097834	0.105886	0.140974	0.117356	0.128918	0.034099	0.106312	0.12331	0.119715	0.067638	0.053933	0.094181	0.031512	0.132886	0.222769	0.132917	0.188909	0.129506	0.19413	0.173646	0.040844	0.096944
757.56143	PC 16:0-18:2	Phosphoch	C42H80NC	0.107913	0.13105	0.115509	0.101928	0.07354	0.105988	0.021165	0.141683	0.169158	0.152283	0.151101	0.147666	0.152378	0.010244	0.002252	0.151468	0.152112	0.070364	0.058656	0.148857	0.116291	0.047466	0.669295	0.206587	0.097223	0.116385	0.128173	0.116481	0.13297	0.042624	0.139212	0.138603	0.15786	0.083158	0.071592	0.118085	0.03818	0.576824	0.244979	0.153252	0.221622	0.163104	0.198859	0.196363	0.038641	0.039083
759.57703	PC 16:0-18:1	Phosphoch	C42H82NC	0.049998	0.049637	0.044048	0.040503	0.027948	0.042427	0.009018	0.028129	0.039806	0.034916	0.03397	0.037928	0.03495	0.00447	0.135263	0.041166	0.039545	0.016515	0.014101	0.036915	0.029648	0.013206	0.111777	0.066174	0.039771	0.039984	0.045195	0.032219	0.044669	0.012881	0.048821	0.041423	0.072459	0.030131	0.025817	0.04373	0.018455	0.927989	0.071184	0.04704	0.059051	0.047389	0.054835	0.0559	0.009945	0.161348
767.55385	PC 17:1-18:3	Phosphoch	C43H78NC	0.022844	0.045965	0.023197	0.038349	0.029825	0.032036	0.010017	0.061227	0.054465	0.052844	0.031497	0.054248	0.050856	0.011301	0.023676	0.105048	0.056249	0.061481	0.054531	0.107962	0.077054	0.027026	0.008168	0.090894	0.047766	0.092867	0.111959	0.138367	0.096371	0.033209	0.071411	0.091087	0.218774	0.052469	0.051079	0.096964	0.070018	0.98676	0.091384	0.058474	0.126655	0.062057	0.087791	0.085272	0.027447	0.580444
769.56659	PC 17:1-18:2	Phosphoch	C43H80NC	0.004289	0.005412	0.003163	5.89E-05	0.004076	0.0034	0.002032	0.008119	0.008994	0.00839	0.0054	7.84E-05	0.006196	0.003688	0.175819	0.000116	0.007455	0.006673	0.004862	0.011518	0.006125	0.004149	0.223719	0.008737	0.004407	0.006649	0.007617	0.005644	0.006611	0.001683	0.006653	0.006358	0.012586	0.004305	0.003581	0.006697	0.003544	0.962221	0.010265	6.87E-05	0.010248	0.0066	0.011376	0.007711	0.004638	0.631315
777.53029	PC 18:3-18:3	Phosphoch	C44H76NC	0.05605	0.08108	0.061785	0.069154	0.047123	0.063039	0.012909	0.127563	0.146103	0.148803	0.105673	0.126186	0.130866	0.017474	0.000115	0.113748	0.09641	0.064398	0.048312	0.131345	0.090843	0.034294	0.128193	0.154256	0.084282	0.093448	0.143529	0.13882	0.122867	0.031706	0.104264	0.11812	0.121178	0.061013	0.04675	0.090265	0.034193	0.156595	0.190795	0.115951	0.186856	0.114308	0.219931	0.165568	0.047787	0.134485
779.54366	PC 18:0-18:5	Phosphoch	C44H78NC	0.017029	0.018883	0.017733	0.014883	0.010706	0.015847	0.003222	0.020969	0.027526	0.02495	0.024777	0.022025	0.024049	0.002599	0.002195	0.021449	0.024023	0.009411	0.007123	0.021206	0.016642	0.007767	0.83778	0.030848	0.013651	0.015749	0.01819	0.016137	0.018915	0.006863	0.020877	0.020302	0.021078	0.011874	0.010548	0.016936	0.005255	0.622388	0.041931	0.024957	0.032296	0.026072	0.030864	0.031224	0.00674	0.021107
779.54584	PC 18:2-18:3	Phosphoch	C44H78NC	0.074435	0.110862	0.080771	0.091399	0.063484	0.08419	0.018021	0.151863	0.171104	0.16654	0.147897	0.152525	0.157986	0.010178	4.47E-05	0.132673	0.117224	0.060754	0.05305	0.146404	0.102021	0.042549	0.413334	0.199658	0.093548	0.107445	0.144239	0.147506	0.138479	0.04135	0.120835	0.133339	0.145255	0.068449	0.055334	0.104643	0.040238	0.22612	0.240229	0.133025	0.213604	0.133815	0.223989	0.188932	0.051557	0.12621
781.56135	PC 18:2-18:2	Phosphoch	C44H80NC	0.035651	0.037637	0.040589	0.035652	0.020969	0.0341	0.007613	0.050492	0.057852	0.054283	0.038589	0.049569	0.050157	0.007255	0.009166	0.039071	0.041456	0.022784	0.017151	0.043025	0.032698	0.011874	0.829654	0.051435	0.027446	0.038972	0.044377	0.041858	0.040818	0.008785	0.051829	0.042827	0.047311	0.027243	0.02613	0.039068	0.011749	0.796414	0.089049	0.049199	0.069096	0.051406	0.062609	0.064272	0.016067	0.021023
783.57747	PC 18:1-18:2	Phosphoch	C44H82NC	0.024466	0.027799	0.02138	0.023813	0.015572	0.022606	0.004551	0.024603	0.027961	0.027163	0.022604	0.023567	0.02518	0.002304	0.291971	0.024238	0.022639	0.010599	0.009221	0.02217	0.017773	0.007236	0.241753	0.034218	0.017873	0.021109	0.025269	0.020146	0.023723	0.006449	0.025038	0.025033	0.039704	0.015171	0.013268	0.023643	0.010503	0.988759	0.042546	0.026132	0.04046	0.027924	0.034353	0.034283	0.007303	0.041617
785.59286	PC 18:1-18:1	Phosphoch	C44H84NC	0.026385	0.032177	0.013331	0.027004	0.018047	0.023389	0.007566	0.030849	0.039047	0.033534	0.032134	0.030514	0.033216	0.003471	0.02973	0.035905	0.036172	0.014723	0.012798	0.03467	0.026853	0.011985	0.599577	0.038947	0.01756	0.024589	0.029606	0.022948	0.02673	0.00807	0.029598	0.030527	0.040749	0.020656	0.017807	0.027867	0.009072	0.839351	0.04782	0.033669	0.056296	0.036289	0.041269	0.043068	0.009153	0.01723
687.48343	PE 16:1-16:1	Phosphoetl	C37H70NC	0.002748	0.001132	0.027575	0.016338	0.029674	0.015494	0.013383	0.001079	9.27E-05	0.018018	4.29E-05	0.03333	0.010513	0.014868	0.592897	0.000116	8.25E-05	0.009323	0.023726	0.002222	0.007094	0.010039	0.294147	7.84E-05	0.012104	0.00585	0.003139	0.003184	0.004871	0.00453	0.000117	0.004255	0.003671	0.00007	0.018225	0.005268	0.0075	0.921877	0.000134	6.87E-05	0.000117	9.28E-05	9.26E-05	0.000101	2.51E-05	0.046355
699.48312	PE 15:0-18:3	Phosphoetl	C38H70NC	0.002393	0.004175	0.002644	0.003258	0.003285	0.003151	0.000691	0.00234	0.004	0.003658	0.003848	0.001998	0.003169	0.000929	0.973656	0.00449	0.002893	0.002729	0.003126	0.005691	0.003786	0.001272	0.355808	0.010354	0.006859	0.004871	0.013035	0.008619	0.008748	0.003145	0.004518	0.008947	0.01005	0.004536	0.008005	0.007211	0.002555	0.421151	0.005572	0.011104	0.015113	0.014425	0.003832	0.010009	0.005114	0.651064
711.48317	PE 14:0-20:4	Phosphoetl	C39H70NC	0.00377	0.001582	0.015425	0.013361	0.021624	0.011153	0.00835	0.001642	0.001807	0.012724	0.001901	0.021312	0.007877	0.00888	0.564578	0.000116	0.000785	0.008652	0.016205	0.003115	0.005775	0.006728	0.294643	0.001041	0.0113	0.004507	0.003693	0.004332	0.004974	0.0038	0.003533	0.00482	0.00429	0.00007	0.014339	0.005411	0.005324	0.885141	0.004788	0.001436	0.000117	0.001794	9.26E-05	0.001646	0.001916	0.118416
713.49895	PE 16:0-18:3	Phosphoetl	C39H72NC	0.028906	0.037301	0.040642	0.038721	0.040596	0.037233	0.00486	0.025893	0.035761	0.062654	0.048211	0.048137	0.044131	0.013951	0.326955	0.038114	0.04235	0.026593	0.027112	0.03762	0.034358	0.007096	0.476147	0.049393	0.038895	0.040559	0.037424	0.031932	0.03964	0.006341	0.031588	0.033551	0.046057	0.026495	0.029271	0.033392	0.007554	0.194346	0.03803	0.032761	0.032969	0.035595	0.034547	0.03478	0.002158	0.143391
715.51514	PE 16:0-18:2	Phosphoetl	C39H74NC	0.013256	0.017053	0.017421	0.016252	0.020495	0.016895	0.002594	0.011674	0.015147	0.024549	0.020722	0.022722	0.018963	0.005388	0.461658	0.019136	0.017574	0.009011	0.012367	0.016123	0.014842	0.004113	0.372729	0.02071	0.017513	0.014885	0.014153	0.013762	0.016205	0.002912	0.012141	0.01079	0.016959	0.010846	0.010712	0.01229	0.002676	0.05779	0.017878	0.01424	0.015112	0.01509	0.013902	0.015244	0.001564	0.534238
739.51479	PE 18:2-18:2	Phosphoetl	C41H74NC	0.016676	0.022005	0.02209	0.017102	0.017591	0.019093	0.002717	0.016654	0.018125	0.032334	0.022216	0.019747	0.021815	0.006232	0.396704	0.016936	0.020094	0.009347	0.008613	0.018082	0.014614	0.005273	0.129837	0.020463	0.01585	0.013247	0.017248	0.012361	0.015834	0.003247	0.019358	0.01416	0.024769	0.011149	0.007843	0.015456	0.006711	0.912566	0.023059	0.013702	0.007519	0.017898	0.011957	0.014827	0.005918	0.747351
741.53284	PE 18:1-18:2	Phosphoetl	C41H76NC	7.85E-05	0.005722	0.004041	0.005515	0.005103	0.004092	0.002335	9.79E-05	0.00318	0.005289	0.004003	0.004175	0.003349	0.001967	0.60119	0.000116	8.25E-05	0.002807	0.001879	0.003975	0.001772	0.001698	0.110106	7.84E-05	0.004519	0.003894	0.005379	0.004893	0.003753	0.002125	0.000117	0.000125	0.007499	0.002953	0.003053	0.002749	0.003021	0.560346	0.000134	0.003699	0.000117	9.28E-05	9.26E-05	0.000827	0.001606	0.039529
813.6261	PE 18:2-23:0	Phosphoetl	C46H88NC	0.002762	0.004015	0.002838	0.003242	0.00224	0.003019	0.000661	0.008485	0.00463	0.004042	0.002238	0.005014	0.004882	0.002278	0.117252	0.004281	0.004645	0.003673	0.002578	0.004213	0.003878	0.000805	0.102574	0.003495	0.002807	0.006244	0.004805	0.004755	0.004421	0.001327	0.004734	0.004166	0.004861	0.002142	0.003	0.003781	0.001175	0.442352	0.00473	0.003142	0.00487	0.003963	0.003935	0.004128	0.000698	0.673385
827.63948	PE 18:2-24:0	Phosphoetl	C47H90NC	0.001162	0.001991	0.003091	0.003487	0.004315	0.002809	0.001244	0.00119	0.00181	0.002947	0.002714	0.004256	0.002583	0.001171	0.775024	0.001803	0.001786	0.001223	0.002371	0.001549	0.001746	0.000421	0.107907	0.001989	0.003378	0.002532	0.002014	0.001943	0.002371	0.000612	0.001657	0.001788	0.002973	0.0013	0.003013	0.002146	0.000793	0.629319	0.00176	0.001581	0.001788	0.001981	0.001865	0.001795	0.000147	0.074876
245.23581	Sphinganine d14:0	Sphingosin	C14H31NC	0.222503	0.106944	0.140643	0.032951	0.029416	0.106491	0.080583	0.256799	0.417307	0.153101	4.29E-05	7.84E-05	0.165466	0.17793	0.518627	0.811091	0.04578	0.563695	0.004181	0.432502	0.37145	0.344571	0.132614	0.022999	0.229228	0.309369	1E-04	8.81E-05	0.112357	0.146341	0.028044	0.261359	0.274176	0.54127	0.029507	0.226871	0.212579	0.350167	0.024339	0.194935	0.000117	9.28E-05	9.26E-05	0.043915	0.085073	0.392353
259.25114	Sphinganine d15:0	Sphingosin	C15H33NC	0.003006	0.0014	0.003964	0.000476	0.000807	0.001931	0.001496	0.003844	0.00486	0.003583	4.29E-05	7.84E-05	0.002482	0.002261	0.661633	0.019146	0.001292	0.01057	4.85E-05	0.005975	0.007406	0.007769	0.160292	0.00098	0.003778	0.003453	1E-04	8.81E-05	0.00168	0.001807	0.000117	0.001531	0.003635	0.008954	6.84E-05	0.002861	0.003702	0.539225	0.000134	0.003903	0.000117	9.28E-05	9.26E-05	0.000868	0.001697	0.484843
261.23031	Sphingosine t14:0	Sphingosin	C14H31NC	0.074144	0.036658	0.027493	0.011294	0.01072	0.032062	0.025979	0.036254	0.059846	0.088147	4.29E-05	7.84E-05	0.036874	0.0383	0.821989	0.277799	0.005728	0.182378	0.00086	0.066351	0.106623	0.120444	0.213009	0.001981	0.033586	0.053789	1E-04	8.81E-05	0.017909	0.024605	0.006132	0.056351	0.068356	0.114679	0.017203	0.052544	0.043383	0.159062	0.006592	0.11147	0.000117	9.28E-05	9.26E-05	0.023673	0.049161	0.820517
271.25117	Sphinganine d16:1	Sphingosin	C16H33NC	0.008884	0.009052	0.00253	5.89E-05	0.002552	0.004615	0.004101	0.018449	0.021838	0.019991	4.29E-05	7.84E-05	0.01208	0.011038	0.194069	0.030754	8.25E-05	0.022479	4.85E-05	0.020566	0.014786	0.013973	0.156973	7.84E-05	0.016757	0.01068	1E-04	8.81E-05	0.005541	0.007768	0.000117	0.000125	0.019385	0.018947	6.84E-05	0.007728	0.010442	0.716777	0.000134	0.014144	0.000117	9.28E-05	9.26E-05	0.002916	0.006276	0.57296
273.26687	Sphinganine d16:0	Sphingosin	C16H35NC	0.118865	0.082232	0.252674	0.015431	0.082281	0.110297	0.087916	0.250276	0.150103	0.459005	0.001133	7.84E-05	0.172119	0.192179	0.531379	1.903019	0.159512	0.724001	0.015372	0.372921	0.634965	0.757381	0.162443	0.119445	0.161505	0.107173	0.005289	0.001984	0.079079	0.071766	0.14394	0.034874	0.139631	0.618073	0.027771	0.192858	0.244052	0.346538	0.01203	0.217932	0.005956	0.002399	0.004445	0.048552	0.094754	0.581571
289.26149	Sphingosine t16:0	Sphingosin	C16H35NC	0.011202	0.003232	0.015429	0.001142	0.008166	0.007834	0.005812	0.010836	0.009406	0.092456	4.29E-05	7.84E-05	0.022564	0.039397	0.432175	0.14574	0.008166	0.06219	0.001353	0.010758	0.045642	0.060984	0.20492	0.004247	0.009348	0.00817	1E-04	8.81E-05	0.004391	0.004353	0.006197	0.004688	0.012968	0.043993	0.004716	0.014512	0.016831	0.229183	0.005771	0.04989	0.000117	9.28E-05	9.26E-05	0.011193	0.021771	0.512667
299.28142	Sphingosine d18:1	Sphingosin	C18H37NC	0.002147	6.68E-05	0.000206	5.89E-05	3.95E-05	0.000504	0.000921	9.79E-05	9.27E-05	9.08E-05	4.29E-05	7.84E-05	8.05E-05	2.22E-05	0.334415	0.000116	8.25E-05	9.09E-05	4.85E-05	8.68E-05	0.000085	2.43E-05	0.339278	0.002451	0.002146	0.003142	0.002984	8.81E-05	0.002162	0.001227	0.000117	0.000125	0.000108	0.002712	6.84E-05	0.000626	0.001166	0.076961	0.000134	6.87E-05	0.000117	9.28E-05	9.26E-05	0.000101	2.51E-05	0.005579
301.298	Sphinganine d18:0	Sphingosin	C18H39NC	0.001494	0.002737	0.009171	5.89E-05	0.003765	0.003445	0.003488	0.003304	9.27E-05	0.021575	4.29E-05	7.84E-05	0.005019	0.00936	0.733788	0.109497	0.012828	0.017924	4.85E-05	0.021631	0.032386	0.043872	0.179657	0.005112	0.001277	0.000102	1E-04	8.81E-05	0.001336	0.002172	0.012188	0.000125	0.000108	0.008978	6.84E-05	0.004294	0.005853	0.32037	0.000134	0.003238	0.000117	9.28E-05	9.26E-05	0.000735	0.001399	0.617019
317.2932	Sphingosine t18:0	Sphingosin	C18H39NC	0.003363	6.68E-05	0.001546	5.89E-05	0.001282	0.001263	0.001357	0.001389	9.27E-05	0.010663	4.29E-05	7.84E-05	0.002453	0.004625	0.596035	0.025028	0.002499	0.004886	4.85E-05	8.68E-05	0.00651	0.010543	0.301879	0.002256	0.00207	0.000102	1E-04	8.81E-05	0.000923	0.001134	0.003019	0.000125	0.000108	0.00007	6.84E-05	0.000678	0.001309	0.759671	0.003618	6.87E-05	0.000117	9.28E-05	9.26E-05	0.000798	0.001577	0.888828
788.47478	SQDG 16:0-16:3	Sulfoquino	C41H72O1	7.85E-05	0.001985	0.000206	0.000862	0.000943	0.000815	0.000758	0.002004	0.001852	0.001372	0.001927	0.001544	0.00174	0.00027	0.033203	0.002092	0.001236	0.0011	4.85E-05	0.001666	0.001228	0.000766	0.415978	0.003031	0.001052	0.001511	0.002693	0.001628	0.001983	0.000839	0.00138	0.003259	0.002843	0.000913	0.000576	0.001794	0.001191	0.779268	0.001895	0.002919	0.004876	0.002802	0.002112	0.002921	0.001177	0.184936
792.50492	SQDG 16:0-16:1	Sulfoquino	C41H76O1	7.85E-05	0.002248	0.000206	5.89E-05	3.95E-05	0.000526	0.000965	9.79E-05	9.27E-05	9.08E-05	0.002075	7.84E-05	0.000487	0.000888	0.948067	0.000116	8.25E-05	9.09E-05	4.85E-05	8.68E-05	0.000085	2.43E-05	0.336481	0.004913	5.37E-05	0.000102	0.002757	8.81E-05	0.001583	0.002193	0.000117	0.005294	0.000108	0.00007	6.84E-05	0.001132	0.002327	0.760437	0.000134	0.003577	0.004731	0.004911	9.26E-05	0.002689	0.002406	0.469158
794.52025	SQDG 16:0-16:0	Sulfoquino	C41H78O1	0.004919	0.010574	0.005925	0.005021	0.00492	0.006272	0.002442	0.005834	0.004408	0.003475	0.008699	0.004469	0.005377	0.002038	0.546845	0.006613	0.004434	0.001985	0.001779	0.004888	0.00394	0.002048	0.140426	0.015274	0.005714	0.006666	0.008624	0.006097	0.008475	0.003962	0.006654	0.015861	0.009153	0.005462	0.003745	0.008175	0.004726	0.916088	0.009072	0.019458	0.02368	0.019798	0.008706	0.016143	0.006828	0.061626
816.50489	SQDG 16:0-18:3	Sulfoquino	C43H76O1	0.051914	0.128201	0.058806	0.059886	0.058505	0.071462	0.031873	0.103346	0.0881	0.077182	0.115352	0.071201	0.091036	0.018281	0.267717	0.1296	0.080362	0.053808	0.038858	0.10204	0.080934	0.036471	0.673493	0.21813	0.064556	0.085394	0.133999	0.093615	0.119139	0.060806	0.091546	0.226145	0.161651	0.059975	0.049176	0.117699	0.07484	0.974176	0.079226	0.168612	0.248098	0.176568	0.110169	0.156534	0.065302	0.376116
818.52068	SQDG 16:0-18:2	Sulfoquino	C43H78O1	0.007677	0.018364	0.008376	0.008953	0.009266	0.010527	0.004423	0.015437	0.012057	0.00956	0.016989	0.009985	0.012806	0.003297	0.382717	0.020434	0.013226	0.009766	0.006028	0.016096	0.01311	0.005566	0.440089	0.041768	0.014258	0.021816	0.028514	0.020671	0.025405	0.010452	0.014925	0.03822	0.02775	0.011304	0.010395	0.020519	0.012087	0.513401	0.017784	0.035368	0.050922	0.036916	0.017588	0.031715	0.014168	0.446046
820.53617	SQDG 16:0-18:1	Sulfoquino	C43H80O1	0.003928	0.00873	0.004362	0.004613	0.00387	0.005101	0.002052	0.006861	0.004717	0.003519	0.008449	0.004217	0.005553	0.002045	0.736126	0.008064	0.006215	0.003286	0.002867	0.006223	0.005331	0.002196	0.868128	0.020176	0.005731	0.008342	0.010959	0.006194	0.01028	0.005907	0.007245	0.017029	0.016301	0.005945	0.004608	0.010226	0.005958	0.988701	0.008661	0.019016	0.026903	0.020678	0.009144	0.01688	0.007856	0.171604
838.48924	SQDG 18:3-18:3	Sulfoquino	C45H74O1	0.01408	0.028827	0.016551	0.015222	0.014812	0.017898	0.006175	0.034739	0.0263	0.025157	0.025715	0.0243	0.027242	0.004255	0.0237	0.048043	0.024952	0.02135	0.014643	0.039889	0.029775	0.013777	0.116617	0.05733	0.019628	0.029196	0.05336	0.04121	0.040145	0.015905	0.035556	0.069616	0.056461	0.020686	0.01648	0.03976	0.022879	0.976103	0.025528	0.041597	0.062945	0.04709	0.044403	0.044313	0.013371	0.665691
840.50472	SQDG 18:2-18:3	Sulfoquino	C45H76O1	7.85E-05	0.010741	0.000206	5.89E-05	3.95E-05	0.002225	0.004761	9.79E-05	9.27E-05	9.08E-05	0.008569	7.84E-05	0.001786	0.003792	0.875858	0.015268	8.25E-05	9.09E-05	4.85E-05	8.68E-05	0.003115	0.006794	0.81635	0.021832	0.008434	0.017533	0.019785	0.013405	0.016198	0.005349	0.000117	0.026798	0.01835	0.00007	0.004214	0.00991	0.012058	0.317608	0.000134	0.015736	0.022814	0.01627	0.014835	0.013958	0.008349	0.627055
842.52053	SQDG 18:1-18:3	Sulfoquino	C45H78O1	0.00198	0.004579	0.002507	0.002559	0.002721	0.002869	0.000995	0.003739	0.0031	0.002294	0.003741	0.002757	0.003126	0.000629	0.638741	0.005574	0.00358	0.002155	0.001544	0.004338	0.003438	0.00163	0.524174	0.008157	0.002618	0.003756	0.004528	0.003255	0.004463	0.00218	0.003386	0.006907	0.00535	0.002053	0.001718	0.003883	0.002212	0.687315	0.002823	0.005636	0.008842	0.00571	0.00405	0.005412	0.002262	0.518259
772.65725	TG 14:1-16:1-16:1	Triacylglyce	C49H88O6	0.01429	0.0091	0.005687	0.00794	0.004454	0.008294	0.003817	0.013573	0.018643	0.014137	0.008223	0.011077	0.013131	0.00387	0.081813	0.013607	0.011654	0.0146	0.011676	0.019065	0.01412	0.003042	0.028393	0.029045	0.012082	0.01885	0.02193	0.014375	0.019256	0.006679	0.018571	0.023973	0.019831	0.02126	0.011473	0.019022	0.004674	0.950223	0.01514	0.00512	0.024406	0.017454	0.014665	0.015357	0.006921	0.391091
774.67294	TG 14:1-16:0-16:1	Triacylglyce	C49H90O6	0.061848	0.030371	0.022551	0.033155	0.017182	0.033021	0.01731	0.051692	0.075357	0.064572	0.031031	0.045934	0.053717	0.017069	0.09345	0.051481	0.047107	0.062419	0.04537	0.079657	0.057207	0.014196	0.042128	0.11466	0.051106	0.074191	0.095956	0.058645	0.078912	0.026338	0.080754	0.10107	0.082341	0.09392	0.047905	0.081198	0.020412	0.88187	0.056262	0.022018	0.106613	0.074044	0.063922	0.064572	0.030567	0.449717
776.68856	TG 14:0-16:0-16:1	Triacylglyce	C49H92O6	0.113205	0.058737	0.039813	0.056762	0.030496	0.059803	0.032096	0.106214	0.142197	0.113451	0.060769	0.085309	0.101588	0.030588	0.068152	0.085022	0.078386	0.101109	0.074136	0.133518	0.094434	0.024136	0.089955	0.221607	0.089882	0.138949	0.157945	0.094689	0.140615	0.053726	0.14277	0.172421	0.125313	0.17624	0.08171	0.139691	0.038681	0.975868	0.104286	0.037276	0.183858	0.132331	0.115551	0.11466	0.052909	0.463616
778.70401	TG 14:0-16:0-16:0	Triacylglyce	C49H94O6	0.09571	0.068877	0.051921	0.055371	0.031129	0.060602	0.023835	0.102441	0.103497	0.105186	0.047212	0.079801	0.087627	0.024871	0.117468	0.075819	0.071314	0.073786	0.056926	0.09857	0.075283	0.014979	0.277137	0.162111	0.066591	0.107531	0.121575	0.075259	0.106613	0.038372	0.106254	0.126619	0.088953	0.125136	0.06543	0.102479	0.025816	0.84653	0.090851	0.037265	0.151239	0.11198	0.10291	0.098849	0.041201	0.765677
786.67312	TG 15:1-16:1-16:1	Triacylglyce	C50H90O6	0.018124	0.012375	0.004948	0.014819	0.007208	0.011495	0.005409	0.019978	0.02496	0.020811	0.010877	0.015643	0.018454	0.005374	0.075566	0.017903	0.011687	0.027893	0.017539	0.026437	0.020292	0.006762	0.052745	0.038231	0.013401	0.030376	0.034446	0.016546	0.0266	0.011027	0.035211	0.032523	0.031095	0.034458	0.015418	0.029741	0.008168	0.622609	0.018375	0.007131	0.040562	0.024704	0.015753	0.021305	0.012477	0.497256
788.68856	TG 15:1-16:0-16:1	Triacylglyce	C50H92O6	0.05471	0.029645	0.020059	0.030588	0.015468	0.030094	0.015174	0.055545	0.067506	0.058778	0.028018	0.042365	0.050442	0.01545	0.06882	0.045079	0.041215	0.05723	0.040387	0.073317	0.051446	0.013958	0.049249	0.106682	0.045461	0.074326	0.084902	0.052691	0.072812	0.024742	0.071132	0.090068	0.074533	0.083293	0.041549	0.072115	0.01863	0.961075	0.051969	0.019788	0.093587	0.066274	0.056463	0.057616	0.026613	0.377089
790.70415	TG 15:0-16:0-16:1	Triacylglyce	C50H94O6	0.102994	0.060409	0.034564	0.057094	0.028521	0.056716	0.029331	0.098564	0.117204	0.101441	0.04925	0.072118	0.087716	0.026919	0.119838	0.074025	0.071474	0.091095	0.066429	0.112627	0.08313	0.018916	0.12905	0.191994	0.07542	0.128726	0.14767	0.084554	0.125673	0.047718	0.118405	0.158004	0.116474	0.147876	0.069856	0.122123	0.034381	0.895974	0.087406	0.031576	0.157932	0.111572	0.100948	0.097887	0.045577	0.373968
792.71956	TG 15:0-16:0-16:0	Triacylglyce	C50H96O6	0.084464	0.058677	0.040046	0.045771	0.026139	0.051019	0.022047	0.088443	0.083841	0.082347	0.038396	0.063967	0.071399	0.020673	0.170045	0.066065	0.059351	0.062287	0.048993	0.082171	0.063774	0.012083	0.289474	0.151538	0.057143	0.097091	0.109457	0.064027	0.095851	0.038075	0.088073	0.118033	0.079964	0.103455	0.054845	0.088874	0.023984	0.737761	0.077892	0.03121	0.123521	0.091913	0.091495	0.083206	0.033547	0.59264

796.65676	TG 16:1-16:1-16:3	Triacylglyce	C51H88O6	0.001257	6.68E-05	0.000206	5.89E-05	3.95E-05	**0.000326**	**0.000525**	9.79E-05	9.27E-05	9.08E-05	0.000692	7.84E-05	**0.00021**	**0.000269**	**0.67329**	0.000116	8.25E-05	0.005282	0.000508	0.001195	**0.001437**	**0.002196**	**0.303139**	0.001762	5.37E-05	0.000102	0.001122	8.81E-05	**0.000626**	**0.000779**	0.000117	0.001385	0.000108	0.00007	0.000546	**0.000445**	**0.00056**	**0.685316**	0.000134	6.87E-05	0.000117	9.28E-05	0.000925	**0.000267**	**0.000369**	**0.380093**
798.67278	TG 14:1-16:1-18:2	Triacylglyce	C51H90O6	0.003571	0.002187	0.001569	0.002446	0.001253	**0.002205**	**0.000899**	0.003571	0.005231	0.004507	0.002341	0.003231	**0.003776**	**0.001124**	**0.040516**	0.003958	0.003098	0.004949	0.003188	0.005886	**0.004216**	**0.001194**	**0.016851**	0.008205	0.003178	0.004871	0.006755	0.00422	**0.005446**	**0.002019**	0.00481	0.006712	0.005444	0.005682	0.00289	**0.005107**	**0.001416**	**0.766739**	0.00414	0.001615	0.007009	0.005026	0.003702	**0.004298**	**0.001966**	**0.389042**
800.68845	TG 14:1-16:1-18:1	Triacylglyce	C51H92O6	0.031014	0.015158	0.014153	0.015759	0.008645	**0.016946**	**0.008355**	0.024853	0.037041	0.030942	0.015705	0.023519	**0.026412**	**0.008046**	**0.105457**	0.02591	0.021045	0.030396	0.020967	0.035629	**0.026789**	**0.006301**	**0.06858**	0.052995	0.02459	0.038711	0.043728	0.027713	**0.037547**	**0.011649**	0.03771	0.046764	0.038913	0.045142	0.023526	**0.038411**	**0.009184**	**0.899622**	0.028257	0.01035	0.047786	0.03442	0.028679	**0.029898**	**0.013479**	**0.365134**
802.70428	TG 16:0-16:1-16:1	Triacylglyce	C51H94O6	0.143241	0.073409	0.048917	0.079863	0.042756	**0.077637**	**0.039901**	0.137037	0.18983	0.140027	0.080085	0.110696	**0.131535**	**0.040565**	**0.067022**	0.110162	0.105041	0.142536	0.098617	0.174852	**0.126242**	**0.032014**	**0.066356**	0.286107	0.116688	0.183278	0.196121	0.125759	**0.181591**	**0.067947**	0.191921	0.240532	0.177161	0.229706	0.11125	**0.190114**	**0.051235**	**0.828403**	0.135546	0.046263	0.236572	0.171328	0.151362	**0.148214**	**0.068749**	**0.462247**
804.71983	TG 16:0-16:0-16:1	Triacylglyce	C51H96O6	0.188291	0.112407	0.060787	0.102851	0.054408	**0.103749**	**0.053627**	0.197175	0.212311	0.194584	0.094922	0.143327	**0.168464**	**0.048656**	**0.080711**	0.145082	0.128416	0.164524	0.124502	0.212427	**0.15499**	**0.03579**	**0.113441**	0.365227	0.138503	0.229747	0.253409	0.142103	**0.225798**	**0.093332**	0.227174	0.304838	0.206979	0.281059	0.132218	**0.230454**	**0.06765**	**0.930257**	0.178316	0.060343	0.290071	0.213389	0.18973	**0.18637**	**0.082829**	**0.499921**
806.73513	TG 16:0-16:0-16:0	Triacylglyce	C51H98O6	0.039218	0.026088	0.040867	0.025673	0.012486	**0.028866**	**0.011591**	0.03391	0.039126	0.044509	0.020337	0.031714	**0.033919**	**0.009063**	**0.464618**	0.037045	0.028202	0.028698	0.019074	0.034988	**0.029601**	**0.007036**	**0.906516**	0.043559	0.019567	0.032873	0.038347	0.027096	**0.032288**	**0.009393**	0.040632	0.039716	0.034282	0.038328	0.026193	**0.03583**	**0.005909**	**0.495722**	0.038666	0.014796	0.051494	0.039831	0.034861	**0.03593**	**0.013349**	**0.631319**
806.73518	TG 14:0-16:0-18:0	Triacylglyce	C51H98O6	0.136307	0.103323	0.072714	0.080989	0.051687	**0.089004**	**0.032267**	0.157493	0.133458	0.142676	0.067085	0.112883	**0.122719**	**0.035052**	**0.152224**	0.112393	0.097696	0.101885	0.075745	0.119898	**0.101524**	**0.016845**	**0.46395**	0.253446	0.088267	0.159508	0.167964	0.108471	**0.155531**	**0.06422**	0.1497	0.205668	0.132436	0.161172	0.094861	**0.148767**	**0.040512**	**0.847086**	0.139468	0.048167	0.205826	0.143283	0.14973	**0.137295**	**0.056647**	**0.646673**
814.7041	TG 15:0-16:1-18:2	Triacylglyce	C52H94O6	0.025868	0.013348	0.010229	0.01509	0.007892	**0.014485**	**0.006943**	0.025544	0.032334	0.028822	0.013519	0.02111	**0.024266**	**0.007296**	**0.06168**	0.020617	0.019853	0.02591	0.019347	0.033796	**0.023905**	**0.006121**	**0.052433**	0.049685	0.022048	0.034101	0.040185	0.024004	**0.034004**	**0.011479**	0.035374	0.04348	0.034542	0.038556	0.020693	**0.034529**	**0.008492**	**0.936522**	0.025057	0.009456	0.044244	0.03034	0.026337	**0.027087**	**0.012455**	**0.38782**
816.71963	TG 15:0-16:1-18:1	Triacylglyce	C52H96O6	0.085738	0.045635	0.026624	0.046934	0.025761	**0.046138**	**0.024316**	0.080826	0.093325	0.087388	0.043587	0.06063	**0.073151**	**0.020612**	**0.094713**	0.063113	0.063984	0.079973	0.05609	0.100092	**0.07265**	**0.017656**	**0.083981**	0.169425	0.066507	0.109469	0.123501	0.075958	**0.108972**	**0.041107**	0.106208	0.141547	0.10689	0.124479	0.063795	**0.108584**	**0.028956**	**0.986656**	0.079843	0.027433	0.136703	0.096205	0.082132	**0.084463**	**0.039198**	**0.362883**
818.73536	TG 16:0-16:1-17:0	Triacylglyce	C52H98O6	0.10732	0.060236	0.033716	0.053332	0.032456	**0.057412**	**0.030413**	0.108867	0.113204	0.103226	0.049459	0.07394	**0.089739**	**0.027251**	**0.114658**	0.075449	0.074063	0.08887	0.069221	0.117972	**0.085115**	**0.01976**	**0.126022**	0.212673	0.079292	0.127211	0.139357	0.081422	**0.127991**	**0.054409**	0.114832	0.17127	0.1234	0.150237	0.071153	**0.126178**	**0.038001**	**0.952797**	0.091158	0.032776	0.159154	0.111819	0.108764	**0.100734**	**0.04558**	**0.415494**
820.75089	TG 16:0-16:0-17:0	Triacylglyce	C52H100O	0.062336	0.044078	0.031104	0.035812	0.02023	**0.038712**	**0.01577**	0.063907	0.055962	0.056737	0.026551	0.049178	**0.050467**	**0.014351**	**0.25266**	0.04586	0.040806	0.040754	0.03591	0.052486	**0.043163**	**0.006288**	**0.573856**	0.11105	0.039494	0.069472	0.073131	0.044205	**0.06747**	**0.028545**	0.062217	0.088128	0.054685	0.06815	0.039998	**0.062636**	**0.017721**	**0.755887**	0.05472	0.021966	0.079939	0.062567	0.058759	**0.05559**	**0.021112**	**0.475747**
822.67287	TG 16:0-16:3-18:3	Triacylglyce	C53H90O6	7.85E-05	0.002412	0.001756	0.003742	0.00189	**0.001976**	**0.00132**	0.005191	0.004166	0.005468	0.003399	0.002917	**0.004228**	**0.001104**	**0.01907**	0.009966	0.008389	0.009305	0.003726	0.00923	**0.008123**	**0.002521**	**0.001303**	0.005959	0.004259	0.008634	0.011672	0.005843	**0.007273**	**0.002918**	0.00706	0.006471	0.006823	0.003603	0.003727	**0.005537**	**0.001722**	**0.284861**	0.00648	0.005376	0.01021	0.007277	0.007865	**0.007442**	**0.001807**	**0.915471**
826.70399	TG 16:0-16:1-18:3	Triacylglyce	C53H94O6	0.011499	0.007234	0.005301	0.007375	0.004365	**0.007155**	**0.002745**	0.011294	0.015667	0.014519	0.007761	0.011108	**0.01207**	**0.003124**	**0.029584**	0.01556	0.012456	0.01434	0.00959	0.017086	**0.013806**	**0.002903**	**0.005851**	0.021981	0.01064	0.017413	0.023722	0.013114	**0.017374**	**0.005591**	0.015477	0.021645	0.018221	0.018484	0.010645	**0.016894**	**0.004121**	**0.881049**	0.014392	0.006895	0.025612	0.015844	0.015438	**0.015636**	**0.006662**	**0.666906**
828.71972	TG 16:0-16:1-18:2	Triacylglyce	C53H96O6	0.071092	0.036568	0.025578	0.040692	0.022908	**0.039368**	**0.019216**	0.068546	0.09366	0.071384	0.043869	0.057332	**0.066958**	**0.018445**	**0.049211**	0.06948	0.063963	0.076577	0.04861	0.095193	**0.070765**	**0.017097**	**0.025866**	0.138758	0.062114	0.103363	0.111692	0.063815	**0.095948**	**0.032836**	0.094137	0.120063	0.088935	0.111727	0.056505	**0.094274**	**0.024621**	**0.929542**	0.075539	0.029067	0.129699	0.088556	0.077631	**0.080098**	**0.035926**	**0.487262**
830.73549	TG 16:0-16:1-18:1	Triacylglyce	C53H98O6	0.149704	0.085812	0.048865	0.086207	0.049482	**0.084014**	**0.041083**	0.171986	0.181835	0.170015	0.084786	0.129247	**0.147574**	**0.040471**	**0.039044**	0.134621	0.125586	0.150839	0.109953	0.197186	**0.143637**	**0.033396**	**0.035911**	0.307904	0.122486	0.205879	0.220724	0.13181	**0.197761**	**0.0754**	0.192408	0.277727	0.183581	0.235614	0.110387	**0.199943**	**0.062585**	**0.9615**	0.161801	0.056058	0.265887	0.17968	0.164795	**0.165644**	**0.074616**	**0.517506**
832.75104	TG 16:0-16:0-18:1	Triacylglyce	C53H100O	0.15074	0.095688	0.044397	0.080122	0.047441	**0.083677**	**0.043331**	0.165305	0.148717	0.152635	0.07976	0.128896	**0.135063**	**0.033564**	**0.069331**	0.107753	0.107967	0.114115	0.090554	0.144563	**0.112991**	**0.019711**	**0.20584**	0.299595	0.102298	0.177323	0.197003	0.126481	**0.18054**	**0.076648**	0.17692	0.237167	0.162334	0.193516	0.097993	**0.173586**	**0.050724**	**0.869858**	0.133653	0.046131	0.244392	0.177482	0.161577	**0.152647**	**0.072146**	**0.569863**
834.76647	TG 16:0-16:0-18:0	Triacylglyce	C53H102O	0.085665	0.06769	0.033742	0.050737	0.030204	**0.053608**	**0.023327**	0.098242	0.069245	0.069775	0.03731	0.067475	**0.068409**	**0.021567**	**0.327958**	0.062512	0.057469	0.053839	0.042791	0.064349	**0.056192**	**0.008562**	**0.821934**	0.128563	0.055052	0.094078	0.102293	0.059442	**0.087886**	**0.030772**	0.084207	0.135332	0.077743	0.076624	0.054646	**0.08571**	**0.029898**	**0.912537**	0.100683	0.033554	0.115827	0.091236	0.083215	**0.084903**	**0.031165**	**0.882735**
836.6796	TG 15:0-18:3-18:3	Triacylglyce	C54H92O6	7.85E-05	6.68E-05	0.001086	0.001561	0.001064	**0.000771**	**0.000668**	0.003351	0.003889	0.003244	0.001139	0.002785	**0.002882**	**0.00105**	**0.005301**	0.000116	0.0024	0.002721	0.001839	0.004161	**0.002248**	**0.001468**	**0.074853**	0.00319	0.002844	0.003958	0.005506	0.002233	**0.003546**	**0.001261**	0.001828	0.000125	0.00231	0.002993	6.84E-05	**0.001465**	**0.001316**	**0.033948**	0.000134	6.87E-05	0.000117	0.003132	0.003206	**0.001331**	**0.001678**	**0.045963**
840.71944	TG 15:1-18:1-18:2	Triacylglyce	C54H96O6	0.006562	0.0036	0.002126	0.003594	0.00227	**0.00363**	**0.001783**	0.007024	0.008338	0.00784	0.004031	0.005851	**0.006617**	**0.001725**	**0.027389**	0.007749	0.006693	0.00765	0.005516	0.008933	**0.007308**	**0.001279**	**0.005635**	0.013607	0.005627	0.009881	0.013177	0.007327	**0.009924**	**0.003513**	0.009038	0.01191	0.011602	0.010405	0.005313	**0.009654**	**0.002677**	**0.894565**	0.00748	0.003065	0.012569	0.009393	0.008194	**0.00814**	**0.003442**	**0.440791**
842.73512	TG 16:0-17:1-18:2	Triacylglyce	C54H98O6	0.027552	0.01423	0.009612	0.015182	0.00843	**0.015001**	**0.007589**	0.025429	0.032139	0.027953	0.014302	0.020962	**0.024157**	**0.006839**	**0.080021**	0.023416	0.021714	0.02596	0.018727	0.035096	**0.024983**	**0.006235**	**0.052699**	0.053458	0.021654	0.036045	0.042856	0.024247	**0.035652**	**0.013183**	0.034339	0.044891	0.037042	0.040502	0.020207	**0.035396**	**0.009364**	**0.972652**	0.025785	0.00993	0.045841	0.030894	0.028336	**0.028157**	**0.012827**	**0.388834**
844.65715	TG 16:3-18:3-18:3	Triacylglyce	C55H88O6	0.004345	0.004045	0.006115	0.005943	0.004146	**0.004919**	**0.001021**	0.007963	0.007726	0.006394	0.00673	0.007567	**0.007276**	**0.000677**	**0.002612**	0.013292	0.008986	0.010388	0.005224	0.01508	**0.010594**	**0.003836**	**0.012679**	0.012003	0.011291	0.015849	0.025678	0.012584	**0.015481**	**0.005962**	0.024871	0.014245	0.010965	0.009255	0.007134	**0.013294**	**0.006975**	**0.608537**	0.012912	0.012814	0.020657	0.016097	0.017053	**0.015907**	**0.003258**	**0.892089**
844.75082	TG 16:1-17:0-18:1	Triacylglyce	C54H100O	0.050589	0.031583	0.016923	0.028362	0.016773	**0.028846**	**0.01386**	0.056986	0.060129	0.05616	0.025887	0.039291	**0.047691**	**0.014653**	**0.070104**	0.040689	0.037402	0.04483	0.036003	0.063635	**0.044512**	**0.011218**	**0.085057**	0.107559	0.042344	0.070681	0.071494	0.044642	**0.067344**	**0.026391**	0.061334	0.09246	0.064201	0.077548	0.039343	**0.066977**	**0.019776**	**0.980758**	0.050864	0.017718	0.086566	0.06256	0.058812	**0.055304**	**0.024873**	**0.479095**
846.76645	TG 16:0-17:0-18:1	Triacylglyce	C54H102O	0.049561	0.030065	0.016544	0.025814	0.014669	**0.027331**	**0.01397**	0.0538	0.048264	0.043825	0.021787	0.036973	**0.04093**	**0.012348**	**0.141553**	0.033723	0.029929	0.034506	0.030229	0.047069	**0.035091**	**0.007**	**0.299016**	0.099672	0.032936	0.055321	0.058802	0.036083	**0.056563**	**0.026658**	0.048726	0.078097	0.048044	0.059955	0.031967	**0.053358**	**0.01705**	**0.826512**	0.043343	0.015387	0.065287	0.049448	0.043744	**0.043442**	**0.018031**	**0.388601**
848.68839	TG 16:1-18:3-18:3	Triacylglyce	C55H92O6	0.00945	0.011461	0.007804	0.012051	0.008252	**0.009804**	**0.001893**	0.019164	0.018114	0.016679	0.010824	0.016214	**0.016199**	**0.003224**	**0.005053**	0.024385	0.019808	0.019313	0.010898	0.02776	**0.020433**	**0.006361**	**0.007178**	0.027274	0.01898	0.029113	0.041373	0.030587	**0.029466**	**0.00803**	0.026596	0.028151	0.023602	0.016198	0.016204	**0.02215**	**0.005671**	**0.134708**	0.032078	0.024329	0.041709	0.026268	0.029673	**0.030811**	**0.006788**	**0.782026**
848.78207	TG 16:0-17:0-18:0	Triacylglyce	C54H104O	0.022718	0.018354	0.010571	0.015151	0.008574	**0.015074**	**0.005738**	0.025882	0.021608	0.019224	0.009716	0.018574	**0.019001**	**0.005929**	**0.31828**	0.016739	0.015402	0.014282	0.01253	0.018621	**0.015515**	**0.002323**	**0.877341**	0.038989	0.015661	0.028737	0.027751	0.014811	**0.02519**	**0.0101**	0.023135	0.034702	0.020225	0.024105	0.013709	**0.023175**	**0.007616**	**0.730986**	0.024976	0.008544	0.031359	0.022182	0.023287	**0.02207**	**0.008354**	**0.60901**
850.70385	TG 16:0-18:3-18:3	Triacylglyce	C55H94O6	0.019562	0.023531	0.022813	0.027595	0.019654	**0.022631**	**0.003308**	0.054073	0.047372	0.046084	0.029461	0.040029	**0.043404**	**0.009254**	**0.00149**	0.080117	0.058172	0.061773	0.031531	0.072519	**0.060822**	**0.01854**	**0.001913**	0.05236	0.040712	0.062284	0.096817	0.05392	**0.061219**	**0.021335**	0.051518	0.058815	0.043328	0.036008	0.035849	**0.045104**	**0.010009**	**0.164764**	0.061871	0.050065	0.086785	0.060784	0.067663	**0.065433**	**0.013522**	**0.718752**
852.71956	TG 16:0-18:2-18:3	Triacylglyce	C55H96O6	0.045522	0.054238	0.037739	0.062622	0.043065	**0.048637**	**0.009829**	0.106137	0.103774	0.09762	0.062508	0.096282	**0.093264**	**0.017679**	**0.001145**	0.173384	0.127539	0.136795	0.074691	0.172309	**0.136944**	**0.040443**	**0.001456**	0.116614	0.090415	0.13467	0.197504	0.111255	**0.130092**	**0.040854**	0.095528	0.11214	0.090791	0.077643	0.071215	**0.089463**	**0.016012**	**0.072185**	0.129779	0.092772	0.180309	0.121418	0.134646	**0.131785**	**0.031608**	**0.943371**
854.73521	TG 16:0-18:2-18:2	Triacylglyce	C55H98O6	0.060522	0.058892	0.033017	0.048987	0.040083	**0.0483**	**0.011865**	0.089307	0.103684	0.094292	0.059338	0.088902	**0.087105**	**0.016625**	**0.002806**	0.125057	0.101543	0.116998	0.069973	0.13661	**0.110036**	**0.025775**	**0.001248**	0.118238	0.06898	0.105791	0.177588	0.100623	**0.114244**	**0.039794**	0.090602	0.108603	0.182089	0.09532	0.05953	**0.107229**	**0.045551**	**0.801914**	0.087878	0.054757	0.163748	0.107559	0.112333	**0.105255**	**0.039777**	**0.730157**
856.75081	TG 16:0-18:1-18:2	Triacylglyce	C55H100O	0.055918	0.039538	0.025593	0.031184	0.023225	**0.035092**	**0.013227**	0.056504	0.065826	0.065429	0.037339	0.05933	**0.056885**	**0.011631**	**0.024418**	0.053273	0.057946	0.055408	0.038816	0.065564	**0.054201**	**0.009775**	**0.031709**	0.088608	0.043409	0.071029	0.092304	0.059462	**0.070962**	**0.020361**	0.063284	0.080009	0.071375	0.071028	0.039712	**0.065082**	**0.015367**	**0.62016**	0.053213	0.025206	0.108205	0.073721	0.06465	**0.064999**	**0.030271**	**0.72419**
858.76667	TG 16:0-18:1-18:1	Triacylglyce	C55H102O	0.108719	0.064646	0.033915	0.053877	0.036076	**0.059447**	**0.030347**	0.113713	0.105192	0.110086	0.071867	0.117595	**0.103691**	**0.018369**	**0.023598**	0.083898	0.088699	0.082087	0.07065	0.104068	**0.08588**	**0.012135**	**0.108143**	0.193435	0.070003	0.133776	0.182328	0.114491	**0.138807**	**0.050569**	0.11692	0.158481	0.120661	0.126414	0.068813	**0.118258**	**0.032159**	**0.465266**	0.088948	0.038863	0.23126	0.149079	0.129593	**0.127549**	**0.071727**	**0.781519**
860.78212	TG 16:0-18:0-18:1	Triacylglyce	C55H104O	0.070461	0.046417	0.021316	0.035498	0.021437	**0.039026**	**0.020483**	0.072917	0.064187	0.05732	0.039704	0.076617	**0.062149**	**0.014633**	**0.074052**	0.042503	0.044463	0.046503	0.037498	0.059424	**0.046078**	**0.008175**	**0.494924**	0.114465	0.041475	0.073709	0.095382	0.064721	**0.07795**	**0.02811**	0.066505	0.10379	0.06261	0.07394	0.038156	**0.069**	**0.023628**	**0.600606**	0.063077	0.021749	0.115099	0.092543	0.077865	**0.074067**	**0.034991**	**0.851401**
862.79785	TG 16:0-18:0-18:0	Triacylglyce	C55H106O	0.060053	0.052502	0.019633	0.047507	0.020231	**0.039985**	**0.018844**	0.081987	0.047589	0.048203	0.023561	0.052367	**0.050742**	**0.020821**	**0.416652**	0.039048	0.038661	0.027518	0.024398	0.035096	**0.032944**	**0.006653**	**0.453491**	0.0885	0.04907	0.078266	0.060883	0.035434	**0.06243**	**0.021448**	0.079477	0.107718	0.049781	0.050361	0.04759	**0.066986**	**0.026286**	**0.771651**	0.079924	0.036087	0.066544	0.054837	0.048461	**0.057171**	**0.016818**	**0.677486**
870.76643	TG 17:1-18:1-18:1	Triacylglyce	C56H102O	0.013937	0.009416	0.004216	0.006843	0.004488	**0.00778**	**0.004031**	0.014109	0.016621	0.014716	0.007291	0.01146	**0.012839**	**0.00361**	**0.069958**	0.011729	0.012092	0.012827	0.010317	0.016157	**0.012624**	**0.002176**	**0.045627**	0.028499	0.011087	0.01835	0.020719	0.013027	**0.018336**	**0.006886**	0.017245	0.024432	0.018211	0.019562	0.00986	**0.017862**	**0.005258**	**0.905568**	0.014415	0.004974	0.023091	0.017386	0.015566	**0.015086**	**0.006563**	**0.466813**
872.68819	TG 14:3-18:3-22:3	Triacylglyce	C57H92O6	0.044768	0.059689	0.042332	0.070004	0.049198	**0.053198**	**0.011508**	0.103356	0.093437	0.09764	0.058797	0.082064	**0.087059**	**0.01762**	**0.007006**	0.150572	0.102989	0.11666	0.054249	0.150117	**0.114917**	**0.0398**	**0.010368**	0.158634	0.117908	0.148338	0.241723	0.157898	**0.1649**	**0.046024**	0.132045	0.161865	0.115394	0.091017	0.097509	**0.119566**	**0.028565**	**0.098193**	0.184376	0.147543	0.247117	0.1638	0.172018	**0.182971**	**0.038273**	**0.518683**
874.70406	TG 18:2-18:3-18:3	Triacylglyce	C57H94O6	0.131475	0.180288	0.117224	0.199846	0.148111	**0.155389**	**0.034197**	0.316399	0.282198	0.265729	0.160035	0.246409	**0.254154**	**0.058552**	**0.011579**	0.417301	0.308343	0.34377	0.164907	0.453961	**0.337656**	**0.112482**	**0.008484**	0.369988	0.26866	0.377312	0.514677	0.381043	**0.382336**	**0.087487**	0.325678	0.384411	0.274521	0.207464	0.222681	**0.282951**	**0.073351**	**0.08746**	0.454724	0.353633	0.583096	0.361634	0.437514	**0.43812**	**0.092577**	**0.35612**
876.71936	TG 18:2-18:2-18:3	Triacylglyce	C57H96O6	0.035805	0.042996	0.046911	0.045216	0.029195	**0.040025**	**0.007387**	0.063271	0.062629	0.058147	0.032779	0.057058	**0.054777**	**0.012592**	**0.053764**	0.076729	0.060145	0.069623	0.032811	0.078048	**0.063471**	**0.018555**	**0.030406**	0.054092	0.039137	0.0589	0.082154	0.0573	**0.058317**	**0.015454**	0.055944	0.061614	0.047738	0.036806	0.039191	**0.048258**	**0.010619**	**0.264675**	0.077655	0.054081	0.092194	0.064715	0.06347	**0.070423**	**0.014784**	**0.241211**
876.71963	TG 18:1-18:2-18:4	Triacylglyce	C57H96O6	0.066244	0.093231	0.058534	0.100696	0.069753	**0.077692**	**0.018247**	0.139502	0.134039	0.131793	0.081723	0.126102	**0.122632**	**0.023368**	**0.009508**	0.20737	0.166767	0.168078	0.089012	0.243788	**0.175003**	**0.05764**	**0.006992**	0.16011	0.118584	0.153975	0.259758	0.171911	**0.172868**	**0.052481**	0.14341	0.152794	0.134129	0.093206	0.098663	**0.12444**	**0.026915**	**0.103684**	0.188518	0.140147	0.277057	0.171456	0.185408	**0.192517**	**0.05099**	**0.564813**
878.73496	TG 18:2-18:2-18:2	Triacylglyce	C57H98O6	0.023735	0.03326	0.025798	0.022917	0.017797	**0.024701**	**0.005618**	0.029817	0.028924	0.031846	0.018372	0.037812	**0.029354**	**0.007049**	**0.281736**	0.04612	0.033823	0.04124	0.020808	0.044097	**0.037218**	**0.01029**	**0.044051**	0.027476	0.020236	0.03253	0.053102	0.038717	**0.034412**	**0.012455**	0.029882	0.026944	0.107156	0.024481	0.019338	**0.04156**	**0.036872**	**0.692072**	0.029532	0.024382	0.048949	0.033944	0.033214	**0.034004**	**0.009173**	**0.954421**
878.73508	TG 18:0-18:3-18:3	Triacylglyce	C57H98O6	0.047195	0.070659	0.033883	0.05138	0.042596	**0.049143**	**0.01367**	0.058937	0.0689	0.071627	0.044465	0.082766	**0.065339**	**0.014428**	**0.105896**	0.108878	0.082742	0.106178	0.053455	0.119894	**0.094229**	**0.026507**	**0.009637**	0.078961	0.05853	0.080923	0.173883	0.112179	**0.100895**	**0.045082**	0.073539	0.07116	0.350028	0.069121	0.050545	**0.122879**	**0.127307**	**0.725307**	0.070187	0.060051	0.145551	0.09507	0.089122	**0.091996**	**0.033098**	**0.731183**
880.75054	TG 18:1-18:2-18:2	Triacylglyce	C57H100O	0.049097	0.067911	0.022172	0.03158	0.034452	**0.041042**	**0.01786**	0.050637	0.056972	0.059838	0.037879	0.070579	**0.055181**	**0.012062**	**0.180571**	0.080383	0.064802	0.085149	0.043256	0.080176	**0.070753**	**0.017177**	**0.027881**	0.07826	0.042695	0.070371	0.17445	0.109123	**0.09498**	**0.050335**	0.059215	0.062301	0.134429	0.067065	0.039473	**0.072497**	**0.03618**	**0.440814**	0.042622	0.031491	0.123132	0.081309	0.070303	**0.069772**	**0.035997**	**0.38898**
882.76616	TG 18:1-18:1-18:2	Triacylglyce	C57H102O	0.053082	0.056316	0.020444	0.028878	0.031469	**0.038038**	**0.015788**	0.05553	0.055871	0.060624	0.03519	0.066922	**0.054827**	**0.011907**	**0.094178**	0.06214	0.062528	0.065981	0.037268	0.068545	**0.059292**	**0.012589**	**0.046416**	0.087528	0.041732	0.072431	0.13191	0.085715	**0.083863**	**0.032524**	0.054309	0.0615	0.077785	0.061867	0.039488	**0.05899**	**0.013875**	**0.154378**	0.037406	0.026601	0.114659	0.073734	0.062404	**0.062961**	**0.034512**	**0.353195**
884.78168	TG 18:1-18:1-18:1	Triacylglyce	C57H104O	0.024183	0.020118	0.018183	0.011822	0.010279	**0.016917**	**0.005802**	0.022542	0.024454	0.026845	0.016337	0.02317	**0.02267**	**0.003905**	**0.103159**	0.019789	0.019404	0.018502	0.01315	0.023602	**0.018889**	**0.003754**	**0.541221**	0.026101	0.012394	0.025509	0.038305	0.026998	**0.025861**	**0.009188**	0.019398	0.023098	0.023932	0.019858	0.014234	**0.020104**	**0.003829**	**0.231972**	0.016765	0.010421	0.044818	0.027549	0.026486	**0.025208**	**0.013054**	**0.929309**
884.78201	TG 18:0-18:1-18:2	Triacylglyce	C57H104O	0.072175	0.066003	0.025274	0.031268	0.036146	**0.046173**	**0.021382**	0.073662	0.070556	0.073189	0.047984	0.075674	**0.068213**	**0.011455**	**0.076661**	0.056679	0.060792	0.056031	0.044364	0.076893	**0.058952**	**0.011745**	**0.2752**	0.119708	0.046878	0.099008	0.164109	0.102525	**0.106446**	**0.04219**	0.059231	0.091803	0.07609	0.074084	0.045929	**0.069427**	**0.017485**	**0.107484**	0.050114	0.031053	0.173229	0.090382	0.090331	**0.087022**	**0.054655**	**0.546852**
886.79734	TG 18:0-18:1-18:1	Triacylglyce	C57H106O	0.037568	0.032483	0.01268	0.02074	0.017679	**0.02423**	**0.010422**	0.038195	0.035923	0.035258	0.020804	0.0405	**0.034136**	**0.007733**	**0.126242**	0.030723	0.029868	0.030058	0.024584	0.034249	**0.029896**	**0.00346**	**0.281897**	0.062828	0.024708	0.047206	0.066491	0.040964	**0.04844**	**0.016982**	0.035379	0.052694	0.0382	0.042582	0.023082	**0.038387**	**0.010789**	**0.296324**	0.03328	0.014544	0.072704	0.044448	0.041379	**0.041271**	**0.021073**	**0.570032**
890.81661	TG 16:0-18:0-20:0	Triacylglyce	C57H110O	0.013041	0.010842	0.007272	0.009221	0.004751	**0.009026**	**0.003194**	0.014944	0.013312	0.01335	0.006657	0.011966	**0.012046**	**0.003192**	**0.173151**	0.011979	0.010476	0.009046	0.007472	0.011161	**0.010027**	**0.001788**	**0.557788**	0.019913	0.010452	0.014816	0.016744	0.010773	**0.01454**	**0.004022**	0.019362	0.020084	0.013405	0.01378	0.010836	**0.015493**	**0.004032**	**0.717797**	0.018059	0.007959	0.02027	0.015583	0.014071	**0.015188**	**0.004683**	**0.820189**
890.82881	TG 14:0-16:0-24:0	Triacylglyce	C57H110O	0.054207	0.044427	0.015703	0.083341	0.015605	**0.042657**	**0.028505**	0.078471	0.045343	0.035728	0.013177	0.047204	**0.043985**	**0.023552**	**0.937973**	0.020681	0.032544	0.016148	0.014769	0.021166	**0.021062**	**0.006995**	**0.138544**	0.072014	0.047283	0.084857	0.047461	0.023724	**0.055068**	**0.023851**	0.071696	0.096303	0.035301	0.044323	0.042687	**0.058062**	**0.025453**	**0.852582**	0.071194	0.039609	0.050468	0.029595	0.033137	**0.0448**	**0.016755**	**0.453602**
898.79698	TG 18:1-18:1-19:1	Triacylglyce	C58H106O	0.004583	0.003427	0.001224	0.002527	0.001528	**0.002658**	**0.001383**	0.00569	0.005092	0.005297	0.002516	0.003734	**0.004466**	**0.001315**	**0.066969**	0.003663	0.003565	0.004164	0.003085	0.003929	**0.003681**	**0.000407**	**0.151166**	0.011435	0.003471	0.006725	0.006328	0.004805	**0.006553**	**0.00302**	0.005004	0.008124	0.006078	0.005996	0.003531	**0.005746**	**0.00168**	**0.615908**	0.004672	0.002	0.008573	0.005321	0.004129	**0.004939**	**0.002383**	**0.375628**
900.81273	TG 15:1-16:1-24:0	Triacylglyce	C58H108O	0.009805	0.007189	0.00336	0.00545	0.003466	**0.005854**	**0.002715**	0.010859	0.01082	0.008823	0.004361	0.008366	**0.008646**	**0.00265**	**0.138491**	0.00742	0.006466	0.007224	0.006849	0.010176	**0.007627**	**0.001471**	**0.235123**	0.019201	0.007318	0.01379	0.013278	0.007218	**0.012161**	**0.005034**	0.012421	0.015585	0.011221	0.013202	0.006198	**0.011725**	**0.003477**	**0.877442**	0.009988	0.003553	0.015144	0.009609	0.009844	**0.009628**	**0.004108**	**0.408682**

904.74945	TG 18:2-18:3-20:2	Triacyglyce	C59H100O	0.006284	0.041839	0.003838	0.007563	0.012117	0.014328	0.015671	0.005446	0.005897	0.005971	0.003545	0.005483	0.005268	0.000992	0.233027	0.008963	0.01046	0.009033	0.015929	0.009706	0.010818	0.002921	0.635693	0.00921	0.015282	0.009393	0.042146	0.012522	0.017711	0.013886	0.007022	0.006904	0.008251	0.004766	0.004481	0.006285	0.001609	0.105021	0.008871	0.006955	0.010819	0.007087	0.00905	0.008556	0.001596	0.181239
904.8441	TG 15:0-16:0-24:0	Triacyglyce	C58H112O	0.017209	0.013285	0.007546	0.012685	0.007155	0.011576	0.004233	0.017743	0.016389	0.01626	0.006934	0.013919	0.014249	0.004314	0.351629	0.011256	0.011989	0.010332	0.010079	0.013138	0.011359	0.001251	0.915158	0.029404	0.012858	0.018744	0.022172	0.01194	0.019024	0.007175	0.018649	0.025258	0.016455	0.018588	0.009943	0.017779	0.005488	0.765833	0.019779	0.006813	0.022083	0.015991	0.016465	0.016226	0.005823	0.51757
906.7652	TG 18:3-18:3-20:0	Triacyglyce	C59H102O	0.011403	0.064324	0.004624	0.010118	0.025502	0.023194	0.024248	0.006499	0.005908	9.08E-05	0.003601	0.005747	0.004369	0.002631	0.122648	0.00979	0.012926	0.012989	0.026046	0.011659	0.014682	0.006484	0.470003	0.014474	0.024734	0.015657	0.074927	0.021697	0.030298	0.025305	0.007993	0.006994	0.015114	0.005035	0.004505	0.007928	0.00426	0.087098	0.007164	0.0109	0.010387	0.006184	0.015004	0.009928	0.003485	0.11241
908.77978	TG 18:2-18:2-20:1	Triacyglyce	C59H104O	0.013958	0.063719	0.004829	0.010881	0.02969	0.024615	0.02371	0.006761	0.00676	0.006938	0.003863	0.006247	0.006114	0.001285	0.119616	0.009259	0.013957	0.015524	0.02763	0.010728	0.015419	0.007266	0.431027	0.01472	0.028233	0.015319	0.077042	0.023272	0.031717	0.02596	0.007726	0.007541	0.009684	0.006588	0.00436	0.00718	0.001937	0.068116	0.007	0.009787	0.012255	0.007049	0.015387	0.010295	0.003585	0.104981
910.7971	TG 18:1-18:2-20:1	Triacyglyce	C59H106O	0.009947	0.037382	0.003444	0.007651	0.019844	0.015653	0.013559	0.008875	0.007773	0.008014	0.004329	0.00751	0.0073	0.001738	0.208976	0.009527	0.0105	0.010824	0.016593	0.010285	0.011546	0.002862	0.526097	0.015887	0.018295	0.014116	0.057421	0.015758	0.024295	0.018578	0.009632	0.010537	0.009938	0.008426	0.004477	0.008602	0.002431	0.097962	0.007228	0.006721	0.010932	0.007819	0.012369	0.009014	0.00249	0.105765
912.81262	TG 18:1-18:1-20:1	Triacyglyce	C59H108O	0.010441	0.032205	0.003543	0.007789	0.018132	0.014422	0.011272	0.010656	0.008931	0.008319	0.004198	0.007931	0.008007	0.002371	0.248254	0.007044	0.009778	0.009342	0.014001	0.009566	0.009946	0.00252	0.411451	0.018623	0.015168	0.013428	0.048351	0.015157	0.022146	0.01477	0.010661	0.014921	0.01112	0.010048	0.005643	0.010479	0.003307	0.123077	0.008671	0.005481	0.014691	0.010001	0.013839	0.010537	0.003791	0.12711
914.81643	TG 18:0-18:2-20:0	Triacyglyce	C59H110O	0.007503	0.010937	0.004466	0.004577	0.006275	0.006751	0.002658	0.007329	0.007735	0.00662	0.004169	0.007636	0.006698	0.001479	0.969432	0.006189	0.006751	0.007741	0.007582	0.007557	0.007164	0.000668	0.745138	0.010722	0.006466	0.00909	0.020949	0.010354	0.011516	0.005531	0.008359	0.010118	0.007662	0.009181	0.005138	0.008092	0.001889	0.226483	0.008097	0.003559	0.013827	0.009456	0.009028	0.008793	0.003664	0.385609
914.82844	TG 16:1-18:1-22:0	Triacyglyce	C59H110O	0.020561	0.019368	0.005818	0.011438	0.011358	0.013708	0.006163	0.019174	0.018036	0.017243	0.008714	0.015874	0.015808	0.004144	0.544836	0.014026	0.014001	0.014466	0.015117	0.018965	0.015315	0.00209	0.59605	0.038097	0.014821	0.0246	0.03388	0.015934	0.025467	0.010432	0.02372	0.030669	0.020653	0.023429	0.012302	0.022155	0.006631	0.565678	0.019488	0.006476	0.02702	0.018854	0.018838	0.018135	0.007378	0.235396
916.84401	TG 16:0-16:1-24:0	Triacyglyce	C59H112O	0.032781	0.023384	0.008849	0.020711	0.011036	0.019352	0.00972	0.031746	0.031167	0.025637	0.012183	0.026842	0.025515	0.00791	0.303498	0.017288	0.022731	0.018538	0.020747	0.027307	0.021322	0.003942	0.685565	0.063125	0.022105	0.042282	0.045675	0.021517	0.038941	0.017524	0.035664	0.04803	0.030121	0.038183	0.019068	0.034213	0.010664	0.62026	0.034316	0.010643	0.045695	0.03189	0.029429	0.030394	0.012676	0.402692
918.85943	TG 16:0-16:0-24:0	Triacyglyce	C59H114O	0.017653	0.014869	0.006676	0.018408	0.007884	0.013098	0.005489	0.018717	0.016163	0.014412	0.006299	0.015001	0.014118	0.004673	0.75966	0.010537	0.012012	0.009022	0.009269	0.012619	0.010692	0.001604	0.374331	0.027376	0.01376	0.019596	0.023708	0.011581	0.019204	0.006614	0.019197	0.029734	0.018772	0.018103	0.009191	0.019	0.007286	0.96403	0.021884	0.007671	0.025065	0.017353	0.016972	0.017789	0.006575	0.74309
928.84366	TG 16:1-17:1-24:0	Triacyglyce	C60H112O	0.012178	0.008773	0.003801	0.006097	0.004182	0.007006	0.003498	0.011949	0.012625	0.010356	0.004788	0.010084	0.00996	0.003082	0.19423	0.007947	0.008015	0.008505	0.007811	0.011207	0.008697	0.001428	0.346326	0.021107	0.008634	0.014795	0.016018	0.008523	0.013815	0.005335	0.014153	0.017732	0.012046	0.014707	0.007292	0.013186	0.003871	0.836271	0.013049	0.004444	0.015637	0.011408	0.009806	0.010869	0.004189	0.35979
930.85941	TG 16:1-18:0-23:0	Triacyglyce	C60H114O	0.022052	0.016147	0.007399	0.014039	0.007808	0.013489	0.006125	0.023064	0.023549	0.020669	0.008929	0.017963	0.018835	0.005966	0.199627	0.014342	0.016271	0.014685	0.014948	0.020892	0.016227	0.002708	0.387238	0.040606	0.017349	0.026871	0.032345	0.013926	0.02622	0.010894	0.026472	0.032753	0.023124	0.026739	0.014316	0.024681	0.006754	0.795178	0.023827	0.007672	0.031538	0.021604	0.020174	0.020963	0.008627	0.422217
932.78096	TG 18:3-18:3-22:1	Triacyglyce	C61H104O	0.001065	0.008703	0.000206	0.001558	0.002258	0.002758	0.003406	9.79E-05	9.27E-05	9.08E-05	4.29E-05	7.84E-05	8.05E-05	2.22E-05	0.116872	0.001618	0.00195	9.09E-05	0.003561	8.68E-05	0.001461	0.001453	0.45618	7.84E-05	0.002918	0.000102	0.007662	0.003295	0.002811	0.003106	0.000117	0.001324	0.000108	0.00007	6.84E-05	0.000337	0.000552	0.117592	0.001794	0.001636	0.000117	9.28E-05	0.001797	0.001087	0.000899	0.267354
932.87488	TG 15:0-16:0-26:0	Triacyglyce	C60H116O	0.01137	0.009208	0.004892	0.008375	0.004819	0.007733	0.002845	0.011967	0.010589	0.009942	0.004773	0.010225	0.009499	0.002754	0.347735	0.007759	0.008131	0.007073	0.006471	0.009242	0.007735	0.001057	0.998658	0.020569	0.009204	0.014214	0.016715	0.009542	0.014049	0.004833	0.013205	0.019051	0.01245	0.012481	0.00693	0.012823	0.004299	0.683012	0.014694	0.005423	0.017913	0.011471	0.011582	0.012216	0.004629	0.55735
934.79564	TG 18:2-18:3-22:1	Triacyglyce	C61H106O	0.004036	0.019401	0.001212	0.002737	0.006158	0.006709	0.007324	0.002536	0.002	9.08E-05	4.29E-05	0.001432	0.00122	0.001123	0.136223	0.002841	0.003745	0.004066	0.007468	0.002966	0.004217	0.001889	0.482352	0.004219	0.00673	0.004903	0.01808	0.005935	0.007973	0.005731	0.002499	0.002112	0.000108	0.00138	0.00172	0.001563	0.000915	0.038734	0.00292	0.002717	0.005587	0.002873	0.003689	0.003557	0.001195	0.130152
936.81079	TG 18:3-20:1-20:1	Triacyglyce	C61H108O	0.006291	0.029098	0.000206	0.004842	0.010051	0.010098	0.011191	9.79E-05	0.002646	0.003157	0.001513	0.002688	0.00202	0.001233	0.147345	0.003506	0.005547	0.00499	0.011311	0.004612	0.005993	0.003065	0.45178	0.006623	0.01067	0.006833	0.032517	0.008888	0.013106	0.010976	0.003566	0.004358	0.004093	0.00007	6.84E-05	0.002431	0.002175	0.065456	0.000134	0.003975	0.005064	0.002769	0.006834	0.003755	0.002516	0.100422
938.82801	TG 18:2-20:1-20:1	Triacyglyce	C61H110O	7.85E-05	0.026871	0.001677	0.004442	0.011315	0.008877	0.010939	0.005226	0.004937	0.004074	0.002629	0.005206	0.004415	0.001102	0.390649	0.005274	0.005935	0.006979	4.85E-05	0.005245	0.004696	0.002692	0.43075	0.007278	0.011948	0.00842	1E-04	8.81E-05	0.005567	0.005284	0.004791	0.006596	0.006643	0.004727	0.002681	0.005087	0.001636	0.851159	0.004344	0.003854	0.00785	0.004886	9.26E-05	0.004206	0.002775	0.623808
940.84327	TG 18:2-20:0-20:1	Triacyglyce	C61H112O	0.007888	0.018215	0.002734	0.004808	0.008233	0.008376	0.00595	0.007412	0.007454	0.006727	0.003158	0.006811	0.006313	0.001795	0.479175	0.005881	0.006394	0.006925	0.008011	0.007295	0.006901	0.00082	0.59806	0.012955	0.007553	0.009618	0.024213	0.008617	0.012591	0.006805	0.008576	0.010712	0.008619	0.00839	0.004159	0.008091	0.002395	0.200537	0.00684	0.003755	0.010544	0.007347	0.008696	0.007436	0.002508	0.150618
942.85891	TG 16:1-18:1-24:0	Triacyglyce	C61H114O	0.016735	0.014005	0.004715	0.010834	0.006907	0.010639	0.004938	0.01682	0.017428	0.013668	0.007389	0.014464	0.013954	0.003992	0.27673	0.011936	0.0135	0.01172	0.011615	0.014419	0.012638	0.001255	0.405942	0.031501	0.012761	0.023146	0.02956	0.012581	0.02191	0.008983	0.019644	0.024472	0.017378	0.021572	0.010621	0.018737	0.005231	0.514243	0.019276	0.006489	0.025156	0.019279	0.016306	0.017301	0.006845	0.388188
944.8746	TG 16:0-18:1-24:0	Triacyglyce	C61H116O	0.020238	0.014533	0.005752	0.013356	0.007644	0.012305	0.005781	0.019248	0.018618	0.014932	0.007769	0.015737	0.015261	0.004572	0.395994	0.011707	0.01359	0.011349	0.012958	0.01754	0.013429	0.002472	0.69975	0.034238	0.01461	0.022701	0.02816	0.013461	0.022634	0.008856	0.021307	0.029697	0.020992	0.023227	0.011076	0.021259	0.006686	0.788826	0.022888	0.006738	0.029009	0.019844	0.019837	0.019663	0.008137	0.595802
946.89025	TG 16:0-18:0-24:0	Triacyglyce	C61H118O	0.006876	0.005816	0.002711	0.005611	0.003291	0.004861	0.001776	0.007075	0.006628	0.006606	0.002627	0.006394	0.005866	0.001827	0.403593	0.004034	0.004684	0.003349	0.003266	0.004996	0.004066	0.000775	0.385696	0.010975	0.005247	0.008681	0.0096	0.004825	0.007866	0.002713	0.007509	0.011622	0.007953	0.006525	0.003752	0.007472	0.002837	0.828213	0.009206	0.003156	0.010668	0.007211	0.006686	0.007385	0.00285	0.791831
956.87438	TG 16:1-18:1-25:0	Triacyglyce	C62H116O	0.009199	0.005941	0.003593	0.006735	0.003277	0.005749	0.002433	0.009372	0.009997	0.008859	0.00381	0.008046	0.008017	0.002458	0.180727	0.005787	0.007172	0.006886	0.006385	0.008944	0.007035	0.00119	0.31944	0.016357	0.00712	0.010818	0.012566	0.006819	0.010736	0.00398	0.011239	0.014736	0.009865	0.011337	0.006047	0.010645	0.003135	0.968894	0.010632	0.003243	0.012831	0.008612	0.008169	0.008697	0.003566	0.418453
958.88993	TG 16:0-18:1-25:0	Triacyglyce	C62H118O	0.011181	0.007678	0.00351	0.006678	0.003587	0.006527	0.003192	0.010302	0.01002	0.00952	0.004095	0.008412	0.00847	0.00255	0.318647	0.005866	0.007368	0.006707	0.006185	0.009388	0.007103	0.001398	0.721383	0.018529	0.007536	0.012157	0.014116	0.007821	0.012032	0.004597	0.011577	0.015041	0.010744	0.012277	0.005977	0.011123	0.003298	0.728777	0.011954	0.00374	0.014902	0.009812	0.009653	0.010012	0.004099	0.484362
960.90597	TG 16:0-17:0-26:0	Triacyglyce	C62H120O	0.003505	0.003151	0.001577	0.002949	0.001786	0.002594	0.000859	0.003945	0.003037	0.003416	0.00145	0.003818	0.003133	0.001006	0.388462	0.002273	0.002397	0.001927	0.002062	0.003167	0.002365	0.000484	0.618704	0.005486	0.00275	0.00395	0.005319	0.00218	0.003937	0.001484	0.00406	0.005419	0.00439	0.003497	0.00206	0.003885	0.001237	0.953614	0.004423	0.001621	0.005775	0.003213	0.003999	0.003806	0.001535	0.89443
964.84008	TG 18:3-20:1-22:1	Triacyglyce	C63H112O	0.001754	0.005916	0.000206	0.001587	0.002085	0.00231	0.00214	0.002442	0.001909	9.08E-05	0.000914	0.001913	0.001454	0.000941	0.436679	0.001909	0.001931	0.002079	0.002877	0.001978	0.002155	0.000409	0.877486	0.002996	0.002702	0.003036	0.008043	0.002531	0.003861	0.002347	0.002383	0.002714	0.000108	0.001561	0.001272	0.001608	0.001024	0.084546	0.002262	0.001578	0.003097	0.001883	0.002683	0.0023	0.000608	0.187887
966.85849	TG 18:2-20:1-22:1	Triacyglyce	C63H114O	0.002726	0.005298	0.000206	0.001925	0.002059	0.002443	0.001847	0.002836	0.002635	0.002385	0.001352	0.002878	0.002417	0.000627	0.977066	0.003048	0.002775	0.002872	0.002662	0.003201	0.002912	0.000215	0.588476	0.004557	0.002752	0.003896	0.006444	0.003661	0.004262	0.001381	0.003126	0.004347	0.003281	0.002922	0.001664	0.003068	0.000959	0.150917	0.002707	0.001537	0.004643	0.002853	0.003275	0.003003	0.001121	0.152081
968.87382	TG 18:1-18:2-24:0	Triacyglyce	C63H116O	0.004742	0.005043	0.001433	0.002741	0.002226	0.003237	0.001585	0.004564	0.005086	0.004105	0.001886	0.004041	0.003936	0.001221	0.456948	0.00354	0.003838	0.00381	0.003748	0.00443	0.003873	0.000332	0.405651	0.008038	0.004242	0.005494	0.00895	0.004245	0.006194	0.002185	0.005549	0.007213	0.005487	0.005206	0.002818	0.005254	0.001574	0.457859	0.005211	0.001902	0.006189	0.004527	0.004738	0.004514	0.001594	0.202245
970.88959	TG 16:1-18:1-26:0	Triacyglyce	C63H118O	0.008726	0.007058	0.00243	0.005612	0.003687	0.005503	0.002528	0.009194	0.007936	0.007456	0.003441	0.00786	0.007177	0.002188	0.295175	0.005792	0.006949	0.005841	0.006354	0.008322	0.006652	0.001044	0.375061	0.015414	0.006638	0.010503	0.012995	0.006727	0.010455	0.003857	0.010857	0.01286	0.010034	0.011268	0.005587	0.010121	0.002735	0.878293	0.010093	0.003372	0.013113	0.00924	0.008851	0.008934	0.00353	0.533517
972.90479	TG 16:0-18:1-26:0	Triacyglyce	C63H120O	0.006613	0.005248	0.002082	0.004189	0.002475	0.004121	0.001894	0.00625	0.006085	0.006225	0.002458	0.005327	0.005269	0.001616	0.332816	0.003232	0.004572	0.003463	0.003834	0.005348	0.00409	0.000867	0.973796	0.011074	0.004698	0.007616	0.009111	0.004589	0.007418	0.002814	0.007278	0.009908	0.006979	0.007487	0.003607	0.007052	0.002251	0.826079	0.008396	0.002451	0.009459	0.006991	0.006115	0.006682	0.002691	0.683981
974.91315	TG 16:0-18:0-26:0	Triacyglyce	C63H122O	0.002719	0.001469	0.000206	0.001861	0.001046	0.00146	0.000934	0.002027	0.002642	0.002739	0.00129	0.002151	0.00217	0.000579	0.186864	0.001801	0.002245	0.001883	0.001632	0.002602	0.002032	0.000389	0.241651	0.002723	0.001751	0.002649	0.004342	0.002122	0.002717	0.000991	0.002787	0.003838	0.003233	0.002989	0.001702	0.00291	0.000782	0.742006	0.003536	6.87E-05	0.004012	0.002911	0.002839	0.002673	0.001533	0.958221
982.88938	TG 18:1-18:2-25:0	Triacyglyce	C64H118O	0.002538	0.001474	0.000206	0.001152	0.000808	0.001236	0.000866	0.002012	0.00194	0.002004	0.000902	0.001733	0.001718	0.00047	0.305448	0.001377	0.001793	0.0016	0.001438	0.001851	0.001612	0.000209	0.372806	0.003964	0.00174	0.002766	0.003232	0.001391	0.002618	0.001059	0.002468	0.003533	0.002631	0.002665	0.001454	0.00255	0.000741	0.909089	0.002422	0.000681	0.003121	0.001912	0.002033	0.002034	0.000891	0.372708

									Average	Stdev						Average	Stdev	t-test						Average	Stdev	t-test
Mass (M)	Name	Compound	FORMULA (	Ler-R1	Ler-R2	Ler-R3	Ler-R4	Ler-R5			arc3-R1	arc3-R2	arc3-R3	arc3-R4	arc3-R5				arc5-R1	arc5-R2	arc5-R3	arc5-R4	arc5-R5			
774.45519	Arabidopside A	Arabidopsi	C43H66O1	0.048612	0.040292	0.055173	0.043099	0.023144	**0.042064**	**0.012009**	0.060663	0.083485	0.064956	0.050341	0.059942	**0.063877**	**0.012193**	**0.021478**	0.080593	0.127768	0.081635	0.052593	0.081614	**0.084841**	**0.027025**	**0.011977**
802.4869	Arabidopside B	Arabidopsi	C45H70O1	0.000393	0.001952	0.000206	0.001023	0.000544	**0.000824**	**0.000699**	0.00159	0.001868	9.08E-05	4.29E-05	7.84E-05	**0.000734**	**0.000914**	**0.866125**	0.000116	8.25E-05	0.002344	0.001643	0.00295	**0.001427**	**0.001297**	**0.386426**
553.50719	Cer d16:0h18:1	Ceramides	C34H67NC	0.018172	0.030759	0.012253	0.023707	0.019399	**0.020858**	**0.006884**	0.027876	0.026095	0.026094	0.014826	0.027314	**0.024441**	**0.005431**	**0.387567**	0.040353	0.033297	0.029638	0.020177	0.038546	**0.032402**	**0.008045**	**0.040688**
569.50143	Cer t18:1-16:0	Ceramides	C34H67NC	0.016822	0.030319	0.013555	0.025452	0.019778	**0.021185**	**0.006724**	0.040583	0.040075	0.038195	0.02139	0.03668	**0.035385**	**0.007976**	**0.015977**	0.059054	0.040664	0.037314	0.02175	0.050632	**0.041883**	**0.014134**	**0.018234**
571.51723	Cer t18:0h16:0	Ceramides	C34H69NC	0.014668	0.020908	0.009811	0.020602	0.013297	**0.015857**	**0.00481**	0.025637	0.027524	0.025651	0.012565	0.025845	**0.023445**	**0.006133**	**0.061179**	0.043696	0.03321	0.031722	0.014362	0.035456	**0.031689**	**0.010734**	**0.016826**
625.56414	Cer t18:1h20:0	Ceramides	C38H75NC	0.006501	0.008511	0.004374	0.007401	0.00542	**0.006441**	**0.001622**	0.012531	0.012254	0.010913	0.006068	0.01011	**0.010375**	**0.002603**	**0.020886**	0.020656	0.012693	0.012279	0.007885	0.015648	**0.013832**	**0.004715**	**0.010632**
651.57974	Cer t18:2h22:0	Ceramides	C40H77NC	0.003858	0.007504	0.004225	0.006645	0.006103	**0.005667**	**0.001571**	0.013182	0.01036	0.010257	0.005125	0.013075	**0.0104**	**0.003269**	**0.019353**	0.020606	0.011556	0.015412	0.008061	0.014441	**0.014015**	**0.004665**	**0.005299**
653.5954	Cer t18:1h22:0	Ceramides	C40H79NC	0.094807	0.12877	0.071055	0.108084	0.079233	**0.09639**	**0.023043**	0.175104	0.170666	0.154345	0.081754	0.156538	**0.147681**	**0.037913**	**0.032359**	0.303717	0.185168	0.186522	0.101746	0.206652	**0.196761**	**0.072119**	**0.018025**
655.61099	Cer t18:0h22:0	Ceramides	C40H81NC	0.018789	0.034689	0.010464	0.023027	0.022391	**0.021872**	**0.00874**	0.035354	0.032686	0.038809	0.020484	0.035826	**0.032632**	**0.00713**	**0.065472**	0.078937	0.044891	0.046974	0.031979	0.05959	**0.052474**	**0.017739**	**0.008564**
665.59501	Cer t18:2h23:0	Ceramides	C41H79NC	0.026666	0.027797	0.028643	0.026055	0.017919	**0.025416**	**0.004308**	0.045787	0.043932	0.044197	0.020588	0.035781	**0.038057**	**0.010516**	**0.037681**	0.058848	0.050927	0.056747	0.029403	0.044954	**0.048176**	**0.011808**	**0.00369**
667.61087	Cer t18:1h23:0	Ceramides	C41H81NC	0.036262	0.066264	0.018782	0.04396	0.035235	**0.0401**	**0.017265**	0.063623	0.06613	0.06732	0.042053	0.066554	**0.061136**	**0.010758**	**0.049511**	0.157808	0.085991	0.088575	0.057213	0.110798	**0.100077**	**0.03747**	**0.011688**
675.57964	Cer t18:1h24:3	Ceramides	C42H77NC	0.001476	0.002091	0.000206	0.002015	0.001573	**0.001472**	**0.000757**	0.003835	0.002708	0.003128	0.001707	0.002863	**0.002848**	**0.000771**	**0.021529**	0.006735	0.002499	0.002753	0.001024	0.002728	**0.003148**	**0.002129**	**0.135953**
679.6113	Cer t18:2h24:0	Ceramides	C42H81NC	0.297559	0.448978	0.214917	0.391602	0.284236	**0.327458**	**0.092614**	0.622348	0.597585	0.620747	0.288554	0.53046	**0.531939**	**0.141075**	**0.026683**	0.918271	0.599866	0.574707	0.32356	0.695576	**0.622396**	**0.215048**	**0.02261**
681.62716	Cer t18:1h24:0	Ceramides	C42H83NC	0.145792	0.238379	0.094248	0.195045	0.154099	**0.165513**	**0.054267**	0.293974	0.273802	0.286228	0.157978	0.260473	**0.254491**	**0.055429**	**0.033391**	0.457062	0.285878	0.295474	0.17007	0.358141	**0.313325**	**0.105194**	**0.023473**
693.62651	Cer t18:1h25:1	Ceramides	C43H83NC	0.063313	0.079039	0.062548	0.063802	0.048133	**0.063367**	**0.010938**	0.103065	0.120117	0.106091	0.05559	0.101236	**0.09722**	**0.024425**	**0.022201**	0.154586	0.110942	0.115742	0.058544	0.114197	**0.110802**	**0.034219**	**0.018355**
695.64215	Cer t18:1h25:0	Ceramides	C43H85NC	0.011266	0.022346	0.007881	0.0162	0.013593	**0.014257**	**0.00546**	0.026724	0.023758	0.026837	0.015739	0.022842	**0.02318**	**0.004521**	**0.022675**	0.051097	0.025798	0.024821	0.016706	0.03479	**0.030642**	**0.013107**	**0.032592**
707.64213	Cer t18:1h26:1	Ceramides	C44H85NC	0.07154	0.148741	0.055515	0.10805	0.089144	**0.094598**	**0.03606**	0.181761	0.185307	0.199999	0.102683	0.200531	**0.174056**	**0.040786**	**0.011466**	0.303673	0.173941	0.174552	0.107658	0.234797	**0.198924**	**0.073836**	**0.021847**
709.65925	Cer t18:1h26:0	Glycosylce	C44H87NC	0.056548	0.097948	0.037565	0.089331	0.076543	**0.071587**	**0.024587**	0.144858	0.13968	0.156655	0.088104	0.131806	**0.132221**	**0.026262**	**0.005475**	0.228591	0.141393	0.122148	0.06967	0.167113	**0.145783**	**0.058488**	**0.030891**
711.67423	Cer t18:0h26:0	Ceramides	C44H89NC	0.017372	0.031352	0.014525	0.030458	0.033791	**0.0255**	**0.008861**	0.036387	0.03924	0.045119	0.028306	0.03704	**0.037218**	**0.006055**	**0.040467**	0.085322	0.053622	0.042793	0.02351	0.069508	**0.054951**	**0.023835**	**0.032123**
646.56836	CE 18:3	Cholestrol (	C45H74O2	0.002894	0.004255	0.002103	0.003838	0.00322	**0.003262**	**0.000837**	0.003828	0.003094	0.002562	0.00138	0.003388	**0.00285**	**0.000942**	**0.485884**	0.00853	0.005194	0.005094	0.003409	0.007098	**0.005865**	**0.001981**	**0.026797**
540.47521	DG 14:0-16:0	Diacylglyce	C33H64O5	0.005483	0.004764	0.002991	0.003819	0.001858	**0.003783**	**0.001431**	0.006253	0.008098	0.006488	0.004313	0.00518	**0.006066**	**0.001432**	**0.035677**	0.006198	0.005542	0.005722	0.004781	0.00791	**0.006031**	**0.001168**	**0.026197**
562.45964	DG 16:0-16:3	Diacylglyce	C35H62O5	0.002565	0.004876	0.000206	0.003564	0.002447	**0.002732**	**0.001716**	0.006138	0.005344	0.005808	0.003464	0.003926	**0.004936**	**0.001179**	**0.0454**	0.007075	0.007182	0.005215	0.004068	0.009056	**0.006519**	**0.00193**	**0.011189**
586.45927	DG 16:3-18:2	Diacylglyce	C37H62O5	0.001453	0.002978	0.001247	0.002099	0.002698	**0.002095**	**0.000754**	0.004248	0.00375	0.003831	0.002402	0.003143	**0.003475**	**0.000718**	**0.018038**	0.007158	0.003991	0.005898	0.002707	0.008597	**0.00567**	**0.002368**	**0.012303**
588.47507	DG 16:1-18:3	Diacylglyce	C37H64O5	0.006536	0.011527	0.006103	0.008677	0.007961	**0.008161**	**0.002151**	0.014223	0.013286	0.010974	0.007533	0.011823	**0.011568**	**0.002584**	**0.053232**	0.022637	0.018288	0.018829	0.012392	0.033811	**0.021192**	**0.00795**	**0.007643**
590.49043	DG 16:0-18:3	Diacylglyce	C37H66O5	0.158579	0.237725	0.145683	0.235092	0.180814	**0.191579**	**0.04282**	0.363083	0.363371	0.352826	0.204875	0.321664	**0.321164**	**0.067202**	**0.006624**	0.548184	0.392293	0.383803	0.236406	0.54541	**0.421219**	**0.130323**	**0.005679**
592.50625	DG 16:1-18:1	Diacylglyce	C37H68O5	0.191132	0.273215	0.164417	0.259107	0.191586	**0.215891**	**0.047452**	0.39117	0.401676	0.38737	0.210956	0.332549	**0.344744**	**0.07947**	**0.014383**	0.596834	0.458234	0.420397	0.26112	0.603434	**0.468004**	**0.141511**	**0.005411**
592.50635	DG 16:0-18:2	Diacylglyce	C37H68O5	0.136176	0.127377	0.193196	0.135798	0.086138	**0.135737**	**0.038183**	0.212466	0.235162	0.225717	0.117197	0.201486	**0.198406**	**0.047167**	**0.049754**	0.256324	0.184918	0.168945	0.096819	0.203838	**0.182169**	**0.057956**	**0.173029**
594.52194	DG 16:0-18:1	Diacylglyce	C37H70O5	0.038464	0.036004	0.024012	0.033351	0.023469	**0.03106**	**0.006925**	0.040909	0.047536	0.048202	0.032327	0.057719	**0.045339**	**0.009424**	**0.025838**	0.050688	0.048143	0.050109	0.026828	0.059603	**0.047074**	**0.012148**	**0.0336**
604.50606	DG 17:0-18:3	Diacylglyce	C38H68O5	0.002022	0.002517	0.001523	0.002236	0.001362	**0.001932**	**0.000483**	0.003233	0.003439	0.003278	0.001787	0.002867	**0.002921**	**0.000668**	**0.027807**	0.004922	0.004546	0.003531	0.00184	0.00492	**0.003952**	**0.00131**	**0.011994**
612.47497	DG 18:3-18:3	Diacylglyce	C39H64O5	0.081813	0.08439	0.09817	0.093448	0.065005	**0.084565**	**0.012789**	0.171158	0.137837	0.15053	0.080343	0.123209	**0.132616**	**0.034112**	**0.018446**	0.208031	0.140302	0.152928	0.075	0.170291	**0.149311**	**0.048746**	**0.02074**
614.49064	DG 18:2-18:3	Diacylglyce	C39H66O5	0.037203	0.056603	0.031578	0.05958	0.044701	**0.045933**	**0.012082**	0.082984	0.078889	0.08208	0.052043	0.076159	**0.074431**	**0.012804**	**0.006785**	0.142784	0.092479	0.088806	0.057785	0.14528	**0.105427**	**0.037741**	**0.009975**
618.52191	DG 18:1-18:2	Diacylglyce	C39H70O5	0.020601	0.028317	0.018402	0.025437	0.020223	**0.022596**	**0.004123**	0.030106	0.031342	0.031247	0.021062	0.029824	**0.028716**	**0.004332**	**0.051394**	0.048948	0.035782	0.034097	0.022713	0.049797	**0.038267**	**0.01132**	**0.019626**
622.55259	DG 18:0-18:1	Diacylglyce	C39H74O5	0.008303	0.007134	0.004215	0.005498	0.00382	**0.005794**	**0.001909**	0.009296	0.008771	0.009006	0.006338	0.015581	**0.009798**	**0.003441**	**0.052411**	0.010485	0.009684	0.007905	0.00619	0.012522	**0.009357**	**0.002425**	**0.032536**
668.53968	DG 16:0-24:6	Diacylglyce	C43H72O5	0.005297	0.00684	0.000206	0.004156	0.007946	**0.004889**	**0.002991**	0.005935	0.001888	0.001909	0.008658	0.003816	**0.004441**	**0.002887**	**0.815598**	0.029187	0.005012	0.021364	0.013531	0.024174	**0.018654**	**0.009502**	**0.014894**
704.63117	DG 18:2-24:0	Diacylglyce	C45H84O5	0.002239	0.003403	0.000206	0.00287	0.002642	**0.002272**	**0.001229**	0.004676	0.004028	0.00412	0.002362	0.00453	**0.003943**	**0.000925**	**0.041183**	0.006584	0.004759	0.005262	0.002707	0.007088	**0.00528**	**0.001722**	**0.013005**
724.60225	DG 18:0-26:6	Diacylglyce	C47H80O5	0.014429	0.01387	0.000206	0.007841	0.009944	**0.009258**	**0.005754**	0.018924	0.005346	0.004626	0.013383	0.010923	**0.01064**	**0.005925**	**0.71793**	0.046145	0.007539	0.031372	0.017083	0.030705	**0.026569**	**0.014794**	**0.040661**
699.56413	Glc-O-t18:1h16:0	Glycosylce	C40H77NC	0.005185	0.008689	0.004782	0.007183	0.006311	**0.00643**	**0.001578**	0.009227	0.008565	0.007761	0.004692	0.007453	**0.007539**	**0.001736**	**0.321192**	0.015686	0.01001	0.007759	0.008252	0.012378	**0.010817**	**0.003268**	**0.026951**
731.55404	Glc t18:1h16:0	Glycosylce	C40H77NC	0.037914	0.049953	0.026347	0.042617	0.030587	**0.037483**	**0.009402**	0.066444	0.070032	0.065152	0.038125	0.066204	**0.061191**	**0.013025**	**0.010859**	0.084316	0.064498	0.055417	0.03297	0.074471	**0.062334**	**0.019655**	**0.034155**
787.61636	Glc t18:1h20:0	Glycosylce	C44H85NC	0.008554	0.013474	0.00846	0.011355	0.007916	**0.009952**	**0.002382**	0.020046	0.015508	0.015359	0.008323	0.017279	**0.015303**	**0.004335**	**0.041916**	0.039282	0.017004	0.01654	0.01116	0.020486	**0.020895**	**0.010807**	**0.057979**
799.65293	Glc d18:1h22:0	Glycosylce	C46H89NC	0.001877	0.003613	0.000206	0.003411	0.002891	**0.0024**	**0.001398**	0.001173	0.001607	0.001722	0.000878	0.001776	**0.001431**	**0.000389**	**0.174083**	0.003672	0.005552	0.005655	0.003877	0.007811	**0.005313**	**0.001671**	**0.017335**
815.6479	Glc t18:1h22:0	Glycosylce	C46H89NC	0.05355	0.074872	0.049516	0.065615	0.050523	**0.058815**	**0.011034**	0.104929	0.098096	0.091624	0.046746	0.085008	**0.08528**	**0.022779**	**0.047559**	0.17859	0.110998	0.106646	0.065815	0.133776	**0.119165**	**0.041275**	**0.013425**
825.66826	Glc d18:2h24:0	Glycosylce	C48H91NC	0.016309	0.020606	0.014083	0.019516	0.013786	**0.01686**	**0.003105**	0.029342	0.024546	0.0233	0.011747	0.021763	**0.022139**	**0.006464**	**0.138352**	0.041281	0.026433	0.025991	0.01524	0.028258	**0.027441**	**0.009276**	**0.041941**
839.6484	Glc t18:3h24:0	Glycosylce	C48H89NC	0.004153	0.005179	0.002808	0.005209	0.003225	**0.004115**	**0.001099**	0.008644	0.007693	0.009074	0.004329	0.006883	**0.007324**	**0.001878**	**0.010881**	0.016119	0.007223	0.007301	0.004798	0.010464	**0.009181**	**0.004369**	**0.036108**
841.6634	Glc t18:2h24:0	Glycosylce	C48H91NC	0.190339	0.291457	0.139651	0.292852	0.198113	**0.222482**	**0.067449**	0.406464	0.398087	0.38258	0.193872	0.333588	**0.342918**	**0.087973**	**0.041224**	0.551648	0.366834	0.340789	0.214768	0.437498	**0.382307**	**0.124253**	**0.035376**
857.69419	Glc t18:1h25:0	Glycosylce	C49H95NC	0.004224	0.006396	0.002717	0.005387	0.004497	**0.004644**	**0.001372**	0.010612	0.010177	0.011406	0.005096	0.011088	**0.009676**	**0.002602**	**0.005058**	0.015371	0.009257	0.007923	0.003755	0.009094	**0.00908**	**0.004162**	**0.053459**
869.69429	Glc t18:2h26:0	Glycosylce	C50H95NC	0.033228	0.039114	0.027795	0.03865	0.026323	**0.033022**	**0.005938**	0.069427	0.066057	0.064752	0.034256	0.061968	**0.059292**	**0.01425**	**0.005199**	0.071955	0.060825	0.049813	0.02722	0.05824	**0.053611**	**0.01674**	**0.032018**

495.33226	LPC 16:0	Lysophospl	C24H50NC	0.004407	0.003633	0.003344	0.002015	0.001249	0.002929	0.001276	0.002029	0.00336	0.003968	0.003082	0.002408	0.00297	0.000768	0.953453	0.000116	0.002691	9.09E-05	0.001085	8.68E-05	0.000814	0.001133	0.024221
517.31684	LPC 18:3	Lysophospl	C26H48NC	0.003983	0.003569	0.003742	0.003536	0.001719	0.00331	0.000907	0.004775	0.004752	0.005832	0.005061	0.00496	0.005076	0.000442	0.004452	0.0059	0.005112	0.005001	0.0028	0.005646	0.004892	0.001227	0.04903
521.34783	LPC 18:1	Lysophospl	C26H52NC	0.001941	0.001552	0.001514	0.001584	0.000986	0.001515	0.000342	9.79E-05	9.27E-05	0.00173	0.00104	0.001015	0.000795	0.0007	0.072632	0.000116	0.001346	9.09E-05	0.000648	8.68E-05	0.000458	0.000551	0.006517
720.4932	PG 16:0-16:1	Phosphatid	C38H73O1	0.001352	6.68E-05	0.00218	0.002759	0.003435	0.001959	0.001306	9.79E-05	9.27E-05	0.002946	0.003425	0.002338	0.00178	0.001585	0.85049	0.000116	8.25E-05	9.09E-05	0.001283	8.68E-05	0.000332	0.000532	0.032616
722.5105	PG 16:0-16:0	Phosphatid	C38H75O1	0.002923	0.003113	0.002177	0.002723	0.002863	0.00276	0.000355	0.002735	9.27E-05	0.002735	0.003303	0.002761	0.002325	0.001271	0.482926	0.002621	0.002207	0.001223	0.001996	0.001806	0.001971	0.000516	0.022645
749.50235	PC 16:3-18:3	Phosphoch	C42H72NC	0.001762	0.002987	0.001788	0.002381	0.001908	0.002165	0.000523	0.00463	0.005694	0.005316	0.003539	0.004758	0.004787	0.000819	0.000312	0.005514	0.004633	0.003382	0.002039	0.006616	0.004437	0.00179	0.026064
755.54582	PC 16:0-18:3	Phosphoch	C42H78NC	0.072179	0.096718	0.071798	0.081434	0.054174	0.07526	0.015529	0.122741	0.150275	0.142084	0.123325	0.124688	0.132623	0.01273	0.000212	0.135518	0.118916	0.053261	0.042443	0.13276	0.09658	0.045087	0.34674
757.56143	PC 16:0-18:2	Phosphoch	C42H80NC	0.107913	0.13105	0.115509	0.101928	0.07354	0.105988	0.021165	0.141683	0.169158	0.152283	0.151101	0.147666	0.152378	0.010244	0.002252	0.151468	0.152112	0.070364	0.058656	0.148857	0.116291	0.047466	0.669295
767.55385	PC 17:1-18:3	Phosphoch	C43H78NC	0.022844	0.045965	0.023197	0.038349	0.029825	0.032036	0.010017	0.061227	0.054465	0.052844	0.031497	0.054248	0.050856	0.011301	0.023676	0.105048	0.056249	0.061481	0.054531	0.107962	0.077054	0.027026	0.008168
777.53029	PC 18:3-18:3	Phosphoch	C44H76NC	0.05605	0.08108	0.061785	0.069154	0.047123	0.063039	0.012909	0.127563	0.146103	0.148803	0.105673	0.126186	0.130866	0.017474	0.000115	0.113748	0.09641	0.064398	0.048312	0.131345	0.090843	0.034294	0.128193
779.54366	PC 18:0-18:5	Phosphoch	C44H78NC	0.017029	0.018883	0.017733	0.014883	0.010706	0.015847	0.003222	0.020969	0.027526	0.02495	0.024777	0.022025	0.024049	0.002599	0.002195	0.021449	0.024023	0.009411	0.007123	0.021206	0.016642	0.007767	0.83778
779.54584	PC 18:2-18:3	Phosphoch	C44H78NC	0.074435	0.110862	0.080771	0.091399	0.063484	0.08419	0.018021	0.151863	0.171104	0.16654	0.147897	0.152525	0.157986	0.010178	4.47E-05	0.132673	0.117224	0.060754	0.05305	0.146404	0.102021	0.042549	0.413334
781.56135	PC 18:2-18:2	Phosphoch	C44H80NC	0.035651	0.037637	0.040589	0.035652	0.020969	0.0341	0.007613	0.050492	0.057852	0.054283	0.038589	0.049569	0.050157	0.007255	0.009166	0.039071	0.041456	0.022784	0.017151	0.043025	0.032698	0.011874	0.829654
785.59286	PC 18:1-18:1	Phosphoch	C44H84NC	0.026385	0.032177	0.013331	0.027004	0.018047	0.023389	0.007566	0.030849	0.039047	0.033534	0.032134	0.030514	0.033216	0.003471	0.02973	0.035905	0.036172	0.014723	0.012798	0.03467	0.026853	0.011985	0.599577
788.47478	SQDG 16:0-16:3	Sulfoquino	C41H72O1	7.85E-05	0.001985	0.000206	0.000862	0.000943	0.000815	0.000758	0.002004	0.001852	0.001372	0.001927	0.001544	0.00174	0.00027	0.033203	0.002092	0.001236	0.0011	4.85E-05	0.001666	0.001228	0.000766	0.415978
838.48924	SQDG 18:3-18:3	Sulfoquino	C45H74O1	0.01408	0.028827	0.016551	0.015222	0.014812	0.017898	0.006175	0.034739	0.0263	0.025157	0.025715	0.0243	0.027242	0.004255	0.0237	0.048043	0.024952	0.02135	0.014643	0.039889	0.029775	0.013777	0.116617
772.65725	TG 14:1-16:1-16:1	Triacylglyce	C49H88O6	0.01429	0.0091	0.005687	0.00794	0.004454	0.008294	0.003817	0.013573	0.018643	0.014137	0.008223	0.011077	0.013131	0.00387	0.081813	0.013607	0.011654	0.0146	0.011676	0.019065	0.01412	0.003042	0.028393
774.67294	TG 14:1-16:0-16:1	Triacylglyce	C49H90O6	0.061848	0.030371	0.022551	0.033155	0.017182	0.033021	0.01731	0.051692	0.075357	0.064572	0.031031	0.045934	0.053717	0.017069	0.09345	0.051481	0.047107	0.062419	0.04537	0.079657	0.057207	0.014196	0.042128
788.68856	TG 15:1-16:0-16:1	Triacylglyce	C50H92O6	0.05471	0.029645	0.020059	0.030588	0.015468	0.030094	0.015174	0.055545	0.067506	0.058778	0.028018	0.042365	0.050442	0.01545	0.06882	0.045079	0.041215	0.05723	0.040387	0.073317	0.051446	0.013958	0.049249
798.67278	TG 14:1-16:1-18:2	Triacylglyce	C51H90O6	0.003571	0.002187	0.001569	0.002446	0.001253	0.002205	0.000899	0.003571	0.005231	0.004507	0.002341	0.003231	0.003776	0.001124	0.040516	0.003958	0.003098	0.004949	0.003188	0.005886	0.004216	0.001194	0.016851
822.67287	TG 16:0-16:3-18:3	Triacylglyce	C53H90O6	7.85E-05	0.002412	0.001756	0.003742	0.00189	0.001976	0.00132	0.005191	0.004166	0.005468	0.003399	0.002917	0.004228	0.001104	0.01907	0.009966	0.008389	0.009305	0.003726	0.00923	0.008123	0.002521	0.001303
826.70399	TG 16:0-16:1-18:3	Triacylglyce	C53H94O6	0.011499	0.007234	0.005301	0.007375	0.004365	0.007155	0.002745	0.011294	0.015667	0.014519	0.007761	0.011108	0.01207	0.003124	0.029584	0.01556	0.012456	0.01434	0.00959	0.017086	0.013806	0.002903	0.005851
828.71972	TG 16:0-16:1-18:2	Triacylglyce	C53H96O6	0.071092	0.036568	0.025578	0.040692	0.022908	0.039368	0.019216	0.068546	0.09366	0.071384	0.043869	0.057332	0.066958	0.018445	0.049211	0.06948	0.063963	0.076577	0.04861	0.095193	0.070765	0.017097	0.025866
830.73549	TG 16:0-16:1-18:1	Triacylglyce	C53H98O6	0.149704	0.085812	0.048865	0.086207	0.049482	0.084014	0.041083	0.171986	0.181835	0.170015	0.084786	0.129247	0.147574	0.040471	0.039044	0.134621	0.125586	0.150839	0.109953	0.197186	0.143637	0.033396	0.035911
836.6796	TG 15:0-18:3-18:3	Triacylglyce	C54H92O6	7.85E-05	6.68E-05	0.001086	0.001561	0.001064	0.000771	0.000668	0.003351	0.003889	0.003244	0.001139	0.002785	0.002882	0.00105	0.005301	0.000116	0.0024	0.002721	0.001839	0.004161	0.002248	0.001468	0.074853
840.71944	TG 15:1-18:1-18:2	Triacylglyce	C54H96O6	0.006562	0.0036	0.002126	0.003594	0.00227	0.00363	0.001783	0.007024	0.008338	0.00784	0.004031	0.005851	0.006617	0.001725	0.027389	0.007749	0.006693	0.00765	0.005516	0.008933	0.007308	0.001279	0.005635
844.65715	TG 16:3-18:3-18:3	Triacylglyce	C55H88O6	0.004345	0.004045	0.006115	0.005943	0.004146	0.004919	0.001021	0.007963	0.007726	0.006394	0.00673	0.007567	0.007276	0.000677	0.002612	0.013292	0.008986	0.010388	0.005224	0.01508	0.010594	0.003836	0.012679
848.68839	TG 16:1-18:3-18:3	Triacylglyce	C55H92O6	0.00945	0.011461	0.007804	0.012051	0.008252	0.009804	0.001893	0.019164	0.018114	0.016679	0.010824	0.016214	0.016199	0.003224	0.005053	0.024385	0.019808	0.019313	0.010898	0.02776	0.020433	0.006361	0.007178
850.70385	TG 16:0-18:3-18:3	Triacylglyce	C55H94O6	0.019562	0.023531	0.022813	0.027595	0.019654	0.022631	0.003308	0.054073	0.047372	0.046084	0.029461	0.040029	0.043404	0.009254	0.00149	0.080117	0.058172	0.061773	0.031531	0.072519	0.060822	0.01854	0.001913
852.71956	TG 16:0-18:2-18:3	Triacylglyce	C55H96O6	0.045522	0.054238	0.037739	0.062622	0.043065	0.048637	0.009829	0.106137	0.103774	0.09762	0.062508	0.096282	0.093264	0.017679	0.001145	0.173384	0.127539	0.136795	0.074691	0.172309	0.136944	0.040443	0.001456
854.73521	TG 16:0-18:2-18:2	Triacylglyce	C55H98O6	0.060522	0.058892	0.033017	0.048987	0.040083	0.0483	0.011865	0.089307	0.103684	0.094292	0.059338	0.088902	0.087105	0.016625	0.002806	0.125057	0.101543	0.116998	0.069973	0.13661	0.110036	0.025775	0.001248
856.75081	TG 16:0-18:1-18:2	Triacylglyce	C55H100O	0.055918	0.039538	0.025593	0.031184	0.023225	0.035092	0.013227	0.056504	0.065826	0.065429	0.037339	0.05933	0.056885	0.011631	0.024418	0.053273	0.057946	0.055408	0.038816	0.065564	0.054201	0.009775	0.031709
858.76667	TG 16:0-18:1-18:1	Triacylglyce	C55H102O	0.108719	0.064646	0.033915	0.053877	0.036076	0.059447	0.030347	0.113713	0.105192	0.110086	0.071867	0.117595	0.103691	0.018369	0.023598	0.083898	0.088699	0.082087	0.07065	0.104068	0.08588	0.012135	0.108143
870.76643	TG 17:1-18:1-18:1	Triacylglyce	C56H102O	0.013937	0.009416	0.004216	0.006843	0.004488	0.00778	0.004031	0.014109	0.016621	0.014716	0.007291	0.01146	0.012839	0.00361	0.069958	0.011729	0.012092	0.012827	0.010317	0.016157	0.012624	0.002176	0.045627
872.68819	TG 14:3-18:3-22:3	Triacylglyce	C57H92O6	0.044768	0.059689	0.042332	0.070004	0.049198	0.053198	0.011508	0.103356	0.093437	0.09764	0.058797	0.082064	0.087059	0.01762	0.007006	0.150572	0.102989	0.11666	0.054249	0.150117	0.114917	0.0398	0.010368
874.70406	TG 18:2-18:3-18:3	Triacylglyce	C57H94O6	0.131475	0.180288	0.117224	0.199846	0.148111	0.155389	0.034197	0.316399	0.282198	0.265729	0.160035	0.246409	0.254154	0.058552	0.011579	0.417301	0.308343	0.34377	0.164907	0.453961	0.337656	0.112482	0.008484
876.71936	TG 18:2-18:2-18:3	Triacylglyce	C57H96O6	0.035805	0.042996	0.046911	0.045216	0.029195	0.040025	0.007387	0.063271	0.062629	0.058147	0.032779	0.057058	0.054777	0.012592	0.053764	0.076729	0.060145	0.069623	0.032811	0.078048	0.063471	0.018555	0.030406
876.71963	TG 18:1-18:2-18:4	Triacylglyce	C57H96O6	0.066244	0.093231	0.058534	0.100696	0.069753	0.077692	0.018247	0.139502	0.134039	0.131793	0.081723	0.126102	0.122632	0.023368	0.009508	0.20737	0.166767	0.168078	0.089012	0.243788	0.175003	0.05764	0.006992
878.73496	TG 18:2-18:2-18:2	Triacylglyce	C57H98O6	0.023735	0.03326	0.025798	0.022917	0.017797	0.024701	0.005618	0.029817	0.028924	0.031846	0.018372	0.037812	0.029354	0.007049	0.281736	0.04612	0.033823	0.04124	0.020808	0.044097	0.037218	0.01029	0.044051
878.73508	TG 18:0-18:3-18:3	Triacylglyce	C57H98O6	0.047195	0.070659	0.033883	0.05138	0.042596	0.049143	0.01367	0.058937	0.0689	0.071627	0.044465	0.082766	0.065339	0.014428	0.105896	0.108878	0.082742	0.106178	0.053455	0.119894	0.094229	0.026507	0.009637
880.75054	TG 18:1-18:2-18:2	Triacylglyce	C57H100O	0.049097	0.067911	0.022172	0.03158	0.034452	0.041042	0.01786	0.050637	0.056972	0.059838	0.037879	0.070579	0.055181	0.012062	0.180571	0.080383	0.064802	0.085149	0.043256	0.080176	0.070753	0.017177	0.027881
882.76616	TG 18:1-18:1-18:2	Triacylglyce	C57H102O	0.053082	0.056316	0.020444	0.028878	0.031469	0.038038	0.015788	0.05553	0.055871	0.060624	0.03519	0.066922	0.054827	0.011907	0.094178	0.06214	0.062528	0.065981	0.037268	0.068545	0.059292	0.012589	0.046416

									**Average**	**Stdev**						**Average**	**Stdev**	**t-test**						**Average**	**Stdev**	**t-test**
Mass (M)	Name	Compound	FORMULA (	Col-0-R1	Col-0-R2	Col-0-R3	Col-0-R4	Col-0-R5			kac1/2-R1	kac1/2-R2	kac1/2-R3	kac1/2-R4	kac1/2-R5				chup1-R1	chup1-R2	chup1-R3	chup1-R4	chup1-R5			
774.45519	Arabidopside A	Arabidopsi	C43H66O1	0.068703	0.044463	0.098902	0.086402	0.052251	**0.070144**	**0.022766**	0.241174	0.194889	0.24499	0.158533	0.166467	**0.201211**	**0.040565**	**0.000233**	0.293636	0.145723	0.286653	0.177236	0.131633	**0.206976**	**0.077736**	**0.005409**
802.4869	Arabidopside B	Arabidopsi	C45H70O1	0.001383	0.000824	0.000937	0.002338	0.000557	**0.001208**	**0.000698**	0.005172	0.006581	0.008372	0.006007	0.006729	**0.006572**	**0.001177**	**2.25E-05**	0.00554	0.005052	0.007936	0.004376	9.26E-05	**0.004599**	**0.002854**	**0.032568**
555.52274	Cer d16:0h18:0	Ceramides	C34H69NC	0.016199	0.016859	0.032023	0.035147	0.017912	**0.023628**	**0.009177**	0.013851	0.016495	0.023027	0.0094	0.010218	**0.014598**	**0.005509**	**0.095951**	0.009831	0.008179	0.01602	0.009928	0.01263	**0.011318**	**0.003075**	**0.021669**
655.61099	Cer t18:0h22:0	Ceramides	C40H81NC	0.019954	0.012829	0.030271	0.035143	0.023927	**0.024425**	**0.008714**	0.024029	0.021559	0.031478	0.012999	0.015042	**0.021021**	**0.007396**	**0.52424**	0.01176	0.010556	0.020903	0.013613	0.013441	**0.014054**	**0.004031**	**0.042168**
584.44385	DG 16:3-18:3	Diacylglyce	C37H60O5	0.010223	0.009537	0.010395	0.022066	0.014543	**0.013353**	**0.005255**	0.007341	0.007542	0.011096	0.00584	0.004516	**0.007267**	**0.002467**	**0.047092**	0.00987	0.008801	0.015776	0.014263	0.024207	**0.014584**	**0.00612**	**0.741807**
720.4932	PG 16:0-16:1	Phosphatid	C38H73O1	0.004872	0.002306	0.000102	1E-04	8.81E-05	**0.001494**	**0.002117**	0.000117	0.003358	0.000108	0.002714	0.002273	**0.001714**	**0.001512**	**0.854412**	0.00437	0.003661	0.006052	0.004384	0.002401	**0.004174**	**0.001324**	**0.043195**
757.56143	PC 16:0-18:2	Phosphoch	C42H80NC	0.206587	0.097223	0.116385	0.128173	0.116481	**0.13297**	**0.042624**	0.139212	0.138603	0.15786	0.083158	0.071592	**0.118085**	**0.03818**	**0.576824**	0.244979	0.153252	0.221622	0.163104	0.198859	**0.196363**	**0.038641**	**0.039083**
779.54366	PC 18:0-18:5	Phosphoch	C44H78NC	0.030848	0.013651	0.015749	0.01819	0.016137	**0.018915**	**0.006863**	0.020877	0.020302	0.021078	0.011874	0.010548	**0.016936**	**0.005255**	**0.622388**	0.041931	0.024957	0.032296	0.026072	0.030864	**0.031224**	**0.00674**	**0.021107**
781.56135	PC 18:2-18:2	Phosphoch	C44H80NC	0.051435	0.027446	0.038972	0.044377	0.041858	**0.040818**	**0.008785**	0.051829	0.042827	0.047311	0.027243	0.02613	**0.039068**	**0.011749**	**0.796414**	0.089049	0.049199	0.069096	0.051406	0.062609	**0.064272**	**0.016067**	**0.021023**
783.57747	PC 18:1-18:2	Phosphoch	C44H82NC	0.034218	0.017873	0.021109	0.025269	0.020146	**0.023723**	**0.006449**	0.025038	0.025033	0.039704	0.015171	0.013268	**0.023643**	**0.010503**	**0.988759**	0.042546	0.026132	0.04046	0.027924	0.034353	**0.034283**	**0.007303**	**0.041617**
785.59286	PC 18:1-18:1	Phosphoch	C44H84NC	0.038947	0.01756	0.024589	0.029606	0.022948	**0.02673**	**0.00807**	0.029598	0.030527	0.040749	0.020656	0.017807	**0.027867**	**0.009072**	**0.839351**	0.04782	0.033669	0.056296	0.036289	0.041269	**0.043068**	**0.009153**	**0.01723**
687.48343	PE 16:1-16:1	Phosphoetl	C37H70NC	7.84E-05	0.012104	0.00585	0.003139	0.003184	**0.004871**	**0.00453**	0.000117	0.004255	0.003671	0.00007	0.018225	**0.005268**	**0.0075**	**0.921877**	0.000134	6.87E-05	0.000117	9.28E-05	9.26E-05	**0.000101**	**2.51E-05**	**0.046355**
741.53284	PE 18:1-18:2	Phosphoetl	C41H76NC	7.84E-05	0.004519	0.003894	0.005379	0.004893	**0.003753**	**0.002125**	0.000117	0.000125	0.007499	0.002953	0.003053	**0.002749**	**0.003021**	**0.560346**	0.000134	0.003699	0.000117	9.28E-05	9.26E-05	**0.000827**	**0.001606**	**0.039529**
299.28142	Sphingosine d18:1	Sphingosin	C18H37NC	0.002451	0.002146	0.003142	0.002984	8.81E-05	**0.002162**	**0.001227**	0.000117	0.000125	0.000108	0.002712	6.84E-05	**0.000626**	**0.001166**	**0.076961**	0.000134	6.87E-05	0.000117	9.28E-05	9.26E-05	**0.000101**	**2.51E-05**	**0.005579**
836.6796	TG 15:0-18:3-18:3	Triacylglyce	C54H92O6	0.00319	0.002844	0.003958	0.005506	0.002233	**0.003546**	**0.001261**	0.001828	0.000125	0.00231	0.002993	6.84E-05	**0.001465**	**0.001316**	**0.033948**	0.000134	6.87E-05	0.000117	0.003132	0.003206	**0.001331**	**0.001678**	**0.045963**
934.79564	TG 18:2-18:3-22:1	Triacylglyce	C61H106O	0.004219	0.00673	0.004903	0.01808	0.005935	**0.007973**	**0.005731**	0.002499	0.002112	0.000108	0.00138	0.00172	**0.001563**	**0.000915**	**0.038734**	0.00292	0.002717	0.005587	0.002873	0.003689	**0.003557**	**0.001195**	**0.130152**
