# Supplementary material for: Size of chloroplasts in Arabidopsis mesophyll cells affects jasmonate biosynthesis
Source: Plant Biol (Stuttg). 2026 May 11;28(5):1330–42. doi: 10.1111/plb.70222 (PMC13358708; doi:10.1111/plb.70222)
Supplement: Supplementary file 1 — Fig. S1. Ratio of MGDG and DGDG in rosette leaves from Ler, arc3 and arc5. Fig. S2. Hierarchical clustering of lipid profiles from kac1/2, chup1 and their wild‐type Col‐0. Fig. S3. Leaf morphology of Ler and arc‐mutants. Fig. S4. Western blot analysis of AOC protein levels in chloroplast mutants. Fig. S5. Touch treatment does not alter rosette area. [file PLB-28-1330-s001.zip › PLB_70222__pe.pdf]

Supplementary figures to:

Size of chloroplasts in Arabidopsis mesophyll cells affects jasmonate biosynthesis

by

Ranjit Baral, Hagen Stellmach, Samuel Ngure Kariithi, Mareike Heilmann, Stephanie Krüger, Joerg Ziegler, Bettina Hause

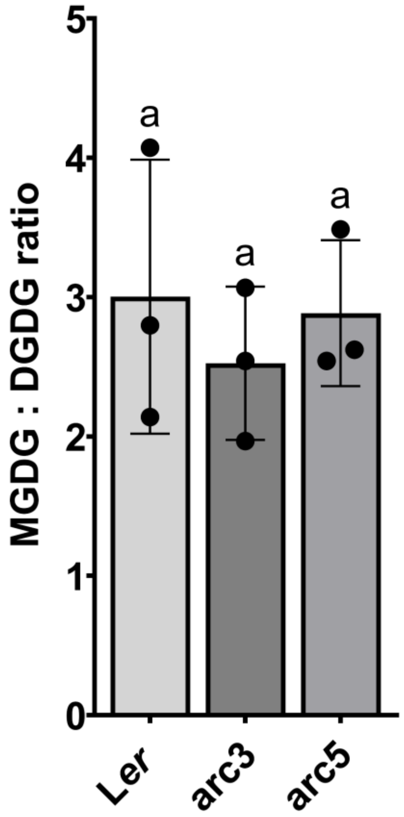

Figure S1. Ratio of MGDG and DGDG in rosette leaves from L*er*, *arc3* and *arc5*.

Ratio of MGDG to DGDG content in wild-type L*er*, *arc3*, and *arc5* plants calculated from total galactolipid quantification. Bars represent means and error bars represent standard deviation (n = 3-4 biological replicates). Different letters denote statistically significant differences according to one-way ANOVA followed by Tukey's multiple comparison test ($p < 0.05$).

(a)

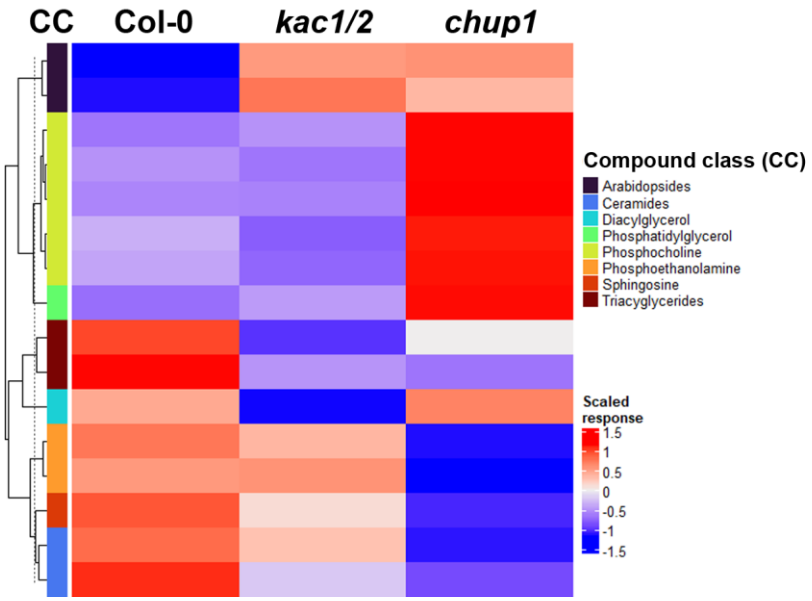

(b)

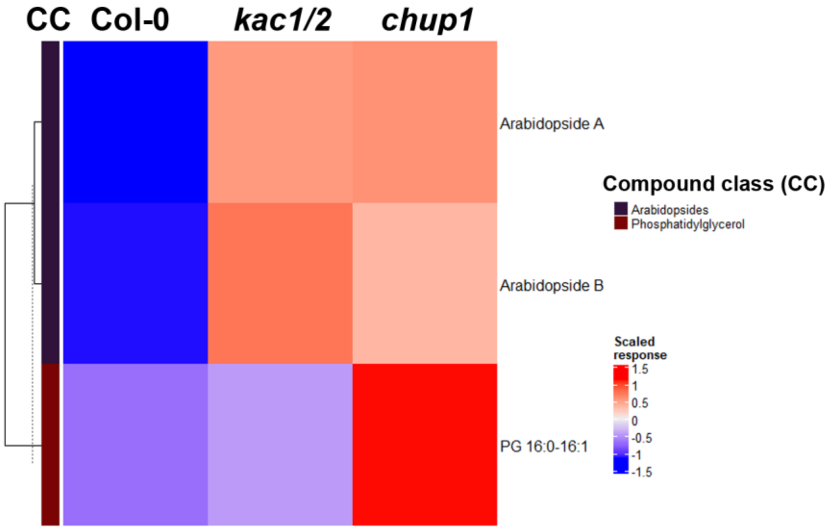

Figure S2. Hierarchical clustering of lipid profiles from *kac1/2*, *chup1* and their wild type Col-0.
(a) Heatmap showing hierarchical clustering of all lipid compound classes (CC) significantly changed across wild-type Col-0 as well as *kac1/2*, and *chup1* mutants (*n* = 5 biological replicates per genotype). Lipid analysis methods as described in Figure 3.
(b) Heatmap showing individual molecular species showing significant changes between wild type and mutants and were annotated with acyl chain compositions (carbon number:number of double bonds).
Compound classes are indicated by color-coded bars in the right margin and are displayed as row-wise z-score normalized abundances as defined in “Scaled response” at the right.

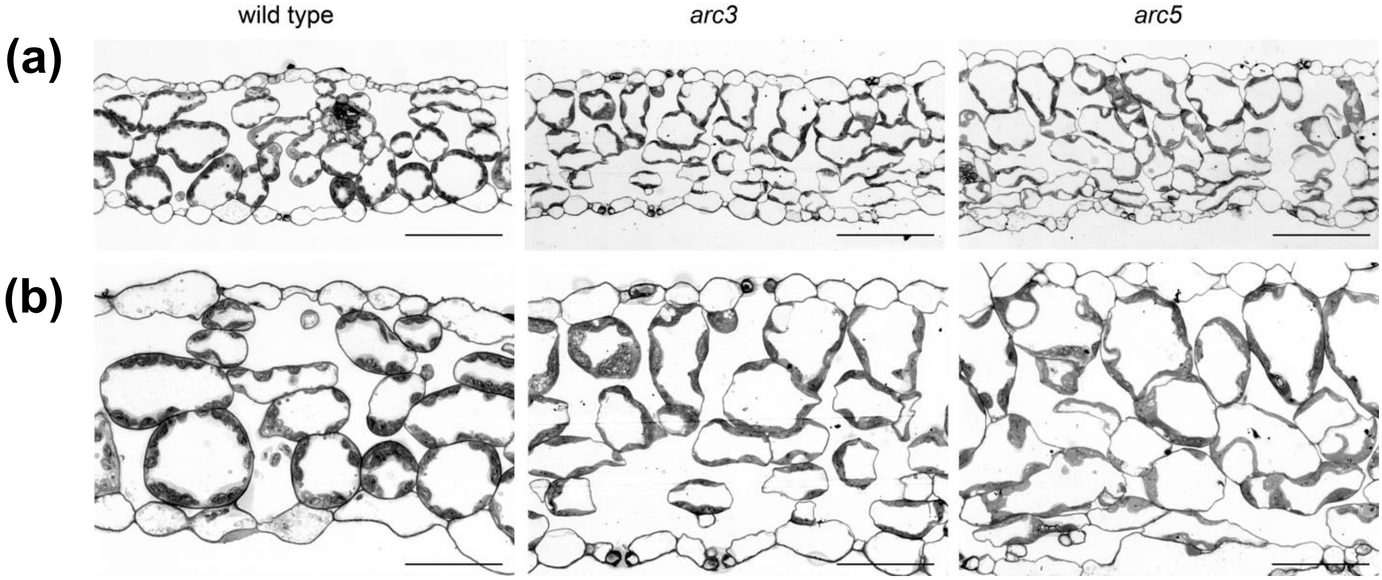

Figure S3. Leaf morphology of L*er* and *arc*-mutants.
Bright field microscopy images of leaf cross-sections showing leaf morphology (a) and chloroplast distribution in mesophyll cells (b) of wild-type L*er*, *arc3*, and *arc5* plants. Note that overall leaf morphology is similar in all three genotypes. Wild-type mesophyll cells contain numerous small, uniformly distributed chloroplasts, whereas *arc* mutant mesophyll cells exhibit reduced chloroplast numbers with enlarged individual plastids. Scale bars indicate 100 µm in (a) and 50 µm in (b).

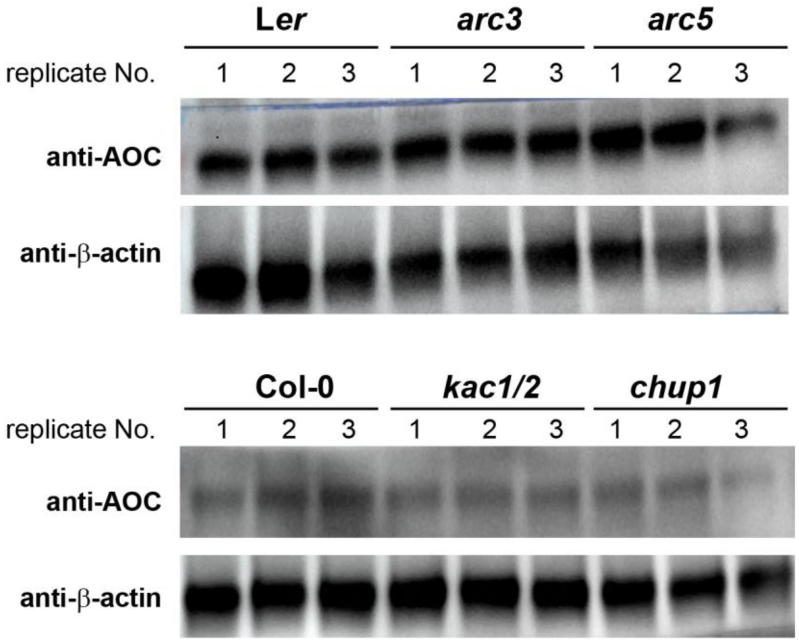

Figure S4. Western blot analysis of AOC protein levels in chloroplast mutants.

Representative Western blots showing AOC protein abundance in L*er*, *arc3*, *arc5* (upper panel) and Col-0, *kac1/2*, *chup1* (lower panel) with three independent biological replicates per genotype. β-actin serves as loading control. Quantification and statistical analysis are presented in Figure 5.

(a)

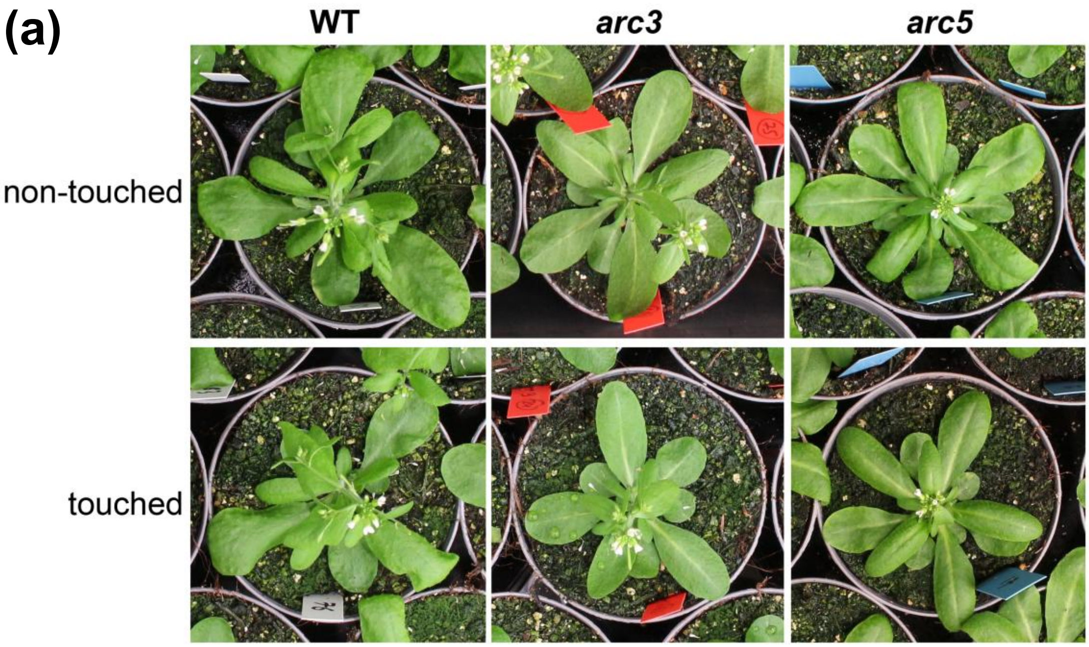

(b)

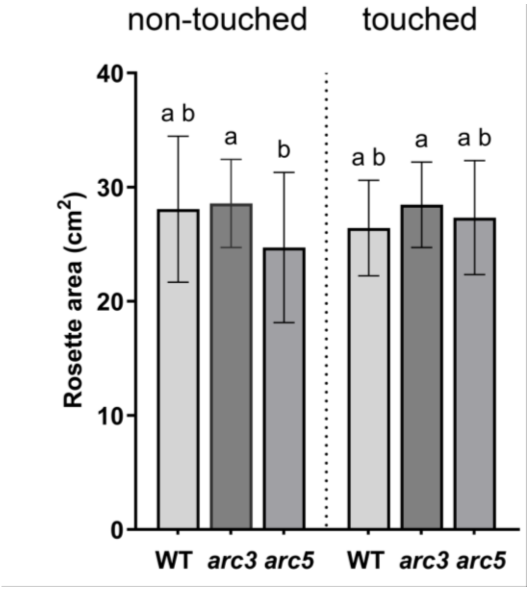

Figure S5. Touch treatment does not alter rosette area

(a) Representative images of wild-type Ler (WT), *arc3*, and *arc5* plants under non-touched (upper panels) and touched (lower panels) conditions at bolting time. Beginning at 14 days after sowing (DAS), rosettes were either left non-touched or gently touched twice daily with a soft brush until bolting.

(b) Bar graphs showing rosette area (cm^2) in non-touched and touched plants (non-touched, n = 33; touched, n = 65). Data are presented as means ± SE. Different letters indicate statistically significant differences ($p < 0.05$) according to one-way ANOVA followed by Tukey's HSD test.
